# Integrated analysis of mRNA-seq and miRNA-seq reveals the potential roles of sex-biased miRNA-mRNA pairs in gonad tissue of dark sleeper *(Odontobutis potamophila)*

**DOI:** 10.1186/s12864-017-3995-9

**Published:** 2017-08-14

**Authors:** Cheng Zhao, Guosong Zhang, Shaowu Yin, Zecheng Li, Qintao Wang, Shuqiao Chen, Guoqin Zhou

**Affiliations:** 1 College of Life Sciences, Key Laboratory of Biodiversity and Biotechnology of Jiangsu Province, Nanjing Normal University, Nanjing, Jiangsu 210023 China; 2 Co-Innovation Center for Marine Bio-Industry Technology of Jiangsu Province, Lianyungang, Jiangsu 222005 China; 3 Nanjing Institute of Fisheries Science, Nanjing, Jiangsu 210036 China

**Keywords:** Dark sleeper, Gonads, miRNA, mRNA, Sex determination, Sex differentiation

## Abstract

**Background:**

The dark sleeper (*Odontobutis potamophila*) is an important commercial fish species which shows a sexually dimorphic growth pattern. However, the lack of sex transcriptomic data is hindering further research and genetically selective breeding of the dark sleeper. In this study, integrated analysis of mRNA and miRNA was performed on gonad tissue to elucidate the molecular mechanisms of sex determination and differentiation in the dark sleeper.

**Results:**

A total of 143 differentially expressed miRNAs and 16,540 differentially expressed genes were identified. Of these, 8103 mRNAs and 75 miRNAs were upregulated in testes, and 8437 mRNAs and 68 miRNAs were upregulated in ovaries. Integrated analysis of miRNA and mRNA expression profiles predicted more than 50,000 miRNA-mRNA interaction sites, and among them 27,583 negative miRNA-mRNA interactions. A number of sex related genes were targeted by sex-biased miRNAs. The relationship between 15 sex-biased genes and 15 sex-biased miRNAs verified by using qRT-PCR were described. Additionally, a number of SNPs were revealed through the transcriptome data.

**Conclusions:**

The overall results of this study facilitate our understanding of the molecular mechanism underlying sex determination and differentiation and provide valuable genomic information for selective breeding of the dark sleeper.

**Electronic supplementary material:**

The online version of this article (doi:10.1186/s12864-017-3995-9) contains supplementary material, which is available to authorized users.

## Background

Teleost fishes exhibit remarkably diverse and plastic sexual development patterns [1], with fish sex determined either by genetics (i.e., genetic sex determination, or GSD) or by environment (i.e., environmental sex determination, or ESD). However, individuals with GSD can also be influenced by many external variables, primarily temperature—that is, GSD + EE (environmental effects) [2]. Despite the various forms of sex determination found in fishes, it is theorized that the genes involved have been well preserved throughout time. Several molecular factors were reported to be responsible for sex determination in gonochoristic species, such as *Dmy* in medaka [3], *Amhr2* in tiger pufferfish [4], *Sdy* in rainbow trout [5], *Amhy* in Patagonian pejerrey [6], and *Dmrt1* in half-smooth tongue sole [7]. These studies all detail the large diversity in sex determination. Generally speaking, sex determination and differentiation in fishes is a challenge because of the wide variety of species and much has yet to be learned about their molecular mechanisms.

The dark sleeper *(Odontobutis potamophila)* is a gonochoristic species widely distributed in the river systems of southeastern China [8]. It is an important commercial fish species due to its delicious taste and high nutritional value, therefore in recent years the dark sleeper has become a very promising aquaculture species in China. A previous survey on dark sleepers found that the male individual grows substantially larger and at a quicker rate than the female [9]. Thus, male individuals have more economic value than females. However, there has been little research addressing genetically selective breeding of the dark sleeper due to the dearth of genomic and transcriptomic resources available and since few of its sequences have been deposited in the NCBI GenBank. Therefore, it is important to understand the regulatory mechanisms of sex determination and gonadal differentiation in this species and enlarge the public EST database. In order to understand these mechanisms in detail at the molecular level and to provide a theoretical basis for selective breeding, the regulatory mechanisms associated with gonadal differences need to be studied at the genomic level.

RNA-seq represents an effective means of providing information about gene expression levels, information which has been widely used for analyzing the variety of expressed genes and demonstrating key genes which are expressed at a particular moment. For instance, the use of RNA-seq analysis has helped researchers to understand the molecular mechanisms underlying sex determination and differentiation and to identify differentially expressed genes in rainbow trout (*Oncorhynchu smykiss*) [10], Nile tilapia(*Oreochromis niloticus*) [11], yellow catfish (*Pelteobagrus fulvidraco*) [12, 13], and turbot (*Scophthalmus maximus*) [14]. These transcriptomic data have provided information on genes in gonads in specific conditions and identified sex-differentially expressed genes.

MicroRNAs (miRNAs) are small noncoding RNA molecules (18–22 nucleotides) which post-transcriptionally regulate gene expression [15, 16]. Most mature miRNA sequences are conserved among fish, amphibians, birds and mammals [17]. Studies have suggested that miRNAs play a crucial role in many biological processes, including metabolism, cell proliferation, differentiation, apoptosis, and developmental timing [18]. Recently, miRNA expression has been examined in the gonads of yellow catfish [19], Nile tilapia [20], and olive flounder [21] by using deep RNA-sequencing technology. These studies suggested that miRNAs are important for sex differentiation and sexual development. In addition, miRNAs are able to target numerous genes, making them a probable regulator in biological processes. Thus, gaining insight into miRNA-mRNA regulatory networks enables a greater understanding of gene expression at the post-transcriptional level [22]. Additional research related to miRNA and mRNA co-expression has also recently been conducted, focusing on the comprehensive network of post-transcriptional regulations [23–25].

In this study, an integration analysis was conducted in order to identify target genes of miRNAs and characterize their functional roles in gonad tissues of the dark sleeper. These findings provide us with further insight into the regulatory mechanisms associated with sex determination and gonadal differentiation. We used bioinformatics analysis and miRNA prediction algorithms in order to find miRNA-mRNA pairs. Real-time PCR was used to validate the differences between testes and ovaries for various differentially expressed genes and miRNAs. Through this research, we identified a large number of SNPs that will facilitate genetic research and selective breeding of this species, which have so far been hindered by a lack of genomic data. The findings of this study facilitate our understanding of the molecular mechanism underlying sex determination and differentiation and provide valuable genomic information for selective breeding of the dark sleeper.

## Methods

### Sample preparation and RNA extraction

Nine one-year-old male (35 ± 6.34 g) and nine one-year-old female (22 ± 5.65 g) dark sleepers were obtained from Nanjing Fisheries Research Institute, Jiangsu Province, China on February 16, 2016, before the breeding season. These dark sleepers were temporarily cultured in aquaria with aeration for two weeks in order to acclimatize them to laboratory conditions before they were dissected. The temperature and PH of the freshwater in the aquaria were kept at 22–24 °C and 7.2–7.4, respectively. Sequential feed restrictions were utilized for two days before the dark sleepers were used for the experiments. One-third of the culture water was replaced every day. Before excision of the gonads, genders were identified by morphological observation. Then the fish were placed in an ice bath for 3–5 min until they were lightly anesthetized. Testes and ovaries from 9 male and 9 female dark sleepers were collected for RNA extraction. Total RNA was extracted from all harvested organs using a high purity RNA fast extract reagent (BioTeke, Beijing, China). Samplings of testes (OT a, OT b, and OT c) and ovaries (OO a, OO b, and OO c) had three biological replicates, each made up of three different individual gonad tissues. The pieces of gonad from each fish were fixed in 4% PFA and sectioned at 5 μm for hematoxylin and eosin (HE) staining and observation by light microscopy to verify that the gonad sampled were in same development stage. Classification of gonad maturation state in this study refers to the Chinese black sleeper, *Bostrichthys sinensis* [26]. All experiments were performed according to the Guidelines for the Care and Use of Laboratory Animals in China. This study was also approved by the Ethics Committee of Experimental Animals at Nanjing Normal University. Total RNA was extracted using miRNeasy Kit (QIAGEN, USA) following the manufacturer’s protocol. The total RNA quantity and purity were analyzed with Bioanalyzer 2100 and RNA 6000 Nano LabChip Kit (Agilent, CA, USA) with RIN number > 7.0.

### Transcriptome sequencing and analysis

For six cDNA library constructions, approximately 5 μg of the total RNA per sample was used for the RNA sample preparations. The library for sequencing was generated using an IlluminaTruSeq RNA Sample Preparation Kit (Illumina, San Diego, CA, USA). Transcriptome sequencing was paired-end sequenced with the IlluminaHiSeq 2500platform, which generated approximately 125 bp paired-end (PE) raw reads by LC Sciences (Houston, TX, USA). The clean reads from the six transcriptomes were obtained from raw data by filtering out adaptor sequences and low-quality reads, then the remaining clean reads were assembled using Trinity software for de novo transcriptome assembly without a reference genome. The quality of the assembly was critically assessed by LC Sciences before subsequent analysis. All the non-redundant sequences were annotated against the Swiss-Prot database, non-redundant protein sequences (Nr), protein family (Pfam), KEGG Ortholog database (KEGG), eukaryotic Ortholog Groups (KOG), and GeneOntology (GO). The expression level of each transcript was measured as the number of clean reads mapped to its sequence. Gene expression level was measured by RPKM with RSEM 1.2.3. We determined the FDR threshold by using DESeq. FDR < 0.05 and fold change >2 were used to identify differentially expressed genes.

### Small RNA library sequencing and analysis

To identify the small RNAs involved in gonad development and sex differentiation, six small RNA libraries were generated from the male and female gonad samples using the IlluminaTruseq Small RNA Preparation kit (Illumina, San Diego, CA, USA) according to Illumina’s TruSeq Small RNA Sample Preparation Guide. Quantity and integrity were evaluated by Aligent 2100 Bioanalyzer, and then the libraries were sequenced by Illumina Hiseq2500 50SE (single end) at the LC-BIO (Hangzhou, China) following instructions for running the instrument. The raw sequences were processed using the Illumina pipeline program. After disregarding contaminated reads, including adapter dimers, junk, low complexity, common RNA families (rRNA, tRNA, snRNA, snoRNA), and repeats, the clean reads were filtered with the software package ACGT101-miR-v4.2 (LC Sciences, Houston, Texas, USA).

The clean sequence reads were mapped with miRBase 21.0, allowing a mismatch of one or two nucleotide bases, to identify known miRNAs and novel 3p- and 5p–derived miRNAs. The differentially expressed miRNAs (DEmiRNAs) between samples were identified by using a Student’s *t*-test based on the experimental design. In this test, the significance threshold was set at 0.05.

### miRNA target predictions

To predict the genes targeted by DE miRNAs, two computational target prediction algorithms (TargetScan 50 and miRanda 3.3a) were used to identify miRNA binding sites. Finally, the data predicted by TargetScan 50 and miRanda 3.3a were combined and the overlaps were calculated.

### Quantitative real-time PCR validation of miRNAs and mRNA

In total, 43 mRNAs and 16 miRNAs were verified by using quantitative real-time PCR (qRT-PCR). For mRNA, primers for qRT-PCR are listed in Additional file 1: Table S1, qRT-PCR method with β-actin as an internal control was used to explore mRNA expression levels. qRT-PCR was performed with an SYBR Green Master kit according to the manufacturer’s protocol (Roche, Basel, Switzerland). The experiments were carried out in triplicate with a total volume of 20 μL in an ABI StepOnePlus, containing 10 μL of SYBR Green Master, 4 μL of cDNA (500 ng), and 3 μL of forward and reverse primers (2 μmol/L). The qRT-PCR was programmed at 95 °C for 10 min, followed by 40 cycles of 95 °C for 15 s, and 55 °C for 1 min. The expression level was calculated by 2^-△△CT^ method and subjected to statistical analysis. For miRNA quantification, the specific RT primers and stem-loop primers are shown in Additional file 2: Table S2. U6 was used as an internal control. The selected miRNAs were analyzed by quantifying the miRNA stem-loop. Quantitative real-time PCR (qRT-PCR) was performed on ABI StepOnePlus system (Applied Biosystems, Foster, CA, USA) using qRT-PCR reagents provided by Toyobo. The thermal cycling program was set as follows: denaturation at 95 °C for 30 s (s), and then 40 cycles of amplification including denaturation at 95 °C for 5 s, annealing at 61 °C for 30 s, and extension at 72 °C for 30 s; after 40 cycles was final extension at 72 °C for 1 min.

### Detection and validation of SNPs

Potential SNPs were called using SAMtools software. SAMtools provides various utilities for manipulating alignments in the SAM format, including sorting, merging, indexing, and generating alignments in a per-position format. First, we called SNPs with the SAMtools mpileup utility. We then piped the BCF output file to SAMtools bcftools, which converted the BCF file into a VCF file. Afterwards, we piped the VCF file into vcfutils.pl with the varFilter-d100 option, which retained SNPs that had read depth higher than 100. In order to validate the accuracy of the SNPs predictions, forty SNPs were randomly selected for SNP validation. Fin tissue samples from 20 juvenile dark sleepers were collected for this SNP validation. Genomic DNA was extracted from pterygiophore tissue samples, using a cell/tissue genomic DNA extraction kit (centrifugal column type, Generay PBiotech, Shanghai). Primers were designed to amplify the flanking sequence of the selected SNPs using Primer Premier 5.0. The amplified PCR products were sequenced by utilizing an ABI3730 sequencer, and the products were analyzed using DNAMAN 8.0.

## Results

### Analysis of transcriptome sequencing of *O. potamophila* gonad tissue

Very few studies focusing on regulatory mechanisms of sex determination and gonadal differentiation in dark sleepers exist. Identifying the expression patterns of miRNAs and mRNAs in dark sleeper gonads is the first step in elucidating their gonadal development and differentiation. This experiment used mature gonads for the purpose of discovering gender specific genes and their putative regulated miRNAs in addition to finding sex-associated molecular markers.

In order to identify mRNA expression profiles in dark sleeper testes and ovaries, six cDNA libraries representing the testes (OT a, OT b, OT c) and ovaries (OO a, OO b, OO c) were constructed with total RNA and subjected to Illumina deep sequencing. In total, 52,751,768, 43,554,254, and 39,713,188 reads were obtained from the male libraries OT a, OT b, and OT c, respectively, and 51,538,466, 51,356,850, and 48,215,604 reads were obtained from the female libraries OO a, OO b, and OO c, respectively. After quality filtering, approximately 95.52%, 96.60%, and 95.27% reads from testis libraries and 97.52%, 97.64%, 97.64% reads from ovary libraries remained to be qualified for assembling. An overview of the sequencing and assembly results for these six libraries is shown in Additional file 3: Table S3. Through the Trinity de novo assembly method, we obtained 43,494 non-redundant genes, and 81,051 transcripts were achieved with an N50 of 2721 bp and an average length of 1599 bp (Additional file 4: Table S4). The length distribution of genes and transcripts larger than 200 bp is shown in Additional file 5: Figure S1.

All-unigene sequences were searched against Swiss-Prot, Nr, Pfam, KEGG, KOG, and GO databases, which returned 19,518 (44.88%), 24,291 (55.85%), 19,364 (44.52%), 13,895 (31.95%), 18,624 (42.82%), and 17,358 (39.91%) matches, respectively (Additional file 6: Table S5).

**Fig. 1:**
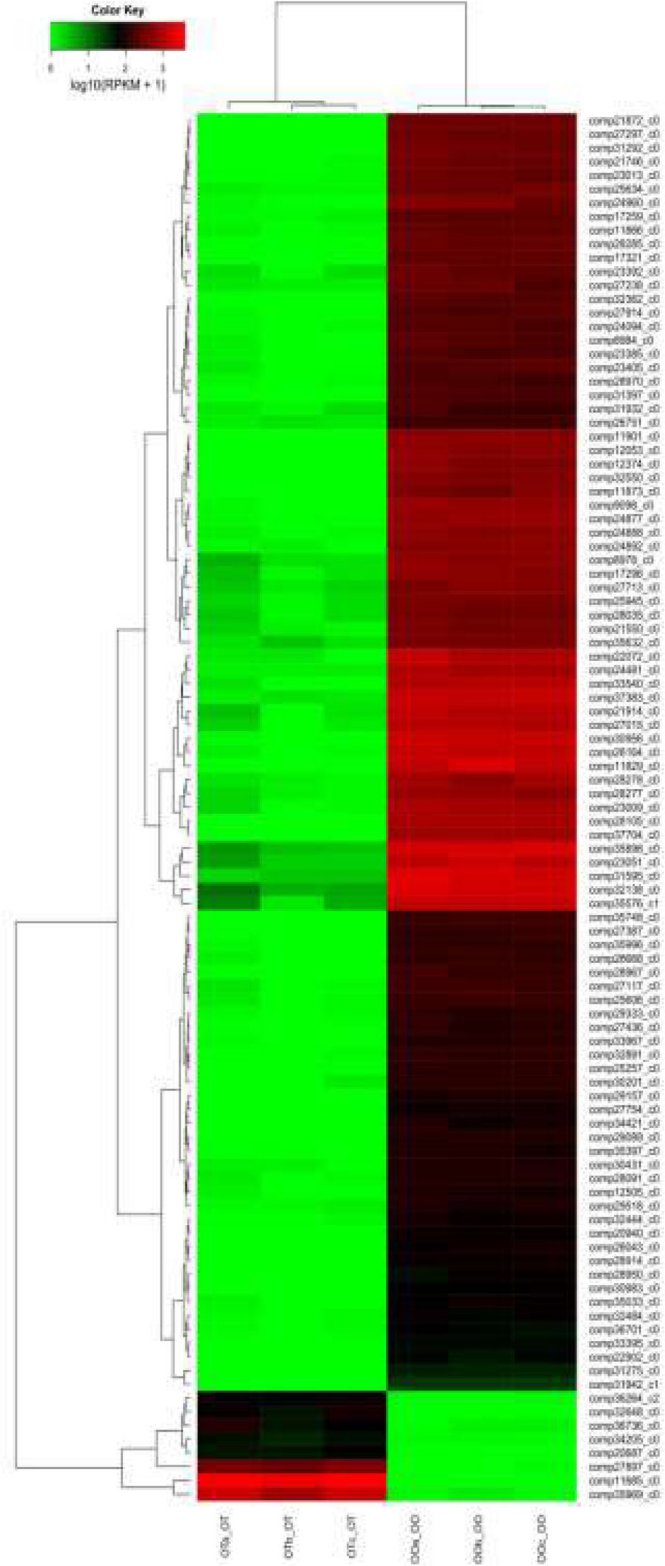
Heatmap of the differentially expressed genes of gonads. Heatmap of the count data of gonads for the differentially expressed genes between male and female individuals (Only the top 100 genes are included)

A total of 8103 male-biased and 8437 female-biased genes were identified in the gonad of the dark sleeper (Additional file 7: Figure S2), which showing significant expression difference between male and female. Clustering of the samples based on grouped males and females separately. A majority of the main 100 differentially expressed genes are female-biased genes, which were included in ZP1, ZP2 and ZP3. Among eight male-biased genes, half of them (FABP2, slc7a2, F10 and SUN1) were annotated (Fig. 1).

**Fig. 2:**
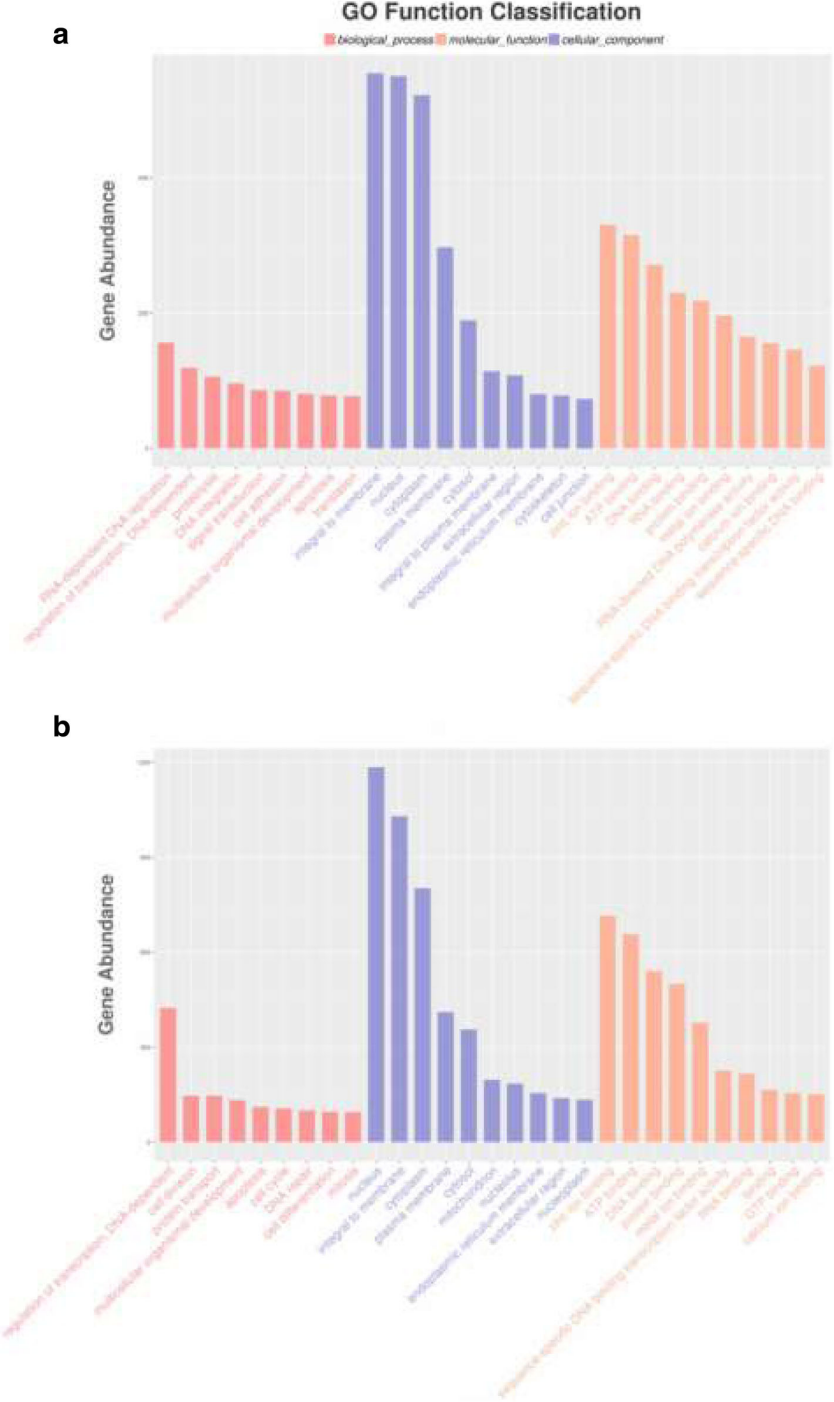
Functional annotation of male-biased genes (**a**) and female-biased genes (**b**) based on gene ontology (GO) categorization. GO analysis was performed for three main categories: biological process, molecular function, and cellular component

Gene ontology (GO) annotation was performed to classify sex-biased genes (Fig. 2). There were 3085 and 5429 DEGs from males and females, respectively, were assigned to a GO category. The most enriched GO-terms for male-biased DEGs included “RNA-dependent DNA replication” in the biological process category, “integral to membrane” in the molecular function category and “zinc ion binding” and “ATP binding” in the cellular component category. As for female-biased DEGs, the most enriched GO-terms at biological process level was “regulation of transcription, DNA-dependent”, the most enriched GO-terms associated with molecular function were “nucleus”, and the most enriched cellular component were “zinc ion binding” and “ATP binding”.

**Fig. 3:**
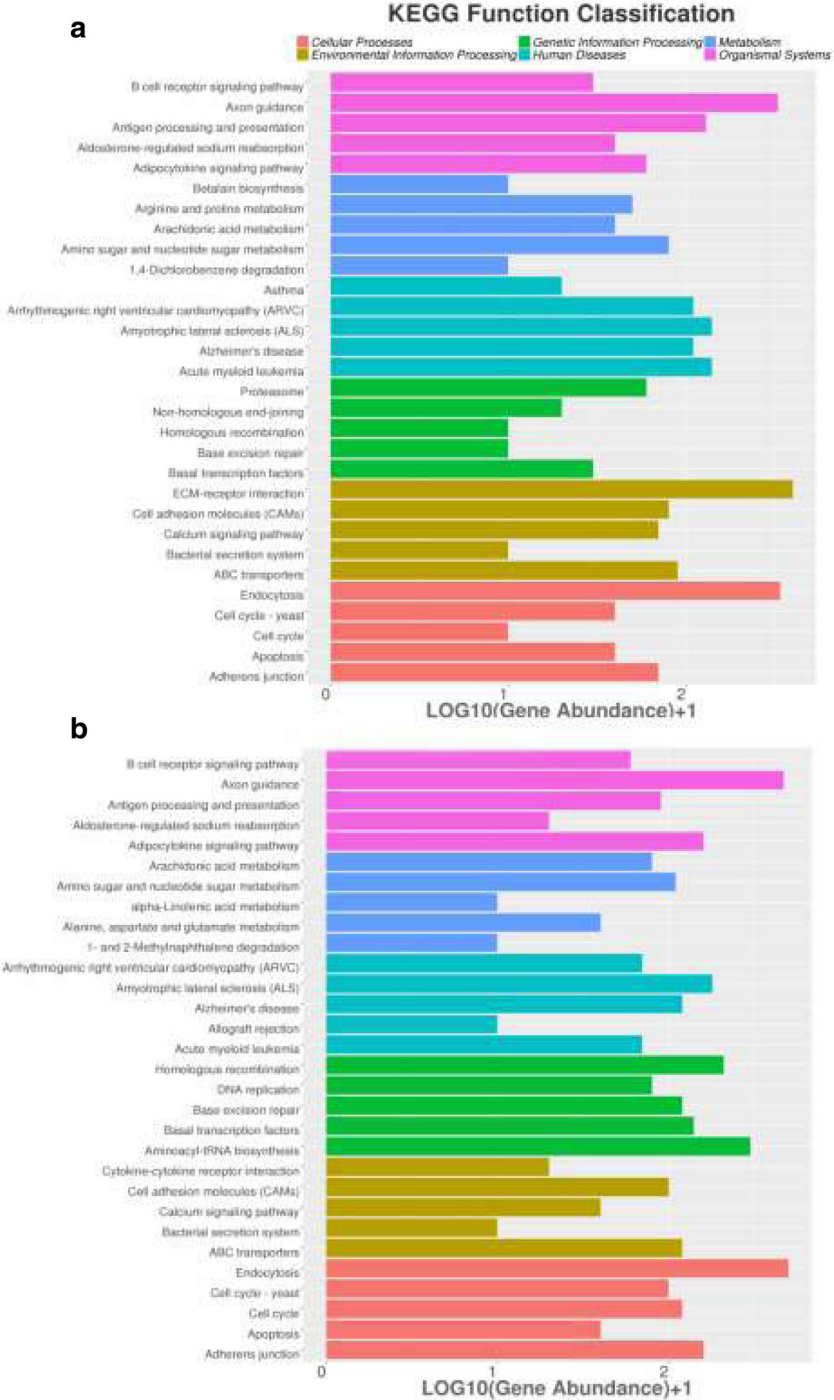
KEGG pathways enriched in the male-biased genes (**a**) and female-biased genes (**b**)

1789 male-biased DEGs and 2450 female-biased DEGs were mapped to KEGG pathways, respectively. Enriched pathways (Fig. 3) associated with male-biased DEGs were axon guidance, ECM-receptor interaction and endocytosis etc. And enriched pathways for female-biased DEGs were axon guidance, aminoacyl-tRNA biosynthesis, endocytosis, homologous recombination, adipocytokine signaling pathway, adherens junction etc.

### Analysis of miRNA sequencing of *O.potamophila* gonad tissue

In order to identify miRNA differentiation between male and female individuals, we relied on the six small RNA libraries representing the testes (OT a, OT b, OT c) and ovaries (OO a, OO b, OO c) constructed with total RNA and subjected to Illumina small RNA deep sequencing. After discarding junk sequences, 8.8, 8.1, 8.7, 4.0, 4.1, and 5.9 million clean reads were generated in OT a, OT b, OT c, OO a, OO b, and OO c samples, respectively (Additional file 8: Table S6). The length distributions of miRNAs were similar among libraries in that 21 nt RNAs were the most abundant (Additional file 9: Figure S3). In total, we identified 591 conserved miRNAs belonging to 164 miRNA families, and 214 predicted novel miRNAs in the six small RNA libraries (Additional file 10: Table S7 and Additional file 11: Table S8).

**Fig. 4:**
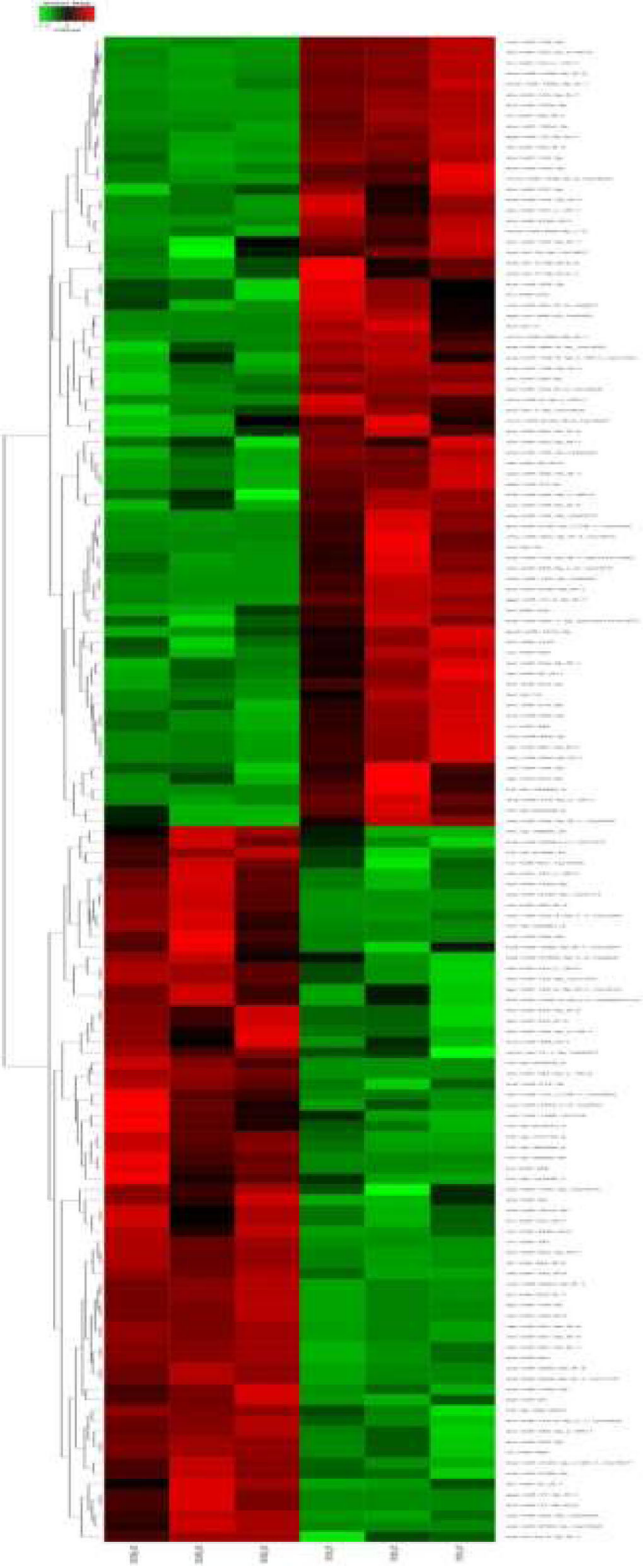
Heatmap of the differentially expressed miRNAs for gonads. Heatmap of the count data of gonads for the differentially expressed miRNAs between male and female individuals (*p*<0.05)

Compared with groups of OT and OO, 75 miRNAs were significantly upregulated in testes, while 68 miRNAs were significantly upregulated in ovaries. (*P* < 0.05, Additional file 12: Table S9) We found many of the same miRNAs that were reported in the previous study, such as miR-143, miR-145, and miR-138, and detected some novel miRNAs as well. In the two libraries of differentially expressed miRNAs, hierarchical clustering results allowed the six samples to be sorted into two distinct groups (Fig. 4).

### Correlation of DE miRNAs and DE mRNAs of *O. potamophila* gonad tissue

**Fig. 5:**
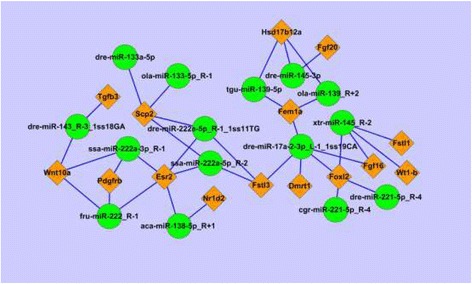
miRNA-mRNA negative correlation network. miRNAs and mRNAs were validated using real-time PCR

In order to determine miRNA-mRNA functional interactions, DE miRNAs and their predicted target lists were investigated for cognate mRNA targets using ACGT101-CORR 1.1. There were 53,374 miRNA-mRNA pairs in the gonad tissue of the dark sleeper. There were 27,583 negative miRNA-mRNA interactions with the involvement of 143 DE miRNAs and 16,540 DE mRNAs in total. Our study described the negative interaction between 15 genes with annotation and 15 miRNAs, which were verified by using qRT-PCR (Fig. 5).

### Confirmation of differential miRNAs and mRNAs by qRT-PCR

We used real-time qRT-PCR to investigate the relative expression levels of the 16 top miRNAs and 43 mRNAs related to sex determination and differentiation. The data obtained through RNA-seq and qRT-PCR was essentially identical (Table 1 and Table 2). Overall, the results of qRT-PCR validated those of RNA-seq and offered support for the reliability of the differentially expressed miRNAs and genes.

**Table 1:** Relative miRNA expression of top 16 DE miRNAs for comparison of the OT versus OO groups with respect to miRNA-seq and quantitative real-time PCR

miR_name	miR_seq	IlluminamiRNA-seq (log2 fold change)	Regulation (OT vs. OO)	Real-time PCR (log2 fold change)
dre-miR-221-5p_R-4	ACCTGGCATACAATGTAGATTT	−1.74	down	0.25
cgr-miR-221-5p_R-4	ACCTGGCATACAATGTAGATTT	−1.74	down	0.25
aca-miR-138-5p_R + 1	AGCTGGTGTTGTGAATCAGGCCG	2.29	up	2.91
cfa-miR-153_R + 2	TTGCATAGTCACAAAAGTGATC	−0.97	down	−0.36
dre-miR-145-3p	GGATTCCTGGAAATACTGTTCT	1.74	up	1.52
xtr-miR-145_R-2	GTCCAGTTTTCCCAGGAATCCC	2.91	up	2.47
fru-miR-222_R-1	AGCTACATCTGGCTACTGGGTCT	−1.53	down	−1.31
ssa-miR-222a-3p_R-1	AGCTACATCTGGCTACTGGGTCT	−1.53	down	−1.31
ola-miR-139_R + 2	TCTACAGTGCATGTGTCTCCAGT	−1.30	down	−0.87
tgu-miR-139-5p	TCTACAGTGCATGTGTCTCCAGT	−1.30	down	−0.87
dre-miR-143_R-3_1ss18GA	TGAGATGAAGCACTGTAA	2.85	up	2.50
dre-miR-17a-2-3p_L-1_1ss19CA	CTGCAGTGGAGGCACTTAAAGC	−1.07	down	−2.17
dre-miR-222a-5p_R-1_1ss11TG	TGCTCAGTAGGCAGTGTAGATC	−1.43	down	−1.26
ssa-miR-222a-5p_R-2	TGCTCAGTAGGCAGTGTAGATC	−1.43	down	−1.26
dre-miR-133a-5p	AGCTGGTAAAATGGAACCAAAT	4.90	up	5.15
ola-miR-133-5p_R-1	AGCTGGTAAAATGGAACCAAAT	4.90	up	5.15

**Table 2:** List of important genes related to sex determination/differentiation in the dark sleeper transcriptome

Accession	Gene name	Illumina mRNA-seq (log2fold change)	Regulation (OT vs. OO)	Real-time PCR (log2 fold change)
comp31991_c0	*Wt1-b*	1.94	up	2.89
comp30718_c0	*Dmrt1*	6.10	up	8.54
comp21149_c0	*Dmrt3a*	5.57	up	6.57
comp23897_c0	*Gata4*	1.20	up	3.38
comp27898_c0	*Gata2*	2.31	up	5.09
comp31039_c0	*Ar*	1.51	up	1.89
comp15491_c0	*Rspo1*	3.37	up	5.46
comp31921_c0	*Ctnnb1*	0.27	-	0.75
comp23019_c0	*Foxl2*	−7.28	down	−9.58
comp36367_c0	*Foxo3*	−1.10	down	−2.65
comp34722_c0	*Wnt10a*	3.02	up	5.62
comp3806_c0	*Cyp19a1*	−1.62	-	0.34
comp13638_c1	*Sox9*	0.97	-	1.84
comp19872_c1	*Sox3*	−6.92	down	−5.64
comp25339_c0	*Sox8*	−4.45	down	−6.97
comp35936_c2	*Esr2*	2.05	up	4.65
comp35195_c0	*Nr3c1*	−1.27	down	−3.47
comp27598_c0	*Nr1d2*	−3.96	down	−2.69
comp19368_c0	*Nr0b1*	2.00	up	4.21
comp24244_c0	*Fgf20*	−6.57	down	−5.14
comp18977_c0	*Fgf16*	−4.09	down	−3.94
comp24848_c0	*Fstl1*	2.07	up	2.46
comp32556_c0	*Fstl3*	3.73	up	1.58
comp4391_c1	*Fstl5*	inf	up	8.73
comp21914_c0	*Zp1*	−10.22	down	−8.36
comp29518_c0	*ZP2*	−8.26	down	−7.54
comp31397_c0	*ZP3*	-inf	down	−6.36
comp28002_c0	*Fem1a*	−1.37	down	−2.12
comp32386_c1	*Fem1c*	−2.93	down	−4.65
comp35903_c2	*Piwil1*	−2.02	down	−1.02
comp36970_c0	*Tdrd7b*	2.94	up	1.98
comp25576_c0	*Hsd17b1*	−7.66	down	−5.71
comp33961_c0	*Hsd16b3*	3.51	up	5.68
comp22996_c0	*Hsd17b7*	−1.60	down	−3.20
comp28954_c0	*Hsd17b12a*	−7.42	down	−4.38
comp16819_c0	*Pdgfrb*	1.68	up	3.59
comp26607_c1	*Pdgfb*	1.42	up	2.45
comp28781_c1	*Scp2*	1.58	up	3.67
comp25148_c0	*Dazl*	4.59	up	−1.25
comp32900_c0	*Dnali1*	6.47	up	4.32
comp23871_c0	*Tgfb1*	1.88	up	4.49
comp30516_c0	*Tgfb2*	7.66	up	3.25
comp24977_c0	*Tgfb3*	−3.75	down	−2.35

### SNP detection and validation

For extended application of the RNA-Seq data, structural variations were discovered using the assembled transcriptome. A total of 1130 and 1226 SNPs were identified in males and females, respectively. A/G was the most abundant type in both males and females (Table 3). Information on all SNPs detected in males and females is presented in Additional file 13: Table S10.

**Table 3:** SNP type in male and female dark sleeper

Type	A-T	A-G	A-C	C-T	C-G	C-A	G-T	G-C	G-A	T-G	T-C	T-A	Total
Sex													
Male	62	240	76	198	48	48	46	42	211	49	147	59	1226
Female	64	196	67	199	41	44	67	38	182	51	137	44	1130

Forty SNPs in twenty dark sleepers were subjected to experimental validation. Among the 40 primer pairs designed for SNP validation, 34 could amplify target sequences. Within these amplified sequences, 19 SNPs were validated, suggesting that approximately 50% of the predicted SNPs were indeed true SNPs. The primers of validated SNPs are listed in Additional file 14: Table S11.

## Discussion

As a commercial fish, the dark sleeper shows a sex dimorphism growth pattern, in that the male dark sleeper grows faster than the female. Artificial breeding and selection of dark sleepers have taken place in recent years. To further facilitate increased production of dark sleepers, the control of their sex is regarded as an important technique. Thus, understanding the detailed mechanisms involved in dark sleeper sex determination and development is necessary. This study presents mRNA and miRNA integrated analyses on the gonad tissue of the dark sleeper. A number of sex specific miRNA-mRNA pairs and SNPs were identified. Taking insight into miRNA-mRNA pairs and their corresponding SNPs can further the understanding of the regulatory mechanisms associated with sex determination and gonadal differentiation in the dark sleeper. This, then, constitutes a genetic breeding basis for this important commercial fish species.

In general, all of the RNA transcripts in the cell were included in the transcriptome data. The transcriptome reflects the mRNAs and miRNAs that are actively expressed under a particular condition. We attempted to acquire the miRNA-mRNA pairs through the use of DE miRNAs and DE mRNAs datasets and miRNA-targeting information. In the present study, a total of 53,374 miRNA-mRNA pairs were identified. In most cases, the negative correlation between miRNAs and their target mRNAs is often considered proof of miRNA targeting [27]. In our results, 27,583 miRNA-mRNA interaction sites with negative correlation were predicted with the involvement of 143 DE miRNAs and 16,540 DE mRNAs, which greatly exceeded our expectations. Moreover, this study validated 43 DE genes related to sex determination or differentiation and the top 16 DE miRNAs through the use of real-time PCR analysis.

In this study, compared with groups of OT and OO, 75 miRNAs were significantly upregulated in testes, while 68 miRNAs were significantly upregulated in ovaries. Increasing evidence suggests that miRNAs play an important role as regulators of reproduction [28, 29], and identifying the miRNA target genes is an important step for understanding their roles in gene regulatory networks.

Our results indicate that the miR-138, miR-145, miR-143, and miR-133 showed significant increased expression in testes compared with that in ovaries. miR-138 can regulate the *PI3K/AKT* signaling pathway by targeting *PDK1* [30]. The *PI3K-AKT* signaling pathway is involved in many fundamental functions including testis development and spermatogenesis [31]. In yellow catfish, there are more molecules of *PI3K-AKT* pathway expressed in YY than in XY testis [32]. Therefore, miR-138 might be involved in testis development and spermatogenesis. miR-143 and miR-145 were proved to be co-transcribed in multipotent murine cardiac progenitors [33]. Moreover, miR-145 has been shown to play an important role in male and female differentiation, and *Sox9*, a male factor acting on Sertoli cells, has been tested as a direct target of miR-145 by luciferase reporter assay [34]. However, *Sox9* was not differentially expressed in the gonad of the dark sleeper, which may be a result of the samples we collected were in relatively late stage. The results of our histologic section indicated that the testes were in IV stage, leading to the production of mature spermatids (Additional file 15: Figure S4). Previous studies have suggested that *Sox9* overexpressed in the immature gonads of Siberian sturgeons and sablefish [35, 36]. Hence, *Sox9* might play a role in an earlier stage of gonadal development in the dark sleeper. In addition, miR-143 is critical for the formation of primordial follicles and regulates ovarian development [37], and it is also a dominant miRNA detected in both ovaries and testes of Atlantic halibut [38]and Nile tilapia [20]. The predicted genes of miR-143 and miR-145 in our study include *Foxl2*, *Fgf16*, *Fgf20*, and *Fem-1a*. *Foxl2* encodes a forkhead transcription factor and is involved in ovary differentiation [39], and showed that it is predominantly expressed in female gonads. However, another ovarian marker, *Cyp19a1*, showed that it is expressed similarly in both male and female gonads. A possible reason for this is that *Cyp19a1* has been proved essential for early sex differentiation and ovarian development, and it is regarded as an early marker of ovarian differentiation [40], but *Foxl2* is crucial for maintenance and functioning of adult ovaries [41]. The histologic section verified that the ovaries were in IV stage, during which mature oocytes can be produced (Additional file 14: Figure S4). *Fgfs* (Fibroblast growth factors) have been shown to participate in a wide variety of biological processes, including cell proliferation, migration, growth, differentiation, sex determination, and sex differentiation [42, 43]. For instance, in zebrafish, *Fgf16* is required for pectoral fin bud formation [44] and *Fgf20* is necessary for triggering regeneration of its fins [45]. In the XX ovaries and phase II oocytes of Nile tilapia, *Fgf16* and *Fgf20* showed higher expression, which suggests that *Fgf16* and *Fgf20* are involved in early oocyte development [46]. Moreover, the gene *Fem-1* has been shown to play a role in the sex determination signaling pathway in *Caenorhabditis elegans* [47]. There are three conservative members of the *Fem-1* family in vertebrates—namely *Fem-1a*, *Fem-1b*, and *Fem-1c*. *Fem-1a* and *Fem-1c* were identified as female-biased genes in our study. The production of connective tissue growth factor can be limited by miR-133 [48]. miR-133 can regulate the oocyte meiosis by down regulating the expression of cyclin B gene by using double-luciferase reporter genes assay [49].

In contrast with the testes, the most abundant expressed miRNAs in the ovaries of the dark sleeper were miR-221, miR-153, miR-222, and miR-139. Both miR-221 and miR-222 can maintain the undifferentiated state of mammalian spermatogonia [50]. *ESR2* and *Gata2* are among the predicted targets of miR-221 and miR-222. The *ESR2* had higher expression in the testes. The expression of estrogen receptors has been shown male-biased in bluehead wrasses [51], Nile tilapia (70dah) [52], and rainbow trout (60-110dpf) [53]. During mouse gonad development, *Gata2* was shown to be sex-biased. The expression of *Gata2* was found to be much greater in the fetal ovaries from 11.5 days postcoitum (dpc) onwards. However, it was not detected in the fetal testes during the period studied(10.5–15.5dpc) [54]. The role of *Gata2* in teleosts needs to be further studied. The role of miR-139 in fishes has yet to be studied, but an increasing number of studies have suggested that miR-139 suppresses the *Wnt* signaling activity by targeting several key genes such as *TCF-4* and *Wnt1* in this pathway [55, 56]. *Wnt* signaling has been known to be involved in mammalian ovary development [57], and in humans, the overexpression of *Wnt4* is associated with sex reversal [58]. *Rspo* plays an essential role in the *Wnt* signaling pathway in medaka [59] and activated *Rspo1* are sufficient to induce ovarian differentiation in XY medaka [60]. In our study, *Rspo* was shown to have male-biased expression, similar to the express pattern found in bluehead wrasses [51]. *Ctnnb1* **(** *β-catenin*) is a central molecule in the *Wnt* signaling pathway [61]. *Ctnnb1* was highly expressed in both the ovaries and testes of the dark sleeper, but was not shown to be sex-biased. *Fst* is another important gene down streamed to the *Wnt* signaling pathway [62]. We detected some *Fst* genes in our study such as *Fstl1*, *Fstl3* and *Fstl5*, all of which showed male-biased expression. Our results seem to verify the opinion that the female-specific function of the *Wnt* pathway is not a general pattern in fishes [63]. Clearly, the function of the *Wnt* pathway in teleost fishes needs to be further studied. miR-17-5p plays an important role in ovarian function in mice [64]. In zebrafish, miR-17a may be involved in the follicle development and maturation of oocytes [65]. *Dmrt1* is one of the predicted target gene of miR-17a. *Dmrt* genes have been reported to have a conserved function related to testis development in vertebrates [1, 66]. We identified *Dmrt1* and *Dmrt3a* as male-biased genes in this study. Our results suggest that *Dmrt1* and *Dmrt3a* may both be important factors for testicular differentiation. It is worth noting that in our study, *Dmrt1* and *Foxl2* were both predicted to be the potential target genes of miR-17a. miR-17a in teleost fishes also warrants further study. miR-153 has been well studied in the context of human cancers, and it has been shown to be markedly down regulated in the cells that undergo EMT(epithelial-mesenchymal transition) [67]. Niu et al. [68] revealed that *TGF-β2* was negatively regulated by miR-153. The signals that come from the *TGF-β* superfamily of proteins, such as *TGF-β1*, *TGF-β2*, and *TGF-β3*, play a critical role in testis development and spermatogenesis [69]. Hence, miR-153 may have a role in sex determination and differentiation of fish species.

Several other genes related to sex determination and differentiation were also identified in this study. *Dnali1* is a type of axonemal dynein, a large motor protein complex which enables the movement of eukaryotic cilia and flagella. Research has shown that male infertility or lateralization defects can occur due to a disruption of axonemal dynein function [70]. *Dnali1* was found to be a male-biased gene in the olive flounder as well [71]. *Tdrd7* has been shown to function during spermatogenesis [72] and is regarded as the marker of the testis. In this study, we found *Tdrd7* to be a male-biased gene showing a similar expression pattern with the Asian seabass [73]. *Piwi* proteins have been shown to act as functional partners with *Tdrds*, working together to regulate spermiogenesis [74]. *Piwil* was reported to be a male-biased gene in the yellow catfish [75], in contrast to the findings of our study. There is evidence that *Piwi* might be involved in mammalian oocytes and early embryos [76]. However, further investigation is needed to gain a better understanding of the functions of *Piwil*. Finally, *Zp* genes, found on the extracellular matrix of oocytes, function as receptors for the binding of sperm in mammals [77, 78]. In our study, Zona pellucida proteins *Zp1*, *Zp2*, and *Zp3* were identified as female-biased genes.

## Conclusion

The present study describes the first integrated analysis of mRNA and miRNA found in gonad tissue of dark sleeper. There were 27,583 negative miRNA-mRNA interactions with the involvement of 143 DE miRNAs and 16,540 DE mRNAs in total. A significant number of common mRNAs and miRNAs involved in sex determination and differentiation were obtained by comparing the mRNA and miRNA expression profiles in the gonad tissue of the dark sleeper. It is a good case for analyzing mRNA and miRNA co-expression in order to uncover the sex differences between males and females of non-model fish species by using NGST.

## Additional files

Additional file 1: Table S1. RT-qPCR primers for mRNAs. (DOCX 18 kb)

Additional file 2: Table S2. RT-qPCR primers for miRNAs. (DOCX 13 kb)

Additional file 3: Table S3. Summary of sequence data generated for dark sleeper transcriptome, and quality filtering. (DOCX 13 kb)

Additional file 4: Table S4. Assembly statistics of reads. (DOCX 12 kb)

Additional file 5: Figure S1. Distribution of assembled genes and transcript length. (DOCX 170 kb)

Additional file 6: Table S5. Blast analysis of non-redundant unigenes against public databases. (DOCX 12 kb)

Additional file 7: Figure S2. Differentially expressed genes in the two sexes for gonads of dark sleeper. (DOCX 126 kb)

Additional file 8: Table S6. Overview of reads for sRNA-seq from raw data to high quality reads, and quality filtering. (DOCX 19 kb)

Additional file 9: Figure S3. Length distribution of small RNA in six miRNA libraries. (DOCX 109 kb)

Additional file 10: Table S7. List of miRNA member in each family in dark sleeper. (DOCX 23 kb)

Additional file 11: Table S8. List of know-miRNA and novel-miRNA of dark sleeper. (DOCX 57 kb)

Additional file 12: Table S9. List of differentially expressed miRNAs of dark sleeper in testis and ovary. (DOCX 34 kb)

Additional file 13: Table S10. All SNP detected in gonad tissue of dark sleeper. (DOCX 262 kb)

Additional file 14: Table S11. Information for validated SNPs. (DOCX 17 kb)

Additional file 15: Figure S4. Histological section analysis of testis and ovary structure in dark sleeper with hematoxylin and eosin staining. (DOCX 1183 kb)

## Abbreviations

*Cyp19a*: Cytochrome P450, family 19, subfamily A
*Dmrt1*: Doublesex and mab-3-related transcription factor 1
*Dmrt3a*: Doublesex and mab-3-related transcription factor 3a
*Dnali1*: Dynein axonemal light intermediate chain 1
*ESR2*: Estrogen receptor 2
*Fem-1*: Feminization-1
*Fgf16*: Fibroblast growth factor 16
*Fgf20*: Fibroblast growth factor 20
*Foxl2*: Forkhead box protein L2
*Fst*: Follistatin-like protein
RPKM: Reads per kilobase per million
*Rspo*: R-spondin
*Tdrd7*: Tudor domain-containing protein 7
TGF-β: Transforming growth factor beta
*Zp*: Zona pellucida proteins
