# Supplementary material for: Integrated analysis of mRNA-seq and miRNA-seq reveals the potential roles of sex-biased miRNA-mRNA pairs in gonad tissue of dark sleeper (Odontobutis potamophila)
Source: BMC Genomics. 2017 Aug 14;18:613. doi: 10.1186/s12864-017-3995-9 (PMC5557427; doi:10.1186/s12864-017-3995-9)
Supplement: Supplementary file 1 — RT-qPCR primers for mRNAs. (DOCX 18 kb) [file 12864_2017_3995_MOESM1_ESM.docx]

**Table S1** RT-qPCR primers for mRNAs

| **Genes name** | **Forward primer (5′-3′)** | **Reverse primer (5′-3′)** |
| --- | --- | --- |
| *Wt1-b* | CCCTCCCAAAACACTCTG | TACTCACAAACCCGCTCTT |
| *Dmrt1* | ACAAAACAACAACCACACATT | GACATTAGAAAGCCCAACAC |
| *Dmrt3a* | TCTTCCCAGCACACAAAC | CACCTCAATGGCACCTAC |
| *Gata4* | TATCTGTGTAATGCCTGTGG | AGTGTAGTGGTGGTAGTGTGG |
| *Gata2* | CCGAATGAAGGGTGAATAGGC | CAACCGAAAGATGTCGAGCAA |
| *Ar* | CTGTGTCCGTGTCTCTGG | GTCCTCTGTTTGGTTGATGT |
| *Rspo1* | TAAGCGAAACCCTCACAC | AAACCGTCCTCCACAAAG |
| *Ctnnb1* | CTGATTTGATGGAGTTGGAC | AAGGTGCTGTGGTGGTAA |
| *Foxl2* | CTTCCTTGTTCATCTTGTGG | TTTGGCTGTGTTGGTGTC |
| *Foxo3* | TTTACTCCACTCCCAGCA | TTATCACCAAGCCCATCA |
| *Wnt10a* | TATGTAAAGGAAAGAAGGAAGG | AGATGAAGCAACGGAGTG |
| *Cyp19a1* | GCCTTCATCATTACCATAGCG | GGCGAACTTTGACAAAACTGT |
| *Sox9* | AGCAGACAACCACCTCATT | AGTCCAGTCGTAGCCCTT |
| *Sox3* | CGTGAAGAGGCTGTGTATTT | GGGACAACAAAAGAGGAGA |
| *Sox8* | CCCTCACAGTCCTTCCTC | CCAAGCCTATCAGTCAAATC |
| *Esr2* | CCTCCTCAACTCCAACCT | ATCACAGCGTCCAACAAA |
| *Nr3c1* | GGACGGATGACCTAACTG | CTGACTCTCTGCCCACTC |
| *Nr1d2* | GACAACCAGCCAACATTC | AGCACTCAACCCCAGATAC |
| *Nr0b1* | AACTCCTCATTGCCCTGT | CGTGAATCTCCTACTTTCCA |
| *Fgf20* | AAAACAAGTGGATAGAGCGT | TAAAGACAGAGCGGCAGA |
| *Fgf16* | GGAACAAAACCACTAAAATGA | TCGCACACAACACCTACA |
| *Fstl1* | CTCAACAAGCATCAGGAAA | CCCAAAGTCGGTCAAGTC |
| *Fstl3* | GCTCTAAGGTGGTGTGTCC | CTGGGGTAAGTGATGTTGTC |
| *Fstl5* | GGACCTGCTATTTGCTGA | GAGAGATTCGGTTGATGGT |
| *Zp1* | TAATCAATCACACGCCAA | GCAACAGCAAACAGACACT |
| *ZP2* | TCAATAGTCCAGCCATCC | CATCCGTCATAAAGCAGAGT |
| *ZP3* | TCATACTTACCCACACCATACT | TCTCACTACCTCCAAACCC |
| *Fem1a* | TTGGTTTATGCTGCGTTT | CTTTGCTTGGTGATGTGTC |
| *Fem1c* | CTGCCTTCTCCTTGATACTG | ATGCTGTGCCGTTGTTAG |
| *Piwil1* | GTTTGCGTCATTTGCTTT | CCAGTTTTAGTCGGGTTGT |
| *Tdrd7b* | TGTCCGTCACCCTAAACT | GCTACCACCAATAAACAGGA |
| *Hsd17b1* | TGCGTTCAGATACTTCACC | GGATGCTGTTCTTCGTCTT |
| *Hsd16b3* | CGGGGATAAAACATACGG | AGAAGAGAAGCGAGTGAGG |
| *Hsd17b7* | TATCCTGCCCTCCTTCTT | TTATTCCAAAGCCTGATGTT |
| *Hsd17b12a* | GCATTCTACTCGTTTTATTTTG | TTTTACATTTTCACTTTCACTCC |
| *Pdgfrb* | TACATCATTCCCATCCCA | TCTCACTGCTCTCCTCCA |
| *Pdgfb* | GACCCCAAACAGCAATAA | TCACTACCTTCTCCCAACC |
| *Scp2* | GAGGCTGTCTGGTTTGTG | GTCTGTGGGTTCATTTTCC |
| *Dazl* | CCCACACTTCATCTTCCC | TCCCGTATTTCATTTTCATC |
| *Dnali1* | AAGGCACAATAGCATAACATAA | AGACCGAAAGAACCCAAC |
| *Tgfb1* | AGAGATTTAGGATGGAAGTGG | CTGGGTTGTGGTGTTTGT |
| *Tgfb2* | AGAAGCAGTGAGTGAGTGG | TGGGAATGATGTAGTTGTTG |
| *Tgfb3* | GTTTATTTTGTCCCTTCATCC | CAGTTGGTTTGCTTCGTC |
| *β-actin* | CTCTTCCAGCCATCCTTCCT | TCAGGTGGGGCAATGATCTT |
