# Supplementary material for: Integrated analysis of mRNA-seq and miRNA-seq reveals the potential roles of sex-biased miRNA-mRNA pairs in gonad tissue of dark sleeper (Odontobutis potamophila)
Source: BMC Genomics. 2017 Aug 14;18:613. doi: 10.1186/s12864-017-3995-9 (PMC5557427; doi:10.1186/s12864-017-3995-9)
Supplement: Supplementary file 2 — RT-qPCR primers for miRNAs. (DOCX 13 kb) [file 12864_2017_3995_MOESM2_ESM.docx]

**Table S2** RT-qPCR primers for miRNAs

| **Name** | **Primer (5′-3′)** |
| --- | --- |
|  |  |
| dre-miR-221-5p RT | CTCAACTGGTGTCGTGGAGTCGGCAATTCAGTTGAGAAATCTAC |
| dre-miR-221-5p F | ACACTCCAGCTGGGACCTGGCATACAATGTAG |
| miR-221-5p | TGTCGTGGAGTCGGCAATTC |
| aca-miR-138-5p RT | CTCAACTGGTGTCGTGGAGTCGGCAATTCAGTTGAGCGGCCTGA |
| aca-miR-138-5p F | ACACTCCAGCTGGGAGCTGGTGTTGTGAATCAG |
| miR-138-5p | TGTCGTGGAGTCGGCAATTC |
| cfa-miR-153 RT | CTCAACTGGTGTCGTGGAGTCGGCAATTCAGTTGAGGATCACTT |
| cfa-miR-153 F | ACACTCCAGCTGGGTTGCATAGTCACAAAAGT |
| miR-153 | TGTCGTGGAGTCGGCAATTC |
| dre-miR-145-3p RT | CTCAACTGGTGTCGTGGAGTCGGCAATTCAGTTGAGAGAACAGT |
| dre-miR-145-3p F | ACACTCCAGCTGGGGGATTCCTGGAAATACTG |
| miR-145-3p | TGTCGTGGAGTCGGCAATTC |
| xtr-miR-145 RT | CTCAACTGGTGTCGTGGAGTCGGCAATTCAGTTGAGGGGATTCC |
| xtr-miR-145 F | ACACTCCAGCTGGGGTCCAGTTTTCCCAGGAA |
| miR-145 | TGTCGTGGAGTCGGCAATTC |
| fru-miR-222 RT | CTCAACTGGTGTCGTGGAGTCGGCAATTCAGTTGAGAGACCCAG |
| fru-miR-222 F | ACACTCCAGCTGGGAGCTACATCTGGCTACTGG |
| miR-222 | TGTCGTGGAGTCGGCAATTC |
| ola-miR-139 RT | CTCAACTGGTGTCGTGGAGTCGGCAATTCAGTTGAGACTGGAGA |
| ola-miR-139 F | ACACTCCAGCTGGGTCTACAGTGCATGTGTCTC |
| miR-139 | TGTCGTGGAGTCGGCAATTC |
| dre-miR-143 RT | CTCAACTGGTGTCGTGGAGTCGGCAATTCAGTTGAGTTACAGTG |
| dre-miR-143 F | ACACTCCAGCTGGGTGAGATGAAGCACT |
| miR-143 | TGTCGTGGAGTCGGCAATTC |
| dre-miR-17a-2-3p RT | CTCAACTGGTGTCGTGGAGTCGGCAATTCAGTTGAGGCTTTAAG |
| dre-miR-17a-2-3p F | ACACTCCAGCTGGGCTGCAGTGGAGGCACTTA |
| miR-17a | TGTCGTGGAGTCGGCAATTC |
| dre-miR-222a-5p RT | CTCAACTGGTGTCGTGGAGTCGGCAATTCAGTTGAGGATCTACA |
| dre-miR-222a-5p F | ACACTCCAGCTGGGTGCTCAGTAGGCAGTGTA |
| miR-222a | TGTCGTGGAGTCGGCAATTC |
| dre-miR-133a-5p RT | CTCAACTGGTGTCGTGGAGTCGGCAATTCAGTTGAGATTTGGTT |
| dre-miR-133a-5p F | ACACTCCAGCTGGGAGCTGGTAAAATGGAACC |
| miR-133a | TGTCGTGGAGTCGGCAATTC |
| u6-RT | AACGCTTCACGAATTTGCGT |
| u6-F | CTCGCTTCGGCAGCACA |
| u6-R | AACGCTTCACGAATTTGCGT |
