# Supplementary material for: Integrated analysis of mRNA-seq and miRNA-seq reveals the potential roles of sex-biased miRNA-mRNA pairs in gonad tissue of dark sleeper (Odontobutis potamophila)
Source: BMC Genomics. 2017 Aug 14;18:613. doi: 10.1186/s12864-017-3995-9 (PMC5557427; doi:10.1186/s12864-017-3995-9)
Supplement: Supplementary file 3 — Summary of sequence data generated for dark sleeper transcriptome, and quality filtering. (DOCX 13 kb) [file 12864_2017_3995_MOESM3_ESM.docx]

**Table S3** Summary of sequence data generated for dark sleeper transcriptome, and quality filtering

| Sample | Raw Data | Valid Data | Valid% | Q20% | Q30% | GC% |
| --- | --- | --- | --- | --- | --- | --- |
| OT a | 55228574 | 52751768 | 95.52 | 93.16 | 86.88 | 47.47 |
| OT b | 45088670 | 43554254 | 96.6 | 93.58 | 87.49 | 47.9 |
| OT c | 41685382 | 39713188 | 95.27 | 93.21 | 87.11 | 47.56 |
| OO a | 52848930 | 51538466 | 97.52 | 92.45 | 86.56 | 48.33 |
| OO b | 52597122 | 51356850 | 97.64 | 93.11 | 87.4 | 48.18 |
| OO c | 49381582 | 48215604 | 97.64 | 93.14 | 87.43 | 48.26 |
