# Supplementary material for: Integrated analysis of mRNA-seq and miRNA-seq reveals the potential roles of sex-biased miRNA-mRNA pairs in gonad tissue of dark sleeper (Odontobutis potamophila)
Source: BMC Genomics. 2017 Aug 14;18:613. doi: 10.1186/s12864-017-3995-9 (PMC5557427; doi:10.1186/s12864-017-3995-9)
Supplement: Supplementary file 4 — Assembly statistics of reads. (DOCX 12 kb) [file 12864_2017_3995_MOESM4_ESM.docx]

**Table S4** Assembly statistics of reads

|  | All | Min Length | Mean Length | Max Length | Total Assembled bases | N50 |
| --- | --- | --- | --- | --- | --- | --- |
| gene | 43494 | 201 | 1235 | 17804 | 53727334 | 2274 |
| transcript | 81051 | 201 | 1599 | 17804 | 129656707 | 2721 |
