# Supplementary figures and images for: Integrated analysis of mRNA-seq and miRNA-seq reveals the potential roles of sex-biased miRNA-mRNA pairs in gonad tissue of dark sleeper (Odontobutis potamophila)
Source: BMC Genomics. 2017 Aug 14;18:613. doi: 10.1186/s12864-017-3995-9 (PMC5557427; doi:10.1186/s12864-017-3995-9)

**Fig. S1** Distribution of the assembled genes and transcript length.


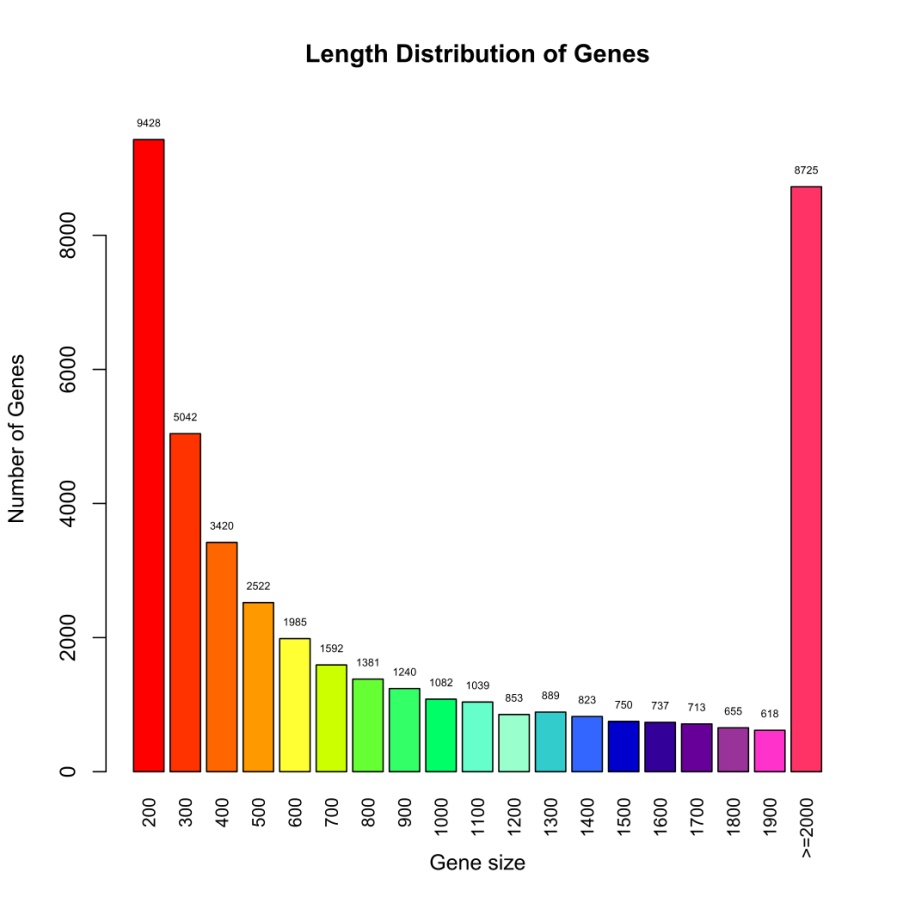

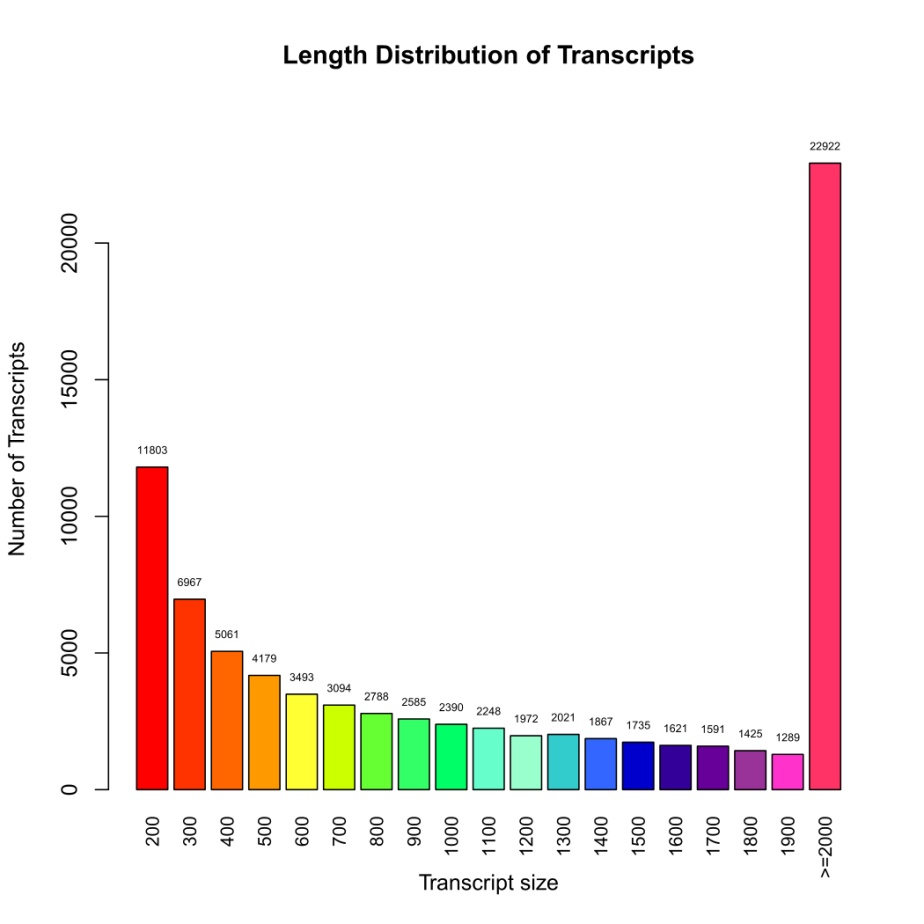

Supplement: Supplementary file 5 — Distribution of assembled genes and transcript length. (DOCX 170 kb) [file 12864_2017_3995_MOESM5_ESM.docx]

**Fig. S3** The length distribution of the small RNA in six miRNA libraries.


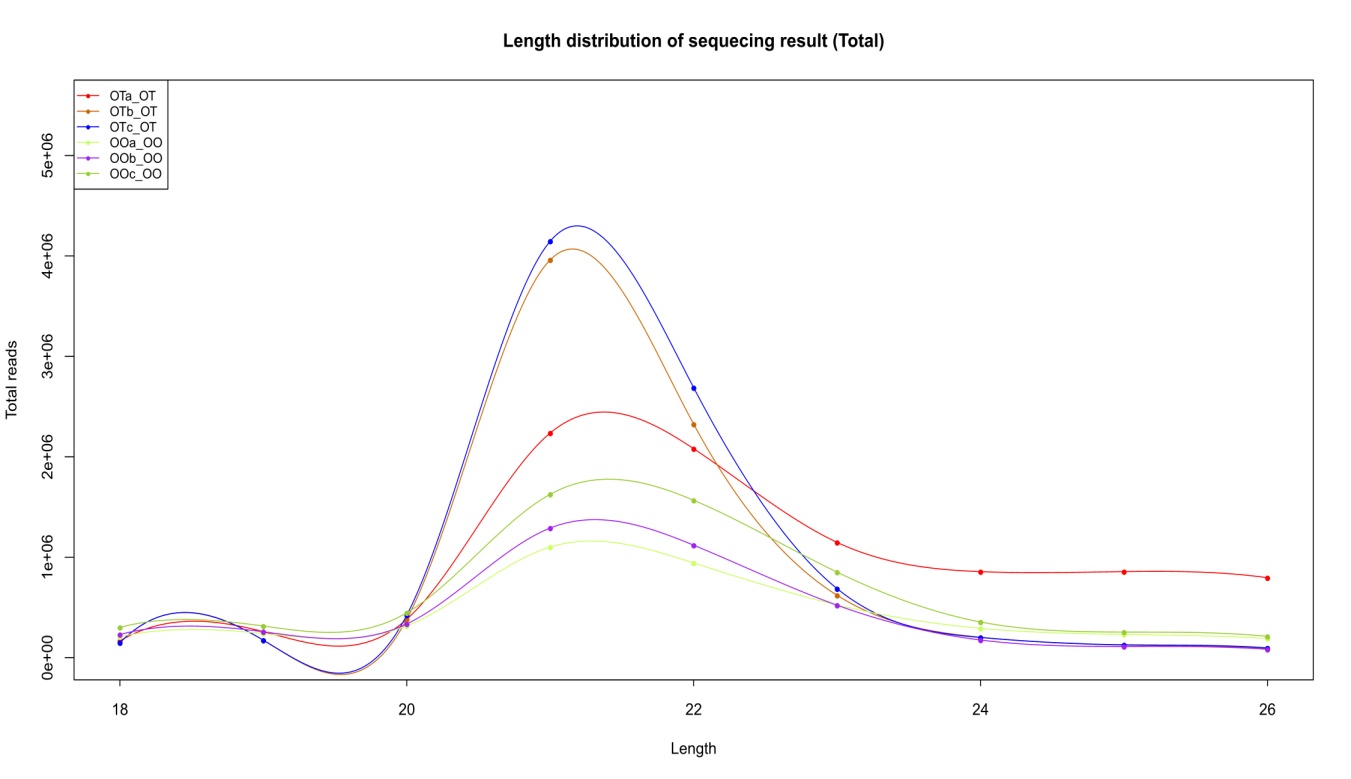

Supplement: Supplementary file 9 — Length distribution of small RNA in six miRNA libraries. (DOCX 109 kb) [file 12864_2017_3995_MOESM9_ESM.docx]
