# Supplementary material for: Integrated analysis of mRNA-seq and miRNA-seq reveals the potential roles of sex-biased miRNA-mRNA pairs in gonad tissue of dark sleeper (Odontobutis potamophila)
Source: BMC Genomics. 2017 Aug 14;18:613. doi: 10.1186/s12864-017-3995-9 (PMC5557427; doi:10.1186/s12864-017-3995-9)
Supplement: Supplementary file 6 — Blast analysis of non-redundant unigenes against public databases. (DOCX 12 kb) [file 12864_2017_3995_MOESM6_ESM.docx]

**Table S5** Blast analysis of non-redundant unigenes against public databases

| gene_number | swiss-prot | nr | Pfam | KEGG | KOG | GO |
| --- | --- | --- | --- | --- | --- | --- |
| 43494 | 19518 | 24291 | 19364 | 13859 | 18624 | 17358 |
| 100% | 44.88% | 55.58% | 44.52% | 31.95% | 42.82% | 39.91% |
