# Supplementary material for: Integrated analysis of mRNA-seq and miRNA-seq reveals the potential roles of sex-biased miRNA-mRNA pairs in gonad tissue of dark sleeper (Odontobutis potamophila)
Source: BMC Genomics. 2017 Aug 14;18:613. doi: 10.1186/s12864-017-3995-9 (PMC5557427; doi:10.1186/s12864-017-3995-9)

**Fig. S2** Differentially expressed genes in the two sexes for gonad in dark sleeper. Red represents up-regulated in testis, green represents up-regulated in ovary.


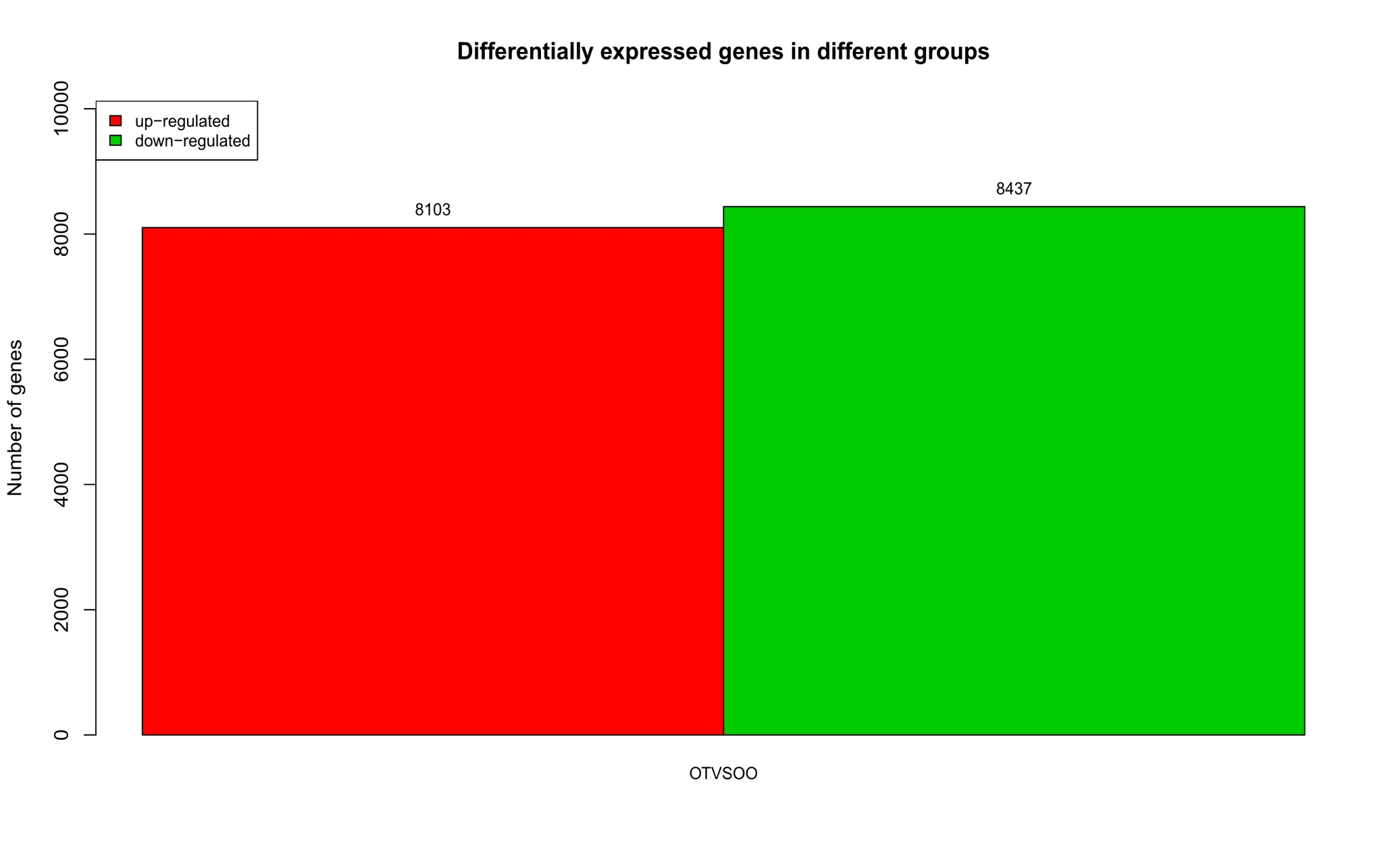

Supplement: Supplementary file 7 — Differentially expressed genes in the two sexes for gonads of dark sleeper. (DOCX 126 kb) [file 12864_2017_3995_MOESM7_ESM.docx]
