# Supplementary material for: Integrated analysis of mRNA-seq and miRNA-seq reveals the potential roles of sex-biased miRNA-mRNA pairs in gonad tissue of dark sleeper (Odontobutis potamophila)
Source: BMC Genomics. 2017 Aug 14;18:613. doi: 10.1186/s12864-017-3995-9 (PMC5557427; doi:10.1186/s12864-017-3995-9)
Supplement: Supplementary file 8 — Overview of reads for sRNA-seq from raw data to high quality reads, and quality filtering. (DOCX 19 kb) [file 12864_2017_3995_MOESM8_ESM.docx]

**Table S6** Overview of reads for sRNA-seq from raw data to high quality reads, and quality filtering

|  |  | **OT_a** |  |  |  | **OT_b** |  |  |  | **OT_c** |  |  |  |
| --- | --- | --- | --- | --- | --- | --- | --- | --- | --- | --- | --- | --- | --- |
| **lib** | type | Total | % of Total | uniq | % of uniq | Total | % of Total | uniq | % of uniq | Total | % of Total | uniq | % of uniq |
| **Raw reads** | NA | 15,346,363 | 100.00 | 3,821,690 | 100 | 16,855,166 | 100.00 | 1,195,364 | 100.00 | 15,802,160 | 100.00 | 1,168,549 | 100.00 |
| **3ADT&length filter** | Sequence type | 6,190,833 | 40.34 | 1,525,026 | 39.9 | 8,550,721 | 50.73 | 700,655 | 58.61 | 6,861,156 | 43.42 | 617,636 | 52.85 |
| **Junk reads** | Sequence type | 45,466 | 0.30 | 27,800 | 0.73 | 16,666 | 0.10 | 7,110 | 0.59 | 29,809 | 0.19 | 14,162 | 1.21 |
| **Rfam** | RNA class | 312,065 | 2.03 | 38,287 | 1 | 241,605 | 1.43 | 30,347 | 2.54 | 205,203 | 1.30 | 30,504 | 2.61 |
| **Repeats** | RNA class | 30,694 | 0.20 | 4,554 | 0.12 | 26,550 | 0.16 | 2,949 | 0.25 | 29,785 | 0.19 | 3,654 | 0.31 |
| **valid reads** | Sequence type | 8,784,094 | 57.24 | 2,228,318 | 58.31 | 8,031,687 | 47.65 | 455,998 | 38.15 | 8,696,222 | 55.03 | 504,792 | 43.20 |
|  |  |  |  |  |  |  |  |  |  |  |  |  |  |
| **rRNA** | RNA class | 218,429 | 1.42 | 17,156 | 0.11 | 175,101 | 1.04 | 15,272 | 0.09 | 135,774 | 0.86 | 14,584 | 0.09 |
| **tRNA** | RNA class | 43,488 | 0.28 | 7,764 | 0.05 | 27,185 | 0.16 | 5,766 | 0.03 | 38,869 | 0.25 | 6,502 | 0.04 |
| **snoRNA** | RNA class | 5,895 | 0.04 | 2,468 | 0.02 | 4,691 | 0.03 | 1,658 | 0.01 | 3,970 | 0.03 | 1,582 | 0.01 |
| **snRNA** | RNA class | 15,927 | 0.10 | 4,447 | 0.03 | 8,935 | 0.05 | 2,904 | 0.02 | 7,471 | 0.05 | 3,163 | 0.02 |
| **other Rfam RNA** | RNA class | 28,326 | 0.18 | 6,452 | 0.04 | 25,693 | 0.15 | 4,747 | 0.03 | 19,119 | 0.12 | 4,673 | 0.03 |

|  |  | **OO_a** |  |  |  | **OO_b** |  |  |  | **OO_c** |  |  |  |
| --- | --- | --- | --- | --- | --- | --- | --- | --- | --- | --- | --- | --- | --- |
| **lib** | type | Total | % of Total | uniq | % of uniq | Total | % of Total | uniq | % of uniq | Total | % of Total | uniq | % of uniq |
| **Raw reads** | NA | 10,741,096 | 100.00 | 1,766,844 | 100.00 | 9,864,856 | 100.00 | 1,275,917 | 100.00 | 12,639,427 | 100.00 | 1,734,912 | 100.00 |
| **3ADT&length filter** | Sequence type | 5,858,719 | 54.54 | 849,350 | 48.07 | 5,263,020 | 53.35 | 652,433 | 51.13 | 5,978,172 | 47.30 | 844,136 | 48.66 |
| **Junk reads** | Sequence type | 16,544 | 0.15 | 8,013 | 0.45 | 14,794 | 0.15 | 6,010 | 0.47 | 18,943 | 0.15 | 8,581 | 0.49 |
| **Rfam** | RNA class | 800,873 | 7.46 | 33,798 | 1.91 | 443,675 | 4.50 | 29,936 | 2.35 | 695,575 | 5.50 | 39,540 | 2.28 |
| **Repeats** | RNA class | 24,571 | 0.23 | 2,833 | 0.16 | 23,559 | 0.24 | 2,607 | 0.20 | 24,664 | 0.20 | 2,772 | 0.16 |
| **valid reads** | Sequence type | 4,050,730 | 37.71 | 874,137 | 49.47 | 4,128,477 | 41.85 | 586,068 | 45.93 | 5,932,435 | 46.94 | 841,143 | 48.48 |
|  |  |  |  |  |  |  |  |  |  |  |  |  |  |
| **rRNA** | RNA class | 742,726 | 6.91 | 19,290 | 0.18 | 394,013 | 3.99 | 17,252 | 0.17 | 628,439 | 4.97 | 24,092 | 0.19 |
| **tRNA** | RNA class | 19,974 | 0.19 | 5,649 | 0.05 | 17,176 | 0.17 | 4,829 | 0.05 | 19,745 | 0.16 | 5,746 | 0.05 |
| **snoRNA** | RNA class | 4,990 | 0.05 | 1,519 | 0.01 | 4,615 | 0.05 | 1,433 | 0.01 | 5,826 | 0.05 | 1,666 | 0.01 |
| **snRNA** | RNA class | 4,862 | 0.05 | 2,120 | 0.02 | 4,111 | 0.04 | 1,780 | 0.02 | 5,612 | 0.04 | 2,223 | 0.02 |
| **other Rfam RNA** | RNA class | 28,321 | 0.26 | 5,220 | 0.05 | 23,760 | 0.24 | 4,642 | 0.05 | 35,953 | 0.28 | 5,813 | 0.05 |

| Overview of reads from raw data to cleaned sequences. |
| --- |
| 3ADT&length filter: reads removed due to 3ADT not found and length with <18 nt and >25 nt were removed(for plants); length with<18 and >26 were remove(for animals) |
| Junk reads:Junk: >=2N, >=7A, >=8C, >=6G, >=7T, >=10Dimer, >=6Trimer, or >=5Tetramer |
| Rfam:Collection of many common non-coding RNA families except micro RNA; http://rfam.janelia.org |
| Repeats:Prototypic sequences representing repetitive DNA from different eukaryotic species; http://www.girinst.org/repbase. |
| Notes:valid reads may not be equal to raw reads - 3ADT&length filter - Junk reads ¨C mRNA ¨C Rfam - Repeats, because there are overlapped sequences between mRNA£¬Rfam and Repeats, details please refer to _comp_others.txt in fold 2_MappedData. |
