# Supplementary material for: Integrated analysis of mRNA-seq and miRNA-seq reveals the potential roles of sex-biased miRNA-mRNA pairs in gonad tissue of dark sleeper (Odontobutis potamophila)
Source: BMC Genomics. 2017 Aug 14;18:613. doi: 10.1186/s12864-017-3995-9 (PMC5557427; doi:10.1186/s12864-017-3995-9)
Supplement: Supplementary file 10 — List of miRNA member in each family in dark sleeper. (DOCX 23 kb) [file 12864_2017_3995_MOESM10_ESM.docx]

**Table S7** List of miRNA member in each family in dark sleeper

| AC | ID | MI | |
| --- | --- | --- | --- |
| MIPF0000001 | mir-17 | hsa-miR-106b-5p_R-1_1ss10GT,hsa-miR-106b-5p_R-1_1ss10GT,dre-miR-17a-5p,dre-miR-17a-5p_R+3_1ss14GC,dre-miR-17a-2-3p_2ss19CT20AC,dre-miR-17a-2-3p_L-1_1ss19CA,dre-miR-20a-5p_R-1,dre-miR-93,ola-miR-106a_R+2,cgr-miR-18a-5p_R-1,cgr-miR-18a-3p_R-2,ccr-miR-18c_R-3,ssa-miR-106b-3p_R-1_2ss1CA9AG,ssa-miR-20b-5p_1ss20GA | |
| MIPF0000002 | let-7 | hsa-let-7d-5p_1ss16CT,mmu-let-7f-1-3p_1ss22CT,dre-let-7a,dre-let-7b,dre-let-7c-3p,dre-let-7c-3p,dre-let-7d-5p,dre-let-7d-5p,dre-let-7e,dre-let-7i,tni-let-7h,cin-let-7e_R-4,ola-let-7a_R+1,ola-let-7a-3p_R+1,ipu-let-7f,pmi-let-7-5p_1ss10CA,hhi-let-7j,ssa-let-7h-5p,ssa-let-7h-3p,ssa-let-7h-3p,ssa-let-7i-2-3p_1ss22CT,ssa-let-7a-4-3p_R-1,ssa-let-7b-3p_R-1_1ss3GA,ssa-let-7e-3p_2ss20TC22CT,cfa-let-7d_R-3,oha-let-7i-5p_R+2_1,oha-let-7i-3p_1ss23AT,oha-let-7i-5p_R+2_2,oha-let-7i-3p_1ss23AT | |
| MIPF0000003 | mir-430 | dre-miR-430a-3p_R-4_1ss9AT,hhi-miR-430b | |
| MIPF0000005 | mir-30 | dre-miR-30e-5p_R-2,dre-miR-30e-3p,dre-miR-30e-5p_R-2,dre-miR-30e-3p,cgr-miR-30c,ipu-miR-30d_L-1 | |
| MIPF0000006 | mir-15 | dre-miR-457b-3p,bta-miR-195_R-2_1ss10AG,tgu-miR-16b-5p,tgu-miR-16b-5p,ccr-miR-457a_L+1R+1,ccr-miR-457b_L+1R-1,chi-miR-195-3p_L-1R-1_1ss12TA,oha-miR-16a-5p_1ss9TC,ssa-miR-15c-5p | |
| MIPF0000007 | mir-181 | gga-miR-181b-5p_R+1,gga-miR-181b-2-3p_L+1R-2,dre-miR-181b-3p_1ss21AG,pma-miR-181a-5p,pma-miR-181a-3p,pma-miR-181a-5p,pma-miR-181a-3p,hhi-miR-181b_R-1 | |
| MIPF0000009 | mir-29 | hsa-miR-29b-1-5p_3ss1GA11TG19TC,hsa-miR-29b-3p_R-1,hsa-miR-29b-2-5p_R+2_1ss10CG,oan-miR-29a-1-5p_R+1_1ss18TC,oan-miR-29a-3p_R+1,ola-miR-29b,cgr-miR-29c-3p_R+1,ipu-miR-29b,oha-miR-29a-3p | |
| MIPF0000011 | mir-19 | dre-miR-19a-5p_L+1R-1,dre-miR-19a-3p_R-2,dre-miR-19a-5p_L+1R-1,dre-miR-19a-3p_R-2,dre-miR-19b-5p_R+1_1ss21AC,dre-miR-19b-3p_R+2,pma-miR-19a_R-2_1ss13CT,ssa-miR-19c-3p_R-1,ssa-miR-19c-4-5p_R+1 | |
| MIPF0000013 | mir-25 | dre-miR-92a-5p,dre-miR-92a-5p,dre-miR-92a-3p_R+1,xtr-miR-92a_R+2,bta-miR-25,pma-miR-25b-3p_R-2_1ss16AG,bbe-miR-92d-3p_1ss11AG,ssa-miR-25-5p_2ss9TA23CT | |
| MIPF0000014 | mir-9 | pma-miR-9a-5p_R-1,pma-miR-9a-3p,oha-miR-9-4-5p_L-2R+3,oha-miR-9-3p_L-2R+1 | |
| MIPF0000018 | mir-154 | hsa-miR-409-5p_R-2,bta-miR-409a_R-2 | |
| MIPF0000019 | mir-8 | dre-miR-200a-5p_1ss10GT,gga-miR-429-5p_L-1_2ss12GA16AG,xtr-miR-429,aca-miR-200b-3p_R+1,aca-miR-200b-3p_R+1,ccr-miR-200a_R+2,efu-mir-200a-p3_1ss9TC | |
| MIPF0000021 | mir-124 | hsa-miR-124-5p_L-1R+1,hsa-miR-124-3p_R+1,dre-miR-124-5p_L-1R+1,gga-miR-124c-5p_R+1,ccr-miR-124a_R+2,efu-miR-124_L-1R-1 | |
| MIPF0000022 | mir-7 | fru-miR-7_R+3,ssa-miR-7a-3-3p_1ss22AT,ssa-miR-7a-5p_R+1,ssa-miR-7a-3-3p_1ss22AT | |
| MIPF0000024 | mir-103 | fru-miR-103,ssa-miR-103-5p_R-1_2ss11TC13AG | |
| MIPF0000026 | mir-218 | fru-miR-218a,ssa-miR-218-3p_L-1_1ss18AG | |
| MIPF0000027 | mir-23 | dre-miR-23a-3p_R-1,ssa-miR-23a-4-5p_L-1_1ss15AC | |
| MIPF0000028 | mir-135 | fru-miR-135b_R+1,dre-miR-135b-3p_L-1,dre-miR-135b-5p_R+1,dre-miR-135b-3p_L-1 | |
| MIPF0000029 | mir-133 | dre-miR-133a-5p,fru-miR-133_L-1R+1,ola-miR-133-5p_R-1,ola-miR-133-3p_L+1R+3,oha-miR-133b-3p_R-2 | |
| MIPF0000031 | mir-196 | age-miR-196 | |
| MIPF0000033 | mir-10 | dre-miR-10b-3p_L-2R+2,dre-miR-10b-5p_R-1,dre-miR-10b-3p_L-2R+2,dre-miR-10b-5p_R-1,dre-miR-100-2-3p_R-1,dre-miR-125b-2-3p_L+1_2ss8GA11TC,xtr-miR-10c_L-1R+1_1ss12AT,bta-miR-125b,cfa-miR-99b,tgu-miR-125-5p,tgu-miR-125-2-3p_R+1_1ss15TC,rno-miR-3588_L-1R+2,pma-miR-10a_L-1_1ss9GA,aca-miR-125a-5p,aca-miR-125a-3p_L-1_1ss12TC,ola-miR-99_R-1_1ss10CT,bbe-miR-100-5p,bbe-miR-10b-5p_L-1R-1_1ss9GA,ssa-miR-99-5p | |
| MIPF0000034 | mir-130 | dre-miR-130c-5p_1ss16TC,dre-miR-130c-5p_1ss4CT,dre-miR-130c-3p,dre-miR-301c-5p_R+1_2ss4CT9GA,fru-miR-301_R+1,ola-miR-130c_R+3,sha-miR-130a_R+1_1ss10GA,ccr-miR-130b,ssa-miR-130a-5p,ssa-miR-130a-2-3p_R-1,ssa-miR-130a-5p,ssa-miR-301a-3p | |
| MIPF0000036 | mir-27 | tni-miR-27e_R-1,cgr-miR-27a-3p,ccr-miR-27c-5p_R+1,ccr-miR-27c-3p_R-1 | |
| MIPF0000038 | mir-1 | gga-miR-1a-2-5p_R-1,gga-miR-1a-2-5p_R-1,gga-miR-1a-3p_R+1,gga-miR-1a-3p_R+1,dre-miR-206-5p_R-1_1ss17GC,pma-miR-1a-3p_R-1_1ss18CT,ola-miR-206_R+1 | |
| MIPF0000039 | mir-34 | hsa-miR-34a-3p_L-1R+1_2ss21CG22TC,hsa-miR-34c-5p_R-2_1ss16CT,dre-miR-34a,dre-miR-34c-5p_R-2_1ss9CT,dre-miR-34c-3p,cgr-miR-34c-5p_R-2_1ss16CT,oha-miR-34a-5p_R+3 | |
| MIPF0000040 | mir-199 | pma-miR-199a-5p_R-1,pma-miR-199a-5p_R-1,pma-miR-199b-3p_L+1R-1,pma-miR-199b-3p_L+1R-1 | |
| MIPF0000041 | mir-24 | ola-miR-24a_R+1,ssa-miR-24a-5p | |
| MIPF0000042 | mir-204 | oan-miR-204-5p,oan-miR-204-3p_L+1R+1_1ss21AC,aca-miR-204a-5p | |
| MIPF0000043 | mir-26 | hsa-miR-26a-5p,hsa-miR-26a-2-3p_1ss19TC,dre-miR-26a-2-3p_1ss8AT,hhi-miR-26_R+1,oha-miR-26-5p,oha-miR-26-2-3p_L-1R+2_1ss7CT | |
| MIPF0000044 | mir-219 | hsa-miR-219a-5p,dre-miR-219-5p,aca-miR-219-1-3p_L-1R+1_1ss12CA,aca-miR-219-5p_R-1,aca-miR-219-1-3p_L-1R+1_1ss12CA,aca-miR-219-5p_R-1 | |
| MIPF0000046 | mir-101 | dre-miR-101a_R-3_1ss12GA,ola-miR-101a-5p,ola-miR-101a-3p_L-1R+2 | |
| MIPF0000048 | mir-128 | oha-miR-128-5p_L-1R+2_2ss10AT19TA,oha-miR-128-3p_R-1 | |
| MIPF0000050 | mir-153 | dre-miR-153a-5p_L-1,cfa-miR-153_R+2 | |
| MIPF0000051 | mir-221 | dre-miR-221-5p_R-4,dre-miR-222a-5p_R-1_1ss11TG,fru-miR-222_R-1,bta-miR-221,aca-miR-222a-5p_L+1R-4,cgr-miR-221-5p_R-4,cgr-miR-221-3p_R-1,gga-miR-222b-5p_R-7,gga-miR-222b-3p_R-1,ipu-mir-221-2-p5_1ss1GT,ssa-miR-222a-5p_R-2,ssa-miR-222a-3p_R-1 | |
| MIPF0000053 | mir-22 | dre-miR-22a-5p_R-1,ola-miR-22_R+1 | |
| MIPF0000054 | mir-216 | tni-miR-216a,aca-miR-216b-5p_1ss1AT | |
| MIPF0000055 | mir-194 | aca-miR-194-5p,ola-miR-194-5p_R+3,ola-miR-194-3p | |
| MIPF0000056 | mir-148 | hsa-miR-152-5p_2ss2GA19CT,fru-miR-152_R-1 | |
| MIPF0000057 | mir-28 | hsa-miR-28-5p_R+1,bta-miR-28_R+1,cgr-miR-151-5p,cgr-miR-151-3p_1ss21GT,cgr-miR-151-5p,cgr-miR-151-3p_1ss21GT | |
| MIPF0000058 | mir-205 | dre-miR-205-3p_R-1_1ss20GA,dre-miR-205-5p_R-1,dre-miR-205-3p_R-1_1ss20GA,ccr-miR-205_R-1 | |
| MIPF0000059 | mir-184 | ola-miR-184-5p_L-1R+2,ccr-miR-184_R-2 | |
| MIPF0000060 | mir-21 | hsa-miR-21-5p,dre-miR-21_1ss23CA,dre-miR-21_L+3R-1,aja-miR-21_R-1,ipu-miR-21_R-1,ssa-miR-21a-2-3p,ssa-miR-21b-3p | |
| MIPF0000061 | mir-365 | aca-miR-365-5p_1ss10CT,aca-miR-365-3p,ccr-miR-365,ssa-miR-365-5p_R+3 | |
| MIPF0000062 | mir-214 | hsa-miR-3120-5p_L-1R+2,hsa-miR-3120-3p_L+2R-1_1ss18CT,hsa-miR-3120-5p_L-1R+2,hsa-miR-3120-3p_L+2R-1_1ss18CT,aca-miR-214-5p,aca-miR-214-3p | |
| MIPF0000063 | mir-192 | ola-miR-192-5p_L+1R-1,ola-miR-192-3p_R-1,ccr-miR-192,ssa-miR-192a-3p_1ss11AC | |
| MIPF0000064 | mir-31 | gga-miR-31-5p_R+1,pmi-miR-31-5p_R+2 | |
| MIPF0000065 | mir-132 | hsa-miR-212-5p,dre-miR-132-5p,xtr-miR-212,ccr-miR-132a,ipu-miR-212,ssa-miR-212a-3p,ssa-miR-212b-5p | |
| MIPF0000066 | mir-183 | chi-miR-183 | |
| MIPF0000067 | mir-223 | hsa-miR-223-5p_L-2R+3,hsa-miR-223-3p,dre-miR-223_R+1,ipu-miR-223_L-1R+4,oha-miR-223-5p,oha-miR-223-3p_R-3_1ss18TC | |
| MIPF0000069 | mir-32 | aca-miR-32-5p_R-1 | |
| MIPF0000070 | mir-33 | gga-miR-33-5p,gga-miR-33-3p_L+1_1ss19GA,bta-miR-33b_1ss10TA,ola-miR-33_R+2,ssa-miR-33b-5p_R-1 | |
| MIPF0000072 | mir-96 | dre-miR-96-3p,xtr-miR-96 | |
| MIPF0000073 | mir-129 | dre-miR-129-3p,dre-miR-129-3p,dre-miR-129-5p_R-1,dre-miR-129-5p_R-1,mmu-miR-129b-5p_R+1 | |
| MIPF0000075 | mir-138 | dre-miR-138-5p_R-2,dre-miR-138-3p_R+1,aca-miR-138-5p_R+1,aca-miR-138-2-3p_L-2R+1_1ss11AC | |
| MIPF0000076 | mir-190 | tni-miR-190_R+1,ssa-miR-190a-3p | |
| MIPF0000077 | mir-217 | fru-miR-217_R+1 | |
| MIPF0000078 | mir-187 | gga-miR-187-5p_R+1_1ss5AG,ola-miR-187_L+3R+4,ccr-miR-187 | |
| MIPF0000079 | mir-145 | dre-miR-145-3p,xtr-miR-145_R-2 | |
| MIPF0000080 | mir-127 | bta-miR-127 | |
| MIPF0000082 | mir-193 | dre-miR-193a-5p,dre-miR-193a-3p,dre-miR-193a-5p,dre-miR-193a-3p,cgr-miR-193b-3p_R-2_1ss9CT | |
| MIPF0000084 | mir-142 | dre-miR-142a-5p,dre-miR-142a-3p_L-1,dre-miR-142a-3p_L-1,ola-miR-142_L-2R+1 | |
| MIPF0000085 | mir-140 | rno-miR-140-5p,rno-miR-140-3p_L-1R+2,rno-miR-140-5p,rno-miR-140-3p_L-1R+2 | |
| MIPF0000086 | mir-210 | dre-miR-210-5p,dre-miR-210-3p,cgr-miR-210-3p_R+1_1ss21GT | |
| MIPF0000093 | mir-144 | dre-miR-144-5p_1ss11GT,dre-miR-144-3p_L+1,tni-miR-144_L+1,pma-miR-144-5p_L+1R-2_1ss11AC,pma-miR-144-3p_R-2_1ss10TG,ola-miR-144_L-1R+2 | |
| MIPF0000094 | mir-143 | dre-miR-143_R-3_1ss18GA,oha-miR-143-5p_L-1R-1,oha-miR-143-3p_1ss22AT | |
| MIPF0000095 | mir-122 | gga-miR-122-5p_R-2,gga-miR-122-3p_L+1R-1,fru-miR-122_R-1,hsa-miR-3591-5p_L+1R-1,ssa-miR-122-2-3p_L+1R-1 | |
| MIPF0000097 | mir-338 | ola-miR-338-5p_1ss6CT,ola-miR-338-3p_R+2,ola-miR-338-3p_R+2 | |
| MIPF0000103 | mir-146 | dre-miR-146b_R+1,ola-miR-146a-5p_R-2_1ss10AT,cgr-miR-146b-5p_R-4_1ss10AT,ssa-miR-146a-3p_R-1_2ss10CT20CT | |
| MIPF0000105 | mir-147 | hhi-miR-147b_L-1R-1_1ss12CT | |
| MIPF0000106 | mir-137 | dre-miR-137-5p_L+1R-4_1ss18GT,tni-miR-137_R-1,xbo-miR-137_L+1R-1_1ss18AG | |
| MIPF0000108 | mir-203 | dre-miR-203a-5p_R-2_1ss10TC,fru-miR-203,aca-miR-203-5p_R-1_1ss10TC,aca-miR-203-3p | |
| MIPF0000109 | mir-186 | bta-miR-186_R-1 | |
| MIPF0000111 | mir-489 | aca-miR-489-5p,aca-miR-489-3p_L-1R+1 |  |
| MIPF0000113 | mir-188 | hsa-miR-532-3p_R-1,bta-miR-532,cfa-miR-660,chi-miR-532-5p,chi-miR-532-3p | |
| MIPF0000114 | mir-375 | tni-miR-375 | |
| MIPF0000115 | mir-126 | ola-miR-126-5p,ola-miR-126-3p_R+2,ola-miR-126-5p,ola-miR-126-3p_R+2 | |
| MIPF0000116 | mir-182 | ccr-miR-182-5p_R-1,ccr-miR-182-3p_L-1R-1,ccr-miR-182-5p_R-1,ccr-miR-182-3p_L-1R-1 | |
| MIPF0000117 | mir-139 | tgu-miR-139-5p,ola-miR-139_R+2 | |
| MIPF0000121 | mir-202 | dre-miR-202-5p_R-1,dre-miR-202-3p_L+2R-2,dre-miR-202-5p_R-1_1ss17TG,ssc-miR-202-5p_L+2_1ss18TC,ssc-miR-202-5p_L+2_1ss18TC,ssa-miR-202-3p_R-1_1ss22GA,efu-miR-202_L-1R-1_2ss9TC13AG | |
| MIPF0000123 | mir-197 | bta-miR-197 | |
| MIPF0000126 | mir-379 | eca-miR-3959_R-1 | |
| MIPF0000128 | mir-450 | bta-miR-450a | |
| MIPF0000129 | mir-455 | dre-miR-455-5p,tni-miR-455,dre-miR-455-3p_L-2R+1,dre-miR-455-3p_L-2R+1 | |
| MIPF0000134 | mir-460 | dre-miR-730_R-2,gga-miR-460b-3p_R-2_2ss9AC21CG,ccr-miR-460-5p,ccr-miR-460-3p,ccr-miR-460-5p,ccr-miR-460-3p | |
| MIPF0000138 | mir-363 | hsa-miR-363-3p,dre-miR-363-3p,sha-miR-92a_L+1R+4 | |
| MIPF0000143 | mir-326 | bta-miR-326_R+1 | |
| MIPF0000148 | mir-451 | xtr-miR-451_R+1,pma-miR-451_R-4_1ss16TC,aca-miR-451-3p_L+2 | |
| MIPF0000157 | mir-155 | aca-miR-155-5p_R+1 | |
| MIPF0000163 | mir-320 | bta-miR-320a | |
| MIPF0000164 | mir-322 | cgr-miR-322-5p,cgr-miR-322-3p_L+1 | |
| MIPF0000168 | mir-378 | bta-miR-378 | |
| MIPF0000173 | mir-499 | dre-miR-499-5p_R-1,aca-miR-499-5p_R-1,aca-miR-499-3p,ola-miR-499_R+1,cgr-miR-499-5p_R-1,cgr-miR-499-3p | |
| MIPF0000174 | mir-454 | ccr-miR-454b_R+2,ssa-miR-454-5p_1ss16CT | |
| MIPF0000178 | mir-208 | ssa-miR-736-5p_L-3_1ss8CT,ssa-miR-736-3p | |
| MIPF0000179 | mir-458 | dre-miR-458-5p_R-1_2ss11AT21AT,fru-miR-458 | |
| MIPF0000185 | mir-542 | hsa-miR-542-3p,cfa-miR-542 | |
| MIPF0000191 | mir-340 | hsa-miR-340-5p,cfa-miR-340 | |
| MIPF0000193 | mir-339 | hsa-miR-339-5p_1ss23GA | |
| MIPF0000194 | mir-191 | chi-miR-191-5p,chi-miR-191-5p | |
| MIPF0000199 | mir-331 | aja-miR-331_R+1 | |
| MIPF0000202 | mir-185 | bta-miR-185_R-1,cgr-miR-185-5p_R-1 | |
| MIPF0000203 | mir-328 | bta-miR-328 | |
| MIPF0000208 | mir-466 | gga-mir-466-p3_1ss8AG,gga-mir-466-p5_1ss5AG,gga-mir-466-p3_1ss5AG,gga-mir-466-p3_1ss14AG | |
| MIPF0000213 | mir-541 | mmu-miR-541-5p_R-3 | |
| MIPF0000219 | mir-484 | bta-miR-484_R+1 | |
| MIPF0000220 | mir-486 | cgr-miR-486-5p,cgr-miR-486-5p | |
| MIPF0000231 | mir-497 | bta-miR-497_R+1 | |
| MIPF0000242 | mir-425 | bta-miR-425-5p_L+1R+1,bta-miR-425-5p_L+1R+1 | |
| MIPF0000288 | mir-374 | hsa-miR-374a-5p_R-1,bta-miR-374a_R-1 | |
| MIPF0000316 | mir-467 | mmu-mir-466i-p5_1ss3GA,mmu-miR-466i-5p_L-2,mmu-miR-466i-5p_R-1,mmu-mir-467g-p5,mmu-mir-467g-p3,hsa-mir-466-p3_1ss13GA | |
| MIPF0000329 | mir-423 | bta-miR-423-5p_R-2,efu-miR-423_R-3 | |
| MIPF0000341 | mir-456 | aca-miR-456_R+1 | |
| MIPF0000360 | mir-551 | ccr-miR-551_1ss14GA | |
| MIPF0000397 | mir-708 | hsa-miR-708-3p_R+1 | |
| MIPF0000419 | mir-574 | hsa-miR-574-5p_L-6R+1_1ss21TG,ggo-miR-574 | |
| MIPF0000483 | mir-1271 | efu-miR-1271_1ss22AT | |
| MIPF0000509 | mir-92 | csa-miR-92c_R-2_1ss9CT | |
| MIPF0000531 | mir-1306 | dre-mir-1306-p3_1ss22GT,chi-miR-1306-5p_R+1,chi-miR-1306-5p_R+1 | |
| MIPF0000532 | mir-885 | bta-miR-885,eca-miR-885-5p | |
| MIPF0000533 | mir-584 | hsa-miR-584-3p_L-2R-2_1ss6TA,ggo-miR-584 | |
| MIPF0000558 | mir-1307 | hsa-miR-1307-5p,bta-miR-1307_R+1,chi-miR-1307-5p_R+4,chi-miR-1307-3p | |
| MIPF0000579 | mir-1273 | ssc-mir-1285-p5_1ss18CA | |
| MIPF0000667 | mir-1249 | cfa-miR-1249 | |
| MIPF0000777 | mir-1468 | bta-miR-1468 | |
| MIPF0000780 | mir-606 | hsa-miR-606_L-1R-1_1ss12AT | |
| MIPF0000788 | mir-1842 | efu-miR-1842_R-3 | |
| MIPF0000792 | mir-1788 | aca-miR-1788-5p_R-1_2ss11CA21CT,aca-miR-1788-3p_R+1,aca-miR-1788-5p_R-1_2ss11CA21CT,aca-miR-1788-3p_R+1 | |
| MIPF0000805 | mir-1388 | ola-miR-1388-5p,ola-miR-1388-3p_R+4,ola-miR-1388-5p,ola-miR-1388-3p_R+4 | |
| MIPF0000812 | mir-2188 | ssa-miR-2188-5p,ssa-miR-2188-3p_1ss14AG | |
| MIPF0001020 | mir-1260a | hsa-miR-1260a_1ss9TG | |
| MIPF0001032 | mir-2954 | gga-miR-2954_R+2 | |
| MIPF0001049 | mir-1451 | gga-miR-1451-5p | |
| MIPF0001050 | mir-3604 | bta-miR-3604 | |
| MIPF0001102 | mir-3150 | hsa-miR-3150b-5p_L-4_1ss9AC | |
| MIPF0001119 | mir-1843 | mmu-miR-1843b-5p_L+1,mmu-miR-1843b-5p_L+1 | |
| MIPF0001206 | mir-1343 | bta-miR-1343-3p_L-3R+2 | |
| MIPF0001261 | mir-3529 | gga-miR-3529_1ss11TG | |
| MIPF0001322 | mir-459 | ccr-miR-459-5p_L+2R-1 | |
| MIPF0001328 | mir-2970 | tgu-miR-2970-5p_1ss21GT | |
| MIPF0001344 | mir-726 | aca-miR-726_R+1 | |
| MIPF0001355 | mir-725 | ssa-miR-725-3p_1ss20AC | |
| MIPF0001371 | mir-727 | ccr-miR-727-5p_1ss11AG,ccr-miR-727-3p_L-1R+1_1ss22AT | |
| MIPF0001445 | mir-6134 | mml-miR-6134_R+3_1ss18GT | |
| MIPF0001514 | mir-738 | ccr-miR-738_R+3 | |
| MIPF0001540 | mir-3615 | hsa-miR-3615_R-1 | |
| MIPF0001543 | mir-734 | ccr-miR-734 | |
| MIPF0001555 | mir-629 | hsa-miR-629-5p_1ss10GC | |
| MIPF0001592 | mir-1957 | mmu-miR-1957a_R-1_1ss15AT | |
| MIPF0001612 | mir-722 | ccr-miR-722_L-2R+3,ssa-miR-722-5p_L-1R+1 | |
| MIPF0001616 | mir-724 | ccr-miR-724,ssa-miR-724-3p | |
| MIPF0001670 | mir-728 | hhi-miR-728_R+1 | |
| MIPF0001672 | mir-6516 | gga-miR-6516-3p_R-3_1ss11AG | |
| MIPF0001689 | mir-737 | dre-miR-737-5p_L-2R+1,dre-miR-737-3p | |
| MIPF0001705 | mir-7147 | dre-miR-7147 | |
| MIPF0001755 | mir-462 | dre-miR-462_1ss12AT | |
| MIPF0001770 | mir-2187 | dre-miR-2187-5p_R-1,dre-miR-2187-3p_L+1R-1_1ss21AG | |
| MIPF0001825 | mir-7641 | hsa-miR-7641_L+2_1ss3TC | |
| MIPF0001831 | mir-723 | ssa-miR-723-3p | |
| MIPF0001916 | mir-7552 | ssa-miR-7552a-5p,ssa-miR-7552a-5p | |
| MIPF0001935 | mir-3149 | hsa-miR-3149_L-2_1ss9GT | |
| MIPF0001937 | mir-1277 | hsa-miR-1277-5p_L-4R-1_1ss20CT,hsa-miR-1277-5p_L-4R-1_1ss20CT | |
| MIPF0001989 | mir-2002 | lva-miR-2002-3p_L-2R-3_1ss5TC | |
| MIPF0002062 | mir-8986 | eca-mir-8986b-p3_1ss19CG | |
| MIPF0002098 | mir-7132 | ccr-miR-7132_R+1_1ss10TA | |
