# Supplementary material for: Integrated analysis of mRNA-seq and miRNA-seq reveals the potential roles of sex-biased miRNA-mRNA pairs in gonad tissue of dark sleeper (Odontobutis potamophila)
Source: BMC Genomics. 2017 Aug 14;18:613. doi: 10.1186/s12864-017-3995-9 (PMC5557427; doi:10.1186/s12864-017-3995-9)
Supplement: Supplementary file 11 — List of know-miRNA and novel-miRNA of dark sleeper. (DOCX 57 kb) [file 12864_2017_3995_MOESM11_ESM.docx]

**Table S8** List of know-miRNA and novel-miRNA of dark sleeper

| miR_name | miR_seq | len |
| --- | --- | --- |
|  |  |  |
| ipu-let-7f | TGAGGTAGTAGATTGTATAGTT | 22 |
| mmu-let-7f-1-3p_1ss22CT | CTATACAATCTATTGCCTTCCT | 22 |
| ssa-miR-19c-4-5p_R+1 | AGTTTTGCTGGTTTGCTTTCAGC | 23 |
| ssa-miR-19c-3p_R-1 | TGTGCAAATCCATGCAAAACT | 21 |
| ipu-miR-21_R-1 | TAGCTTATCAGACTGGTGTTGG | 22 |
| ssa-miR-21b-3p | CGACAACAGTCTGTAGGCTGTC | 22 |
| dre-miR-202-5p_R-1 | TTCCTATGCATATACCTCTTT | 21 |
| dre-miR-202-3p_L+2R-2 | AAAGAGGCATAGGGCATGGGAA | 22 |
| dre-miR-210-5p | AGCCACTGACTAACGCACATTG | 22 |
| dre-miR-210-3p | CTGTGCGTGTGACAGCGGCTAA | 22 |
| ssc-mir-1285-p5_1ss18CA | ATCGCGCCTGTGAATAGAC | 19 |
| dre-miR-17a-5p | CAAAGTGCTTACAGTGCAGGTA | 22 |
| dre-miR-17a-2-3p_2ss19CT20AC | ACTGCAGTGGAGGCACTTTCAGC | 23 |
| dre-miR-20a-5p_R-1 | TAAAGTGCTTATAGTGCAGGTA | 22 |
| dre-miR-21_1ss23CA | TAGCTTATCAGACTGGTGTTGGA | 23 |
| ssa-miR-21a-2-3p | CGACAACAGTCTGTAAGCTGTC | 22 |
| dre-miR-34a | TGGCAGTGTCTTAGCTGGTTGT | 22 |
| hsa-miR-34a-3p_L-1R+1_2ss21CG22TC | AATCAGCAAGTATACTGCCGCA | 22 |
| dre-miR-143_R-3_1ss18GA | TGAGATGAAGCACTGTAA | 18 |
| dre-miR-202-5p_R-1_1ss17TG | TTCCTATGCATATACCGCTTT | 21 |
| efu-miR-202_L-1R-1_2ss9TC13AG | AAAGAGGCGTAGGGCATGGGAAA | 23 |
| gga-mir-466-p3_1ss8AG | TGTATATGTATATATATATATA | 22 |
| gga-mir-466-p5_1ss5AG | ATATGTGTATGTATGTATATA | 21 |
| gga-mir-466-p3_1ss5AG | ATGTGTGTATATATATATATATA | 23 |
| gga-mir-466-p3_1ss14AG | TATGTATATATATGTATATATA | 22 |
| mmu-mir-467g-p5 | TACATATATATACATACATA | 20 |
| hsa-mir-466-p3_1ss13GA | GTGTATATGTGTATATATA | 19 |
| mmu-mir-467g-p3 | TACATATATATACATACATA | 20 |
| hsa-mir-620-p5_1ss2CA | CAGTATATATATATATATA | 19 |
| mmu-miR-1187_R-3 | TATGTGTGTGTGTATGTGTG | 20 |
| dre-mir-1306-p3_1ss22GT | ACGTTAGCTCTGGTGGTGATGT | 22 |
| mmu-mir-1983-p3_1ss5TC | AGGTCGTGAGTTCGAGCCTCA | 21 |
| eca-mir-8986b-p3_1ss19CG | GTCCTGGTGTGCTAGAGTGCTCG | 23 |
| dre-let-7a | TGAGGTAGTAGGTTGTATAGTT | 22 |
| ssa-let-7a-4-3p_R-1 | CTATACAGTCTATTGCCTTCC | 21 |
| dre-let-7i | TGAGGTAGTAGTTTGTGCTGTT | 22 |
| ssa-let-7i-2-3p_1ss22CT | CTGCGCAAGCTACTGCCTTGCT | 22 |
| dre-let-7e | TGAGGTAGTAGATTGAATAGTT | 22 |
| ssa-let-7e-3p_2ss20TC22CT | CTATACAATCTACTGTCTTCCT | 22 |
| dre-let-7b | TGAGGTAGTAGGTTGTGTGGTT | 22 |
| ssa-let-7b-3p_R-1_1ss3GA | CTATACAACCTACTGCCTTCC | 21 |
| dre-miR-101a_R-3_1ss12GA | TACAGTACTGTAATAACTG | 19 |
| dre-miR-138-5p_R-2 | AGCTGGTGTTGTGAATCAGG | 20 |
| dre-miR-138-3p_R+1 | GCTATTTCACAACACCAGGGTT | 22 |
| efu-mir-200a-p3_1ss9TC | CCGGGTGCCCGGCCGGCA | 18 |
| ipu-mir-221-2-p5_1ss1GT | TGTCTGTGTGTGTGTGTGTC | 20 |
| mmu-mir-466i-p5_1ss3GA | GTATGTGTATATATATACA | 19 |
| hsa-miR-574-5p_L-6R+1_1ss21TG | TGTGTGTGTGAGTGGGTG | 18 |
| hsa-miR-1260a_1ss9TG | ATCCCACCGCTGCCACCA | 18 |
| gga-miR-1a-2-5p_R-1 | ACATACTTCTTTATGTACCCAT | 22 |
| gga-miR-1a-3p_R+1 | TGGAATGTAAAGAAGTATGTAT | 22 |
| gga-miR-1a-2-5p_R-1 | ACATACTTCTTTATGTACCCAT | 22 |
| gga-miR-1a-3p_R+1 | TGGAATGTAAAGAAGTATGTAT | 22 |
| pma-miR-1a-3p_R-1_1ss18CT | TGGAATGTAAAGAAGTATGTT | 21 |
| fru-miR-7_R+3 | TGGAAGACTAGTGATTTTGTTGTT | 24 |
| ssa-miR-7a-3-3p_1ss22AT | CAACAAATCACAGTCTGCCAAT | 22 |
| ssa-miR-7a-5p_R+1 | TGGAAGACTAGTGATTTTGTTGTT | 24 |
| ssa-miR-7a-3-3p_1ss22AT | CAACAAATCACAGTCTGCCAAT | 22 |
| pma-miR-9a-5p_R-1 | TCTTTGGTTATCTAGCTGTAT | 21 |
| pma-miR-9a-3p | TAAAGCTAGATAACCGAAAGTA | 22 |
| oha-miR-9-4-5p_L-2R+3 | TTTCGGTTATCTAGCTTTATGA | 22 |
| oha-miR-9-3p_L-2R+1 | ATACAGCTAGATAACCAAAGAT | 22 |
| dre-miR-10b-5p_R-1 | TACCCTGTAGAACCGAATTTGT | 22 |
| dre-miR-10b-3p_L-2R+2 | AGATTCGATTCTAGGGGAGTAT | 22 |
| dre-miR-10b-5p_R-1 | TACCCTGTAGAACCGAATTTGT | 22 |
| dre-miR-10b-3p_L-2R+2 | AGATTCGATTCTAGGGGAGTAT | 22 |
| bbe-miR-10b-5p_L-1R-1_1ss9GA | ACCCTGTAGATCCGATCTTGTG | 22 |
| xtr-miR-10c_L-1R+1_1ss12AT | ACCCTGTAGATTCGAATTTGTG | 22 |
| pma-miR-10a_L-1_1ss9GA | ACCCTGTAGACTCGAATTTGT | 21 |
| ssa-miR-15c-5p | TAGCAGCGCATCATGGTTTGA | 21 |
| tgu-miR-16b-5p | TAGCAGCACGTAAATATTGGAG | 22 |
| tgu-miR-16b-5p | TAGCAGCACGTAAATATTGGAG | 22 |
| oha-miR-16a-5p_1ss9TC | TAGCAGCACATAAATATTGGAG | 22 |
| dre-miR-17a-5p_R+3_1ss14GC | CAAAGTGCTTACACTGCAGGTAGTT | 25 |
| dre-miR-17a-2-3p_L-1_1ss19CA | CTGCAGTGGAGGCACTTAAAGC | 22 |
| cgr-miR-18a-5p_R-1 | TAAGGTGCATCTAGTGCAGATA | 22 |
| cgr-miR-18a-3p_R-2 | ACTGCCCTAAGTGCTCCTTCT | 21 |
| ccr-miR-18c_R-3 | TAAGGTGCATCTTGTGTAGT | 20 |
| dre-miR-19a-5p_L+1R-1 | GCTAGTTTTGCATAGTTGCACT | 22 |
| dre-miR-19a-3p_R-2 | TGTGCAAATCTATGCAAAACT | 21 |
| dre-miR-19a-5p_L+1R-1 | GCTAGTTTTGCATAGTTGCACT | 22 |
| dre-miR-19a-3p_R-2 | TGTGCAAATCTATGCAAAACT | 21 |
| dre-miR-19b-5p_R+1_1ss21AC | AGTTTTGCTGGTTTGCATTCCGC | 23 |
| dre-miR-19b-3p_R+2 | TGTGCAAATCCATGCAAAACTGATT | 25 |
| pma-miR-19a_R-2_1ss13CT | TGTGCAAACCTATGCAAAGCT | 21 |
| ssa-miR-20b-5p_1ss20GA | CAAAGTGCTCACAGTGCAGATA | 22 |
| hsa-miR-21-5p | TAGCTTATCAGACTGATGTTGA | 22 |
| aja-miR-21_R-1 | CAACAGCAGTCGATGGGCTGT | 21 |
| dre-miR-21_L+3R-1 | ATCTAGCTTATCAGACTGGTGTTGG | 25 |
| mmu-miR-21c_R-1_1ss19CA | TAGCTTATCAGACTGGTAAA | 20 |
| dre-miR-22a-5p_R-1 | AGTTCTTCACTGGCAAGCTTT | 21 |
| ola-miR-22_R+1 | AAGCTGCCAGCTGAAGAACTGT | 22 |
| ssa-miR-23a-4-5p_L-1_1ss15AC | GGGTTCCTGGCACCGTGATTT | 21 |
| dre-miR-23a-3p_R-1 | ATCACATTGCCAGGGATTTCC | 21 |
| ssa-miR-24a-5p | TGCCTACTGAACTGGTATCAGT | 22 |
| ola-miR-24a_R+1 | TGGCTCAGTTCAGCAGGAACAGT | 23 |
| ssa-miR-25-5p_2ss9TA23CT | AGGCGGAGACTTGGGCAATTGCT | 23 |
| bta-miR-25 | CATTGCACTTGTCTCGGTCTGA | 22 |
| pma-miR-25b-3p_R-2_1ss16AG | CATTGCACTAGTCTCGGTCT | 20 |
| hhi-miR-26_R+1 | TTCAAGTAATCCAGGATAGGCT | 22 |
| dre-miR-26a-2-3p_1ss8AT | CCTATTCTTGATTACTTGCACT | 22 |
| hsa-miR-26a-5p | TTCAAGTAATCCAGGATAGGCT | 22 |
| hsa-miR-26a-2-3p_1ss19TC | CCTATTCTTGATTACTTGCTTC | 22 |
| oha-miR-26-5p | TTCAAGTAATCCAGGATAGGCT | 22 |
| oha-miR-26-2-3p_L-1R+2_1ss7CT | CCTATTCTTGATTACTTGCACT | 22 |
| ccr-miR-27c-5p_R+1 | CAGGACTTAACCCACTTGTGAACA | 24 |
| ccr-miR-27c-3p_R-1 | TTCACAGTGGTTAAGTTCTGC | 21 |
| cgr-miR-27a-3p | TTCACAGTGGCTAAGTTCCGC | 21 |
| tni-miR-27e_R-1 | TTCACAGTGGCTAAGTTCAGT | 21 |
| bta-miR-28_R+1 | AAGGAGCTCACAGTCTATTGAGA | 23 |
| hsa-miR-28-5p_R+1 | AAGGAGCTCACAGTCTATTGAGA | 23 |
| oan-miR-29a-1-5p_R+1_1ss18TC | ACTGATTTCTTTTGGTGCTCAGA | 23 |
| oan-miR-29a-3p_R+1 | TAGCACCATTTGAAATCGGTTA | 22 |
| hsa-miR-29b-1-5p_3ss1GA11TG19TC | ACTGGTTTCAGATGGTGGCTTAGA | 24 |
| ola-miR-29b | TAGCACCATTTGAAATCAGTGT | 22 |
| hsa-miR-29b-2-5p_R+2_1ss10CG | CTGGTTTCAGATGGTGGCTTAGAT | 24 |
| hsa-miR-29b-3p_R-1 | TAGCACCATTTGAAATCAGTGT | 22 |
| cgr-miR-29c-3p_R+1 | TAGCACCATTTGAAATCGGTTA | 22 |
| oha-miR-29a-3p | TAGCACCATTTGAAATCGGTTA | 22 |
| ipu-miR-29b | GCTGAATTCAGATGGTGCCATAGA | 24 |
| dre-miR-30e-5p_R-2 | TGTAAACATCCTTGACTGGA | 20 |
| dre-miR-30e-3p | CTTTCAGTCGGATGTTTGCAGC | 22 |
| dre-miR-30e-5p_R-2 | TGTAAACATCCTTGACTGGA | 20 |
| dre-miR-30e-3p | CTTTCAGTCGGATGTTTGCAGC | 22 |
| cgr-miR-30c | TGTAAACATCCTACACTCTCAGC | 23 |
| ipu-miR-30d_L-1 | TTTCAGTTGGATGTTTGCTGT | 21 |
| pmi-miR-31-5p_R+2 | AGGCAAGATGTTGGCATAGCTGT | 23 |
| gga-miR-31-5p_R+1 | AGGCAAGATGTTGGCATAGCTGT | 23 |
| aca-miR-32-5p_R-1 | TATTGCACATTACTAAGTTGC | 21 |
| gga-miR-33-5p | GTGCATTGTAGTTGCATTGC | 20 |
| gga-miR-33-3p_L+1_1ss19GA | CAATGTTCCTGCAGTGCAATA | 21 |
| ssa-miR-33b-5p_R-1 | GTGCATTGTAGTTGCATTGC | 20 |
| ola-miR-33_R+2 | CAATGTACCTGCAGTGCAACA | 21 |
| bta-miR-33b_1ss10TA | GTGCATTGCAGTTGCATTGC | 20 |
| dre-miR-34c-5p_R-2_1ss9CT | AGGCAGTGTAGTTAGTTGATT | 21 |
| dre-miR-34c-3p | AATCACTAACCTCACTACCAGG | 22 |
| hsa-miR-34c-5p_R-2_1ss16CT | AGGCAGTGTAGTTAGTTGATT | 21 |
| cgr-miR-34c-5p_R-2_1ss16CT | AGGCAGTGTAGTTAGTTGATT | 21 |
| oha-miR-34a-5p_R+3 | TGGCAGTGTCTTAGCTGGTTGTTAGA | 26 |
| dre-miR-92a-5p | AGGTTGGGATTGGTAGCAATGCT | 23 |
| xtr-miR-92a_R+2 | TATTGCACTTGTCCCGGCCTGTT | 23 |
| dre-miR-92a-5p | AGGTTGGGATTGGTAGCAATGCT | 23 |
| dre-miR-92a-3p_R+1 | TATTGCACTTGTCCCGGCCTGTT | 23 |
| bbe-miR-92d-3p_1ss11AG | TATTGCACTTGTCCTGGCCTGT | 22 |
| csa-miR-92c_R-2_1ss9CT | TATTGCACTTGTCCCGGCCG | 20 |
| sha-miR-92a_L+1R+4 | CTATTGCACTTGTCCCGGCCTGT | 23 |
| dre-miR-93 | AAAAGTGCTGTTTGTGCAGGTA | 22 |
| xtr-miR-96 | TTTGGCACTAGCACATTTTTGCT | 23 |
| dre-miR-96-3p | CAATTATGTGTAGTGCCAATAT | 22 |
| ssa-miR-99-5p | AACCCGTAGATCCGATCTTGTG | 22 |
| ola-miR-99_R-1_1ss10CT | CAAGCTCGCTTCTGTGGGTCT | 21 |
| cfa-miR-99b | CACCCGTAGAACCGACCTTGCG | 22 |
| bbe-miR-100-5p | AACCCGTAGATCCGAACTTGTG | 22 |
| dre-miR-100-2-3p_R-1 | CAAGCTCGTGTCTATAGGTAT | 21 |
| ola-miR-101a-5p | TCAGTTATCACAGTGCTGATGC | 22 |
| ola-miR-101a-3p_L-1R+2 | TACAGTACTGTGATAACTGAAG | 22 |
| cin-miR-101_R-3_1ss17AC | TACAGTACTGTGATAACTA | 19 |
| ssa-miR-103-5p_R-1_2ss11TC13AG | AGCCTCTTTACGGTGCTGCCTTG | 23 |
| fru-miR-103 | AGCAGCATTGTACAGGGCTATGA | 23 |
| ola-miR-106a_R+2 | TAAAGTGCTTACAGTGCAGGTAT | 23 |
| ssa-miR-106b-3p_R-1_2ss1CA9AG | ACTGCAGTGTGAGCACTTCTTTC | 23 |
| hsa-miR-106b-5p_R-1_1ss10GT | TAAAGTGCTTACAGTGCAGA | 20 |
| hsa-miR-106b-5p_R-1_1ss10GT | TAAAGTGCTTACAGTGCAGA | 20 |
| fru-miR-122_R-1 | TGGAGTGTGACAATGGTGTTT | 21 |
| ssa-miR-122-2-3p_L+1R-1 | AAACGCCATTATCACACTAAAT | 22 |
| gga-miR-122-5p_R-2 | TGGAGTGTGACAATGGTGTTT | 21 |
| gga-miR-122-3p_L+1R-1 | AAACGCCATTATCACACTAAAT | 22 |
| dre-miR-124-5p_L-1R+1 | GTGTTCACAGCGGACCTTGATT | 22 |
| efu-miR-124_L-1R-1 | TAAGGCACGCGGTGAATGCCA | 21 |
| hsa-miR-124-5p_L-1R+1 | GTGTTCACAGCGGACCTTGATT | 22 |
| hsa-miR-124-3p_R+1 | TAAGGCACGCGGTGAATGCCA | 21 |
| gga-miR-124c-5p_R+1 | CATTCACCGCGTGCCTTAATTG | 22 |
| ccr-miR-124a_R+2 | TCAAGGTCCGCTGTGAACACGA | 22 |
| bta-miR-125b | TCCCTGAGACCCTAACTTGTGA | 22 |
| dre-miR-125b-2-3p_L+1_2ss8GA11TC | ACGGGTTAGGCTCTCGGGAGCT | 22 |
| tgu-miR-125-5p | TCCCTGAGACCCTAACTTGTGA | 22 |
| tgu-miR-125-2-3p_R+1_1ss15TC | ACGGGTTAGGCTCTCGGGAGCT | 22 |
| aca-miR-125a-5p | TCCCTGAGACCCTTAACCTGTG | 22 |
| aca-miR-125a-3p_L-1_1ss12TC | CAGGTGAGGTCCTTGGGAACT | 21 |
| cin-miR-125-5p_R-3_1ss18CG | TCCCTGAGACCCTAAAAG | 18 |
| ola-miR-126-5p | CATTATTACTTTTGGTACGCG | 21 |
| ola-miR-126-3p_R+2 | TCGTACCGTGAGTAATAATGCA | 22 |
| ola-miR-126-5p | CATTATTACTTTTGGTACGCG | 21 |
| ola-miR-126-3p_R+2 | TCGTACCGTGAGTAATAATGCA | 22 |
| bta-miR-127 | TCGGATCCGTCTGAGCTTGGCT | 22 |
| oha-miR-128-5p_L-1R+2_2ss10AT19TA | GGGGCCGTTACACTGTCAGAGA | 22 |
| oha-miR-128-3p_R-1 | TCACAGTGAACCGGTCTCTTT | 21 |
| dre-miR-129-5p_R-1 | CTTTTTGCGGTCTGGGCTTGC | 21 |
| dre-miR-129-3p | AAGCCCTTACCCCAAAAAGCAT | 22 |
| dre-miR-129-5p_R-1 | CTTTTTGCGGTCTGGGCTTGC | 21 |
| dre-miR-129-3p | AAGCCCTTACCCCAAAAAGCAT | 22 |
| mmu-miR-129b-5p_R+1 | GCTTTTTGGGGTAAGGGCTTCCT | 23 |
| dre-miR-130c-5p_1ss16TC | GCCCTTTTTCTGTTGCACTACT | 22 |
| ola-miR-130c_R+3 | CAGTGCAATATTAAAAGGGCAT | 22 |
| dre-miR-130c-5p_1ss4CT | GCCTTTTTTCTGTTGTACTACT | 22 |
| dre-miR-130c-3p | CAGTGCAATATTAAAAGGGCAT | 22 |
| ssa-miR-130a-5p | ACTCTTTCCCTGTTGCACTACT | 22 |
| ssa-miR-130a-2-3p_R-1 | CAGTGCAATAATGAAAGGGCAT | 22 |
| ssa-miR-130a-5p | ACTCTTTCCCTGTTGCACTACT | 22 |
| ccr-miR-130b | CAGTGCAATAATGAAAGGGCAT | 22 |
| sha-miR-130a_R+1_1ss10GA | CAGTGCAATATAAAAAGGGCAT | 22 |
| dre-miR-132-5p | ACCGTGGCATTAGATTGTTACT | 22 |
| ccr-miR-132a | TAACAGTCTACAGCCATGGTCG | 22 |
| ccr-miR-132b_R-1 | ACCATGGCTGTAGACTGTTAC | 21 |
| dre-miR-133a-5p | AGCTGGTAAAATGGAACCAAAT | 22 |
| fru-miR-133_L-1R+1 | TTGGTCCCCTTCAACCAGCTGT | 22 |
| ola-miR-133-5p_R-1 | AGCTGGTAAAATGGAACCAAAT | 22 |
| ola-miR-133-3p_L+1R+3 | TTGGTCCCCTTCAACCAGCTGT | 22 |
| oha-miR-133b-3p_R-2 | TTTGGTCCCCTTCAACCAGCT | 21 |
| fru-miR-135b_R+1 | TATGGCTTTTTATTCCTATCTGA | 23 |
| dre-miR-135b-3p_L-1 | TATAGGGATGGAAGCCATGCA | 21 |
| dre-miR-135b-5p_R+1 | TATGGCTTTTTATTCCTATCTGA | 23 |
| dre-miR-135b-3p_L-1 | TATAGGGATGGAAGCCATGCA | 21 |
| dre-miR-137-5p_L+1R-4_1ss18GT | CACGGGTATTCTTGGGTTGAT | 21 |
| tni-miR-137_R-1 | TTATTGCTTGAGAATACGCGT | 21 |
| xbo-miR-137_L+1R-1_1ss18AG | TTATTGCTTGAGAATACGCGTT | 22 |
| aca-miR-138-5p_R+1 | AGCTGGTGTTGTGAATCAGGCCG | 23 |
| aca-miR-138-2-3p_L-2R+1_1ss11AC | GCTACTTCCCAACACCAGGGT | 21 |
| ola-miR-139_R+2 | TCTACAGTGCATGTGTCTCCAGT | 23 |
| tgu-miR-139-5p | TCTACAGTGCATGTGTCTCCAGT | 23 |
| rno-miR-140-5p | CAGTGGTTTTACCCTATGGTAG | 22 |
| rno-miR-140-3p_L-1R+2 | ACCACAGGGTAGAACCACGGAC | 22 |
| rno-miR-140-5p | CAGTGGTTTTACCCTATGGTAG | 22 |
| rno-miR-140-3p_L-1R+2 | ACCACAGGGTAGAACCACGGAC | 22 |
| dre-miR-142a-5p | CATAAAGTAGAAAGCACTACT | 21 |
| dre-miR-142a-3p_L-1 | GTAGTGTTTCCTACTTTATGGA | 22 |
| ola-miR-142_L-2R+1 | CATAAAGTAGAAAGCACTACT | 21 |
| dre-miR-142a-3p_L-1 | GTAGTGTTTCCTACTTTATGGA | 22 |
| oha-miR-143-5p_L-1R-1 | GGTGCAGTGCTGCATCTCTGG | 21 |
| oha-miR-143-3p_1ss22AT | TGAGATGAAGCACTGTAGCTCT | 22 |
| dre-miR-144-5p_1ss11GT | GGATATCATCTTATACTGTAAGT | 23 |
| tni-miR-144_L+1 | CTACAGTATAGATGATGTACT | 21 |
| ola-miR-144_L-1R+2 | GGATATCATCTTATACTGTAAGT | 23 |
| dre-miR-144-3p_L+1 | CTACAGTATAGATGATGTACT | 21 |
| pma-miR-144-5p_L+1R-2_1ss11AC | AGGATATCATCCTATACTGTA | 21 |
| pma-miR-144-3p_R-2_1ss10TG | TACAGTATAGATGATGTGCT | 20 |
| xtr-miR-145_R-2 | GTCCAGTTTTCCCAGGAATCCC | 22 |
| dre-miR-145-3p | GGATTCCTGGAAATACTGTTCT | 22 |
| mmu-miR-145b_R-2_1ss18GA | GTCCAGTTTTCCCAGGAAA | 19 |
| ola-miR-146a-5p_R-2_1ss10AT | TGAGAACTGTATTCCATAGATGG | 23 |
| ssa-miR-146a-3p_R-1_2ss10CT20CT | ATCTATGGGTTCAGTTCTTTTG | 22 |
| dre-miR-146b_R+1 | TGAGAACTGAATTCCAAGGGTGT | 23 |
| cgr-miR-146b-5p_R-4_1ss10AT | TGAGAACTGTATTCCATAG | 19 |
| hhi-miR-147b_L-1R-1_1ss12CT | TGTGCGGAAATGCTTCTGCTC | 21 |
| ola-miR-150_R+1 | ACTCCCAATCCTTGTACCAGTG | 22 |
| cgr-miR-151-5p | TCGAGGAGCTCACAGTCTAGT | 21 |
| cgr-miR-151-3p_1ss21GT | CTAGACTGAGGCTCCTTGAGT | 21 |
| cgr-miR-151-5p | TCGAGGAGCTCACAGTCTAGT | 21 |
| cgr-miR-151-3p_1ss21GT | CTAGACTGAGGCTCCTTGAGT | 21 |
| hsa-miR-152-5p_2ss2GA19CT | AAGTTCTGTGATACACTCTGACT | 23 |
| fru-miR-152_R-1 | TCAGTGCATAACAGAACTTTG | 21 |
| dre-miR-153a-5p_L-1 | TCATTTTTGTGATGTTGCAGCT | 22 |
| cfa-miR-153_R+2 | TTGCATAGTCACAAAAGTGATC | 22 |
| aca-miR-155-5p_R+1 | TTAATGCTAATCGTGATAGGGGT | 23 |
| pma-miR-181a-5p | AACATTCAACGCTGTCGGTGAGT | 23 |
| pma-miR-181a-3p | ACCATCGACCGTTGACTGTACC | 22 |
| pma-miR-181a-5p | AACATTCAACGCTGTCGGTGAGT | 23 |
| pma-miR-181a-3p | ACCATCGACCGTTGACTGTACC | 22 |
| hhi-miR-181b_R-1 | AACATTCATTGCTGTCGGTGGGT | 23 |
| dre-miR-181b-3p_1ss21AG | CTCACTGATCAATGAATGCAGA | 22 |
| gga-miR-181b-5p_R+1 | AACATTCATTGCTGTCGGTGGGT | 23 |
| gga-miR-181b-2-3p_L+1R-2 | CTCACTGATCAATGAATGCA | 20 |
| ccr-miR-182-5p_R-1 | TTTGGCAATGGTAGAACTCACA | 22 |
| ccr-miR-182-3p_L-1R-1 | TGGTTCTAGACTTGCCAACT | 20 |
| ccr-miR-182-5p_R-1 | TTTGGCAATGGTAGAACTCACA | 22 |
| ccr-miR-182-3p_L-1R-1 | TGGTTCTAGACTTGCCAACT | 20 |
| chi-miR-183 | TATGGCACTGGTAGAATTCACT | 22 |
| ola-miR-184-5p_L-1R+2 | CCTTATCACTTTTCCAGCCCAGC | 23 |
| ccr-miR-184_R-2 | TGGACGGAGAACTGATAAGG | 20 |
| bta-miR-185_R-1 | TGGAGAGAAAGGCAGTTCCTG | 21 |
| cgr-miR-185-5p_R-1 | TGGAGAGAAAGGCAGTTCCTG | 21 |
| bta-miR-186_R-1 | CAAAGAATTCTCCTTTTGGGC | 21 |
| gga-miR-187-5p_R+1_1ss5AG | GGCTGCAACACAGGACATGGGAA | 23 |
| ccr-miR-187 | TCGTGTCTTGTGTTGCAGCCAGT | 23 |
| ola-miR-187_L+3R+4 | ATCGGCTGCAACACAGGACATGGGT | 25 |
| tni-miR-190_R+1 | TGATATGTTTGATATATTAGGTT | 23 |
| ssa-miR-190a-3p | ACTATATATCAAACATATTCCT | 22 |
| chi-miR-191-5p | CAACGGAATCCCAAAAGCAGCT | 22 |
| chi-miR-191-5p | CAACGGAATCCCAAAAGCAGCT | 22 |
| ccr-miR-192 | ATGACCTATGAATTGACAGCC | 21 |
| ssa-miR-192a-3p_1ss11AC | CCTGTCAGTTCTGTAGGCCACT | 22 |
| ola-miR-192-5p_L+1R-1 | ATGACCTATGAATTGACAGCC | 21 |
| ola-miR-192-3p_R-1 | CCTGTCAGTTCTGTAGGCCACT | 22 |
| dre-miR-193a-5p | TGGGTCTTTGCGGGCAAGGTGA | 22 |
| dre-miR-193a-3p | AACTGGCCTACAAAGTCCCAGT | 22 |
| dre-miR-193a-5p | TGGGTCTTTGCGGGCAAGGTGA | 22 |
| dre-miR-193a-3p | AACTGGCCTACAAAGTCCCAGT | 22 |
| cgr-miR-193b-3p_R-2_1ss9CT | AACTGGCCTACAAAGTCCCG | 20 |
| ola-miR-194-5p_R+3 | TGTAACAGCAACTCCATGTGGA | 22 |
| ola-miR-194-3p | CCAGTGGAGGTGCTGTTACCTG | 22 |
| aca-miR-194-5p | TGTAACAGCAACTCCATGTGGA | 22 |
| bta-miR-195_R-2_1ss10AG | TAGCAGCACGGAAATATTGG | 20 |
| chi-miR-195-3p_L-1R-1_1ss12TA | CAATATTGGCAGTGCTGCT | 19 |
| age-miR-196 | TAGGTAGTTTCATGTTGTTGGG | 22 |
| bta-miR-197 | TTCACCACCTTCTCCACCCAGC | 22 |
| pma-miR-199a-5p_R-1 | CCCAGTGTTCAGACTACCTGTT | 22 |
| pma-miR-199b-3p_L+1R-1 | ACAGTAGTCTGCACATTGGTT | 21 |
| pma-miR-199a-5p_R-1 | CCCAGTGTTCAGACTACCTGTT | 22 |
| pma-miR-199b-3p_L+1R-1 | ACAGTAGTCTGCACATTGGTT | 21 |
| gga-miR-199b_1ss19GT | CAGTAGTCTGCACATTTGTT | 20 |
| dre-miR-200a-5p_1ss10GT | CATCTTACCTGACAGTGCTGGA | 22 |
| ccr-miR-200a_R+2 | TAACACTGTCTGGTAACGATGTT | 23 |
| aca-miR-200b-3p_R+1 | TAATACTGCCTGGTAATGATGAT | 23 |
| aca-miR-200b-3p_R+1 | TAATACTGCCTGGTAATGATGAT | 23 |
| ssc-miR-202-5p_L+2_1ss18TC | CCTTCCTATGCATATACCTCTTT | 23 |
| ssc-miR-202-5p_L+2_1ss18TC | CCTTCCTATGCATATACCTCTTT | 23 |
| ssa-miR-202-3p_R-1_1ss22GA | TTCCTATGCATATACCGCTTTA | 22 |
| dre-miR-203a-5p_R-2_1ss10TC | AGTGGTTCTCAACAGTTCAACA | 22 |
| fru-miR-203 | GTGAAATGTTTAGGACCACTTG | 22 |
| aca-miR-203-5p_R-1_1ss10TC | AGTGGTTCTCAACAGTTCAACA | 22 |
| aca-miR-203-3p | GTGAAATGTTTAGGACCACTTG | 22 |
| oan-miR-204-5p | TTCCCTTTGTCATCCTATGCCT | 22 |
| oan-miR-204-3p_L+1R+1_1ss21AC | GCAGGGACAGCAAAGGGATGCC | 22 |
| aca-miR-204a-5p | TTCCCTTTGTCATCCTATGCCT | 22 |
| ccr-miR-205_R-1 | TCCTTCATTCCACCGGAGTCT | 21 |
| dre-miR-205-3p_R-1_1ss20GA | GATTTCAGTGGTGTGAAGTAT | 21 |
| dre-miR-205-5p_R-1 | TCCTTCATTCCACCGGAGTCT | 21 |
| dre-miR-205-3p_R-1_1ss20GA | GATTTCAGTGGTGTGAAGTAT | 21 |
| dre-miR-206-5p_R-1_1ss17GC | ACATGCTTCCTTATATCCCCAT | 22 |
| ola-miR-206_R+1 | TGGAATGTAAGGAAGTGTGTGG | 22 |
| cgr-miR-210-3p_R+1_1ss21GT | CTGTGCGTGTGACAGCGGCTTAT | 23 |
| ipu-miR-212 | ACCTTGGCTCTAGACTGCTTACT | 23 |
| ssa-miR-212a-3p | TAACAGTCTACAGTCATGGCT | 21 |
| ssa-miR-212b-5p | ACCTTGGCTCTAGACTGCTTACT | 23 |
| xtr-miR-212 | TAACAGTCTACAGTCATGGCT | 21 |
| hsa-miR-212-5p | ACCTTGGCTCTAGACTGCTTACT | 23 |
| aca-miR-214-5p | TGCCTGTCTACACTTGCTGTGC | 22 |
| aca-miR-214-3p | ACAGCAGGCACAGACAGGCAGT | 22 |
| tni-miR-216a | AAATCTCAGCTGGCAACTGTGA | 22 |
| aca-miR-216b-5p_1ss1AT | TAATCTCTGCAGGCAAATGTGA | 22 |
| fru-miR-217_R+1 | TACTGCATCAGGAACTGATTGGC | 23 |
| fru-miR-218a | TTGTGCTTGATCTAACCATGTG | 22 |
| ssa-miR-218-3p_L-1_1ss18AG | CATGGTTCCGTCAAGCGCCAGG | 22 |
| aca-miR-219-5p_R-1 | TGATTGTCCAAACGCAATTCTT | 22 |
| aca-miR-219-1-3p_L-1R+1_1ss12CA | AGAATTGTGTATGGACATCTGT | 22 |
| aca-miR-219-5p_R-1 | TGATTGTCCAAACGCAATTCTT | 22 |
| aca-miR-219-1-3p_L-1R+1_1ss12CA | AGAATTGTGTATGGACATCTGT | 22 |
| hsa-miR-219a-5p | TGATTGTCCAAACGCAATTCT | 21 |
| dre-miR-219-5p | TGATTGTCCAAACGCAATTCTT | 22 |
| dre-miR-221-5p_R-4 | ACCTGGCATACAATGTAGATTT | 22 |
| bta-miR-221 | AGCTACATTGTCTGCTGGGTTT | 22 |
| cgr-miR-221-5p_R-4 | ACCTGGCATACAATGTAGATTT | 22 |
| cgr-miR-221-3p_R-1 | AGCTACATTGTCTGCTGGGTTT | 22 |
| dre-miR-222a-5p_R-1_1ss11TG | TGCTCAGTAGGCAGTGTAGATC | 22 |
| fru-miR-222_R-1 | AGCTACATCTGGCTACTGGGTCT | 23 |
| ssa-miR-222a-5p_R-2 | TGCTCAGTAGGCAGTGTAGATC | 22 |
| ssa-miR-222a-3p_R-1 | AGCTACATCTGGCTACTGGGTCT | 23 |
| gga-miR-222b-5p_R-7 | TGCTCAGTAGTCAGTGTAG | 19 |
| gga-miR-222b-3p_R-1 | AGCTACATCTGATTACTGGGTCA | 23 |
| aca-miR-222a-5p_L+1R-4 | TGCTCAGTAGTCAGTGTAG | 19 |
| hsa-miR-223-5p_L-2R+3 | TGTATTTGACAAGCTGAGTTGGA | 23 |
| hsa-miR-223-3p | TGTCAGTTTGTCAAATACCCCA | 22 |
| oha-miR-223-5p | GTGTATTTGACAAGCTGAGTT | 21 |
| oha-miR-223-3p_R-3_1ss18TC | TGTCAGTTTGTCAAATACA | 19 |
| dre-miR-223_R+1 | TGTCAGTTTGTCAAATACCCCA | 22 |
| ipu-miR-223_L-1R+4 | AGTATTTGACAGACTGTGGTTGA | 23 |
| dre-miR-301c-5p_R+1_2ss4CT9GA | GCTTTGACAATGTTGCACTACT | 22 |
| fru-miR-301_R+1 | CAGTGCAATAGTATTGTCATAGC | 23 |
| ssa-miR-301a-3p | CAGTGCAATAGTATTGTCATAGC | 23 |
| bta-miR-320a | AAAAGCTGGGTTGAGAGGGCGA | 22 |
| cgr-miR-322-5p | CAGCAGCAATTCATGTTTTGG | 21 |
| cgr-miR-322-3p_L+1 | AAAACATGAAGCGCTGCAACAC | 22 |
| bta-miR-326_R+1 | CCTCTGGGCCCTTCCTCCAGA | 21 |
| bta-miR-328 | CTGGCCCTCTCTGCCCTTCCGT | 22 |
| aja-miR-331_R+1 | GCCCCTGGGCCTATCCTAGAAC | 22 |
| ola-miR-338-5p_1ss6CT | AACAATATCCTGGTGCTGCCTGAGT | 25 |
| ola-miR-338-3p_R+2 | TCCAGCATCAGTGATTTTGTTGC | 23 |
| ola-miR-338-3p_R+2 | TCCAGCATCAGTGATTTTGTTGC | 23 |
| bta-miR-339b_R+3 | TCCCTGTCCTCCAGGAGCTCACA | 23 |
| hsa-miR-339-5p_1ss23GA | TCCCTGTCCTCCAGGAGCTCACA | 23 |
| cfa-miR-340 | TTATAAAGCAATGAGACTGATT | 22 |
| hsa-miR-340-5p | TTATAAAGCAATGAGACTGATT | 22 |
| dre-miR-363-3p | AATTGCACGGTATCCATCTGTA | 22 |
| hsa-miR-363-3p | AATTGCACGGTATCCATCTGTA | 22 |
| ssa-miR-365-5p_R+3 | AGGGACTTTTAGGGGCAGCTGTG | 23 |
| ccr-miR-365 | TAATGCCCCTAAAAATCCTTAT | 22 |
| aca-miR-365-5p_1ss10CT | AGGGACTTTTAGGGGCAGCTGTG | 23 |
| aca-miR-365-3p | TAATGCCCCTAAAAATCCTTAT | 22 |
| bta-miR-374a_R-1 | TTATAATACAACCTGATAAGT | 21 |
| hsa-miR-374a-5p_R-1 | TTATAATACAACCTGATAAGT | 21 |
| tni-miR-375 | TTTGTTCGTTCGGCTCGCGTTA | 22 |
| bta-miR-378 | ACTGGACTTGGAGTCAGAAGGC | 22 |
| hsa-miR-409-5p_R-2 | AGGTTACCCGAGCAACTTTGC | 21 |
| bta-miR-409a_R-2 | AGGTTACCCGAGCAACTTTGC | 21 |
| efu-miR-423_R-3 | TGAGGGGCAGAGAGCGAGACT | 21 |
| bta-miR-423-5p_R-2 | TGAGGGGCAGAGAGCGAGACT | 21 |
| bta-miR-425-5p_L+1R+1 | AATGACACGATCACTCCCGTTGAA | 24 |
| bta-miR-425-5p_L+1R+1 | AATGACACGATCACTCCCGTTGAA | 24 |
| gga-miR-429-5p_L-1_2ss12GA16AG | GTCTTACCAGACAAGGTTAGA | 21 |
| xtr-miR-429 | TAATACTGTCTGGTAATGCCGT | 22 |
| hhi-miR-430b | TAAGTGCTTCTCTTTGGGGTTG | 22 |
| dre-miR-430a-3p_R-4_1ss9AT | TAAGTGCTTTTTGTTGGG | 18 |
| bta-miR-450a | TTTTGCGATGTGTTCCTAATAT | 22 |
| xtr-miR-451_R+1 | AAACCGTTACCATTACTGAGTTT | 23 |
| aca-miR-451-3p_L+2 | TTTAGTAATGGTAAGGGTTCT | 21 |
| pma-miR-451_R-4_1ss16TC | AAACCGTTACCATTACTGTA | 20 |
| ssa-miR-454-5p_1ss16CT | ACCCTATCAATATTGTCTCTGC | 22 |
| ccr-miR-454b_R+2 | TAGTGCAATATTGCTTATAGGGT | 23 |
| tni-miR-455 | TATGTGCCCTTGGACTACATCG | 22 |
| dre-miR-455-3p_L-2R+1 | GCAGTCCATGGGCATATACACT | 22 |
| dre-miR-455-5p | TATGTGCCCTTGGACTACATCG | 22 |
| dre-miR-455-3p_L-2R+1 | GCAGTCCATGGGCATATACACT | 22 |
| aca-miR-456_R+1 | CAGGCTGGTTAGATGGTTGTCT | 22 |
| ccr-miR-457b_L+1R-1 | TAGCAGCACATAAATACTGGA | 21 |
| dre-miR-457b-3p | TCCAGTATTGCTGTTCTGCTGT | 22 |
| ccr-miR-457a_L+1R+1 | TAGCAGCACATCAATATTGGCA | 22 |
| dre-miR-458-5p_R-1_2ss11AT21AT | AGCGCCATTTTCAGAGCTATT | 21 |
| fru-miR-458 | ATAGCTCTTTAAATGGTACTGC | 22 |
| ccr-miR-459-5p_L+2R-1 | TCAGTAACAAGGATTCATCCTGT | 23 |
| ccr-miR-460-5p | CCTGCATTGTACACACTGTGCG | 22 |
| ccr-miR-460-3p | CACAGCGCATACAATGTGGATG | 22 |
| ccr-miR-460-5p | CCTGCATTGTACACACTGTGCG | 22 |
| ccr-miR-460-3p | CACAGCGCATACAATGTGGATG | 22 |
| dre-miR-462_1ss12AT | TAACGGAACCCTTAATGCAGCT | 22 |
| mmu-miR-466i-5p_L-2 | TGTGTGTGTGTGTGTGTG | 18 |
| mmu-miR-466i-5p_R-1 | TGTGTGTGTGTGTGTGTGT | 19 |
| bta-miR-484_R+1 | TCAGGCTCAGTCCCCTCCCGATT | 23 |
| cgr-miR-486-5p | TCCTGTACTGAGCTGCCCCGAG | 22 |
| cgr-miR-486-5p | TCCTGTACTGAGCTGCCCCGAG | 22 |
| aca-miR-489-5p | TGGTCGTATGTATGACGTCATT | 22 |
| aca-miR-489-3p_L-1R+1 | TGACATCATATGTACGGCTGCT | 22 |
| bta-miR-497_R+1 | CAGCAGCACACTGTGGTTTGTAT | 23 |
| aca-miR-499-5p_R-1 | TTAAGACTTGCAGTGATGTTT | 21 |
| aca-miR-499-3p | AACATCACTTTAAGTCTGTGCT | 22 |
| cgr-miR-499-5p_R-1 | TTAAGACTTGCAGTGATGTTT | 21 |
| cgr-miR-499-3p | GAACATCACAGCAAGTCTGTGC | 22 |
| dre-miR-499-5p_R-1 | TTAAGACTTGCAGTGATGTTT | 21 |
| ola-miR-499_R+1 | AACATCACTTTAAGTCTGTGCT | 22 |
| bta-miR-532 | CATGCCTTGAGTGTAGGACCGT | 22 |
| hsa-miR-532-3p_R-1 | CCTCCCACACCCAAGGCTTGC | 21 |
| chi-miR-532-5p | CATGCCTTGAGTGTAGGACCGT | 22 |
| chi-miR-532-3p | CCTCCCACACCCAAGGCTTGC | 21 |
| mmu-miR-541-5p_R-3 | AAGGGATTCTGATGTTGGTCAC | 22 |
| cfa-miR-542 | TGTGACAGATTGATAACTGAAA | 22 |
| hsa-miR-542-3p | TGTGACAGATTGATAACTGAAA | 22 |
| ccr-miR-551_1ss14GA | GCGACCCATCCTTAGTTTCTG | 21 |
| ggo-miR-574 | CACGCTCATGCACACACCCACA | 22 |
| ggo-miR-584 | TTATGGTTTGCCTGGGACTGA | 21 |
| hsa-miR-584-3p_L-2R-2_1ss6TA | AGTACCAGGCCAACCAGG | 18 |
| hsa-miR-606_L-1R-1_1ss12AT | AACTACTGAATATCAAAGA | 19 |
| hsa-miR-629-5p_1ss10GC | TGGGTTTACCTTGGGAGAACT | 21 |
| cfa-miR-660 | TACCCATTGCATATCGGAGTTG | 22 |
| hsa-miR-708-3p_R+1 | CAACTAGACTGTGAGCTTCTAGT | 23 |
| ssa-miR-722-5p_L-1R+1 | TTTGAAACGTTTTAGCCAAAA | 21 |
| ccr-miR-722_L-2R+3 | TTTTGCAGAAACGTTTCAGATT | 22 |
| ssa-miR-723-3p | AGACATCAGATAAATCTGTGCT | 22 |
| ccr-miR-724 | TTAAAGGGAATTTGCGACTGTT | 22 |
| ssa-miR-724-3p | CAGCCACACCTTCCTTTTAAGA | 22 |
| ssa-miR-725-3p_1ss20AC | TTCAGTCATTGTTTCTGGTCGT | 22 |
| aca-miR-726_R+1 | TTCACTACTAGCAGAACTCGGT | 22 |
| ccr-miR-727-5p_1ss11AG | TCAGTCTTCAGTTCCTCCCAGC | 22 |
| ccr-miR-727-3p_L-1R+1_1ss22AT | TTGAGGCGAGTTGAAGACTTTA | 22 |
| hhi-miR-728_R+1 | ATACTAAGTACACTACGTTTAT | 22 |
| dre-miR-730_R-2 | TCCTCATTGTGCATGCTGTGT | 21 |
| gga-miR-460b-3p_R-2_2ss9AC21CG | CACAGCGCCTGCAATGTGGAG | 21 |
| ssa-miR-731-5p | AATGACACGTTTTCTCCCGGATT | 23 |
| ssa-miR-731-3p_L-1R-1_1ss21CA | ACCGGGAATTTCGTGTCAGA | 20 |
| ssa-miR-734-5p | GAACTATTCTGCAACATTTGTT | 22 |
| ssa-miR-734-3p_1ss22TC | TAAATGCTGCAGAATTGTGCTC | 22 |
| ccr-miR-734 | TAAATGCTGCAGAATCGTACCG | 22 |
| dre-miR-735-5p_R+8_1ss18TA | GGCTGGTCCGAAGGCGGAGGGTTAGT | 26 |
| ssa-miR-736-5p_L-3_1ss8CT | CTTTTTGTTTGTATTATGT | 19 |
| ssa-miR-736-3p | GTAAGACGAACAAAAAGTTTGT | 22 |
| dre-miR-737-5p_L-2R+1 | TTTTTTAGGTTTTGATTTTT | 20 |
| dre-miR-737-3p | AATCAAAACCTAAAGAAAATA | 21 |
| ccr-miR-738_R+3 | GCTACGGCCCGCGTCGGGACCT | 22 |
| eca-miR-885-5p | TCCATTACACTACCCTGCCTCT | 22 |
| bta-miR-885 | TCCATTACACTACCCTGCCTCT | 22 |
| mmu-miR-1187_R-2_1ss2AT | TTTGTGTGTGTGTATGTGTGT | 21 |
| cfa-miR-1249 | ACGCCCTTCCCCCCCTTCTTCA | 22 |
| cgr-miR-1260_L+3 | CAAATCCCACCGCTGCCACCA | 21 |
| efu-miR-1271_1ss22AT | CTTGGCACCTGGTAAGCACTCT | 22 |
| hsa-miR-1277-5p_L-4R-1_1ss20CT | ATATATATATATGTATGTA | 19 |
| hsa-miR-1277-5p_L-4R-1_1ss20CT | ATATATATATATGTATGTA | 19 |
| chi-miR-1306-5p_R+1 | CCACCTCCCCTGCAAACGTCCA | 22 |
| chi-miR-1306-5p_R+1 | CCACCTCCCCTGCAAACGTCCA | 22 |
| hsa-miR-1307-5p | TCGACCGGACCTCGACCGGCT | 21 |
| bta-miR-1307_R+1 | ACTCGGCGTGGCGTCGGTCGTGG | 23 |
| chi-miR-1307-5p_R+4 | TCGACCGGACCTCGACCGGCT | 21 |
| chi-miR-1307-3p | ACTCGGCGTGGCGTCGGTCGTGG | 23 |
| bta-miR-1343-3p_L-3R+2 | CTGGGGCCCGCACTCTCGC | 19 |
| oan-miR-1357_L+1_1ss19CT | GATTATGAGATCTGAGGGTC | 20 |
| oan-miR-1386_1ss1CA | ATCCTGGCTGGCTCGCCA | 18 |
| ola-miR-1388-5p | AGGACTGTCCAACCTGAGAATG | 22 |
| ola-miR-1388-3p_R+4 | ATCTCAGGTTCGTCAGCCCATG | 22 |
| ola-miR-1388-5p | AGGACTGTCCAACCTGAGAATG | 22 |
| ola-miR-1388-3p_R+4 | ATCTCAGGTTCGTCAGCCCATG | 22 |
| oan-miR-1404_R-4_1ss2TA | TAGGGGAAAAAGAAGAGT | 18 |
| gga-miR-1451-5p | TCGCACAGGAGCAAGTTACCGC | 22 |
| bta-miR-1468 | CTCCGTTTGCCTGTTTTGCTGA | 22 |
| gga-miR-1555-3p_L-2R+1_1ss3GT | TGGTTATTGGTTTTGTGTGAC | 21 |
| gga-miR-1638_L-1R-1_1ss8GT | TAGTTTTTGTTGTTTGTT | 18 |
| gga-miR-1692_R-2_1ss1TA | AGTAGCTCAGTTGGTAGA | 18 |
| aca-miR-1788-5p_R-1_2ss11CA21CT | GGCTTGTTTTAAGTTGCCTGT | 21 |
| aca-miR-1788-3p_R+1 | CAGGCAGCTAAAGCAAGTCTGT | 22 |
| aca-miR-1788-5p_R-1_2ss11CA21CT | GGCTTGTTTTAAGTTGCCTGT | 21 |
| aca-miR-1788-3p_R+1 | CAGGCAGCTAAAGCAAGTCTGT | 22 |
| gga-miR-1797_L-2R-2_1ss8AT | TTGGATCTGAGCAGGAAC | 18 |
| efu-miR-1842_R-3 | TTGGCTCTGCGAGGTCGGCTC | 21 |
| mmu-miR-1843b-5p_L+1 | TATGGAGGTCTCTGTCTGACTT | 22 |
| mmu-miR-1843b-5p_L+1 | TATGGAGGTCTCTGTCTGACTT | 22 |
| mmu-miR-1957a_R-1_1ss15AT | CAGTGGTAGAGCATTTGA | 18 |
| lva-miR-2002-3p_L-2R-3_1ss5TC | AACACATCTGCTGGTTTT | 18 |
| dre-miR-2184_R+1 | AACAGTAAGAGTTTATGTGCTG | 22 |
| dre-miR-2187-5p_R-1 | TTAATTAGTATAGCCTGTTTT | 21 |
| dre-miR-2187-3p_L+1R-1_1ss21AG | TTTACAGGCTATGCTAATCTGT | 22 |
| ssa-miR-2188-5p | AAGGTCCAACCTCACATGTCCT | 22 |
| ssa-miR-2188-3p_1ss14AG | GCTGTGTGAGGTCGGACCTATC | 22 |
| bta-miR-2410_L+1R-5 | ACTGAGCTGAGGAGGACC | 18 |
| bta-miR-2416_L-1R-4_1ss9CT | GCAGTGCTCACTGTGGAA | 18 |
| gga-miR-2954_R+2 | CATCCCCATTCCACTCCTAGCAGT | 24 |
| tgu-miR-2970-5p_1ss21GT | GACAGTCAGCAGTTGGTCTGT | 21 |
| hsa-miR-3120-5p_L-1R+2 | CTGTCTGTGCCTGCTGTACAGG | 22 |
| hsa-miR-3120-3p_L+2R-1_1ss18CT | TGCACAGCAAGTGTAGATAGGC | 22 |
| hsa-miR-3120-5p_L-1R+2 | CTGTCTGTGCCTGCTGTACAGG | 22 |
| hsa-miR-3120-3p_L+2R-1_1ss18CT | TGCACAGCAAGTGTAGATAGGC | 22 |
| hsa-miR-3149_L-2_1ss9GT | TGTATGTATATGTGTGTGTAT | 21 |
| hsa-miR-3150b-5p_L-4_1ss9AC | CTCGCGGATCTCCCCAGC | 18 |
| hsa-miR-3168_R+1_1ss14AC | GAGTTCTACAGTCCGACA | 18 |
| gga-miR-3529_1ss11TG | AGGCAGACTGGGACTTGTTGT | 21 |
| rno-miR-3574_L-1R+1_1ss2CT | TAGCCGCTGTCACACGCACAGT | 22 |
| rno-miR-3588_L-1R+2 | CACAAGTTAGGGTCTCAGGGACT | 23 |
| hsa-miR-3591-5p_L+1R-1 | ATTTAGTGTGATAATGGCGTTTG | 23 |
| bta-miR-3604 | TAACCAATGTGCAGACTACTGT | 22 |
| hsa-miR-3615_R-1 | TCTCTCGGCTCCTCGCGGCT | 20 |
| eca-miR-3959_R-1 | TGTATGTCAACTGATCCACAGT | 22 |
| cin-miR-4185-3p_L+1R-2_1ss20GT | TTGTATTCATACTGTCTGATC | 21 |
| bta-miR-4286_L+2R+1 | GAACCCCACTCCTGGTACCA | 20 |
| ssc-miR-4332_L+3R+3_1ss23CA | GTCCACGGCCGCCGCCGGGCGCATTT | 26 |
| hsa-miR-4443_R+1 | TTGGAGGCGTGGGTTTTT | 18 |
| hsa-miR-4448_1ss6CG | GGCTCGTTGGTCTAGGGGTA | 20 |
| hsa-miR-4454_L-2_1ss8AG | ATCCGGGTCACGGCACCA | 18 |
| hsa-miR-4508_L+1_1ss17CT | AGCGGGGCTGGGCGCGTG | 18 |
| hsa-miR-4792_L+1R+1_1ss10GT | CCGGTGAGCTCTCGCTGGCC | 20 |
| bfl-miR-4905_L-1R-2_1ss16TG | ATGTGGAAGATATTGTAGA | 19 |
| mmu-miR-5100_R-2 | TCGAATCCCAGCGGTGCCT | 19 |
| hsa-miR-6125_R-2_1ss13GC | GCGGAAGGCGGACCGGCG | 18 |
| mml-miR-6134_R+3_1ss18GT | TGAGGTAGTAGGATGTATAGTT | 22 |
| mmu-miR-6239_R-2_1ss6TG | TAGCGGTGGATCACTCGG | 18 |
| mmu-miR-6240_L+1R-3 | TCCAAAGCATCGCGAAGGCCCACG | 24 |
| mmu-miR-6402_L-3R-1_1ss22GC | CAGTTTTCCCAGGAACCCC | 19 |
| mmu-miR-6412_R-2_1ss15AT | TCGAAACCATCCTCTGCTAC | 20 |
| gga-miR-6516-3p_R-3_1ss11AG | CATGTATGATGCTGCACACA | 20 |
| mmu-miR-6937-5p_R-6_1ss5TG | TAGCGGTAAGGGCTGGGT | 18 |
| mmu-miR-6961-3p_L-1R-3_1ss6TG | CCTCGTTCTTCCTGGCTC | 18 |
| mmu-miR-7042-5p_L-2_1ss10CA | GAGACAGAAGAAGGGCCAC | 19 |
| ssa-miR-7132b-5p | GACTTGGTCAAAGCTCCTCAGC | 22 |
| ssa-miR-7132b-3p | TGAGGCGTTTAGAACAAGTTCA | 22 |
| ccr-miR-7132_R+1_1ss10TA | TGAGGAGTTAAGAGCAAGTAAA | 22 |
| ccr-miR-7133 | TAGTTTGATTCACAGCACAAGA | 22 |
| ssc-miR-7134-5p_R+1 | ATGTCCGCGGGTTCCCTATCCC | 22 |
| dre-miR-7147 | TGTACCATGCTGGTAGCCAGT | 21 |
| dre-miR-7149-5p_R-1_1ss11AG | TGTGAATCCTGCACTGGAAG | 20 |
| mml-miR-7192-5p_R-5_1ss4CA | TCAATCAGAATTAAAAAA | 18 |
| mml-miR-7208-3p_R-3 | AGCAAAGCAAAGCTCAGT | 18 |
| ipu-miR-7550_L-1R+4 | TCCGGCTCGAAGGACCATTTT | 21 |
| ssa-miR-7552a-5p | TTACAATTAAAGGATATTTCTT | 22 |
| ssa-miR-7552a-5p | TTACAATTAAAGGATATTTCTT | 22 |
| hsa-miR-7641_L+2_1ss3TC | GTCTGATCTCGGAAGCTAAGC | 21 |
| hsa-miR-7977_1ss6AG | TTCCCGGCCAACGCACCA | 18 |
| mmu-miR-8094_L-2R+5 | CTGAAGGACAACGAGAAGAACCGC | 24 |
| mmu-miR-8109_L-1R-1_1ss18CG | CGCCGCGTGCCGGCCGGGG | 19 |
| ssa-miR-8159-5p_R-1 | TCAGTAACTGGAATCTGTCCCTG | 23 |
| ssa-miR-8160-5p_1ss21CT | AGAATAATGCCAGCAGTCGGTC | 22 |
| ssa-miR-8160-3p_1ss19GA | CCAGCACTGGTGTTATTGAGA | 21 |
| ssa-miR-8160-5p_1ss21CT | AGAATAATGCCAGCAGTCGGTC | 22 |
| ssa-miR-8160-3p_1ss19GA | CCAGCACTGGTGTTATTGAGA | 21 |
| eca-miR-8909_R-4_1ss19CA | CCTGAAATTCTGATGCACA | 19 |
| eca-miR-9083_L-7_1ss13TA | CGGTGAGCTATCGTCGGC | 18 |
| eca-miR-9112_L-4R-2_1ss19AC | CATGATGCTCTCTGCGCC | 18 |
| efu-miR-9226_L-4_1ss22GA | GTCCCTGTTCGGGCGCCA | 18 |
| dre-let-7d-5p | TGAGGTAGTTGGTTGTATGGTT | 22 |
| dre-let-7c-3p | CTGTACAACCTTCTAGCTTTCC | 22 |
| dre-let-7d-5p | TGAGGTAGTTGGTTGTATGGTT | 22 |
| dre-let-7c-3p | CTGTACAACCTTCTAGCTTTCC | 22 |
| ola-let-7a_R+1 | TGAGGTAGTAGGTTGTATAGTTT | 23 |
| ola-let-7a-3p_R+1 | CTATACAACTTACTGTCTTTCC | 22 |
| ssa-let-7h-5p | TGAGGTAGTAAGTTGTGTTGTT | 22 |
| ssa-let-7h-3p | CTATACAACTTACTGCCTTCCT | 22 |
| tni-let-7h | TGAGGTAGTAAGTTGTGTTGTT | 22 |
| ssa-let-7h-3p | CTATACAACTTACTGCCTTCCT | 22 |
| oha-let-7i-5p_R+2_1 | TGAGGTAGTAGTTTGTGCTGTTAT | 24 |
| oha-let-7i-3p_1ss23AT | CTGCGCAAGCTACTGCCTTGCTT | 23 |
| oha-let-7i-5p_R+2_2 | TGAGGTAGTAGTTTGTGCTGTTCA | 24 |
| oha-let-7i-3p_1ss23AT | CTGCGCAAGCTACTGCCTTGCTT | 23 |
| hsa-let-7d-5p_1ss16CT | AGAGGTAGTAGGTTGTATAGTT | 22 |
| cfa-let-7d_R-3 | CTATACGACCTGCTGCCTTTCT | 22 |
| hhi-let-7j | TGAGGTAGTTGTTTGTACAGTT | 22 |
| cin-let-7e_R-4 | TGAGGTAGTTGGTTGTAT | 18 |
| mmu-let-7k_R+2_1ss9GT | TGAGGTAGTAGGTTGTGTGCA | 21 |
| pmi-let-7-5p_1ss10CA | TGAGGTAGTAGGTTGTAAAGA | 21 |
| PC-5p-110922_10 | TCAGTTGATTTGCTTGTTCGCATGC | 25 |
| PC-3p-356229_3 | TCAGACAGAAACTGTTCCTGCT | 22 |
| PC-5p-110922_10 | TCAGTTGATTTGCTTGTTCGCATGC | 25 |
| PC-3p-356229_3 | TCAGACAGAAACTGTTCCTGCT | 22 |
| PC-5p-551911_2 | TGCACCACCGCGCCATCCC | 19 |
| PC-3p-2185057_1 | TGGGGAGGGGGTGGGGAGTTTCAGT | 25 |
| PC-5p-693012_2 | GAGTCTGGACCTGGTCTAAGAC | 22 |
| PC-3p-204887_5 | TAGTCTCGGACATGTCTTGGT | 21 |
| PC-5p-613031_2 | GTGTGTTGCTGTTACTTCACTGTC | 24 |
| PC-3p-731770_2 | TTATTGGATTAAAAAGTT | 18 |
| PC-5p-741120_2 | TAGCATGTTAAAATTTGAC | 19 |
| PC-3p-451690_2 | CTTTCAGTTAAGGCTTCTT | 19 |
| PC-5p-398351_2 | TGACAAGGTAAACAGCTAGCAGACT | 25 |
| PC-3p-4192042_1 | TAGTAACTAACTGAAATAAAAAGCCT | 26 |
| PC-5p-185959_5 | TTCTGTAAAGTGTCTATGTAGCATT | 25 |
| PC-3p-4371904_1 | CATTTTAGTTTAGAGTCGTTCTAAAA | 26 |
| PC-5p-2292265_1 | TCTGTTCTGGGCTAAAGACGGAGAGG | 26 |
| PC-3p-2293370_1 | GTTCTGGACTAAAGACGGAGAGGAGG | 26 |
| PC-5p-74911_18 | TCGTTTTGCCAGTGTGCTGGAC | 22 |
| PC-3p-68238_20 | TCGTTTTGCCAGTGTGCTGGACGAC | 25 |
| PC-5p-32222_56 | TTCTCATTGAGCTTGCCAACGCC | 23 |
| PC-3p-406098_2 | CAATGCAACGTGGCCAGGAGCTGT | 24 |
| PC-5p-426187_2 | TGATCAACTGGAGATTGTCACTGAGT | 26 |
| PC-3p-1184793_1 | AGGATTGCATGTGGAGTGGTTTCTCT | 26 |
| PC-5p-164700_6 | TGCGAAATAATGACCAGGCAAGG | 23 |
| PC-3p-606349_2 | TCAATTCTAAGGCCAGAAGGGTT | 23 |
| PC-5p-225130_4 | TATGATGCTCATGAATTTGGTCT | 23 |
| PC-3p-1629271_1 | CTGCAGGCGTCGCAAGGACAGCAGCA | 26 |
| PC-5p-99077_12 | GATAACGATATTTCTTGCGATAT | 23 |
| PC-3p-456020_2 | TGTAATGTAATGACCATACTAAA | 23 |
| PC-5p-44099_38 | TCCAGTCGGAGGTCCGCTCACTAC | 24 |
| PC-3p-188720_5 | TGCTCTTCCGGTTCGGAGACCAGCC | 25 |
| PC-5p-192777_5 | ATAGCTCGGGACTAGTATTACC | 22 |
| PC-3p-685784_2 | TGTGATTTGTAATAATTGCAGAGGT | 25 |
| PC-5p-262562_4 | GAACATGATGTAAAGCGCTT | 20 |
| PC-3p-637806_2 | CTATATAAATGAACACCATTTA | 22 |
| PC-5p-58920_26 | AGTATCATGTCAACTATATTGT | 22 |
| PC-3p-61296_24 | AATATAGTTTACCTGATGCTCT | 22 |
| PC-5p-141830_7 | TGTTTCTGATATTCTTGAGGCTCC | 24 |
| PC-3p-4195415_1 | TGGCTGCCAACGGTATTGATGTGCCC | 26 |
| PC-5p-543704_2 | TAGTTTAGCTTTAGCTGCTTCAGC | 24 |
| PC-3p-235402_4 | TAGTTTAGCTTTAGCTGCTTCAGCC | 25 |
| PC-5p-129186_8 | GGTTGGACTTACTGGTGTCG | 20 |
| PC-5p-129186_8 | GGTTGGACTTACTGGTGTCG | 20 |
| PC-5p-732345_2 | TTGCTGGAGTGGCTCCTCCTTGC | 23 |
| PC-3p-2540228_1 | TGAGAGAAGCATTTACTGCCACCT | 24 |
| PC-5p-233195_4 | TCTGACAGTGTTTGTGTTC | 19 |
| PC-3p-2152982_1 | GTTATGCTGCATGCACTTCTCATC | 24 |
| PC-5p-303141_3 | ATTCTGTGTGTCAAACCTCATGT | 23 |
| PC-3p-3848661_1 | GGCTTAAACAGAGTTGTAAATGCATG | 26 |
| PC-5p-338151_3 | GCAGTCGATCGCTTAGGCT | 19 |
| PC-3p-508325_2 | TTAAGGGAATGCCTATCTTAAGTCC | 25 |
| PC-5p-104293_11 | TGTAAGCCTGGAGGTAAAGTGCC | 23 |
| PC-3p-648082_2 | TAAGGAAGGTGAGAGGATTATGGT | 24 |
| PC-5p-107532_10 | TTTGTCATTTTATTTCGTTAGC | 22 |
| PC-3p-19920_92 | TAACGAAATAAAGTGGCAAGA | 21 |
| PC-5p-81365_16 | ATCTCTGAGGAAATGATTGGCT | 22 |
| PC-3p-85279_15 | CTCCATCAGGCCTTCAGAGTGA | 22 |
| PC-5p-186496_5 | TACTATGAGTTTGGTAACAGGGC | 23 |
| PC-3p-482853_2 | TAGGGTAGTTCATAAGATTAAGTGC | 25 |
| PC-5p-706406_2 | TGATTTCCAATAATTGAGACAGA | 23 |
| PC-3p-3253896_1 | ATCTACATTAATGAAAAGAACAATGT | 26 |
| PC-5p-438959_2 | CCGTGAACGGGTCGTGAAATGTT | 23 |
| PC-3p-235844_4 | CACTTCACGACCCGTTCATGA | 21 |
| PC-5p-159181_6 | GTGTATGTTATCAAAAGAGTGACT | 24 |
| PC-3p-284980_3 | GAAGCTTGTACATAGCTAAAGGAGTA | 26 |
| PC-5p-213453_4 | TAGAATTAAGGCAGTTCTGAAGG | 23 |
| PC-3p-2197936_1 | TGGTGTTCCTAATAATTCTTTAGGTG | 26 |
| PC-5p-321148_3 | TAAGTCTAAAGATGTTTTTGATG | 23 |
| PC-3p-2019312_1 | TTTAAAGAATCTTTACTTGTGCCCAT | 26 |
| PC-5p-692015_2 | TTGCCAGCAGTTTGCCTGGCTTGGCG | 26 |
| PC-3p-185424_5 | CCAGTCAGACTGCTGGTATGTC | 22 |
| PC-5p-3126897_1 | TGTAACGTGTTCTAAGAAGAAACATG | 26 |
| PC-3p-3237047_1 | GGAGCTAGAGCTGCGGCCGACAAGT | 25 |
| PC-5p-85904_14 | TAAGAGATGTATAATAAGCAGTTTT | 25 |
| PC-3p-108149_10 | TATTGTACATGTCTTTATGAATAGCA | 26 |
| PC-5p-88108_14 | ACAGGTCTGTGGTTTTAGA | 19 |
| PC-3p-186860_5 | TTAGAATGATCTTAGTTCATAGA | 23 |
| PC-5p-41113_42 | TCCCACGTTCGAACACGTGCT | 21 |
| PC-3p-1241844_1 | TGATCGTGCTTATGCAGTGCTTCAGC | 26 |
| PC-5p-376111_3 | AGTCAGATCAAACTGAAAATGT | 22 |
| PC-5p-376111_3 | AGTCAGATCAAACTGAAAATGT | 22 |
| PC-5p-111653_10 | TTGATTGTAATGTGTTTTCAGTTAGG | 26 |
| PC-3p-136763_7 | TCAGTTATAATAAGTATTTTGTAAC | 25 |
| PC-5p-740769_2 | TCGTGAAGTGCGTGCAAGTTCG | 22 |
| PC-3p-526036_2 | CGACCCGTTCACGACCGTTCACG | 23 |
| PC-5p-373039_3 | TCGGACTGGATTAGTTTTACCTAGG | 25 |
| PC-3p-293277_3 | TTATTACCAGAGGTCCTCT | 19 |
| PC-5p-103237_11 | CCGTAGACCACACTCAGTGCGGTT | 24 |
| PC-3p-341326_3 | AGTATCTATCCACAAGAAGAG | 21 |
| PC-5p-2298385_1 | GATGATTTGAGTCTAGTGGTCTTCA | 25 |
| PC-3p-4039078_1 | ATTACACTCATGGGTAAAGGAGGC | 24 |
| PC-5p-249873_4 | AGTGCATGGATGCAGTTCTGGCTC | 24 |
| PC-3p-280340_3 | TACTGAACTATATCGACGACTGGCT | 25 |
| PC-5p-155855_6 | GTTCGATTGTGGATCACTCCCC | 22 |
| PC-3p-239614_4 | TCAACTCGTCTGTTGTTCTGGCCG | 24 |
| PC-5p-80886_16 | ATTTAAGTCACTGTAGTGT | 19 |
| PC-3p-44944_37 | ACTGCAGTGGAGGCACTTTCT | 21 |
| PC-5p-570253_2 | ACGTCAGCGGAGAGCTAAAC | 20 |
| PC-3p-4370408_1 | GAGCCCTTTACCGTTTGCTTGGAATT | 26 |
| PC-5p-282_8970 | TAGCAGCACATCATTACTGGTA | 22 |
| PC-3p-22798_81 | CCAGTGTATGATGTGCTGCTTC | 22 |
| PC-5p-300845_3 | GGTAGAATTAACAGTACCA | 19 |
| PC-3p-743865_2 | AGAACAAATATTCCGACCTGGCAGC | 25 |
| PC-5p-4261627_1 | TGTGGTGCGGAGCAAGAAGAAAGAGT | 26 |
| PC-3p-471000_2 | TGGATGTTCTGCCTCTCACACC | 22 |
| PC-5p-107914_10 | TATTTCTACAGTGTTGAACGCATG | 24 |
| PC-3p-140042_7 | GATGAGAAACATGCCTTGACGTTG | 24 |
| PC-5p-423534_2 | TGTCTCTGTAGATAGGACC | 19 |
| PC-3p-448961_2 | TATTTTTATGATGAACTTT | 19 |
| PC-5p-55262_28 | TGGATTTACTGAGTGGAGGCGT | 22 |
| PC-3p-54212_29 | TAGAGCCGCGGCCCCATTCAGA | 22 |
| PC-5p-522566_2 | ACTTCTTGATTGCAGAGT | 18 |
| PC-3p-4002241_1 | GAGCATGTGGAAGAAGCTGAGTGGGC | 26 |
| PC-5p-400729_2 | TTCAGCAGTAACTGAGTGACATTGG | 25 |
| PC-3p-2018000_1 | TGTCCTCTCCTACACTTGAGATAGTC | 26 |
| PC-5p-642529_2 | GGAGTACCTTGATGCTAAACCCATC | 25 |
| PC-3p-442030_2 | TTGAAAAGATGAGTTTGGTCCCAAGG | 26 |
| PC-5p-182660_5 | ACTGGGCTCAGGCTGTCA | 18 |
| PC-3p-627107_2 | AGAATGAAGGCCCACATCGACATG | 24 |
| PC-5p-3549035_1 | AAAAGAACAAAGTGTTAAATAATGTT | 26 |
| PC-3p-3122404_1 | TTTTAAAGAGGAGGTCCAGAGTAAGC | 26 |
| PC-5p-4586989_1 | TCCTGCCTGTCCTGGGATGGGAC | 23 |
| PC-3p-243151_4 | TCAACCTTGGTACCAGGCTAGA | 22 |
| PC-5p-40957_42 | TATTTGTGAACACACCGTAAGAGC | 24 |
| PC-3p-72698_19 | ATACGATTAGTTCTTACAGTGGTC | 24 |
| PC-5p-150449_7 | TTCAGTTATCATAGTACTGTACT | 23 |
| PC-3p-52162_30 | CACCGGTACCATGATAACTGA | 21 |
| PC-5p-57538_26 | TCGTTATGCATGTTGTCTTACTCC | 24 |
| PC-3p-492751_2 | TATTAGGTGAGGTGCATCTCTGTA | 24 |
| PC-5p-93176_13 | TACAGCCTTCACGACACCTGTT | 22 |
| PC-3p-339230_3 | TTCATGCGTATGATGCTGAGCAT | 23 |
| PC-5p-175643_5 | TCAAATAGTCTCTGCGCCAGCTGTT | 25 |
| PC-3p-324797_3 | TCTGCCGGTATTTCCACCTTCGACC | 25 |
| PC-5p-136792_7 | ATCTATCAGATGCCAAGTTAGC | 22 |
| PC-3p-195974_5 | AGGACACTAGATGACATGA | 19 |
| PC-5p-322750_3 | GAGGCCAACATATCTTTGGCATCCGC | 26 |
| PC-3p-3428228_1 | ATCTTGACTGGGTTGACAACATAAGG | 26 |
| PC-5p-46352_36 | ACAGTTGAGACTCTGAGA | 18 |
| PC-3p-3720142_1 | ATGATTGTGACACCCAGACAGACTC | 25 |
| PC-5p-466153_2 | TGAAGATGAAAATTATGATGCAAGAC | 26 |
| PC-5p-3728595_1 | ATGGTGGTGGTCGGAGAGGGCTGT | 24 |
| PC-5p-621078_2 | AGCACTGCGCGTTTTTGA | 18 |
| PC-5p-2630547_1 | CCGGGCCATTCAATCAGACGGCACTC | 26 |
| PC-5p-4244870_1 | CGTAGACATACACGCTGATAGCT | 23 |
| PC-5p-695083_2 | CCATGTTCACTGTTGCCCTGGCT | 23 |
| PC-3p-461914_2 | GACACTGGACCGTCCTTTAGA | 21 |
| PC-5p-202384_5 | TGGTCTCTGTGGATTTGAGT | 20 |
| PC-3p-3692732_1 | TCATGTCTCGTCGTTTTCACATGAGT | 26 |
| PC-3p-1933629_1 | GTCCCCAGCCTTGGCTTCTTTAGGGT | 26 |
| PC-5p-354029_3 | ATTCTTTTTGGTGCTGAAG | 19 |
| PC-5p-340929_3 | TATGTCTATTAATTTGTCAATTTAAA | 26 |
| PC-3p-63970_23 | GGAGGTGGAGCGTGAGCG | 18 |
| PC-5p-729413_2 | GAACATGATGTAAAGCGCTTTGGG | 24 |
| PC-5p-669453_2 | TAGACTGTGTTTGAGTGCACTCT | 23 |
| PC-3p-167205_6 | TCCTGAGACTGGGCCTCACA | 20 |
| PC-5p-360466_3 | AAAAGGATGATAAAGCGAGTGATA | 24 |
| PC-5p-740194_2 | TGTTCAAATCCGACTAAAACC | 21 |
| PC-3p-515807_2 | GGGGAGACTGGGAGAGGGTCAGA | 23 |
| PC-3p-2087199_1 | GACTAAGGAGTTCAGCTTCTGGATCT | 26 |
| PC-5p-512034_2 | TGCCTGACACGCCATCCTTCCAGTTC | 26 |
| PC-3p-4618816_1 | TTGTGACAGTCGTGGAATGTCGT | 23 |
| PC-5p-454950_2 | GTCTCTCGATGGCTAGGAAGCT | 22 |
| PC-5p-514217_2 | AGTCGAATGGGCTCCAGTGAGGTGG | 25 |
| PC-5p-394615_2 | TCCCTCCTTGTAAATCGCA | 19 |
| PC-3p-165812_6 | TTTCGTCCTGGTTTAGACCT | 20 |
| PC-5p-270697_3 | ACCGCAGCGGCACCGTGGATTGT | 23 |
| PC-5p-2196480_1 | CTAATATAGTGTGTAAAATTGTG | 23 |
| PC-3p-79666_16 | TCATCTCCATTTACCTGGTGC | 21 |
| PC-3p-241172_4 | GAGAGGGTTGCGTCAGGAAGG | 21 |
| PC-5p-201121_5 | GAGTTTGACTTCTTCAGATTTC | 22 |
| PC-3p-185775_5 | ATGGCAATGTCTGTGAAGG | 19 |
| PC-5p-236554_4 | ATTTTATTATTTTTAGCTT | 19 |
| PC-3p-420423_2 | GTTATCAGGCTTCAGGTCA | 19 |
| PC-3p-4769719_1 | TTTATCAGAGACACCTATTCAGC | 23 |
| PC-5p-2390540_1 | GATTTGAAAGCGTTGTAAGATTATGG | 26 |
| PC-3p-563372_2 | ACGACTTGCACTCGCTCCATCT | 22 |
| PC-5p-482511_2 | AAAGAAGAACTGCTGAATGAGC | 22 |
| PC-5p-603367_2 | TCAGGATGGTCATGAATGGCT | 21 |
| PC-3p-257220_4 | TACGTGCCATAAAGCGAGCCTT | 22 |
| PC-3p-4743745_1 | TCAGATCCAACTAAAACCTGTTCAGA | 26 |
| PC-5p-576924_2 | GGATGCCTTTGACAGTCTACCCATGT | 26 |
| PC-5p-382066_2 | ATGCTGTACATGTGTCTGTTCATCC | 25 |
| PC-5p-590851_2 | CTGTGTCTGCGTAGGTTTT | 19 |
| PC-3p-194638_5 | TGCGAATGTGCTCTAGTTACAGCCT | 25 |
| PC-5p-280450_3 | TTGTCTTTTGAACATAGCTGAGGC | 24 |
| PC-5p-1319691_1 | GCCTTGGCCAAAGCGGTTCAGATCC | 25 |
| PC-5p-583366_2 | TCTTAAAGGGACAGTGCAGTATTTTG | 26 |
| PC-3p-2964087_1 | GATTTCTACAAGTGGCTGACGGA | 23 |
| PC-5p-690190_2 | TTTCTGAGGAAGCTGAGGAAGT | 22 |
| PC-5p-242094_4 | ACGATCCTCCTGGTACCA | 18 |
| PC-3p-4650422_1 | TTGAGGACAGGACCCTGACAGGGCC | 25 |
| PC-5p-4615333_1 | CTCTGTTTGTGTATGTACTCTGTGTG | 26 |
| PC-3p-4482998_1 | GAAATGCTGCAACCATCCCTACC | 23 |
| PC-5p-650395_2 | CGCGTCTTGTAAGAGGAG | 18 |
| PC-5p-44518_38 | TTCGGCGAATACAGTGGAAT | 20 |
| PC-5p-713515_2 | CGGCTGAGTTATATTCTGAGTTC | 23 |
| PC-3p-4663508_1 | TGTGACGTAATGTATTTTTTGTTTTT | 26 |
| PC-5p-297203_3 | TTTTAGTTGGATTTGAACAGGT | 22 |
| PC-5p-546977_2 | TGAGCCTAGTGCTGGAGGTC | 20 |
| PC-5p-183921_5 | AAGCAGCACATCATACACTGGTA | 23 |
| PC-3p-181754_5 | AAATGTATAATGTGCAGGA | 19 |
| PC-3p-192161_5 | CTGTTTTGTCTGTTCTGAT | 19 |
| PC-5p-401249_2 | GGAAATGATAGTGGACATGAGG | 22 |
| PC-3p-102298_11 | TTCCAAAGTAGGCTCTGGCTCC | 22 |
| PC-3p-1383588_1 | TTATCTCCAGTGGACATGTCGTGGC | 25 |
| PC-5p-121028_9 | TTTGTAAACATTTATCTCTATCTGT | 25 |
| PC-5p-1597048_1 | TCATCGTGGAGATGGACAACAGCC | 24 |
| PC-3p-3284064_1 | TGAACAGGTTTTAGATGGATTTGAAC | 26 |
| PC-3p-422509_2 | AGACGAGGACAGAGCTGCTGC | 21 |
| PC-5p-181730_5 | AGGCAGTGTTATGTTAGCTGA | 21 |
| PC-5p-234998_4 | TATGTGTGTATGTACTGTATGTG | 23 |
| PC-5p-242625_4 | TTTGGACCTGGTTTAGACC | 19 |
| PC-5p-225981_4 | TAGCAGCATTAAGTGTAGTTATTGC | 25 |
