# Supplementary material for: Integrated analysis of mRNA-seq and miRNA-seq reveals the potential roles of sex-biased miRNA-mRNA pairs in gonad tissue of dark sleeper (Odontobutis potamophila)
Source: BMC Genomics. 2017 Aug 14;18:613. doi: 10.1186/s12864-017-3995-9 (PMC5557427; doi:10.1186/s12864-017-3995-9)
Supplement: Supplementary file 12 — List of differentially expressed miRNAs of dark sleeper in testis and ovary. (DOCX 34 kb) [file 12864_2017_3995_MOESM12_ESM.docx]

**TableS9** List of differentially expressed miRNAs of dark sleeper in testis and ovary

| Index | miR_name | miR_seq | up/down | log2(fold_change) | pvalue(t_test) | OO(mean) | OT(mean) | Expression level |
| --- | --- | --- | --- | --- | --- | --- | --- | --- |
| 1 | dre-miR-221-5p_R-4 | ACCTGGCATACAATGTAGATTT | down | -1.74 | 2.94E-05 | 380 | 114 | middle |
| 2 | cgr-miR-221-5p_R-4 | ACCTGGCATACAATGTAGATTT | down | -1.74 | 2.94E-05 | 380 | 114 | middle |
| 3 | aca-miR-138-5p_R+1 | AGCTGGTGTTGTGAATCAGGCCG | up | 2.29 | 4.06E-04 | 138 | 675 | middle |
| 4 | cfa-miR-153_R+2 | TTGCATAGTCACAAAAGTGATC | down | -0.97 | 5.22E-04 | 1,646 | 840 | middle |
| 5 | dre-miR-145-3p | GGATTCCTGGAAATACTGTTCT | up | 1.74 | 5.75E-04 | 3,400 | 11,326 | high |
| 6 | xtr-miR-145_R-2 | GTCCAGTTTTCCCAGGAATCCC | up | 2.91 | 6.12E-04 | 18,931 | 141,970 | high |
| 7 | fru-miR-222_R-1 | AGCTACATCTGGCTACTGGGTCT | down | -1.53 | 7.12E-04 | 6,447 | 2,238 | middle |
| 8 | ssa-miR-222a-3p_R-1 | AGCTACATCTGGCTACTGGGTCT | down | -1.53 | 7.12E-04 | 6,447 | 2,238 | middle |
| 9 | ola-miR-139_R+2 | TCTACAGTGCATGTGTCTCCAGT | down | -1.30 | 1.12E-03 | 1,805 | 732 | middle |
| 10 | tgu-miR-139-5p | TCTACAGTGCATGTGTCTCCAGT | down | -1.30 | 1.12E-03 | 1,805 | 732 | middle |
| 11 | dre-miR-143_R-3_1ss18GA | TGAGATGAAGCACTGTAA | up | 2.85 | 1.38E-03 | 1 | 7 | low |
| 12 | dre-miR-17a-2-3p_L-1_1ss19CA | CTGCAGTGGAGGCACTTAAAGC | down | -1.07 | 1.39E-03 | 63 | 30 | middle |
| 13 | dre-miR-222a-5p_R-1_1ss11TG | TGCTCAGTAGGCAGTGTAGATC | down | -1.43 | 1.42E-03 | 2,355 | 872 | middle |
| 14 | ssa-miR-222a-5p_R-2 | TGCTCAGTAGGCAGTGTAGATC | down | -1.43 | 1.42E-03 | 2,355 | 872 | middle |
| 15 | dre-miR-133a-5p | AGCTGGTAAAATGGAACCAAAT | up | 4.90 | 1.79E-03 | 4 | 126 | middle |
| 16 | ola-miR-133-5p_R-1 | AGCTGGTAAAATGGAACCAAAT | up | 4.90 | 1.79E-03 | 4 | 126 | middle |
| 17 | gga-miR-1a-3p_R+1 | TGGAATGTAAAGAAGTATGTAT | up | 3.70 | 1.96E-03 | 191 | 2,479 | middle |
| 18 | ola-miR-184-5p_L-1R+2 | CCTTATCACTTTTCCAGCCCAGC | down | -inf | 1.97E-03 | 34 | 0 | middle |
| 19 | xtr-miR-92a_R+2 | TATTGCACTTGTCCCGGCCTGTT | down | -1.34 | 2.11E-03 | 4,532 | 1,790 | middle |
| 20 | dre-miR-92a-3p_R+1 | TATTGCACTTGTCCCGGCCTGTT | down | -1.34 | 2.11E-03 | 4,532 | 1,790 | middle |
| 21 | ssa-miR-725-3p_1ss20AC | TTCAGTCATTGTTTCTGGTCGT | up | 1.33 | 2.12E-03 | 46 | 116 | middle |
| 22 | ssa-miR-190a-3p | ACTATATATCAAACATATTCCT | up | 1.30 | 2.16E-03 | 334 | 820 | middle |
| 23 | bta-miR-221 | AGCTACATTGTCTGCTGGGTTT | down | -0.91 | 2.20E-03 | 5,700 | 3,040 | middle |
| 24 | cgr-miR-221-3p_R-1 | AGCTACATTGTCTGCTGGGTTT | down | -0.91 | 2.20E-03 | 5,700 | 3,040 | middle |
| 25 | aca-miR-214-3p | ACAGCAGGCACAGACAGGCAGT | down | -0.53 | 2.88E-03 | 13,116 | 9,069 | high |
| 26 | fru-miR-133_L-1R+1 | TTGGTCCCCTTCAACCAGCTGT | up | 4.19 | 2.96E-03 | 166 | 3,021 | middle |
| 27 | ola-miR-133-3p_L+1R+3 | TTGGTCCCCTTCAACCAGCTGT | up | 4.19 | 2.96E-03 | 166 | 3,021 | middle |
| 28 | oha-miR-133b-3p_R-2 | TTTGGTCCCCTTCAACCAGCT | up | 3.05 | 3.19E-03 | 18 | 150 | middle |
| 29 | PC-3p-731770_2 | TTATTGGATTAAAAAGTT | down | -3.93 | 3.57E-03 | 9 | 1 | middle |
| 30 | dre-miR-455-3p_L-2R+1 | GCAGTCCATGGGCATATACACT | down | -1.36 | 3.89E-03 | 864 | 338 | middle |
| 31 | gga-miR-1a-2-5p_R-1 | ACATACTTCTTTATGTACCCAT | up | 4.55 | 4.20E-03 | 3 | 65 | middle |
| 32 | gga-miR-33-5p | GTGCATTGTAGTTGCATTGC | up | 1.20 | 4.51E-03 | 212 | 489 | middle |
| 33 | ssa-miR-33b-5p_R-1 | GTGCATTGTAGTTGCATTGC | up | 1.20 | 4.51E-03 | 212 | 489 | middle |
| 34 | ssa-miR-2188-5p | AAGGTCCAACCTCACATGTCCT | down | -1.39 | 4.86E-03 | 8,515 | 3,244 | high |
| 35 | ola-miR-33_R+2 | CAATGTACCTGCAGTGCAACA | up | 1.27 | 4.95E-03 | 170 | 410 | middle |
| 36 | ola-miR-126-5p | CATTATTACTTTTGGTACGCG | up | 0.91 | 5.03E-03 | 2,685 | 5,060 | middle |
| 37 | pma-miR-199a-5p_R-1 | CCCAGTGTTCAGACTACCTGTT | up | 1.94 | 5.04E-03 | 6,362 | 24,480 | high |
| 38 | ssa-miR-734-5p | GAACTATTCTGCAACATTTGTT | up | 5.12 | 5.28E-03 | 4 | 142 | middle |
| 39 | ccr-miR-187 | TCGTGTCTTGTGTTGCAGCCAGT | down | -7.81 | 5.48E-03 | 851 | 4 | middle |
| 40 | hsa-miR-3120-3p_L+2R-1_1ss18CT | TGCACAGCAAGTGTAGATAGGC | down | -3.31 | 5.49E-03 | 6 | 1 | low |
| 41 | dre-miR-144-5p_1ss11GT | GGATATCATCTTATACTGTAAGT | down | -1.13 | 5.66E-03 | 635 | 290 | middle |
| 42 | ola-miR-144_L-1R+2 | GGATATCATCTTATACTGTAAGT | down | -1.13 | 5.66E-03 | 635 | 290 | middle |
| 43 | dre-miR-142a-5p | CATAAAGTAGAAAGCACTACT | down | -0.94 | 5.79E-03 | 5,117 | 2,668 | middle |
| 44 | ola-miR-142_L-2R+1 | CATAAAGTAGAAAGCACTACT | down | -0.94 | 5.79E-03 | 5,117 | 2,668 | middle |
| 45 | tni-miR-190_R+1 | TGATATGTTTGATATATTAGGTT | up | 1.09 | 5.80E-03 | 2,173 | 4,615 | middle |
| 46 | mmu-miR-466i-5p_L-2 | TGTGTGTGTGTGTGTGTG | up | 1.84 | 5.81E-03 | 2 | 9 | low |
| 47 | tni-miR-455 | TATGTGCCCTTGGACTACATCG | down | -1.39 | 5.94E-03 | 3,412 | 1,306 | middle |
| 48 | dre-miR-455-5p | TATGTGCCCTTGGACTACATCG | down | -1.39 | 5.94E-03 | 3,412 | 1,306 | middle |
| 49 | hsa-miR-26a-2-3p_1ss19TC | CCTATTCTTGATTACTTGCTTC | up | 0.77 | 6.40E-03 | 81 | 139 | middle |
| 50 | bta-miR-25 | CATTGCACTTGTCTCGGTCTGA | down | -1.02 | 6.60E-03 | 10,140 | 5,015 | high |
| 51 | PC-5p-382066_2 | ATGCTGTACATGTGTCTGTTCATCC | down | -4.53 | 7.31E-03 | 7 | 0 | low |
| 52 | PC-3p-61296_24 | AATATAGTTTACCTGATGCTCT | down | -1.69 | 8.27E-03 | 22 | 7 | middle |
| 53 | fru-miR-103 | AGCAGCATTGTACAGGGCTATGA | up | 0.73 | 8.28E-03 | 2,852 | 4,724 | middle |
| 54 | dre-miR-138-5p_R-2 | AGCTGGTGTTGTGAATCAGG | up | 2.11 | 8.92E-03 | 112 | 482 | middle |
| 55 | dre-miR-210-3p | CTGTGCGTGTGACAGCGGCTAA | up | 1.20 | 9.20E-03 | 1,815 | 4,174 | middle |
| 56 | efu-miR-423_R-3 | TGAGGGGCAGAGAGCGAGACT | down | -2.18 | 9.68E-03 | 1 | 0 | low |
| 57 | bta-miR-423-5p_R-2 | TGAGGGGCAGAGAGCGAGACT | down | -2.18 | 9.68E-03 | 1 | 0 | low |
| 58 | pmi-let-7-5p_1ss10CA | TGAGGTAGTAGGTTGTAAAGA | up | 2.15 | 9.89E-03 | 0 | 2 | low |
| 59 | hsa-miR-29b-1-5p_3ss1GA11TG19TC | ACTGGTTTCAGATGGTGGCTTAGA | up | 0.65 | 1.02E-02 | 25 | 39 | middle |
| 60 | dre-miR-30e-5p_R-2 | TGTAAACATCCTTGACTGGA | up | 0.49 | 1.03E-02 | 21,369 | 29,934 | high |
| 61 | ssa-miR-130a-5p | ACTCTTTCCCTGTTGCACTACT | down | -0.75 | 1.17E-02 | 283 | 168 | middle |
| 62 | ccr-miR-184_R-2 | TGGACGGAGAACTGATAAGG | down | -7.95 | 1.26E-02 | 5,011 | 20 | middle |
| 63 | ssa-miR-20b-5p_1ss20GA | CAAAGTGCTCACAGTGCAGATA | down | -1.63 | 1.26E-02 | 603 | 195 | middle |
| 64 | dre-miR-2184_R+1 | AACAGTAAGAGTTTATGTGCTG | up | 3.76 | 1.38E-02 | 237 | 3,212 | middle |
| 65 | oha-miR-143-5p_L-1R-1 | GGTGCAGTGCTGCATCTCTGG | up | 2.24 | 1.42E-02 | 683 | 3,220 | middle |
| 66 | ssa-miR-8160-5p_1ss21CT | AGAATAATGCCAGCAGTCGGTC | down | -inf | 1.54E-02 | 130 | 0 | middle |
| 67 | dre-miR-210-5p | AGCCACTGACTAACGCACATTG | up | 0.82 | 1.56E-02 | 1,671 | 2,947 | middle |
| 68 | PC-5p-202384_5 | TGGTCTCTGTGGATTTGAGT | up | 2.70 | 1.58E-02 | 0 | 3 | low |
| 69 | ssa-let-7a-4-3p_R-1 | CTATACAGTCTATTGCCTTCC | down | -0.64 | 1.60E-02 | 153 | 98 | middle |
| 70 | oha-miR-143-3p_1ss22AT | TGAGATGAAGCACTGTAGCTCT | up | 3.18 | 1.61E-02 | 29,836 | 271,042 | high |
| 71 | dre-miR-93 | AAAAGTGCTGTTTGTGCAGGTA | down | -0.68 | 1.65E-02 | 4,002 | 2,495 | middle |
| 72 | dre-miR-2187-5p_R-1 | TTAATTAGTATAGCCTGTTTT | up | 6.51 | 1.73E-02 | 12 | 1,138 | middle |
| 73 | dre-miR-7147 | TGTACCATGCTGGTAGCCAGT | up | 0.64 | 1.75E-02 | 70 | 109 | middle |
| 74 | PC-5p-423534_2 | TGTCTCTGTAGATAGGACC | down | -inf | 1.78E-02 | 3 | 0 | low |
| 75 | ccr-miR-365 | TAATGCCCCTAAAAATCCTTAT | up | 0.75 | 1.84E-02 | 532 | 895 | middle |
| 76 | aca-miR-365-3p | TAATGCCCCTAAAAATCCTTAT | up | 0.75 | 1.84E-02 | 532 | 895 | middle |
| 77 | mmu-miR-466i-5p_R-1 | TGTGTGTGTGTGTGTGTGT | up | 2.46 | 1.89E-02 | 1 | 6 | low |
| 78 | PC-5p-282_8970 | TAGCAGCACATCATTACTGGTA | down | -0.64 | 1.92E-02 | 3,559 | 2,290 | middle |
| 79 | aca-miR-138-2-3p_L-2R+1_1ss11AC | GCTACTTCCCAACACCAGGGT | up | 2.69 | 1.94E-02 | 0 | 2 | low |
| 80 | ccr-miR-551_1ss14GA | GCGACCCATCCTTAGTTTCTG | down | -0.95 | 1.98E-02 | 56 | 29 | middle |
| 81 | ccr-miR-454b_R+2 | TAGTGCAATATTGCTTATAGGGT | down | -0.71 | 2.04E-02 | 1,065 | 651 | middle |
| 82 | dre-miR-737-3p | AATCAAAACCTAAAGAAAATA | up | 0.62 | 2.11E-02 | 118 | 181 | middle |
| 83 | dre-miR-125b-2-3p_L+1_2ss8GA11TC | ACGGGTTAGGCTCTCGGGAGCT | down | -0.74 | 2.12E-02 | 57 | 34 | middle |
| 84 | tgu-miR-125-2-3p_R+1_1ss15TC | ACGGGTTAGGCTCTCGGGAGCT | down | -0.74 | 2.12E-02 | 57 | 34 | middle |
| 85 | hsa-miR-4508_L+1_1ss17CT | AGCGGGGCTGGGCGCGTG | down | -1.14 | 2.13E-02 | 18 | 8 | middle |
| 86 | oan-miR-29a-3p_R+1 | TAGCACCATTTGAAATCGGTTA | up | 1.55 | 2.16E-02 | 1,317 | 3,859 | middle |
| 87 | cgr-miR-29c-3p_R+1 | TAGCACCATTTGAAATCGGTTA | up | 1.55 | 2.16E-02 | 1,317 | 3,859 | middle |
| 88 | oha-miR-29a-3p | TAGCACCATTTGAAATCGGTTA | up | 1.55 | 2.16E-02 | 1,317 | 3,859 | middle |
| 89 | oha-miR-9-3p_L-2R+1 | ATACAGCTAGATAACCAAAGAT | up | inf | 2.23E-02 | 0 | 1 | low |
| 90 | dre-miR-22a-5p_R-1 | AGTTCTTCACTGGCAAGCTTT | up | 1.11 | 2.37E-02 | 1,723 | 3,728 | middle |
| 91 | dre-miR-23a-3p_R-1 | ATCACATTGCCAGGGATTTCC | up | 0.19 | 2.37E-02 | 21,879 | 24,988 | high |
| 92 | ssa-miR-202-3p_R-1_1ss22GA | TTCCTATGCATATACCGCTTTA | up | 2.26 | 2.43E-02 | 9 | 43 | middle |
| 93 | ssa-miR-734-3p_1ss22TC | TAAATGCTGCAGAATTGTGCTC | up | 4.82 | 2.43E-02 | 10 | 288 | middle |
| 94 | ccr-miR-724 | TTAAAGGGAATTTGCGACTGTT | up | 1.56 | 2.50E-02 | 514 | 1,516 | middle |
| 95 | ssa-miR-23a-4-5p_L-1_1ss15AC | GGGTTCCTGGCACCGTGATTT | down | -1.92 | 2.52E-02 | 94 | 25 | middle |
| 96 | pmi-miR-31-5p_R+2 | AGGCAAGATGTTGGCATAGCTGT | down | -6.73 | 2.56E-02 | 1,082 | 10 | middle |
| 97 | gga-miR-31-5p_R+1 | AGGCAAGATGTTGGCATAGCTGT | down | -6.73 | 2.56E-02 | 1,082 | 10 | middle |
| 98 | mmu-miR-145b_R-2_1ss18GA | GTCCAGTTTTCCCAGGAAA | up | 3.03 | 2.67E-02 | 14 | 116 | middle |
| 99 | ssc-miR-202-5p_L+2_1ss18TC | CCTTCCTATGCATATACCTCTTT | up | 1.86 | 2.72E-02 | 35 | 129 | middle |
| 100 | dre-miR-2187-3p_L+1R-1_1ss21AG | TTTACAGGCTATGCTAATCTGT | up | 6.10 | 2.73E-02 | 20 | 1,396 | middle |
| 101 | ssa-miR-103-5p_R-1_2ss11TC13AG | AGCCTCTTTACGGTGCTGCCTTG | up | 0.61 | 2.89E-02 | 55 | 83 | middle |
| 102 | ssa-miR-8160-3p_1ss19GA | CCAGCACTGGTGTTATTGAGA | down | -inf | 2.92E-02 | 3 | 0 | low |
| 103 | bbe-miR-100-5p | AACCCGTAGATCCGAACTTGTG | up | 0.95 | 2.96E-02 | 86,545 | 167,387 | high |
| 104 | fru-miR-458 | ATAGCTCTTTAAATGGTACTGC | down | -2.16 | 3.09E-02 | 648 | 145 | middle |
| 105 | hsa-miR-3150b-5p_L-4_1ss9AC | CTCGCGGATCTCCCCAGC | down | -1.33 | 3.09E-02 | 87 | 35 | middle |
| 106 | PC-3p-448961_2 | TATTTTTATGATGAACTTT | down | -inf | 3.17E-02 | 3 | 0 | low |
| 107 | efu-miR-124_L-1R-1 | TAAGGCACGCGGTGAATGCCA | up | 2.51 | 3.27E-02 | 12 | 68 | middle |
| 108 | hsa-miR-124-3p_R+1 | TAAGGCACGCGGTGAATGCCA | up | 2.51 | 3.27E-02 | 12 | 68 | middle |
| 109 | pma-miR-181a-5p | AACATTCAACGCTGTCGGTGAGT | up | 0.50 | 3.27E-02 | 13,080 | 18,529 | high |
| 110 | oan-miR-1386_1ss1CA | ATCCTGGCTGGCTCGCCA | down | -1.71 | 3.29E-02 | 160 | 49 | middle |
| 111 | hsa-miR-223-5p_L-2R+3 | TGTATTTGACAAGCTGAGTTGGA | up | 1.21 | 3.34E-02 | 3 | 7 | low |
| 112 | csa-miR-92c_R-2_1ss9CT | TATTGCACTTGTCCCGGCCG | up | 2.61 | 3.45E-02 | 0 | 2 | low |
| 113 | ipu-miR-21_R-1 | TAGCTTATCAGACTGGTGTTGG | down | -1.34 | 3.53E-02 | 369,028 | 146,180 | high |
| 114 | dre-miR-19a-5p_L+1R-1 | GCTAGTTTTGCATAGTTGCACT | down | -1.18 | 3.53E-02 | 52 | 23 | middle |
| 115 | PC-5p-44099_38 | TCCAGTCGGAGGTCCGCTCACTAC | down | -6.60 | 3.56E-02 | 88 | 1 | middle |
| 116 | PC-3p-79666_16 | TCATCTCCATTTACCTGGTGC | down | -1.81 | 3.61E-02 | 6 | 2 | low |
| 117 | mmu-let-7f-1-3p_1ss22CT | CTATACAATCTATTGCCTTCCT | down | -0.91 | 3.68E-02 | 54 | 29 | middle |
| 118 | oha-miR-223-3p_R-3_1ss18TC | TGTCAGTTTGTCAAATACA | up | inf | 3.70E-02 | 0 | 1 | low |
| 119 | fru-miR-301_R+1 | CAGTGCAATAGTATTGTCATAGC | down | -0.88 | 3.73E-02 | 449 | 244 | middle |
| 120 | ssa-miR-301a-3p | CAGTGCAATAGTATTGTCATAGC | down | -0.88 | 3.73E-02 | 449 | 244 | middle |
| 121 | aca-miR-456_R+1 | CAGGCTGGTTAGATGGTTGTCT | down | -0.69 | 3.79E-02 | 930 | 578 | middle |
| 122 | hsa-miR-106b-5p_R-1_1ss10GT | TAAAGTGCTTACAGTGCAGA | down | -0.95 | 3.97E-02 | 10 | 5 | middle |
| 123 | ssa-miR-724-3p | CAGCCACACCTTCCTTTTAAGA | up | 1.28 | 4.00E-02 | 5 | 11 | middle |
| 124 | dre-let-7i | TGAGGTAGTAGTTTGTGCTGTT | up | 1.67 | 4.07E-02 | 11,040 | 35,064 | high |
| 125 | dre-let-7b | TGAGGTAGTAGGTTGTGTGGTT | up | 0.81 | 4.13E-02 | 15,987 | 27,967 | high |
| 126 | ssa-miR-24a-5p | TGCCTACTGAACTGGTATCAGT | down | -1.82 | 4.15E-02 | 1,953 | 554 | middle |
| 127 | dre-miR-10b-5p_R-1 | TACCCTGTAGAACCGAATTTGT | up | 0.73 | 4.16E-02 | 2,195 | 3,643 | middle |
| 128 | hsa-let-7d-5p_1ss16CT | AGAGGTAGTAGGTTGTATAGTT | up | 1.14 | 4.18E-02 | 32 | 71 | middle |
| 129 | xbo-miR-137_L+1R-1_1ss18AG | TTATTGCTTGAGAATACGCGTT | down | -1.83 | 4.29E-02 | 2 | 1 | low |
| 130 | PC-5p-141830_7 | TGTTTCTGATATTCTTGAGGCTCC | down | -2.90 | 4.31E-02 | 7 | 1 | low |
| 131 | dre-let-7a | TGAGGTAGTAGGTTGTATAGTT | up | 0.57 | 4.42E-02 | 137,769 | 204,482 | high |
| 132 | fru-miR-203 | GTGAAATGTTTAGGACCACTTG | up | 0.72 | 4.43E-02 | 58 | 95 | middle |
| 133 | aca-miR-203-3p | GTGAAATGTTTAGGACCACTTG | up | 0.72 | 4.43E-02 | 58 | 95 | middle |
| 134 | ola-miR-22_R+1 | AAGCTGCCAGCTGAAGAACTGT | up | 1.18 | 4.43E-02 | 50,420 | 114,599 | high |
| 135 | gga-mir-466-p3_1ss5AG | ATGTGTGTATATATATATATATA | up | inf | 4.43E-02 | 0 | 4 | low |
| 136 | dre-miR-130c-5p_1ss16TC | GCCCTTTTTCTGTTGCACTACT | down | -0.69 | 4.49E-02 | 51 | 32 | middle |
| 137 | PC-5p-182660_5 | ACTGGGCTCAGGCTGTCA | up | inf | 4.53E-02 | 0 | 19 | middle |
| 138 | cgr-miR-27a-3p | TTCACAGTGGCTAAGTTCCGC | up | 1.05 | 4.58E-02 | 480 | 994 | middle |
| 139 | oha-let-7i-5p_R+2_1 | TGAGGTAGTAGTTTGTGCTGTTAT | up | 2.15 | 4.59E-02 | 14 | 61 | middle |
| 140 | oha-let-7i-5p_R+2_2 | TGAGGTAGTAGTTTGTGCTGTTCA | up | 2.15 | 4.59E-02 | 14 | 61 | middle |
| 141 | PC-5p-613031_2 | GTGTGTTGCTGTTACTTCACTGTC | down | -2.51 | 4.80E-02 | 9 | 2 | middle |
| 142 | mml-miR-6134_R+3_1ss18GT | TGAGGTAGTAGGATGTATAGTT | up | 0.87 | 4.87E-02 | 8 | 15 | middle |
| 143 | hsa-miR-4454_L-2_1ss8AG | ATCCGGGTCACGGCACCA | down | -2.44 | 4.91E-02 | 850 | 157 | middle |
