# Supplementary material for: Integrated analysis of mRNA-seq and miRNA-seq reveals the potential roles of sex-biased miRNA-mRNA pairs in gonad tissue of dark sleeper (Odontobutis potamophila)
Source: BMC Genomics. 2017 Aug 14;18:613. doi: 10.1186/s12864-017-3995-9 (PMC5557427; doi:10.1186/s12864-017-3995-9)
Supplement: Supplementary file 13 — All SNP detected in gonad tissue of dark sleeper. (DOCX 262 kb) [file 12864_2017_3995_MOESM13_ESM.docx]

**Table S10** All SNP detected in gonad tissue of dark sleeper

| **transcript** | **Pos** | **Ref** | **OOa_OO** | **OOb_OO** | **OOc_OO** | **OTa_OT** | **OTc_OT** | **OTb_OT** |
| --- | --- | --- | --- | --- | --- | --- | --- | --- |
| comp17814_c0_seq1 | 87 | A | - | - | - | A->T | A->T | A->T |
| comp18108_c0_seq1 | 124 | A | - | - | - | A->T | A->T | A->T |
| comp18108_c0_seq1 | 129 | A | - | - | - | A->T | A->T | A->T |
| comp126012_c0_seq1 | 407 | A | - | - | - | A->T | A->T | A->T |
| comp180856_c0_seq1 | 300 | A | - | - | - | A->T | A->T | A->T |
| comp10394_c0_seq1 | 347 | A | - | - | - | A->T | A->T | A->T |
| comp18108_c0_seq1 | 102 | A | - | - | - | A->T | A->T | A->T |
| comp34147_c0_seq1 | 825 | A | - | - | - | A->T | A->T | A->T |
| comp34147_c0_seq1 | 832 | A | - | - | - | A->T | A->T | A->T |
| comp31638_c0_seq1 | 19 | A | - | - | - | A->T | A->T | A->T |
| comp20890_c0_seq1 | 1878 | A | - | - | - | A->T | A->T | A->T |
| comp26312_c0_seq2 | 189 | A | - | - | - | A->T | A->T | A->T |
| comp11151_c0_seq1 | 362 | A | - | - | - | A->T | A->T | A->T |
| comp18108_c0_seq1 | 92 | A | - | - | - | A->T | A->T | A->T |
| comp15092_c0_seq1 | 189 | A | - | - | - | A->T | A->T | A->T |
| comp126012_c0_seq1 | 421 | A | - | - | - | A->T | A->T | A->T |
| comp14335_c0_seq1 | 188 | A | - | - | - | A->T | A->T | A->T |
| comp31638_c0_seq1 | 13 | A | - | - | - | A->T | A->T | A->T |
| comp14548_c0_seq1 | 147 | A | - | - | - | A->T | A->T | A->T |
| comp829777_c0_seq1 | 27 | A | - | - | - | A->T | A->T | A->T |
| comp829777_c0_seq1 | 37 | A | - | - | - | A->T | A->T | A->T |
| comp829777_c0_seq1 | 39 | A | - | - | - | A->T | A->T | A->T |
| comp12145_c0_seq1 | 304 | A | - | - | - | A->T | A->T | A->T |
| comp17326_c0_seq1 | 205 | A | - | - | - | A->T | A->T | A->T |
| comp15471_c0_seq1 | 422 | A | - | - | - | A->T | A->T | A->T |
| comp13400_c0_seq1 | 268 | A | - | - | - | A->T | A->T | A->T |
| comp35451_c2_seq1 | 705 | A | - | - | - | A->T | A->T | A->T |
| comp18320_c0_seq1 | 107 | A | - | - | - | A->T | A->T | A->T |
| comp24724_c1_seq1 | 276 | A | - | - | - | A->T | A->T | A->T |
| comp17751_c0_seq1 | 738 | A | - | - | - | A->T | A->T | A->T |
| comp22651_c0_seq1 | 20 | A | - | - | - | A->T | A->T | A->T |
| comp17613_c0_seq1 | 515 | A | - | - | - | A->T | A->T | A->T |
| comp313815_c0_seq1 | 188 | A | - | - | - | A->T | A->T | A->T |
| comp17206_c0_seq1 | 331 | A | - | - | - | A->T | A->T | A->T |
| comp15111_c0_seq1 | 473 | A | - | - | - | A->T | A->T | A->T |
| comp10370_c0_seq1 | 194 | A | - | - | - | A->T | A->T | A->T |
| comp18538_c0_seq1 | 309 | A | - | - | - | A->T | A->T | A->T |
| comp16444_c0_seq1 | 28 | A | - | - | - | A->T | A->T | A->T |
| comp14270_c0_seq1 | 55 | A | - | - | - | A->T | A->T | A->T |
| comp14461_c0_seq1 | 216 | A | - | - | - | A->T | A->T | A->T |
| comp765_c0_seq1 | 83 | A | - | - | - | A->T | A->T | A->T |
| comp36615_c0_seq5 | 340 | A | - | - | - | A->T | A->T | A->T |
| comp17454_c0_seq1 | 990 | A | - | - | - | A->T | A->T | A->T |
| comp10370_c0_seq1 | 121 | A | - | - | - | A->T | A->T | A->T |
| comp12300_c0_seq1 | 173 | A | - | - | - | A->T | A->T | A->T |
| comp35472_c0_seq1 | 62 | A | - | - | - | A->T | A->T | A->T |
| comp18431_c0_seq1 | 98 | A | - | - | - | A->T | A->T | A->T |
| comp13609_c0_seq1 | 143 | A | - | - | - | A->T | A->T | A->T |
| comp36913_c0_seq1 | 4040 | A | - | - | - | A->T | A->T | A->T |
| comp11390_c0_seq1 | 293 | A | - | - | - | A->T | A->T | A->T |
| comp35944_c0_seq2 | 668 | A | - | - | - | A->T | A->T | A->T |
| comp16505_c0_seq1 | 163 | A | - | - | - | A->T | A->T | A->T |
| comp30230_c0_seq1 | 2516 | A | - | - | - | A->T | A->T | A->T |
| comp17317_c0_seq1 | 323 | A | - | - | - | A->T | A->T | A->T |
| comp13732_c1_seq1 | 270 | A | - | - | - | A->T | A->T | A->T |
| comp34147_c0_seq1 | 762 | A | - | - | - | A->T | A->T | A->T |
| comp12705_c0_seq1 | 175 | A | - | - | - | A->T | A->T | A->T |
| comp14468_c0_seq1 | 380 | A | - | - | - | A->T | A->T | A->T |
| comp21819_c1_seq1 | 99 | A | - | - | - | A->T | A->T | A->T |
| comp12880_c0_seq1 | 106 | A | - | - | - | A->T | A->T | A->T |
| comp17759_c0_seq2 | 1462 | A | - | - | - | A->T | A->T | A->T |
| comp13733_c0_seq1 | 81 | A | - | - | - | A->T | A->T | A->T |
| comp11682_c0_seq1 | 23 | A | - | - | - | A->T | A->T | A->T |
| comp30466_c1_seq1 | 1905 | A | - | - | - | A->T | A->T | A->T |
| comp17697_c0_seq1 | 121 | A | - | - | - | A->C | A->C | A->C |
| comp14617_c0_seq1 | 457 | A | - | - | - | A->C | A->C | A->C |
| comp1411_c0_seq1 | 318 | A | - | - | - | A->C | A->C | A->C |
| comp18908_c0_seq1 | 426 | A | - | - | - | A->C | A->C | A->C |
| comp12397_c0_seq1 | 109 | A | - | - | - | A->C | A->C | A->C |
| comp17454_c0_seq1 | 942 | A | - | - | - | A->C | A->C | A->C |
| comp13782_c0_seq1 | 323 | A | - | - | - | A->C | A->C | A->C |
| comp26308_c0_seq2 | 45 | A | - | - | - | A->C | A->C | A->C |
| comp17454_c0_seq1 | 976 | A | - | - | - | A->C | A->C | A->C |
| comp13538_c0_seq1 | 56 | A | - | - | - | A->C | A->C | A->C |
| comp17072_c0_seq1 | 348 | A | - | - | - | A->C | A->C | A->C |
| comp16613_c0_seq1 | 188 | A | - | - | - | A->C | A->C | A->C |
| comp16067_c0_seq1 | 247 | A | - | - | - | A->C | A->C | A->C |
| comp27713_c0_seq1 | 601 | A | - | - | - | A->C | A->C | A->C |
| comp24277_c0_seq1 | 38 | A | - | - | - | A->C | A->C | A->C |
| comp12988_c0_seq1 | 100 | A | - | - | - | A->C | A->C | A->C |
| comp24164_c0_seq1 | 603 | A | - | - | - | A->C | A->C | A->C |
| comp15806_c0_seq1 | 336 | A | - | - | - | A->C | A->C | A->C |
| comp17491_c0_seq1 | 1887 | A | - | - | - | A->C | A->C | A->C |
| comp36901_c0_seq1 | 2392 | A | - | - | - | A->C | A->C | A->C |
| comp17564_c0_seq1 | 129 | A | - | - | - | A->C | A->C | A->C |
| comp17454_c0_seq1 | 928 | A | - | - | - | A->C | A->C | A->C |
| comp17454_c0_seq1 | 896 | A | - | - | - | A->C | A->C | A->C |
| comp13618_c0_seq1 | 197 | A | - | - | - | A->C | A->C | A->C |
| comp17485_c0_seq1 | 613 | A | - | - | - | A->C | A->C | A->C |
| comp32581_c0_seq1 | 90 | A | - | - | - | A->C | A->C | A->C |
| comp33515_c1_seq1 | 3066 | A | - | - | - | A->C | A->C | A->C |
| comp4858_c0_seq1 | 75 | A | - | - | - | A->C | A->C | A->C |
| comp15727_c0_seq1 | 937 | A | - | - | - | A->C | A->C | A->C |
| comp17298_c0_seq1 | 764 | A | - | - | - | A->C | A->C | A->C |
| comp16551_c1_seq1 | 116 | A | - | - | - | A->C | A->C | A->C |
| comp33780_c0_seq2 | 194 | A | - | - | - | A->C | A->C | A->C |
| comp31638_c0_seq1 | 9 | A | - | - | - | A->C | A->C | A->C |
| comp17814_c0_seq1 | 194 | A | - | - | - | A->C | A->C | A->C |
| comp13546_c0_seq1 | 993 | A | - | - | - | A->C | A->C | A->C |
| comp2371_c0_seq1 | 137 | A | - | - | - | A->C | A->C | A->C |
| comp16507_c0_seq1 | 111 | A | - | - | - | A->C | A->C | A->C |
| comp1247468_c0_seq1 | 231 | A | - | - | - | A->C | A->C | A->C |
| comp25907_c0_seq9 | 84 | A | - | - | - | A->C | A->C | A->C |
| comp17206_c0_seq1 | 346 | A | - | - | - | A->C | A->C | A->C |
| comp13653_c0_seq1 | 115 | A | - | - | - | A->C | A->C | A->C |
| comp12736_c0_seq1 | 666 | A | - | - | - | A->C | A->C | A->C |
| comp1131337_c0_seq1 | 226 | A | - | - | - | A->C | A->C | A->C |
| comp116_c0_seq1 | 81 | A | - | - | - | A->C | A->C | A->C |
| comp12880_c0_seq1 | 112 | A | - | - | - | A->C | A->C | A->C |
| comp12880_c0_seq1 | 28 | A | - | - | - | A->C | A->C | A->C |
| comp17220_c0_seq1 | 345 | A | - | - | - | A->C | A->C | A->C |
| comp17621_c1_seq1 | 731 | A | - | - | - | A->C | A->C | A->C |
| comp12300_c0_seq1 | 66 | A | - | - | - | A->C | A->C | A->C |
| comp829777_c0_seq1 | 29 | A | - | - | - | A->C | A->C | A->C |
| comp11915_c0_seq1 | 206 | A | - | - | - | A->C | A->C | A->C |
| comp11915_c0_seq1 | 218 | A | - | - | - | A->C | A->C | A->C |
| comp13538_c0_seq1 | 9 | A | - | - | - | A->C | A->C | A->C |
| comp18108_c0_seq1 | 97 | A | - | - | - | A->C | A->C | A->C |
| comp34147_c0_seq1 | 853 | A | - | - | - | A->C | A->C | A->C |
| comp16733_c0_seq1 | 179 | A | - | - | - | A->C | A->C | A->C |
| comp29486_c1_seq1 | 1390 | A | - | - | - | A->C | A->C | A->C |
| comp18108_c0_seq1 | 101 | A | - | - | - | A->C | A->C | A->C |
| comp13647_c1_seq1 | 133 | A | - | - | - | A->C | A->C | A->C |
| comp34147_c0_seq1 | 833 | A | - | - | - | A->C | A->C | A->C |
| comp126012_c0_seq1 | 410 | A | - | - | - | A->C | A->C | A->C |
| comp126012_c0_seq1 | 408 | A | - | - | - | A->C | A->C | A->C |
| comp18045_c0_seq1 | 247 | A | - | - | - | A->C | A->C | A->C |
| comp10394_c0_seq1 | 290 | A | - | - | - | A->C | A->C | A->C |
| comp34147_c0_seq1 | 810 | A | - | - | - | A->C | A->C | A->C |
| comp1389618_c0_seq1 | 298 | A | - | - | - | A->C | A->C | A->C |
| comp35760_c0_seq1 | 14 | A | - | - | - | A->C | A->C | A->C |
| comp17955_c2_seq1 | 219 | A | - | - | - | A->G | A->G | A->G |
| comp10043_c0_seq1 | 61 | A | - | - | - | A->G | A->G | A->G |
| comp14270_c0_seq1 | 147 | A | - | - | - | A->G | A->G | A->G |
| comp35100_c0_seq4 | 508 | A | - | - | - | A->G | A->G | A->G |
| comp19073_c0_seq1 | 739 | A | - | - | - | A->G | A->G | A->G |
| comp36466_c0_seq2 | 931 | A | - | - | - | A->G | A->G | A->G |
| comp36761_c3_seq4 | 77 | A | - | - | - | A->G | A->G | A->G |
| comp34993_c0_seq1 | 116 | A | - | - | - | A->G | A->G | A->G |
| comp12880_c0_seq1 | 115 | A | - | - | - | A->G | A->G | A->G |
| comp36800_c0_seq11 | 245 | A | - | - | - | A->G | A->G | A->G |
| comp25541_c0_seq1 | 766 | A | - | - | - | A->G | A->G | A->G |
| comp1112596_c0_seq1 | 188 | A | - | - | - | A->G | A->G | A->G |
| comp16780_c1_seq1 | 141 | A | - | - | - | A->G | A->G | A->G |
| comp29813_c0_seq1 | 131 | A | - | - | - | A->G | A->G | A->G |
| comp13926_c0_seq1 | 171 | A | - | - | - | A->G | A->G | A->G |
| comp15081_c0_seq1 | 977 | A | - | - | - | A->G | A->G | A->G |
| comp13196_c0_seq1 | 328 | A | - | - | - | A->G | A->G | A->G |
| comp34701_c0_seq1 | 1412 | A | - | - | - | A->G | A->G | A->G |
| comp26388_c0_seq1 | 41 | A | - | - | - | A->G | A->G | A->G |
| comp17866_c0_seq1 | 89 | A | - | - | - | A->G | A->G | A->G |
| comp16279_c0_seq1 | 434 | A | - | - | - | A->G | A->G | A->G |
| comp13149_c0_seq1 | 453 | A | - | - | - | A->G | A->G | A->G |
| comp12880_c0_seq1 | 99 | A | - | - | - | A->G | A->G | A->G |
| comp1132626_c0_seq1 | 243 | A | - | - | - | A->G | A->G | A->G |
| comp14270_c0_seq1 | 159 | A | - | - | - | A->G | A->G | A->G |
| comp16505_c0_seq1 | 109 | A | - | - | - | A->G | A->G | A->G |
| comp34540_c0_seq1 | 409 | A | - | - | - | A->G | A->G | A->G |
| comp36199_c0_seq22 | 79 | A | - | - | - | A->G | A->G | A->G |
| comp13538_c0_seq1 | 65 | A | - | - | - | A->G | A->G | A->G |
| comp32884_c0_seq1 | 366 | A | - | - | - | A->G | A->G | A->G |
| comp11685_c0_seq1 | 148 | A | - | - | - | A->G | A->G | A->G |
| comp17697_c0_seq1 | 92 | A | - | - | - | A->G | A->G | A->G |
| comp20585_c0_seq2 | 415 | A | - | - | - | A->G | A->G | A->G |
| comp17476_c2_seq1 | 401 | A | - | - | - | A->G | A->G | A->G |
| comp27713_c0_seq1 | 558 | A | - | - | - | A->G | A->G | A->G |
| comp34241_c0_seq1 | 2822 | A | - | - | - | A->G | A->G | A->G |
| comp17454_c0_seq1 | 939 | A | - | - | - | A->G | A->G | A->G |
| comp13073_c0_seq1 | 350 | A | - | - | - | A->G | A->G | A->G |
| comp18046_c0_seq1 | 86 | A | - | - | - | A->G | A->G | A->G |
| comp13444_c0_seq1 | 268 | A | - | - | - | A->G | A->G | A->G |
| comp13662_c0_seq1 | 1061 | A | - | - | - | A->G | A->G | A->G |
| comp12988_c0_seq1 | 46 | A | - | - | - | A->G | A->G | A->G |
| comp4823_c0_seq1 | 168 | A | - | - | - | A->G | A->G | A->G |
| comp18705_c0_seq2 | 548 | A | - | - | - | A->G | A->G | A->G |
| comp22309_c0_seq1 | 947 | A | - | - | - | A->G | A->G | A->G |
| comp16938_c0_seq1 | 1462 | A | - | - | - | A->G | A->G | A->G |
| comp35812_c0_seq3 | 91 | A | - | - | - | A->G | A->G | A->G |
| comp28261_c0_seq2 | 1172 | A | - | - | - | A->G | A->G | A->G |
| comp15806_c0_seq1 | 294 | A | - | - | - | A->G | A->G | A->G |
| comp19610_c0_seq1 | 100 | A | - | - | - | A->G | A->G | A->G |
| comp16547_c0_seq1 | 189 | A | - | - | - | A->G | A->G | A->G |
| comp1305_c0_seq1 | 172 | A | - | - | - | A->G | A->G | A->G |
| comp17454_c0_seq1 | 929 | A | - | - | - | A->G | A->G | A->G |
| comp31818_c0_seq1 | 2557 | A | - | - | - | A->G | A->G | A->G |
| comp30547_c0_seq1 | 1811 | A | - | - | - | A->G | A->G | A->G |
| comp29207_c0_seq1 | 947 | A | - | - | - | A->G | A->G | A->G |
| comp9029_c0_seq1 | 300 | A | - | - | - | A->G | A->G | A->G |
| comp18554_c0_seq1 | 334 | A | - | - | - | A->G | A->G | A->G |
| comp16483_c0_seq1 | 1482 | A | - | - | - | A->G | A->G | A->G |
| comp17423_c0_seq1 | 613 | A | - | - | - | A->G | A->G | A->G |
| comp14522_c0_seq1 | 217 | A | - | - | - | A->G | A->G | A->G |
| comp537131_c0_seq1 | 468 | A | - | - | - | A->G | A->G | A->G |
| comp18243_c0_seq1 | 265 | A | - | - | - | A->G | A->G | A->G |
| comp17814_c0_seq1 | 157 | A | - | - | - | A->G | A->G | A->G |
| comp34543_c0_seq1 | 1009 | A | - | - | - | A->G | A->G | A->G |
| comp928044_c0_seq1 | 143 | A | - | - | - | A->G | A->G | A->G |
| comp17287_c0_seq1 | 65 | A | - | - | - | A->G | A->G | A->G |
| comp33078_c0_seq1 | 614 | A | - | - | - | A->G | A->G | A->G |
| comp17072_c0_seq1 | 309 | A | - | - | - | A->G | A->G | A->G |
| comp16585_c0_seq1 | 69 | A | - | - | - | A->G | A->G | A->G |
| comp16795_c0_seq1 | 116 | A | - | - | - | A->G | A->G | A->G |
| comp1050971_c0_seq1 | 275 | A | - | - | - | A->G | A->G | A->G |
| comp17454_c0_seq1 | 925 | A | - | - | - | A->G | A->G | A->G |
| comp14651_c0_seq1 | 1390 | A | - | - | - | A->G | A->G | A->G |
| comp13923_c0_seq1 | 950 | A | - | - | - | A->G | A->G | A->G |
| comp17814_c0_seq1 | 138 | A | - | - | - | A->G | A->G | A->G |
| comp13323_c0_seq1 | 118 | A | - | - | - | A->G | A->G | A->G |
| comp12397_c0_seq1 | 141 | A | - | - | - | A->G | A->G | A->G |
| comp14369_c2_seq1 | 135 | A | - | - | - | A->G | A->G | A->G |
| comp28506_c0_seq1 | 1681 | A | - | - | - | A->G | A->G | A->G |
| comp12880_c0_seq1 | 38 | A | - | - | - | A->G | A->G | A->G |
| comp22438_c0_seq1 | 93 | A | - | - | - | A->G | A->G | A->G |
| comp17866_c0_seq1 | 114 | A | - | - | - | A->G | A->G | A->G |
| comp30620_c0_seq1 | 408 | A | - | - | - | A->G | A->G | A->G |
| comp36913_c0_seq1 | 1848 | A | - | - | - | A->G | A->G | A->G |
| comp805703_c0_seq1 | 193 | A | - | - | - | A->G | A->G | A->G |
| comp18243_c0_seq1 | 262 | A | - | - | - | A->G | A->G | A->G |
| comp33780_c0_seq2 | 140 | A | - | - | - | A->G | A->G | A->G |
| comp10836_c0_seq1 | 127 | A | - | - | - | A->G | A->G | A->G |
| comp17561_c0_seq1 | 103 | A | - | - | - | A->G | A->G | A->G |
| comp17814_c0_seq1 | 167 | A | - | - | - | A->G | A->G | A->G |
| comp3815_c0_seq1 | 118 | A | - | - | - | A->G | A->G | A->G |
| comp14023_c0_seq1 | 234 | A | - | - | - | A->G | A->G | A->G |
| comp1050017_c0_seq1 | 205 | A | - | - | - | A->G | A->G | A->G |
| comp14424_c0_seq1 | 845 | A | - | - | - | A->G | A->G | A->G |
| comp16441_c0_seq1 | 437 | A | - | - | - | A->G | A->G | A->G |
| comp12880_c0_seq1 | 56 | A | - | - | - | A->G | A->G | A->G |
| comp15278_c0_seq1 | 788 | A | - | - | - | A->G | A->G | A->G |
| comp27713_c0_seq1 | 884 | A | - | - | - | A->G | A->G | A->G |
| comp17239_c0_seq2 | 55 | A | - | - | - | A->G | A->G | A->G |
| comp19084_c0_seq1 | 169 | A | - | - | - | A->G | A->G | A->G |
| comp1009704_c0_seq1 | 60 | A | - | - | - | A->G | A->G | A->G |
| comp34947_c0_seq1 | 444 | A | - | - | - | A->G | A->G | A->G |
| comp183581_c0_seq1 | 1876 | A | - | - | - | A->G | A->G | A->G |
| comp18656_c1_seq1 | 265 | A | - | - | - | A->G | A->G | A->G |
| comp33639_c0_seq2 | 4509 | A | - | - | - | A->G | A->G | A->G |
| comp17866_c0_seq1 | 109 | A | - | - | - | A->G | A->G | A->G |
| comp30606_c0_seq3 | 952 | A | - | - | - | A->G | A->G | A->G |
| comp10370_c0_seq1 | 184 | A | - | - | - | A->G | A->G | A->G |
| comp22870_c0_seq1 | 660 | A | - | - | - | A->G | A->G | A->G |
| comp16031_c1_seq1 | 197 | A | - | - | - | A->G | A->G | A->G |
| comp22404_c0_seq2 | 147 | A | - | - | - | A->G | A->G | A->G |
| comp4760_c0_seq1 | 77 | A | - | - | - | A->G | A->G | A->G |
| comp18437_c0_seq1 | 425 | A | - | - | - | A->G | A->G | A->G |
| comp17520_c1_seq1 | 246 | A | - | - | - | A->G | A->G | A->G |
| comp23734_c0_seq1 | 88 | A | - | - | - | A->G | A->G | A->G |
| comp10370_c0_seq1 | 127 | A | - | - | - | A->G | A->G | A->G |
| comp33678_c0_seq6 | 20 | A | - | - | - | A->G | A->G | A->G |
| comp23738_c0_seq1 | 100 | A | - | - | - | A->G | A->G | A->G |
| comp14986_c0_seq1 | 77 | A | - | - | - | A->G | A->G | A->G |
| comp36000_c0_seq1 | 54 | A | - | - | - | A->G | A->G | A->G |
| comp17898_c0_seq1 | 322 | A | - | - | - | A->G | A->G | A->G |
| comp18183_c0_seq1 | 280 | A | - | - | - | A->G | A->G | A->G |
| comp18208_c0_seq1 | 782 | A | - | - | - | A->G | A->G | A->G |
| comp17835_c1_seq1 | 308 | A | - | - | - | A->G | A->G | A->G |
| comp22050_c0_seq1 | 2201 | A | - | - | - | A->G | A->G | A->G |
| comp17206_c0_seq1 | 340 | A | - | - | - | A->G | A->G | A->G |
| comp17613_c0_seq1 | 371 | A | - | - | - | A->G | A->G | A->G |
| comp17409_c0_seq1 | 512 | A | - | - | - | A->G | A->G | A->G |
| comp24057_c0_seq1 | 502 | A | - | - | - | A->G | A->G | A->G |
| comp26388_c0_seq1 | 34 | A | - | - | - | A->G | A->G | A->G |
| comp12968_c0_seq1 | 126 | A | - | - | - | A->G | A->G | A->G |
| comp17921_c0_seq2 | 476 | A | - | - | - | A->G | A->G | A->G |
| comp14473_c0_seq1 | 170 | A | - | - | - | A->G | A->G | A->G |
| comp35342_c0_seq6 | 138 | A | - | - | - | A->G | A->G | A->G |
| comp26388_c0_seq1 | 37 | A | - | - | - | A->G | A->G | A->G |
| comp18538_c0_seq1 | 533 | A | - | - | - | A->G | A->G | A->G |
| comp17866_c0_seq1 | 104 | A | - | - | - | A->G | A->G | A->G |
| comp30593_c0_seq1 | 38 | A | - | - | - | A->G | A->G | A->G |
| comp14316_c0_seq1 | 150 | A | - | - | - | A->G | A->G | A->G |
| comp33603_c0_seq1 | 1970 | A | - | - | - | A->G | A->G | A->G |
| comp26388_c0_seq1 | 118 | A | - | - | - | A->G | A->G | A->G |
| comp34450_c0_seq1 | 2836 | A | - | - | - | A->G | A->G | A->G |
| comp36142_c0_seq1 | 626 | A | - | - | - | A->G | A->G | A->G |
| comp13196_c0_seq1 | 259 | A | - | - | - | A->G | A->G | A->G |
| comp16484_c0_seq1 | 173 | A | - | - | - | A->G | A->G | A->G |
| comp36532_c0_seq7 | 1348 | A | - | - | - | A->G | A->G | A->G |
| comp17697_c0_seq1 | 123 | A | - | - | - | A->G | A->G | A->G |
| comp10922_c0_seq1 | 156 | A | - | - | - | A->G | A->G | A->G |
| comp24319_c0_seq1 | 1163 | A | - | - | - | A->G | A->G | A->G |
| comp30606_c0_seq3 | 725 | A | - | - | - | A->G | A->G | A->G |
| comp1037986_c0_seq1 | 459 | A | - | - | - | A->G | A->G | A->G |
| comp14720_c0_seq1 | 121 | A | - | - | - | A->G | A->G | A->G |
| comp14912_c0_seq1 | 401 | A | - | - | - | A->G | A->G | A->G |
| comp32341_c1_seq2 | 623 | A | - | - | - | A->G | A->G | A->G |
| comp1081_c0_seq1 | 347 | A | - | - | - | A->G | A->G | A->G |
| comp14834_c0_seq1 | 1123 | A | - | - | - | A->G | A->G | A->G |
| comp23161_c0_seq2 | 539 | A | - | - | - | A->G | A->G | A->G |
| comp23161_c0_seq2 | 488 | A | - | - | - | A->G | A->G | A->G |
| comp14548_c0_seq1 | 68 | A | - | - | - | A->G | A->G | A->G |
| comp36913_c0_seq1 | 278 | A | - | - | - | A->G | A->G | A->G |
| comp33660_c7_seq9 | 1590 | A | - | - | - | A->G | A->G | A->G |
| comp1043421_c0_seq1 | 247 | A | - | - | - | A->G | A->G | A->G |
| comp20759_c0_seq1 | 981 | A | - | - | - | A->G | A->G | A->G |
| comp28773_c0_seq1 | 459 | A | - | - | - | A->G | A->G | A->G |
| comp1346_c0_seq1 | 228 | A | - | - | - | A->G | A->G | A->G |
| comp1459_c0_seq1 | 271 | A | - | - | - | A->G | A->G | A->G |
| comp12827_c0_seq1 | 1039 | A | - | - | - | A->G | A->G | A->G |
| comp185892_c0_seq1 | 163 | A | - | - | - | A->G | A->G | A->G |
| comp185892_c0_seq1 | 166 | A | - | - | - | A->G | A->G | A->G |
| comp36005_c0_seq1 | 435 | A | - | - | - | A->G | A->G | A->G |
| comp36005_c0_seq1 | 345 | A | - | - | - | A->G | A->G | A->G |
| comp11447_c0_seq1 | 811 | A | - | - | - | A->G | A->G | A->G |
| comp14689_c0_seq2 | 221 | A | - | - | - | A->G | A->G | A->G |
| comp10394_c0_seq1 | 319 | A | - | - | - | A->G | A->G | A->G |
| comp15469_c0_seq1 | 307 | A | - | - | - | A->G | A->G | A->G |
| comp18085_c0_seq1 | 195 | A | - | - | - | A->G | A->G | A->G |
| comp35207_c1_seq3 | 143 | A | - | - | - | A->G | A->G | A->G |
| comp36403_c0_seq3 | 34 | A | - | - | - | A->G | A->G | A->G |
| comp24956_c0_seq1 | 276 | A | - | - | - | A->G | A->G | A->G |
| comp34147_c0_seq1 | 846 | A | - | - | - | A->G | A->G | A->G |
| comp34147_c0_seq1 | 842 | A | - | - | - | A->G | A->G | A->G |
| comp13627_c1_seq1 | 634 | A | - | - | - | A->G | A->G | A->G |
| comp180856_c0_seq1 | 310 | A | - | - | - | A->G | A->G | A->G |
| comp12548_c0_seq1 | 158 | A | - | - | - | A->G | A->G | A->G |
| comp1043421_c0_seq1 | 226 | A | - | - | - | A->G | A->G | A->G |
| comp185892_c0_seq1 | 151 | A | - | - | - | A->G | A->G | A->G |
| comp180856_c0_seq1 | 262 | A | - | - | - | A->G | A->G | A->G |
| comp15794_c0_seq1 | 158 | A | - | - | - | A->G | A->G | A->G |
| comp15993_c0_seq1 | 92 | A | - | - | - | A->G | A->G | A->G |
| comp180856_c0_seq1 | 307 | A | - | - | - | A->G | A->G | A->G |
| comp180856_c0_seq1 | 268 | A | - | - | - | A->G | A->G | A->G |
| comp17309_c0_seq1 | 304 | A | - | - | - | A->G | A->G | A->G |
| comp227090_c0_seq1 | 561 | A | - | - | - | A->G | A->G | A->G |
| comp1043421_c0_seq1 | 210 | A | - | - | - | A->G | A->G | A->G |
| comp17814_c0_seq1 | 89 | A | - | - | - | A->G | A->G | A->G |
| comp17998_c0_seq1 | 271 | C | - | - | - | C->A | C->A | C->A |
| comp23195_c0_seq1 | 127 | C | - | - | - | C->A | C->A | C->A |
| comp17700_c0_seq1 | 239 | C | - | - | - | C->A | C->A | C->A |
| comp12880_c0_seq1 | 133 | C | - | - | - | C->A | C->A | C->A |
| comp17665_c0_seq1 | 2328 | C | - | - | - | C->A | C->A | C->A |
| comp12705_c0_seq1 | 174 | C | - | - | - | C->A | C->A | C->A |
| comp14863_c0_seq1 | 552 | C | - | - | - | C->A | C->A | C->A |
| comp17454_c0_seq1 | 948 | C | - | - | - | C->A | C->A | C->A |
| comp20525_c0_seq1 | 547 | C | - | - | - | C->A | C->A | C->A |
| comp25191_c0_seq1 | 2461 | C | - | - | - | C->A | C->A | C->A |
| comp18231_c0_seq1 | 767 | C | - | - | - | C->A | C->A | C->A |
| comp36901_c0_seq1 | 419 | C | - | - | - | C->A | C->A | C->A |
| comp17506_c0_seq1 | 638 | C | - | - | - | C->A | C->A | C->A |
| comp1574_c0_seq1 | 176 | C | - | - | - | C->A | C->A | C->A |
| comp17613_c0_seq1 | 453 | C | - | - | - | C->A | C->A | C->A |
| comp16547_c0_seq1 | 194 | C | - | - | - | C->A | C->A | C->A |
| comp17541_c1_seq1 | 448 | C | - | - | - | C->A | C->A | C->A |
| comp16929_c0_seq1 | 509 | C | - | - | - | C->A | C->A | C->A |
| comp13538_c0_seq1 | 20 | C | - | - | - | C->A | C->A | C->A |
| comp36508_c1_seq4 | 213 | C | - | - | - | C->A | C->A | C->A |
| comp36679_c0_seq2 | 827 | C | - | - | - | C->A | C->A | C->A |
| comp30593_c0_seq1 | 39 | C | - | - | - | C->A | C->A | C->A |
| comp10370_c0_seq1 | 195 | C | - | - | - | C->A | C->A | C->A |
| comp15773_c0_seq1 | 70 | C | - | - | - | C->A | C->A | C->A |
| comp17206_c0_seq1 | 334 | C | - | - | - | C->A | C->A | C->A |
| comp10370_c0_seq1 | 130 | C | - | - | - | C->A | C->A | C->A |
| comp35199_c0_seq4 | 624 | C | - | - | - | C->A | C->A | C->A |
| comp15621_c0_seq1 | 1274 | C | - | - | - | C->A | C->A | C->A |
| comp17952_c0_seq1 | 732 | C | - | - | - | C->A | C->A | C->A |
| comp1320_c0_seq1 | 16 | C | - | - | - | C->A | C->A | C->A |
| comp14997_c0_seq1 | 168 | C | - | - | - | C->A | C->A | C->A |
| comp17832_c0_seq1 | 367 | C | - | - | - | C->A | C->A | C->A |
| comp12736_c0_seq1 | 667 | C | - | - | - | C->A | C->A | C->A |
| comp36884_c0_seq3 | 1041 | C | - | - | - | C->A | C->A | C->A |
| comp17658_c0_seq1 | 106 | C | - | - | - | C->A | C->A | C->A |
| comp829777_c0_seq1 | 30 | C | - | - | - | C->A | C->A | C->A |
| comp1049874_c0_seq1 | 260 | C | - | - | - | C->A | C->A | C->A |
| comp18150_c0_seq1 | 164 | C | - | - | - | C->A | C->A | C->A |
| comp185892_c0_seq1 | 142 | C | - | - | - | C->A | C->A | C->A |
| comp34147_c0_seq1 | 835 | C | - | - | - | C->A | C->A | C->A |
| comp34147_c0_seq1 | 839 | C | - | - | - | C->A | C->A | C->A |
| comp18134_c0_seq1 | 238 | C | - | - | - | C->A | C->A | C->A |
| comp11151_c0_seq1 | 383 | C | - | - | - | C->A | C->A | C->A |
| comp1077695_c0_seq1 | 162 | C | - | - | - | C->A | C->A | C->A |
| comp18094_c0_seq1 | 511 | C | - | - | - | C->G | C->G | C->G |
| comp10394_c0_seq1 | 284 | C | - | - | - | C->G | C->G | C->G |
| comp12782_c0_seq1 | 464 | C | - | - | - | C->G | C->G | C->G |
| comp18108_c0_seq1 | 88 | C | - | - | - | C->G | C->G | C->G |
| comp14652_c0_seq1 | 1145 | C | - | - | - | C->G | C->G | C->G |
| comp1288_c0_seq1 | 105 | C | - | - | - | C->G | C->G | C->G |
| comp20890_c0_seq1 | 1885 | C | - | - | - | C->G | C->G | C->G |
| comp12388_c0_seq1 | 85 | C | - | - | - | C->G | C->G | C->G |
| comp34147_c0_seq1 | 852 | C | - | - | - | C->G | C->G | C->G |
| comp18108_c0_seq1 | 138 | C | - | - | - | C->G | C->G | C->G |
| comp14652_c0_seq1 | 1160 | C | - | - | - | C->G | C->G | C->G |
| comp14652_c0_seq1 | 1148 | C | - | - | - | C->G | C->G | C->G |
| comp16631_c0_seq1 | 284 | C | - | - | - | C->G | C->G | C->G |
| comp33660_c7_seq9 | 1596 | C | - | - | - | C->G | C->G | C->G |
| comp36913_c0_seq1 | 189 | C | - | - | - | C->G | C->G | C->G |
| comp829777_c0_seq1 | 33 | C | - | - | - | C->G | C->G | C->G |
| comp1003065_c0_seq1 | 127 | C | - | - | - | C->G | C->G | C->G |
| comp829777_c0_seq1 | 28 | C | - | - | - | C->G | C->G | C->G |
| comp18243_c0_seq1 | 516 | C | - | - | - | C->G | C->G | C->G |
| comp16851_c0_seq1 | 521 | C | - | - | - | C->G | C->G | C->G |
| comp34294_c0_seq1 | 39 | C | - | - | - | C->G | C->G | C->G |
| comp829777_c0_seq1 | 98 | C | - | - | - | C->G | C->G | C->G |
| comp12977_c0_seq1 | 512 | C | - | - | - | C->G | C->G | C->G |
| comp1734_c0_seq1 | 209 | C | - | - | - | C->G | C->G | C->G |
| comp13145_c0_seq1 | 103 | C | - | - | - | C->G | C->G | C->G |
| comp14316_c0_seq1 | 241 | C | - | - | - | C->G | C->G | C->G |
| comp13723_c0_seq1 | 131 | C | - | - | - | C->G | C->G | C->G |
| comp18562_c0_seq1 | 185 | C | - | - | - | C->G | C->G | C->G |
| comp14461_c0_seq1 | 199 | C | - | - | - | C->G | C->G | C->G |
| comp12280_c0_seq1 | 115 | C | - | - | - | C->G | C->G | C->G |
| comp17239_c0_seq2 | 91 | C | - | - | - | C->G | C->G | C->G |
| comp34885_c0_seq2 | 14 | C | - | - | - | C->G | C->G | C->G |
| comp36760_c0_seq1 | 1937 | C | - | - | - | C->G | C->G | C->G |
| comp17454_c0_seq1 | 963 | C | - | - | - | C->G | C->G | C->G |
| comp16675_c1_seq1 | 390 | C | - | - | - | C->G | C->G | C->G |
| comp13627_c0_seq1 | 144 | C | - | - | - | C->G | C->G | C->G |
| comp17239_c0_seq1 | 79 | C | - | - | - | C->G | C->G | C->G |
| comp10370_c0_seq1 | 145 | C | - | - | - | C->G | C->G | C->G |
| comp18145_c0_seq1 | 138 | C | - | - | - | C->G | C->G | C->G |
| comp17697_c0_seq1 | 75 | C | - | - | - | C->G | C->G | C->G |
| comp33258_c0_seq2 | 13 | C | - | - | - | C->G | C->G | C->G |
| comp28715_c0_seq1 | 1434 | C | - | - | - | C->T | C->T | C->T |
| comp33600_c0_seq3 | 155 | C | - | - | - | C->T | C->T | C->T |
| comp16780_c1_seq1 | 249 | C | - | - | - | C->T | C->T | C->T |
| comp12548_c0_seq1 | 182 | C | - | - | - | C->T | C->T | C->T |
| comp34938_c2_seq1 | 2 | C | - | - | - | C->T | C->T | C->T |
| comp11151_c0_seq1 | 380 | C | - | - | - | C->T | C->T | C->T |
| comp180856_c0_seq1 | 237 | C | - | - | - | C->T | C->T | C->T |
| comp27663_c0_seq1 | 40 | C | - | - | - | C->T | C->T | C->T |
| comp180856_c0_seq1 | 234 | C | - | - | - | C->T | C->T | C->T |
| comp14652_c0_seq1 | 1133 | C | - | - | - | C->T | C->T | C->T |
| comp19036_c0_seq1 | 82 | C | - | - | - | C->T | C->T | C->T |
| comp35760_c0_seq1 | 20 | C | - | - | - | C->T | C->T | C->T |
| comp35760_c0_seq1 | 19 | C | - | - | - | C->T | C->T | C->T |
| comp1037986_c0_seq1 | 435 | C | - | - | - | C->T | C->T | C->T |
| comp10394_c0_seq1 | 335 | C | - | - | - | C->T | C->T | C->T |
| comp34486_c0_seq1 | 647 | C | - | - | - | C->T | C->T | C->T |
| comp13880_c0_seq1 | 219 | C | - | - | - | C->T | C->T | C->T |
| comp14432_c0_seq1 | 312 | C | - | - | - | C->T | C->T | C->T |
| comp1563_c0_seq1 | 373 | C | - | - | - | C->T | C->T | C->T |
| comp921630_c0_seq1 | 456 | C | - | - | - | C->T | C->T | C->T |
| comp34147_c0_seq1 | 826 | C | - | - | - | C->T | C->T | C->T |
| comp36913_c0_seq1 | 1039 | C | - | - | - | C->T | C->T | C->T |
| comp13433_c0_seq1 | 531 | C | - | - | - | C->T | C->T | C->T |
| comp13433_c0_seq2 | 546 | C | - | - | - | C->T | C->T | C->T |
| comp14970_c1_seq1 | 776 | C | - | - | - | C->T | C->T | C->T |
| comp11447_c0_seq1 | 736 | C | - | - | - | C->T | C->T | C->T |
| comp11447_c0_seq1 | 808 | C | - | - | - | C->T | C->T | C->T |
| comp15079_c0_seq1 | 510 | C | - | - | - | C->T | C->T | C->T |
| comp19296_c0_seq1 | 169 | C | - | - | - | C->T | C->T | C->T |
| comp30980_c0_seq2 | 2868 | C | - | - | - | C->T | C->T | C->T |
| comp135_c0_seq1 | 285 | C | - | - | - | C->T | C->T | C->T |
| comp18108_c0_seq1 | 105 | C | - | - | - | C->T | C->T | C->T |
| comp11447_c0_seq1 | 688 | C | - | - | - | C->T | C->T | C->T |
| comp11447_c0_seq1 | 778 | C | - | - | - | C->T | C->T | C->T |
| comp11151_c0_seq1 | 389 | C | - | - | - | C->T | C->T | C->T |
| comp17790_c0_seq1 | 135 | C | - | - | - | C->T | C->T | C->T |
| comp12880_c0_seq1 | 55 | C | - | - | - | C->T | C->T | C->T |
| comp1283473_c0_seq1 | 109 | C | - | - | - | C->T | C->T | C->T |
| comp17611_c0_seq2 | 442 | C | - | - | - | C->T | C->T | C->T |
| comp1077833_c0_seq1 | 265 | C | - | - | - | C->T | C->T | C->T |
| comp21925_c0_seq1 | 25 | C | - | - | - | C->T | C->T | C->T |
| comp1201921_c0_seq1 | 393 | C | - | - | - | C->T | C->T | C->T |
| comp28354_c1_seq1 | 2680 | C | - | - | - | C->T | C->T | C->T |
| comp126012_c0_seq1 | 419 | C | - | - | - | C->T | C->T | C->T |
| comp36982_c0_seq2 | 287 | C | - | - | - | C->T | C->T | C->T |
| comp13238_c0_seq1 | 183 | C | - | - | - | C->T | C->T | C->T |
| comp17086_c0_seq1 | 173 | C | - | - | - | C->T | C->T | C->T |
| comp8371_c0_seq1 | 265 | C | - | - | - | C->T | C->T | C->T |
| comp17206_c0_seq1 | 379 | C | - | - | - | C->T | C->T | C->T |
| comp26040_c1_seq1 | 3631 | C | - | - | - | C->T | C->T | C->T |
| comp32172_c3_seq2 | 324 | C | - | - | - | C->T | C->T | C->T |
| comp17955_c1_seq1 | 695 | C | - | - | - | C->T | C->T | C->T |
| comp33806_c0_seq2 | 156 | C | - | - | - | C->T | C->T | C->T |
| comp11_c0_seq1 | 287 | C | - | - | - | C->T | C->T | C->T |
| comp22406_c0_seq1 | 164 | C | - | - | - | C->T | C->T | C->T |
| comp15471_c0_seq1 | 306 | C | - | - | - | C->T | C->T | C->T |
| comp35059_c0_seq1 | 1237 | C | - | - | - | C->T | C->T | C->T |
| comp35975_c1_seq2 | 1360 | C | - | - | - | C->T | C->T | C->T |
| comp17843_c1_seq1 | 1254 | C | - | - | - | C->T | C->T | C->T |
| comp36142_c0_seq1 | 629 | C | - | - | - | C->T | C->T | C->T |
| comp14816_c0_seq1 | 570 | C | - | - | - | C->T | C->T | C->T |
| comp17249_c0_seq1 | 246 | C | - | - | - | C->T | C->T | C->T |
| comp14432_c0_seq1 | 535 | C | - | - | - | C->T | C->T | C->T |
| comp31839_c0_seq1 | 80 | C | - | - | - | C->T | C->T | C->T |
| comp847610_c0_seq1 | 64 | C | - | - | - | C->T | C->T | C->T |
| comp36158_c0_seq2 | 2989 | C | - | - | - | C->T | C->T | C->T |
| comp28590_c0_seq1 | 283 | C | - | - | - | C->T | C->T | C->T |
| comp24434_c0_seq1 | 935 | C | - | - | - | C->T | C->T | C->T |
| comp19837_c0_seq2 | 889 | C | - | - | - | C->T | C->T | C->T |
| comp25757_c0_seq1 | 368 | C | - | - | - | C->T | C->T | C->T |
| comp34582_c0_seq2 | 710 | C | - | - | - | C->T | C->T | C->T |
| comp24697_c2_seq2 | 1686 | C | - | - | - | C->T | C->T | C->T |
| comp2315_c0_seq1 | 59 | C | - | - | - | C->T | C->T | C->T |
| comp18115_c1_seq1 | 187 | C | - | - | - | C->T | C->T | C->T |
| comp34789_c1_seq4 | 2 | C | - | - | - | C->T | C->T | C->T |
| comp36499_c0_seq1 | 2602 | C | - | - | - | C->T | C->T | C->T |
| comp29255_c0_seq3 | 888 | C | - | - | - | C->T | C->T | C->T |
| comp1237248_c0_seq1 | 149 | C | - | - | - | C->T | C->T | C->T |
| comp15794_c2_seq1 | 145 | C | - | - | - | C->T | C->T | C->T |
| comp30640_c1_seq2 | 1560 | C | - | - | - | C->T | C->T | C->T |
| comp34465_c0_seq2 | 802 | C | - | - | - | C->T | C->T | C->T |
| comp18183_c0_seq1 | 278 | C | - | - | - | C->T | C->T | C->T |
| comp36913_c0_seq1 | 941 | C | - | - | - | C->T | C->T | C->T |
| comp1038106_c0_seq1 | 193 | C | - | - | - | C->T | C->T | C->T |
| comp11363_c0_seq1 | 23 | C | - | - | - | C->T | C->T | C->T |
| comp17564_c0_seq1 | 82 | C | - | - | - | C->T | C->T | C->T |
| comp14126_c0_seq1 | 281 | C | - | - | - | C->T | C->T | C->T |
| comp14424_c0_seq1 | 521 | C | - | - | - | C->T | C->T | C->T |
| comp34283_c3_seq2 | 994 | C | - | - | - | C->T | C->T | C->T |
| comp31443_c1_seq1 | 1515 | C | - | - | - | C->T | C->T | C->T |
| comp32531_c0_seq4 | 500 | C | - | - | - | C->T | C->T | C->T |
| comp15428_c0_seq1 | 1306 | C | - | - | - | C->T | C->T | C->T |
| comp13039_c0_seq1 | 435 | C | - | - | - | C->T | C->T | C->T |
| comp17729_c0_seq1 | 215 | C | - | - | - | C->T | C->T | C->T |
| comp26163_c0_seq1 | 270 | C | - | - | - | C->T | C->T | C->T |
| comp20345_c1_seq1 | 240 | C | - | - | - | C->T | C->T | C->T |
| comp13157_c0_seq1 | 195 | C | - | - | - | C->T | C->T | C->T |
| comp17283_c0_seq1 | 363 | C | - | - | - | C->T | C->T | C->T |
| comp18719_c0_seq1 | 640 | C | - | - | - | C->T | C->T | C->T |
| comp36142_c0_seq1 | 429 | C | - | - | - | C->T | C->T | C->T |
| comp15737_c1_seq1 | 115 | C | - | - | - | C->T | C->T | C->T |
| comp2371_c0_seq1 | 132 | C | - | - | - | C->T | C->T | C->T |
| comp3204_c0_seq1 | 191 | C | - | - | - | C->T | C->T | C->T |
| comp1821_c0_seq1 | 599 | C | - | - | - | C->T | C->T | C->T |
| comp32784_c0_seq3 | 795 | C | - | - | - | C->T | C->T | C->T |
| comp801951_c0_seq1 | 119 | C | - | - | - | C->T | C->T | C->T |
| comp27260_c2_seq1 | 657 | C | - | - | - | C->T | C->T | C->T |
| comp34947_c0_seq1 | 520 | C | - | - | - | C->T | C->T | C->T |
| comp15870_c0_seq1 | 196 | C | - | - | - | C->T | C->T | C->T |
| comp19298_c1_seq1 | 302 | C | - | - | - | C->T | C->T | C->T |
| comp19014_c0_seq1 | 105 | C | - | - | - | C->T | C->T | C->T |
| comp14308_c0_seq1 | 552 | C | - | - | - | C->T | C->T | C->T |
| comp1134459_c0_seq1 | 10 | C | - | - | - | C->T | C->T | C->T |
| comp17600_c0_seq1 | 620 | C | - | - | - | C->T | C->T | C->T |
| comp16326_c0_seq1 | 245 | C | - | - | - | C->T | C->T | C->T |
| comp33780_c0_seq2 | 156 | C | - | - | - | C->T | C->T | C->T |
| comp17693_c0_seq1 | 465 | C | - | - | - | C->T | C->T | C->T |
| comp16993_c1_seq1 | 107 | C | - | - | - | C->T | C->T | C->T |
| comp2654_c0_seq1 | 391 | C | - | - | - | C->T | C->T | C->T |
| comp28618_c1_seq1 | 159 | C | - | - | - | C->T | C->T | C->T |
| comp28618_c1_seq1 | 156 | C | - | - | - | C->T | C->T | C->T |
| comp21438_c0_seq1 | 2285 | C | - | - | - | C->T | C->T | C->T |
| comp35016_c0_seq1 | 232 | C | - | - | - | C->T | C->T | C->T |
| comp17454_c0_seq1 | 975 | C | - | - | - | C->T | C->T | C->T |
| comp13315_c1_seq1 | 177 | C | - | - | - | C->T | C->T | C->T |
| comp30275_c0_seq1 | 2887 | C | - | - | - | C->T | C->T | C->T |
| comp21258_c0_seq1 | 518 | C | - | - | - | C->T | C->T | C->T |
| comp24663_c0_seq1 | 108 | C | - | - | - | C->T | C->T | C->T |
| comp17454_c0_seq1 | 924 | C | - | - | - | C->T | C->T | C->T |
| comp13627_c0_seq1 | 174 | C | - | - | - | C->T | C->T | C->T |
| comp29567_c0_seq1 | 162 | C | - | - | - | C->T | C->T | C->T |
| comp19298_c0_seq1 | 78 | C | - | - | - | C->T | C->T | C->T |
| comp35387_c0_seq1 | 2624 | C | - | - | - | C->T | C->T | C->T |
| comp13538_c0_seq1 | 13 | C | - | - | - | C->T | C->T | C->T |
| comp13516_c0_seq1 | 280 | C | - | - | - | C->T | C->T | C->T |
| comp34007_c0_seq1 | 853 | C | - | - | - | C->T | C->T | C->T |
| comp12880_c0_seq1 | 70 | C | - | - | - | C->T | C->T | C->T |
| comp1180224_c0_seq1 | 138 | C | - | - | - | C->T | C->T | C->T |
| comp18078_c0_seq1 | 766 | C | - | - | - | C->T | C->T | C->T |
| comp15951_c0_seq1 | 370 | C | - | - | - | C->T | C->T | C->T |
| comp17113_c0_seq1 | 297 | C | - | - | - | C->T | C->T | C->T |
| comp18320_c0_seq1 | 128 | C | - | - | - | C->T | C->T | C->T |
| comp16102_c0_seq1 | 763 | C | - | - | - | C->T | C->T | C->T |
| comp15832_c0_seq1 | 1130 | C | - | - | - | C->T | C->T | C->T |
| comp17454_c0_seq1 | 903 | C | - | - | - | C->T | C->T | C->T |
| comp15871_c0_seq1 | 399 | C | - | - | - | C->T | C->T | C->T |
| comp15386_c0_seq1 | 570 | C | - | - | - | C->T | C->T | C->T |
| comp31681_c0_seq1 | 398 | C | - | - | - | C->T | C->T | C->T |
| comp32617_c0_seq1 | 567 | C | - | - | - | C->T | C->T | C->T |
| comp13653_c0_seq1 | 122 | C | - | - | - | C->T | C->T | C->T |
| comp17239_c0_seq2 | 81 | C | - | - | - | C->T | C->T | C->T |
| comp32998_c0_seq1 | 1720 | C | - | - | - | C->T | C->T | C->T |
| comp34151_c0_seq1 | 845 | C | - | - | - | C->T | C->T | C->T |
| comp29257_c0_seq1 | 1623 | C | - | - | - | C->T | C->T | C->T |
| comp34847_c0_seq2 | 2665 | C | - | - | - | C->T | C->T | C->T |
| comp18141_c0_seq1 | 326 | C | - | - | - | C->T | C->T | C->T |
| comp8748_c1_seq1 | 200 | C | - | - | - | C->T | C->T | C->T |
| comp36524_c0_seq1 | 576 | C | - | - | - | C->T | C->T | C->T |
| comp34399_c0_seq2 | 1838 | C | - | - | - | C->T | C->T | C->T |
| comp22308_c0_seq1 | 1606 | C | - | - | - | C->T | C->T | C->T |
| comp17004_c0_seq1 | 143 | C | - | - | - | C->T | C->T | C->T |
| comp35203_c1_seq4 | 179 | C | - | - | - | C->T | C->T | C->T |
| comp15892_c0_seq1 | 431 | C | - | - | - | C->T | C->T | C->T |
| comp17595_c0_seq1 | 300 | C | - | - | - | C->T | C->T | C->T |
| comp14521_c0_seq1 | 409 | C | - | - | - | C->T | C->T | C->T |
| comp33258_c0_seq3 | 198 | C | - | - | - | C->T | C->T | C->T |
| comp32288_c2_seq1 | 744 | C | - | - | - | C->T | C->T | C->T |
| comp30460_c0_seq3 | 2074 | C | - | - | - | C->T | C->T | C->T |
| comp36905_c0_seq5 | 38 | C | - | - | - | C->T | C->T | C->T |
| comp35100_c0_seq4 | 565 | C | - | - | - | C->T | C->T | C->T |
| comp14325_c0_seq1 | 282 | C | - | - | - | C->T | C->T | C->T |
| comp21347_c0_seq1 | 861 | C | - | - | - | C->T | C->T | C->T |
| comp30826_c0_seq1 | 1169 | C | - | - | - | C->T | C->T | C->T |
| comp11_c0_seq1 | 321 | C | - | - | - | C->T | C->T | C->T |
| comp12988_c0_seq1 | 118 | C | - | - | - | C->T | C->T | C->T |
| comp36908_c0_seq9 | 277 | C | - | - | - | C->T | C->T | C->T |
| comp12977_c0_seq1 | 644 | C | - | - | - | C->T | C->T | C->T |
| comp34343_c0_seq8 | 2247 | C | - | - | - | C->T | C->T | C->T |
| comp1556_c0_seq1 | 977 | C | - | - | - | C->T | C->T | C->T |
| comp18031_c0_seq1 | 342 | C | - | - | - | C->T | C->T | C->T |
| comp34157_c0_seq1 | 2804 | C | - | - | - | C->T | C->T | C->T |
| comp34616_c0_seq1 | 2996 | C | - | - | - | C->T | C->T | C->T |
| comp31597_c0_seq3 | 2028 | C | - | - | - | C->T | C->T | C->T |
| comp36732_c1_seq8 | 316 | C | - | - | - | C->T | C->T | C->T |
| comp16505_c0_seq1 | 88 | C | - | - | - | C->T | C->T | C->T |
| comp22040_c0_seq2 | 75 | C | - | - | - | C->T | C->T | C->T |
| comp12880_c0_seq1 | 104 | C | - | - | - | C->T | C->T | C->T |
| comp994660_c0_seq1 | 155 | C | - | - | - | C->T | C->T | C->T |
| comp36761_c3_seq4 | 98 | C | - | - | - | C->T | C->T | C->T |
| comp12880_c0_seq1 | 98 | C | - | - | - | C->T | C->T | C->T |
| comp10574_c0_seq1 | 176 | C | - | - | - | C->T | C->T | C->T |
| comp17524_c0_seq1 | 725 | C | - | - | - | C->T | C->T | C->T |
| comp36898_c0_seq1 | 665 | C | - | - | - | C->T | C->T | C->T |
| comp12977_c0_seq1 | 446 | C | - | - | - | C->T | C->T | C->T |
| comp16124_c0_seq1 | 372 | C | - | - | - | C->T | C->T | C->T |
| comp18049_c1_seq1 | 1416 | C | - | - | - | C->T | C->T | C->T |
| comp17454_c0_seq1 | 969 | C | - | - | - | C->T | C->T | C->T |
| comp13047_c0_seq1 | 368 | C | - | - | - | C->T | C->T | C->T |
| comp17176_c0_seq1 | 557 | C | - | - | - | C->T | C->T | C->T |
| comp13125_c0_seq2 | 826 | G | - | - | - | G->A | G->A | G->A |
| comp18162_c0_seq1 | 261 | G | - | - | - | G->A | G->A | G->A |
| comp1721_c0_seq1 | 288 | G | - | - | - | G->A | G->A | G->A |
| comp23161_c0_seq2 | 1210 | G | - | - | - | G->A | G->A | G->A |
| comp36959_c0_seq2 | 516 | G | - | - | - | G->A | G->A | G->A |
| comp36761_c3_seq4 | 92 | G | - | - | - | G->A | G->A | G->A |
| comp36761_c3_seq4 | 93 | G | - | - | - | G->A | G->A | G->A |
| comp18656_c1_seq1 | 264 | G | - | - | - | G->A | G->A | G->A |
| comp20439_c0_seq1 | 433 | G | - | - | - | G->A | G->A | G->A |
| comp17051_c0_seq2 | 105 | G | - | - | - | G->A | G->A | G->A |
| comp33284_c0_seq1 | 154 | G | - | - | - | G->A | G->A | G->A |
| comp19084_c0_seq1 | 97 | G | - | - | - | G->A | G->A | G->A |
| comp14916_c0_seq1 | 226 | G | - | - | - | G->A | G->A | G->A |
| comp22040_c0_seq2 | 133 | G | - | - | - | G->A | G->A | G->A |
| comp35818_c1_seq1 | 685 | G | - | - | - | G->A | G->A | G->A |
| comp22040_c0_seq2 | 150 | G | - | - | - | G->A | G->A | G->A |
| comp36134_c2_seq1 | 457 | G | - | - | - | G->A | G->A | G->A |
| comp22040_c0_seq2 | 69 | G | - | - | - | G->A | G->A | G->A |
| comp14775_c0_seq1 | 233 | G | - | - | - | G->A | G->A | G->A |
| comp672_c1_seq1 | 485 | G | - | - | - | G->A | G->A | G->A |
| comp1919_c0_seq1 | 399 | G | - | - | - | G->A | G->A | G->A |
| comp29405_c1_seq1 | 241 | G | - | - | - | G->A | G->A | G->A |
| comp14896_c0_seq1 | 733 | G | - | - | - | G->A | G->A | G->A |
| comp34993_c0_seq1 | 97 | G | - | - | - | G->A | G->A | G->A |
| comp1073_c0_seq1 | 179 | G | - | - | - | G->A | G->A | G->A |
| comp25308_c1_seq1 | 2285 | G | - | - | - | G->A | G->A | G->A |
| comp32270_c0_seq1 | 3391 | G | - | - | - | G->A | G->A | G->A |
| comp17867_c0_seq1 | 60 | G | - | - | - | G->A | G->A | G->A |
| comp15533_c1_seq1 | 415 | G | - | - | - | G->A | G->A | G->A |
| comp36898_c0_seq1 | 5098 | G | - | - | - | G->A | G->A | G->A |
| comp17483_c0_seq1 | 108 | G | - | - | - | G->A | G->A | G->A |
| comp16026_c0_seq1 | 212 | G | - | - | - | G->A | G->A | G->A |
| comp35676_c0_seq1 | 2331 | G | - | - | - | G->A | G->A | G->A |
| comp16505_c0_seq1 | 76 | G | - | - | - | G->A | G->A | G->A |
| comp16031_c0_seq1 | 526 | G | - | - | - | G->A | G->A | G->A |
| comp15115_c0_seq1 | 167 | G | - | - | - | G->A | G->A | G->A |
| comp17873_c0_seq1 | 707 | G | - | - | - | G->A | G->A | G->A |
| comp18228_c0_seq1 | 780 | G | - | - | - | G->A | G->A | G->A |
| comp17807_c0_seq1 | 413 | G | - | - | - | G->A | G->A | G->A |
| comp33435_c0_seq1 | 1102 | G | - | - | - | G->A | G->A | G->A |
| comp29323_c0_seq1 | 1760 | G | - | - | - | G->A | G->A | G->A |
| comp18317_c0_seq1 | 904 | G | - | - | - | G->A | G->A | G->A |
| comp15579_c0_seq1 | 287 | G | - | - | - | G->A | G->A | G->A |
| comp33258_c0_seq2 | 148 | G | - | - | - | G->A | G->A | G->A |
| comp25734_c0_seq3 | 193 | G | - | - | - | G->A | G->A | G->A |
| comp32185_c0_seq5 | 202 | G | - | - | - | G->A | G->A | G->A |
| comp15115_c1_seq1 | 473 | G | - | - | - | G->A | G->A | G->A |
| comp10370_c0_seq1 | 149 | G | - | - | - | G->A | G->A | G->A |
| comp36801_c0_seq5 | 271 | G | - | - | - | G->A | G->A | G->A |
| comp17613_c0_seq1 | 563 | G | - | - | - | G->A | G->A | G->A |
| comp18431_c0_seq1 | 80 | G | - | - | - | G->A | G->A | G->A |
| comp15218_c0_seq1 | 122 | G | - | - | - | G->A | G->A | G->A |
| comp36709_c0_seq3 | 303 | G | - | - | - | G->A | G->A | G->A |
| comp853895_c0_seq1 | 230 | G | - | - | - | G->A | G->A | G->A |
| comp35760_c1_seq5 | 2968 | G | - | - | - | G->A | G->A | G->A |
| comp17454_c0_seq1 | 930 | G | - | - | - | G->A | G->A | G->A |
| comp18093_c0_seq1 | 149 | G | - | - | - | G->A | G->A | G->A |
| comp10514_c0_seq1 | 188 | G | - | - | - | G->A | G->A | G->A |
| comp33782_c0_seq1 | 689 | G | - | - | - | G->A | G->A | G->A |
| comp1567_c0_seq1 | 283 | G | - | - | - | G->A | G->A | G->A |
| comp34552_c0_seq1 | 73 | G | - | - | - | G->A | G->A | G->A |
| comp13828_c0_seq1 | 932 | G | - | - | - | G->A | G->A | G->A |
| comp18699_c0_seq1 | 775 | G | - | - | - | G->A | G->A | G->A |
| comp17922_c0_seq1 | 47 | G | - | - | - | G->A | G->A | G->A |
| comp35878_c0_seq4 | 1624 | G | - | - | - | G->A | G->A | G->A |
| comp22644_c0_seq1 | 359 | G | - | - | - | G->A | G->A | G->A |
| comp1711137_c0_seq1 | 103 | G | - | - | - | G->A | G->A | G->A |
| comp17051_c0_seq2 | 94 | G | - | - | - | G->A | G->A | G->A |
| comp1250186_c0_seq1 | 133 | G | - | - | - | G->A | G->A | G->A |
| comp1933_c0_seq1 | 383 | G | - | - | - | G->A | G->A | G->A |
| comp29377_c0_seq1 | 501 | G | - | - | - | G->A | G->A | G->A |
| comp10370_c0_seq1 | 128 | G | - | - | - | G->A | G->A | G->A |
| comp32784_c0_seq3 | 815 | G | - | - | - | G->A | G->A | G->A |
| comp27713_c0_seq1 | 940 | G | - | - | - | G->A | G->A | G->A |
| comp36760_c0_seq1 | 1922 | G | - | - | - | G->A | G->A | G->A |
| comp15688_c0_seq1 | 709 | G | - | - | - | G->A | G->A | G->A |
| comp17454_c0_seq1 | 927 | G | - | - | - | G->A | G->A | G->A |
| comp22057_c0_seq1 | 132 | G | - | - | - | G->A | G->A | G->A |
| comp12397_c0_seq1 | 54 | G | - | - | - | G->A | G->A | G->A |
| comp14795_c0_seq1 | 410 | G | - | - | - | G->A | G->A | G->A |
| comp31195_c0_seq1 | 886 | G | - | - | - | G->A | G->A | G->A |
| comp18244_c0_seq1 | 23 | G | - | - | - | G->A | G->A | G->A |
| comp35983_c1_seq8 | 372 | G | - | - | - | G->A | G->A | G->A |
| comp1134459_c0_seq1 | 15 | G | - | - | - | G->A | G->A | G->A |
| comp1717_c0_seq1 | 39 | G | - | - | - | G->A | G->A | G->A |
| comp3815_c0_seq1 | 113 | G | - | - | - | G->A | G->A | G->A |
| comp3815_c0_seq1 | 141 | G | - | - | - | G->A | G->A | G->A |
| comp1313176_c0_seq1 | 293 | G | - | - | - | G->A | G->A | G->A |
| comp18517_c0_seq1 | 498 | G | - | - | - | G->A | G->A | G->A |
| comp3223_c0_seq1 | 264 | G | - | - | - | G->A | G->A | G->A |
| comp14579_c0_seq1 | 235 | G | - | - | - | G->A | G->A | G->A |
| comp36760_c0_seq1 | 1913 | G | - | - | - | G->A | G->A | G->A |
| comp35316_c0_seq1 | 1583 | G | - | - | - | G->A | G->A | G->A |
| comp15758_c0_seq1 | 165 | G | - | - | - | G->A | G->A | G->A |
| comp17206_c0_seq1 | 329 | G | - | - | - | G->A | G->A | G->A |
| comp15692_c0_seq1 | 178 | G | - | - | - | G->A | G->A | G->A |
| comp1009704_c0_seq1 | 19 | G | - | - | - | G->A | G->A | G->A |
| comp17938_c0_seq2 | 90 | G | - | - | - | G->A | G->A | G->A |
| comp27612_c0_seq1 | 234 | G | - | - | - | G->A | G->A | G->A |
| comp15115_c1_seq1 | 497 | G | - | - | - | G->A | G->A | G->A |
| comp10056_c0_seq1 | 320 | G | - | - | - | G->A | G->A | G->A |
| comp16613_c0_seq1 | 133 | G | - | - | - | G->A | G->A | G->A |
| comp18045_c0_seq1 | 218 | G | - | - | - | G->A | G->A | G->A |
| comp1034023_c0_seq1 | 58 | G | - | - | - | G->A | G->A | G->A |
| comp15239_c0_seq1 | 511 | G | - | - | - | G->A | G->A | G->A |
| comp17491_c0_seq1 | 1661 | G | - | - | - | G->A | G->A | G->A |
| comp33624_c0_seq1 | 3103 | G | - | - | - | G->A | G->A | G->A |
| comp13696_c0_seq1 | 46 | G | - | - | - | G->A | G->A | G->A |
| comp36422_c0_seq1 | 6413 | G | - | - | - | G->A | G->A | G->A |
| comp15111_c0_seq1 | 314 | G | - | - | - | G->A | G->A | G->A |
| comp16328_c0_seq1 | 163 | G | - | - | - | G->A | G->A | G->A |
| comp17206_c0_seq1 | 350 | G | - | - | - | G->A | G->A | G->A |
| comp493804_c0_seq1 | 705 | G | - | - | - | G->A | G->A | G->A |
| comp12397_c0_seq1 | 177 | G | - | - | - | G->A | G->A | G->A |
| comp17922_c0_seq1 | 101 | G | - | - | - | G->A | G->A | G->A |
| comp27881_c0_seq1 | 256 | G | - | - | - | G->A | G->A | G->A |
| comp420571_c0_seq1 | 275 | G | - | - | - | G->A | G->A | G->A |
| comp22388_c0_seq1 | 782 | G | - | - | - | G->A | G->A | G->A |
| comp1842_c0_seq1 | 234 | G | - | - | - | G->A | G->A | G->A |
| comp25783_c1_seq1 | 544 | G | - | - | - | G->A | G->A | G->A |
| comp20291_c0_seq1 | 269 | G | - | - | - | G->A | G->A | G->A |
| comp13578_c0_seq2 | 367 | G | - | - | - | G->A | G->A | G->A |
| comp31791_c0_seq3 | 340 | G | - | - | - | G->A | G->A | G->A |
| comp17922_c0_seq1 | 87 | G | - | - | - | G->A | G->A | G->A |
| comp25678_c0_seq1 | 345 | G | - | - | - | G->A | G->A | G->A |
| comp15758_c0_seq1 | 153 | G | - | - | - | G->A | G->A | G->A |
| comp12736_c0_seq1 | 385 | G | - | - | - | G->A | G->A | G->A |
| comp17039_c0_seq1 | 177 | G | - | - | - | G->A | G->A | G->A |
| comp17524_c0_seq1 | 638 | G | - | - | - | G->A | G->A | G->A |
| comp36532_c0_seq7 | 978 | G | - | - | - | G->A | G->A | G->A |
| comp13827_c0_seq1 | 64 | G | - | - | - | G->A | G->A | G->A |
| comp17693_c0_seq1 | 317 | G | - | - | - | G->A | G->A | G->A |
| comp1721_c0_seq1 | 309 | G | - | - | - | G->A | G->A | G->A |
| comp13662_c0_seq1 | 133 | G | - | - | - | G->A | G->A | G->A |
| comp17959_c0_seq1 | 439 | G | - | - | - | G->A | G->A | G->A |
| comp17796_c0_seq1 | 22 | G | - | - | - | G->A | G->A | G->A |
| comp1043421_c0_seq1 | 252 | G | - | - | - | G->A | G->A | G->A |
| comp1090_c0_seq1 | 311 | G | - | - | - | G->A | G->A | G->A |
| comp21895_c0_seq1 | 381 | G | - | - | - | G->A | G->A | G->A |
| comp829777_c0_seq1 | 38 | G | - | - | - | G->A | G->A | G->A |
| comp925327_c0_seq1 | 209 | G | - | - | - | G->A | G->A | G->A |
| comp36049_c0_seq1 | 1036 | G | - | - | - | G->A | G->A | G->A |
| comp35161_c0_seq1 | 2473 | G | - | - | - | G->A | G->A | G->A |
| comp36005_c0_seq1 | 184 | G | - | - | - | G->A | G->A | G->A |
| comp36005_c0_seq1 | 302 | G | - | - | - | G->A | G->A | G->A |
| comp11447_c0_seq1 | 790 | G | - | - | - | G->A | G->A | G->A |
| comp185892_c0_seq1 | 175 | G | - | - | - | G->A | G->A | G->A |
| comp31726_c0_seq1 | 347 | G | - | - | - | G->A | G->A | G->A |
| comp21988_c0_seq1 | 551 | G | - | - | - | G->A | G->A | G->A |
| comp11447_c0_seq1 | 781 | G | - | - | - | G->A | G->A | G->A |
| comp10394_c0_seq1 | 329 | G | - | - | - | G->A | G->A | G->A |
| comp144319_c0_seq1 | 315 | G | - | - | - | G->A | G->A | G->A |
| comp1260725_c0_seq1 | 102 | G | - | - | - | G->A | G->A | G->A |
| comp14021_c0_seq1 | 1385 | G | - | - | - | G->A | G->A | G->A |
| comp120387_c0_seq1 | 318 | G | - | - | - | G->A | G->A | G->A |
| comp13647_c1_seq1 | 154 | G | - | - | - | G->A | G->A | G->A |
| comp15636_c0_seq1 | 208 | G | - | - | - | G->A | G->A | G->A |
| comp34147_c0_seq1 | 841 | G | - | - | - | G->A | G->A | G->A |
| comp4664_c0_seq1 | 136 | G | - | - | - | G->A | G->A | G->A |
| comp27260_c2_seq1 | 686 | G | - | - | - | G->A | G->A | G->A |
| comp11151_c0_seq1 | 392 | G | - | - | - | G->A | G->A | G->A |
| comp1037986_c0_seq1 | 419 | G | - | - | - | G->A | G->A | G->A |
| comp6030_c0_seq1 | 62 | G | - | - | - | G->A | G->A | G->A |
| comp11151_c0_seq1 | 377 | G | - | - | - | G->A | G->A | G->A |
| comp11151_c0_seq1 | 401 | G | - | - | - | G->A | G->A | G->A |
| comp15796_c0_seq1 | 976 | G | - | - | - | G->A | G->A | G->A |
| comp18689_c0_seq1 | 375 | G | - | - | - | G->A | G->A | G->A |
| comp34161_c1_seq2 | 1317 | G | - | - | - | G->A | G->A | G->A |
| comp9596_c0_seq1 | 33 | G | - | - | - | G->A | G->A | G->A |
| comp180856_c0_seq1 | 247 | G | - | - | - | G->A | G->A | G->A |
| comp180856_c0_seq1 | 253 | G | - | - | - | G->A | G->A | G->A |
| comp17314_c0_seq1 | 225 | G | - | - | - | G->A | G->A | G->A |
| comp26137_c0_seq1 | 157 | G | - | - | - | G->A | G->A | G->A |
| comp180856_c0_seq1 | 250 | G | - | - | - | G->A | G->A | G->A |
| comp16299_c0_seq1 | 234 | G | - | - | - | G->A | G->A | G->A |
| comp24072_c0_seq1 | 498 | G | - | - | - | G->A | G->A | G->A |
| comp9504_c0_seq1 | 10 | G | - | - | - | G->A | G->A | G->A |
| comp227090_c0_seq1 | 579 | G | - | - | - | G->A | G->A | G->A |
| comp9504_c0_seq1 | 9 | G | - | - | - | G->A | G->A | G->A |
| comp35050_c2_seq1 | 845 | G | - | - | - | G->A | G->A | G->A |
| comp15928_c0_seq1 | 262 | G | - | - | - | G->A | G->A | G->A |
| comp17796_c0_seq1 | 23 | G | - | - | - | G->A | G->A | G->A |
| comp3182_c0_seq1 | 104 | G | - | - | - | G->C | G->C | G->C |
| comp18431_c0_seq1 | 70 | G | - | - | - | G->C | G->C | G->C |
| comp16969_c0_seq1 | 428 | G | - | - | - | G->C | G->C | G->C |
| comp18336_c3_seq1 | 427 | G | - | - | - | G->C | G->C | G->C |
| comp27412_c0_seq1 | 590 | G | - | - | - | G->C | G->C | G->C |
| comp13993_c0_seq1 | 278 | G | - | - | - | G->C | G->C | G->C |
| comp22722_c0_seq2 | 203 | G | - | - | - | G->C | G->C | G->C |
| comp36142_c0_seq1 | 401 | G | - | - | - | G->C | G->C | G->C |
| comp18037_c0_seq1 | 187 | G | - | - | - | G->C | G->C | G->C |
| comp110_c0_seq1 | 329 | G | - | - | - | G->C | G->C | G->C |
| comp35700_c0_seq2 | 2084 | G | - | - | - | G->C | G->C | G->C |
| comp14651_c0_seq1 | 1378 | G | - | - | - | G->C | G->C | G->C |
| comp13538_c0_seq1 | 17 | G | - | - | - | G->C | G->C | G->C |
| comp17814_c0_seq1 | 142 | G | - | - | - | G->C | G->C | G->C |
| comp17454_c0_seq1 | 897 | G | - | - | - | G->C | G->C | G->C |
| comp35577_c0_seq1 | 107 | G | - | - | - | G->C | G->C | G->C |
| comp7240_c0_seq1 | 15 | G | - | - | - | G->C | G->C | G->C |
| comp17814_c0_seq1 | 197 | G | - | - | - | G->C | G->C | G->C |
| comp17814_c0_seq1 | 192 | G | - | - | - | G->C | G->C | G->C |
| comp16681_c0_seq1 | 312 | G | - | - | - | G->C | G->C | G->C |
| comp13338_c0_seq1 | 710 | G | - | - | - | G->C | G->C | G->C |
| comp16326_c0_seq1 | 265 | G | - | - | - | G->C | G->C | G->C |
| comp17623_c0_seq1 | 198 | G | - | - | - | G->C | G->C | G->C |
| comp33678_c0_seq6 | 19 | G | - | - | - | G->C | G->C | G->C |
| comp13538_c0_seq1 | 14 | G | - | - | - | G->C | G->C | G->C |
| comp17871_c0_seq1 | 467 | G | - | - | - | G->C | G->C | G->C |
| comp18115_c1_seq1 | 147 | G | - | - | - | G->C | G->C | G->C |
| comp18134_c0_seq1 | 308 | G | - | - | - | G->C | G->C | G->C |
| comp35715_c0_seq1 | 731 | G | - | - | - | G->C | G->C | G->C |
| comp11728_c0_seq1 | 12 | G | - | - | - | G->C | G->C | G->C |
| comp11447_c0_seq1 | 800 | G | - | - | - | G->C | G->C | G->C |
| comp14652_c0_seq1 | 1158 | G | - | - | - | G->C | G->C | G->C |
| comp14126_c0_seq1 | 68 | G | - | - | - | G->C | G->C | G->C |
| comp185920_c0_seq1 | 410 | G | - | - | - | G->C | G->C | G->C |
| comp11151_c0_seq1 | 374 | G | - | - | - | G->C | G->C | G->C |
| comp34938_c2_seq1 | 1 | G | - | - | - | G->C | G->C | G->C |
| comp18183_c0_seq1 | 107 | G | - | - | - | G->C | G->C | G->C |
| comp18134_c0_seq1 | 197 | G | - | - | - | G->C | G->C | G->C |
| comp16221_c0_seq2 | 270 | G | - | - | - | G->T | G->T | G->T |
| comp17636_c1_seq1 | 138 | G | - | - | - | G->T | G->T | G->T |
| comp15215_c0_seq1 | 351 | G | - | - | - | G->T | G->T | G->T |
| comp180856_c0_seq1 | 301 | G | - | - | - | G->T | G->T | G->T |
| comp14831_c0_seq1 | 130 | G | - | - | - | G->T | G->T | G->T |
| comp144_c0_seq1 | 525 | G | - | - | - | G->T | G->T | G->T |
| comp829777_c0_seq1 | 26 | G | - | - | - | G->T | G->T | G->T |
| comp18108_c0_seq1 | 84 | G | - | - | - | G->T | G->T | G->T |
| comp11151_c0_seq1 | 367 | G | - | - | - | G->T | G->T | G->T |
| comp11151_c0_seq1 | 371 | G | - | - | - | G->T | G->T | G->T |
| comp12388_c0_seq1 | 68 | G | - | - | - | G->T | G->T | G->T |
| comp11151_c0_seq1 | 349 | G | - | - | - | G->T | G->T | G->T |
| comp33386_c0_seq2 | 59 | G | - | - | - | G->T | G->T | G->T |
| comp10394_c0_seq1 | 413 | G | - | - | - | G->T | G->T | G->T |
| comp34147_c0_seq1 | 822 | G | - | - | - | G->T | G->T | G->T |
| comp34147_c0_seq1 | 848 | G | - | - | - | G->T | G->T | G->T |
| comp10394_c0_seq1 | 323 | G | - | - | - | G->T | G->T | G->T |
| comp10394_c0_seq1 | 338 | G | - | - | - | G->T | G->T | G->T |
| comp14652_c0_seq1 | 1150 | G | - | - | - | G->T | G->T | G->T |
| comp11447_c0_seq1 | 787 | G | - | - | - | G->T | G->T | G->T |
| comp31726_c0_seq1 | 332 | G | - | - | - | G->T | G->T | G->T |
| comp11454_c0_seq1 | 401 | G | - | - | - | G->T | G->T | G->T |
| comp16076_c0_seq1 | 179 | G | - | - | - | G->T | G->T | G->T |
| comp1003065_c0_seq1 | 166 | G | - | - | - | G->T | G->T | G->T |
| comp1131492_c0_seq1 | 122 | G | - | - | - | G->T | G->T | G->T |
| comp10369_c0_seq1 | 195 | G | - | - | - | G->T | G->T | G->T |
| comp15606_c0_seq1 | 107 | G | - | - | - | G->T | G->T | G->T |
| comp31638_c0_seq1 | 12 | G | - | - | - | G->T | G->T | G->T |
| comp10043_c0_seq1 | 63 | G | - | - | - | G->T | G->T | G->T |
| comp12977_c0_seq1 | 455 | G | - | - | - | G->T | G->T | G->T |
| comp12977_c0_seq1 | 718 | G | - | - | - | G->T | G->T | G->T |
| comp15003_c0_seq1 | 323 | G | - | - | - | G->T | G->T | G->T |
| comp829777_c0_seq1 | 70 | G | - | - | - | G->T | G->T | G->T |
| comp689381_c0_seq1 | 1104 | G | - | - | - | G->T | G->T | G->T |
| comp18604_c0_seq1 | 379 | G | - | - | - | G->T | G->T | G->T |
| comp23226_c1_seq1 | 1882 | G | - | - | - | G->T | G->T | G->T |
| comp30682_c1_seq2 | 2261 | G | - | - | - | G->T | G->T | G->T |
| comp1543025_c0_seq1 | 144 | G | - | - | - | G->T | G->T | G->T |
| comp10370_c0_seq1 | 152 | G | - | - | - | G->T | G->T | G->T |
| comp13427_c0_seq1 | 380 | G | - | - | - | G->T | G->T | G->T |
| comp14775_c0_seq1 | 272 | G | - | - | - | G->T | G->T | G->T |
| comp16326_c0_seq1 | 256 | G | - | - | - | G->T | G->T | G->T |
| comp18431_c0_seq1 | 48 | G | - | - | - | G->T | G->T | G->T |
| comp1134459_c0_seq1 | 14 | G | - | - | - | G->T | G->T | G->T |
| comp17814_c0_seq1 | 116 | G | - | - | - | G->T | G->T | G->T |
| comp10370_c0_seq1 | 123 | G | - | - | - | G->T | G->T | G->T |
| comp17814_c0_seq1 | 147 | G | - | - | - | G->T | G->T | G->T |
| comp11_c0_seq1 | 143 | G | - | - | - | G->T | G->T | G->T |
| comp18417_c0_seq1 | 704 | G | - | - | - | G->T | G->T | G->T |
| comp11800_c0_seq1 | 115 | G | - | - | - | G->T | G->T | G->T |
| comp13538_c0_seq1 | 12 | G | - | - | - | G->T | G->T | G->T |
| comp15653_c0_seq1 | 610 | G | - | - | - | G->T | G->T | G->T |
| comp16651_c0_seq1 | 242 | G | - | - | - | G->T | G->T | G->T |
| comp18517_c1_seq1 | 46 | G | - | - | - | G->T | G->T | G->T |
| comp1793_c0_seq1 | 290 | G | - | - | - | G->T | G->T | G->T |
| comp12124_c0_seq1 | 67 | G | - | - | - | G->T | G->T | G->T |
| comp13640_c0_seq1 | 577 | G | - | - | - | G->T | G->T | G->T |
| comp4666_c0_seq1 | 136 | G | - | - | - | G->T | G->T | G->T |
| comp30366_c0_seq3 | 318 | G | - | - | - | G->T | G->T | G->T |
| comp18094_c0_seq1 | 120 | G | - | - | - | G->T | G->T | G->T |
| comp13001_c0_seq1 | 150 | G | - | - | - | G->T | G->T | G->T |
| comp17454_c0_seq1 | 819 | G | - | - | - | G->T | G->T | G->T |
| comp26163_c0_seq1 | 300 | G | - | - | - | G->T | G->T | G->T |
| comp16505_c0_seq1 | 45 | G | - | - | - | G->T | G->T | G->T |
| comp11750_c0_seq1 | 29 | G | - | - | - | G->T | G->T | G->T |
| comp1102_c0_seq1 | 158 | G | - | - | - | G->T | G->T | G->T |
| comp12977_c0_seq1 | 709 | G | - | - | - | G->T | G->T | G->T |
| comp19481_c0_seq1 | 481 | T | - | - | - | T->A | T->A | T->A |
| comp46990_c0_seq1 | 728 | T | - | - | - | T->A | T->A | T->A |
| comp13794_c0_seq1 | 196 | T | - | - | - | T->A | T->A | T->A |
| comp32798_c0_seq1 | 179 | T | - | - | - | T->A | T->A | T->A |
| comp24277_c0_seq1 | 35 | T | - | - | - | T->A | T->A | T->A |
| comp17218_c0_seq1 | 121 | T | - | - | - | T->A | T->A | T->A |
| comp17057_c0_seq1 | 290 | T | - | - | - | T->A | T->A | T->A |
| comp14134_c0_seq1 | 131 | T | - | - | - | T->A | T->A | T->A |
| comp1071765_c0_seq1 | 192 | T | - | - | - | T->A | T->A | T->A |
| comp12880_c0_seq1 | 39 | T | - | - | - | T->A | T->A | T->A |
| comp17814_c0_seq1 | 198 | T | - | - | - | T->A | T->A | T->A |
| comp32220_c0_seq1 | 2025 | T | - | - | - | T->A | T->A | T->A |
| comp16934_c0_seq1 | 103 | T | - | - | - | T->A | T->A | T->A |
| comp17454_c0_seq1 | 960 | T | - | - | - | T->A | T->A | T->A |
| comp13538_c0_seq1 | 7 | T | - | - | - | T->A | T->A | T->A |
| comp34265_c0_seq2 | 202 | T | - | - | - | T->A | T->A | T->A |
| comp17693_c0_seq1 | 318 | T | - | - | - | T->A | T->A | T->A |
| comp10370_c0_seq1 | 167 | T | - | - | - | T->A | T->A | T->A |
| comp17938_c0_seq2 | 91 | T | - | - | - | T->A | T->A | T->A |
| comp17206_c0_seq1 | 342 | T | - | - | - | T->A | T->A | T->A |
| comp17814_c0_seq1 | 200 | T | - | - | - | T->A | T->A | T->A |
| comp17530_c0_seq1 | 240 | T | - | - | - | T->A | T->A | T->A |
| comp13876_c0_seq1 | 259 | T | - | - | - | T->A | T->A | T->A |
| comp13444_c0_seq1 | 296 | T | - | - | - | T->A | T->A | T->A |
| comp16444_c0_seq1 | 24 | T | - | - | - | T->A | T->A | T->A |
| comp16998_c0_seq1 | 168 | T | - | - | - | T->A | T->A | T->A |
| comp3921_c0_seq1 | 98 | T | - | - | - | T->A | T->A | T->A |
| comp829777_c0_seq1 | 32 | T | - | - | - | T->A | T->A | T->A |
| comp17658_c0_seq1 | 81 | T | - | - | - | T->A | T->A | T->A |
| comp18045_c0_seq1 | 450 | T | - | - | - | T->A | T->A | T->A |
| comp17385_c0_seq1 | 12 | T | - | - | - | T->A | T->A | T->A |
| comp1043421_c0_seq1 | 235 | T | - | - | - | T->A | T->A | T->A |
| comp11447_c0_seq1 | 801 | T | - | - | - | T->A | T->A | T->A |
| comp11447_c0_seq1 | 805 | T | - | - | - | T->A | T->A | T->A |
| comp10394_c0_seq1 | 317 | T | - | - | - | T->A | T->A | T->A |
| comp34147_c0_seq1 | 855 | T | - | - | - | T->A | T->A | T->A |
| comp31638_c0_seq1 | 20 | T | - | - | - | T->A | T->A | T->A |
| comp34147_c0_seq1 | 838 | T | - | - | - | T->A | T->A | T->A |
| comp34147_c0_seq1 | 844 | T | - | - | - | T->A | T->A | T->A |
| comp18134_c0_seq1 | 262 | T | - | - | - | T->A | T->A | T->A |
| comp21618_c0_seq1 | 256 | T | - | - | - | T->A | T->A | T->A |
| comp10394_c0_seq1 | 341 | T | - | - | - | T->A | T->A | T->A |
| comp120387_c0_seq1 | 336 | T | - | - | - | T->A | T->A | T->A |
| comp14870_c1_seq1 | 48 | T | - | - | - | T->A | T->A | T->A |
| comp15359_c0_seq1 | 69 | T | - | - | - | T->C | T->C | T->C |
| comp829777_c0_seq1 | 116 | T | - | - | - | T->C | T->C | T->C |
| comp1563_c0_seq1 | 357 | T | - | - | - | T->C | T->C | T->C |
| comp180856_c0_seq1 | 205 | T | - | - | - | T->C | T->C | T->C |
| comp23161_c0_seq2 | 137 | T | - | - | - | T->C | T->C | T->C |
| comp1077695_c0_seq1 | 163 | T | - | - | - | T->C | T->C | T->C |
| comp1563_c0_seq1 | 356 | T | - | - | - | T->C | T->C | T->C |
| comp120387_c0_seq1 | 312 | T | - | - | - | T->C | T->C | T->C |
| comp10394_c0_seq1 | 336 | T | - | - | - | T->C | T->C | T->C |
| comp35939_c0_seq14 | 137 | T | - | - | - | T->C | T->C | T->C |
| comp11414_c0_seq1 | 208 | T | - | - | - | T->C | T->C | T->C |
| comp34147_c0_seq1 | 840 | T | - | - | - | T->C | T->C | T->C |
| comp13287_c0_seq1 | 54 | T | - | - | - | T->C | T->C | T->C |
| comp1260725_c0_seq1 | 94 | T | - | - | - | T->C | T->C | T->C |
| comp23166_c1_seq2 | 72 | T | - | - | - | T->C | T->C | T->C |
| comp12666_c0_seq1 | 123 | T | - | - | - | T->C | T->C | T->C |
| comp36765_c0_seq3 | 143 | T | - | - | - | T->C | T->C | T->C |
| comp16998_c0_seq1 | 233 | T | - | - | - | T->C | T->C | T->C |
| comp15375_c0_seq1 | 690 | T | - | - | - | T->C | T->C | T->C |
| comp14652_c0_seq1 | 1159 | T | - | - | - | T->C | T->C | T->C |
| comp17654_c0_seq1 | 346 | T | - | - | - | T->C | T->C | T->C |
| comp13495_c0_seq1 | 210 | T | - | - | - | T->C | T->C | T->C |
| comp34316_c0_seq1 | 312 | T | - | - | - | T->C | T->C | T->C |
| comp34316_c0_seq1 | 351 | T | - | - | - | T->C | T->C | T->C |
| comp185892_c0_seq1 | 160 | T | - | - | - | T->C | T->C | T->C |
| comp36898_c0_seq1 | 1043 | T | - | - | - | T->C | T->C | T->C |
| comp10599_c0_seq1 | 1339 | T | - | - | - | T->C | T->C | T->C |
| comp126012_c0_seq1 | 422 | T | - | - | - | T->C | T->C | T->C |
| comp126012_c0_seq1 | 420 | T | - | - | - | T->C | T->C | T->C |
| comp185892_c0_seq1 | 181 | T | - | - | - | T->C | T->C | T->C |
| comp1797_c0_seq1 | 186 | T | - | - | - | T->C | T->C | T->C |
| comp35760_c0_seq1 | 12 | T | - | - | - | T->C | T->C | T->C |
| comp21618_c0_seq1 | 240 | T | - | - | - | T->C | T->C | T->C |
| comp35760_c0_seq1 | 13 | T | - | - | - | T->C | T->C | T->C |
| comp917414_c0_seq1 | 210 | T | - | - | - | T->C | T->C | T->C |
| comp33660_c7_seq9 | 1610 | T | - | - | - | T->C | T->C | T->C |
| comp829777_c0_seq1 | 31 | T | - | - | - | T->C | T->C | T->C |
| comp1003065_c0_seq1 | 125 | T | - | - | - | T->C | T->C | T->C |
| comp23656_c0_seq1 | 39 | T | - | - | - | T->C | T->C | T->C |
| comp8966_c1_seq1 | 601 | T | - | - | - | T->C | T->C | T->C |
| comp23812_c0_seq1 | 58 | T | - | - | - | T->C | T->C | T->C |
| comp1131337_c0_seq1 | 249 | T | - | - | - | T->C | T->C | T->C |
| comp16364_c0_seq1 | 202 | T | - | - | - | T->C | T->C | T->C |
| comp12530_c0_seq1 | 392 | T | - | - | - | T->C | T->C | T->C |
| comp15248_c0_seq1 | 549 | T | - | - | - | T->C | T->C | T->C |
| comp25835_c0_seq1 | 251 | T | - | - | - | T->C | T->C | T->C |
| comp568869_c0_seq1 | 206 | T | - | - | - | T->C | T->C | T->C |
| comp24888_c0_seq1 | 292 | T | - | - | - | T->C | T->C | T->C |
| comp21668_c0_seq1 | 148 | T | - | - | - | T->C | T->C | T->C |
| comp16208_c0_seq1 | 151 | T | - | - | - | T->C | T->C | T->C |
| comp22487_c0_seq1 | 1374 | T | - | - | - | T->C | T->C | T->C |
| comp32798_c0_seq1 | 25 | T | - | - | - | T->C | T->C | T->C |
| comp17206_c0_seq1 | 388 | T | - | - | - | T->C | T->C | T->C |
| comp26824_c0_seq1 | 443 | T | - | - | - | T->C | T->C | T->C |
| comp588799_c0_seq1 | 175 | T | - | - | - | T->C | T->C | T->C |
| comp1034023_c0_seq1 | 147 | T | - | - | - | T->C | T->C | T->C |
| comp23814_c0_seq1 | 55 | T | - | - | - | T->C | T->C | T->C |
| comp12496_c0_seq1 | 66 | T | - | - | - | T->C | T->C | T->C |
| comp24492_c1_seq1 | 262 | T | - | - | - | T->C | T->C | T->C |
| comp16538_c0_seq1 | 158 | T | - | - | - | T->C | T->C | T->C |
| comp12930_c0_seq1 | 548 | T | - | - | - | T->C | T->C | T->C |
| comp35161_c0_seq1 | 2404 | T | - | - | - | T->C | T->C | T->C |
| comp18466_c0_seq1 | 59 | T | - | - | - | T->C | T->C | T->C |
| comp13723_c0_seq1 | 134 | T | - | - | - | T->C | T->C | T->C |
| comp16505_c0_seq1 | 90 | T | - | - | - | T->C | T->C | T->C |
| comp15115_c1_seq1 | 440 | T | - | - | - | T->C | T->C | T->C |
| comp1878_c0_seq1 | 410 | T | - | - | - | T->C | T->C | T->C |
| comp4931_c0_seq1 | 543 | T | - | - | - | T->C | T->C | T->C |
| comp1154876_c0_seq1 | 110 | T | - | - | - | T->C | T->C | T->C |
| comp1144648_c0_seq1 | 56 | T | - | - | - | T->C | T->C | T->C |
| comp178_c0_seq1 | 125 | T | - | - | - | T->C | T->C | T->C |
| comp16505_c0_seq1 | 85 | T | - | - | - | T->C | T->C | T->C |
| comp953463_c0_seq1 | 402 | T | - | - | - | T->C | T->C | T->C |
| comp22018_c0_seq2 | 853 | T | - | - | - | T->C | T->C | T->C |
| comp35715_c0_seq1 | 1586 | T | - | - | - | T->C | T->C | T->C |
| comp17454_c0_seq1 | 909 | T | - | - | - | T->C | T->C | T->C |
| comp17454_c0_seq1 | 926 | T | - | - | - | T->C | T->C | T->C |
| comp14542_c0_seq1 | 576 | T | - | - | - | T->C | T->C | T->C |
| comp34772_c0_seq2 | 1649 | T | - | - | - | T->C | T->C | T->C |
| comp2683_c0_seq1 | 133 | T | - | - | - | T->C | T->C | T->C |
| comp17906_c0_seq2 | 185 | T | - | - | - | T->C | T->C | T->C |
| comp17814_c0_seq1 | 135 | T | - | - | - | T->C | T->C | T->C |
| comp13538_c0_seq1 | 15 | T | - | - | - | T->C | T->C | T->C |
| comp10370_c0_seq1 | 108 | T | - | - | - | T->C | T->C | T->C |
| comp35585_c0_seq1 | 1352 | T | - | - | - | T->C | T->C | T->C |
| comp14_c0_seq1 | 205 | T | - | - | - | T->C | T->C | T->C |
| comp16347_c0_seq1 | 396 | T | - | - | - | T->C | T->C | T->C |
| comp32398_c0_seq2 | 281 | T | - | - | - | T->C | T->C | T->C |
| comp31749_c0_seq2 | 203 | T | - | - | - | T->C | T->C | T->C |
| comp13627_c0_seq1 | 132 | T | - | - | - | T->C | T->C | T->C |
| comp12959_c0_seq1 | 227 | T | - | - | - | T->C | T->C | T->C |
| comp36917_c0_seq9 | 32 | T | - | - | - | T->C | T->C | T->C |
| comp18340_c0_seq1 | 133 | T | - | - | - | T->C | T->C | T->C |
| comp15433_c0_seq1 | 137 | T | - | - | - | T->C | T->C | T->C |
| comp1977_c0_seq1 | 108 | T | - | - | - | T->C | T->C | T->C |
| comp17057_c0_seq1 | 284 | T | - | - | - | T->C | T->C | T->C |
| comp30674_c0_seq1 | 446 | T | - | - | - | T->C | T->C | T->C |
| comp36415_c0_seq3 | 241 | T | - | - | - | T->C | T->C | T->C |
| comp1790_c0_seq1 | 94 | T | - | - | - | T->C | T->C | T->C |
| comp13627_c0_seq1 | 148 | T | - | - | - | T->C | T->C | T->C |
| comp21762_c0_seq1 | 1169 | T | - | - | - | T->C | T->C | T->C |
| comp1132626_c0_seq1 | 285 | T | - | - | - | T->C | T->C | T->C |
| comp23166_c1_seq2 | 45 | T | - | - | - | T->C | T->C | T->C |
| comp16208_c0_seq1 | 364 | T | - | - | - | T->C | T->C | T->C |
| comp10370_c0_seq1 | 158 | T | - | - | - | T->C | T->C | T->C |
| comp36299_c0_seq1 | 57 | T | - | - | - | T->C | T->C | T->C |
| comp21559_c0_seq2 | 672 | T | - | - | - | T->C | T->C | T->C |
| comp377331_c0_seq1 | 89 | T | - | - | - | T->C | T->C | T->C |
| comp20177_c0_seq1 | 187 | T | - | - | - | T->C | T->C | T->C |
| comp16505_c0_seq1 | 75 | T | - | - | - | T->C | T->C | T->C |
| comp29240_c0_seq1 | 555 | T | - | - | - | T->C | T->C | T->C |
| comp27713_c0_seq1 | 549 | T | - | - | - | T->C | T->C | T->C |
| comp17176_c0_seq1 | 620 | T | - | - | - | T->C | T->C | T->C |
| comp10370_c0_seq1 | 141 | T | - | - | - | T->C | T->C | T->C |
| comp16505_c0_seq1 | 72 | T | - | - | - | T->C | T->C | T->C |
| comp13627_c0_seq1 | 145 | T | - | - | - | T->C | T->C | T->C |
| comp32451_c0_seq2 | 252 | T | - | - | - | T->C | T->C | T->C |
| comp15218_c0_seq1 | 400 | T | - | - | - | T->C | T->C | T->C |
| comp13918_c0_seq1 | 666 | T | - | - | - | T->C | T->C | T->C |
| comp17483_c0_seq1 | 210 | T | - | - | - | T->C | T->C | T->C |
| comp22855_c0_seq1 | 322 | T | - | - | - | T->C | T->C | T->C |
| comp26147_c0_seq1 | 641 | T | - | - | - | T->C | T->C | T->C |
| comp18700_c0_seq1 | 259 | T | - | - | - | T->C | T->C | T->C |
| comp29846_c1_seq2 | 159 | T | - | - | - | T->C | T->C | T->C |
| comp17257_c0_seq1 | 1499 | T | - | - | - | T->C | T->C | T->C |
| comp23946_c0_seq1 | 1507 | T | - | - | - | T->C | T->C | T->C |
| comp12397_c0_seq1 | 43 | T | - | - | - | T->C | T->C | T->C |
| comp23226_c1_seq1 | 603 | T | - | - | - | T->C | T->C | T->C |
| comp26121_c0_seq1 | 497 | T | - | - | - | T->C | T->C | T->C |
| comp15486_c0_seq1 | 139 | T | - | - | - | T->C | T->C | T->C |
| comp17526_c0_seq1 | 706 | T | - | - | - | T->C | T->C | T->C |
| comp18272_c1_seq1 | 438 | T | - | - | - | T->C | T->C | T->C |
| comp36800_c0_seq2 | 183 | T | - | - | - | T->C | T->C | T->C |
| comp11817_c0_seq2 | 559 | T | - | - | - | T->C | T->C | T->C |
| comp17051_c0_seq2 | 104 | T | - | - | - | T->C | T->C | T->C |
| comp12977_c0_seq1 | 456 | T | - | - | - | T->C | T->C | T->C |
| comp9803_c0_seq1 | 2663 | T | - | - | - | T->C | T->C | T->C |
| comp15261_c0_seq2 | 39 | T | - | - | - | T->G | T->G | T->G |
| comp35760_c0_seq1 | 9 | T | - | - | - | T->G | T->G | T->G |
| comp34938_c2_seq1 | 3 | T | - | - | - | T->G | T->G | T->G |
| comp180856_c0_seq1 | 238 | T | - | - | - | T->G | T->G | T->G |
| comp15870_c0_seq1 | 182 | T | - | - | - | T->G | T->G | T->G |
| comp16551_c0_seq1 | 668 | T | - | - | - | T->G | T->G | T->G |
| comp180856_c0_seq1 | 265 | T | - | - | - | T->G | T->G | T->G |
| comp180856_c0_seq1 | 298 | T | - | - | - | T->G | T->G | T->G |
| comp15606_c0_seq1 | 106 | T | - | - | - | T->G | T->G | T->G |
| comp11151_c0_seq1 | 395 | T | - | - | - | T->G | T->G | T->G |
| comp34147_c0_seq1 | 823 | T | - | - | - | T->G | T->G | T->G |
| comp42420_c0_seq1 | 239 | T | - | - | - | T->G | T->G | T->G |
| comp23166_c1_seq2 | 61 | T | - | - | - | T->G | T->G | T->G |
| comp18469_c0_seq1 | 269 | T | - | - | - | T->G | T->G | T->G |
| comp14652_c0_seq1 | 1217 | T | - | - | - | T->G | T->G | T->G |
| comp24888_c0_seq1 | 198 | T | - | - | - | T->G | T->G | T->G |
| comp15704_c1_seq1 | 900 | T | - | - | - | T->G | T->G | T->G |
| comp16074_c0_seq1 | 1228 | T | - | - | - | T->G | T->G | T->G |
| comp17425_c1_seq1 | 129 | T | - | - | - | T->G | T->G | T->G |
| comp16102_c0_seq1 | 729 | T | - | - | - | T->G | T->G | T->G |
| comp672_c0_seq1 | 672 | T | - | - | - | T->G | T->G | T->G |
| comp23258_c0_seq1 | 19 | T | - | - | - | T->G | T->G | T->G |
| comp35059_c0_seq1 | 1234 | T | - | - | - | T->G | T->G | T->G |
| comp31286_c0_seq1 | 811 | T | - | - | - | T->G | T->G | T->G |
| comp1247468_c0_seq1 | 245 | T | - | - | - | T->G | T->G | T->G |
| comp22147_c0_seq1 | 1666 | T | - | - | - | T->G | T->G | T->G |
| comp17613_c0_seq1 | 370 | T | - | - | - | T->G | T->G | T->G |
| comp22873_c2_seq1 | 406 | T | - | - | - | T->G | T->G | T->G |
| comp15115_c1_seq1 | 458 | T | - | - | - | T->G | T->G | T->G |
| comp22468_c0_seq1 | 1404 | T | - | - | - | T->G | T->G | T->G |
| comp13338_c0_seq1 | 711 | T | - | - | - | T->G | T->G | T->G |
| comp1213001_c0_seq1 | 161 | T | - | - | - | T->G | T->G | T->G |
| comp28618_c1_seq1 | 160 | T | - | - | - | T->G | T->G | T->G |
| comp13319_c0_seq1 | 392 | T | - | - | - | T->G | T->G | T->G |
| comp15727_c0_seq1 | 1401 | T | - | - | - | T->G | T->G | T->G |
| comp1202092_c0_seq1 | 93 | T | - | - | - | T->G | T->G | T->G |
| comp17191_c0_seq1 | 400 | T | - | - | - | T->G | T->G | T->G |
| comp18134_c0_seq1 | 219 | T | - | - | - | T->G | T->G | T->G |
| comp15486_c0_seq1 | 58 | T | - | - | - | T->G | T->G | T->G |
| comp13627_c0_seq1 | 151 | T | - | - | - | T->G | T->G | T->G |
| comp15278_c0_seq1 | 543 | T | - | - | - | T->G | T->G | T->G |
| comp15806_c0_seq1 | 295 | T | - | - | - | T->G | T->G | T->G |
| comp33258_c0_seq3 | 132 | T | - | - | - | T->G | T->G | T->G |
| comp35167_c0_seq2 | 770 | T | - | - | - | T->G | T->G | T->G |
| comp16812_c0_seq1 | 938 | T | - | - | - | T->G | T->G | T->G |
| comp12530_c0_seq1 | 296 | T | - | - | - | T->G | T->G | T->G |
| comp11_c0_seq1 | 322 | T | - | - | - | T->G | T->G | T->G |
| comp22040_c0_seq2 | 111 | T | - | - | - | T->G | T->G | T->G |
| comp12880_c0_seq1 | 110 | T | - | - | - | T->G | T->G | T->G |
| comp17454_c0_seq1 | 882 | T | - | - | - | T->G | T->G | T->G |
| comp13486_c0_seq1 | 373 | T | - | - | - | T->G | T->G | T->G |
| comp35904_c0_seq12 | 1253 | A | A->T | A->T | A->T | - | - | - |
| comp14828_c0_seq1 | 752 | A | A->T | A->T | A->T | - | - | - |
| comp11699_c0_seq1 | 1052 | A | A->T | A->T | A->T | - | - | - |
| comp12722_c0_seq1 | 1395 | A | A->T | A->T | A->T | - | - | - |
| comp33527_c1_seq1 | 770 | A | A->T | A->T | A->T | - | - | - |
| comp17321_c0_seq1 | 218 | A | A->T | A->T | A->T | - | - | - |
| comp12480_c0_seq1 | 33 | A | A->T | A->T | A->T | - | - | - |
| comp34553_c1_seq9 | 3522 | A | A->T | A->T | A->T | - | - | - |
| comp25005_c0_seq1 | 662 | A | A->T | A->T | A->T | - | - | - |
| comp13614_c0_seq1 | 168 | A | A->T | A->T | A->T | - | - | - |
| comp15294_c0_seq1 | 66 | A | A->T | A->T | A->T | - | - | - |
| comp11699_c0_seq1 | 1098 | A | A->T | A->T | A->T | - | - | - |
| comp11683_c0_seq1 | 506 | A | A->T | A->T | A->T | - | - | - |
| comp15658_c0_seq1 | 291 | A | A->T | A->T | A->T | - | - | - |
| comp24389_c0_seq1 | 1828 | A | A->T | A->T | A->T | - | - | - |
| comp1304818_c0_seq1 | 72 | A | A->T | A->T | A->T | - | - | - |
| comp694013_c0_seq1 | 166 | A | A->T | A->T | A->T | - | - | - |
| comp1129_c0_seq1 | 1079 | A | A->T | A->T | A->T | - | - | - |
| comp31265_c1_seq2 | 718 | A | A->T | A->T | A->T | - | - | - |
| comp22827_c0_seq1 | 2671 | A | A->T | A->T | A->T | - | - | - |
| comp22827_c0_seq1 | 2672 | A | A->T | A->T | A->T | - | - | - |
| comp8051_c0_seq1 | 124 | A | A->T | A->T | A->T | - | - | - |
| comp16103_c1_seq1 | 211 | A | A->T | A->T | A->T | - | - | - |
| comp34587_c1_seq5 | 458 | A | A->T | A->T | A->T | - | - | - |
| comp30993_c0_seq1 | 356 | A | A->T | A->T | A->T | - | - | - |
| comp17443_c0_seq1 | 294 | A | A->T | A->T | A->T | - | - | - |
| comp34026_c0_seq1 | 29 | A | A->T | A->T | A->T | - | - | - |
| comp36391_c1_seq7 | 2 | A | A->T | A->T | A->T | - | - | - |
| comp13259_c0_seq1 | 142 | A | A->T | A->T | A->T | - | - | - |
| comp16103_c1_seq1 | 181 | A | A->T | A->T | A->T | - | - | - |
| comp120974_c0_seq1 | 9 | A | A->T | A->T | A->T | - | - | - |
| comp12105_c0_seq1 | 281 | A | A->T | A->T | A->T | - | - | - |
| comp836728_c0_seq1 | 305 | A | A->T | A->T | A->T | - | - | - |
| comp16006_c1_seq1 | 114 | A | A->T | A->T | A->T | - | - | - |
| comp16918_c0_seq1 | 106 | A | A->T | A->T | A->T | - | - | - |
| comp14817_c0_seq1 | 409 | A | A->T | A->T | A->T | - | - | - |
| comp10400_c0_seq1 | 502 | A | A->T | A->T | A->T | - | - | - |
| comp15869_c0_seq1 | 328 | A | A->T | A->T | A->T | - | - | - |
| comp16918_c0_seq1 | 112 | A | A->T | A->T | A->T | - | - | - |
| comp1558_c0_seq1 | 24 | A | A->T | A->T | A->T | - | - | - |
| comp1083751_c0_seq1 | 237 | A | A->T | A->T | A->T | - | - | - |
| comp1558_c0_seq1 | 16 | A | A->T | A->T | A->T | - | - | - |
| comp34742_c0_seq2 | 340 | A | A->T | A->T | A->T | - | - | - |
| comp16632_c0_seq1 | 426 | A | A->T | A->T | A->T | - | - | - |
| comp35726_c0_seq1 | 1042 | A | A->T | A->T | A->T | - | - | - |
| comp12298_c0_seq1 | 196 | A | A->T | A->T | A->T | - | - | - |
| comp17423_c1_seq1 | 53 | A | A->T | A->T | A->T | - | - | - |
| comp12789_c0_seq1 | 2 | A | A->T | A->T | A->T | - | - | - |
| comp12230_c0_seq1 | 391 | A | A->T | A->T | A->T | - | - | - |
| comp12709_c0_seq3 | 132 | A | A->T | A->T | A->T | - | - | - |
| comp28710_c0_seq1 | 29 | A | A->T | A->T | A->T | - | - | - |
| comp36842_c0_seq3 | 1713 | A | A->T | A->T | A->T | - | - | - |
| comp28710_c0_seq1 | 35 | A | A->T | A->T | A->T | - | - | - |
| comp26695_c0_seq1 | 7 | A | A->T | A->T | A->T | - | - | - |
| comp36746_c0_seq5 | 110 | A | A->T | A->T | A->T | - | - | - |
| comp36732_c1_seq8 | 111 | A | A->T | A->T | A->T | - | - | - |
| comp12210_c0_seq1 | 528 | A | A->T | A->T | A->T | - | - | - |
| comp36828_c0_seq2 | 2012 | A | A->T | A->T | A->T | - | - | - |
| comp30024_c0_seq1 | 14 | A | A->T | A->T | A->T | - | - | - |
| comp3298_c0_seq1 | 118 | A | A->T | A->T | A->T | - | - | - |
| comp12128_c0_seq2 | 205 | A | A->T | A->T | A->T | - | - | - |
| comp34008_c2_seq6 | 38 | A | A->T | A->T | A->T | - | - | - |
| comp34219_c0_seq12 | 2215 | A | A->G | A->G | A->G | - | - | - |
| comp24972_c0_seq1 | 2574 | A | A->G | A->G | A->G | - | - | - |
| comp15623_c0_seq1 | 34 | A | A->G | A->G | A->G | - | - | - |
| comp17279_c0_seq1 | 30 | A | A->G | A->G | A->G | - | - | - |
| comp33527_c1_seq1 | 2070 | A | A->G | A->G | A->G | - | - | - |
| comp11699_c0_seq1 | 864 | A | A->G | A->G | A->G | - | - | - |
| comp12275_c0_seq1 | 209 | A | A->G | A->G | A->G | - | - | - |
| comp2051_c0_seq1 | 284 | A | A->G | A->G | A->G | - | - | - |
| comp9125_c0_seq1 | 225 | A | A->G | A->G | A->G | - | - | - |
| comp16918_c0_seq1 | 78 | A | A->G | A->G | A->G | - | - | - |
| comp10933_c0_seq1 | 133 | A | A->G | A->G | A->G | - | - | - |
| comp21030_c0_seq1 | 2044 | A | A->G | A->G | A->G | - | - | - |
| comp36476_c0_seq2 | 713 | A | A->G | A->G | A->G | - | - | - |
| comp15137_c0_seq1 | 416 | A | A->G | A->G | A->G | - | - | - |
| comp17649_c0_seq1 | 180 | A | A->G | A->G | A->G | - | - | - |
| comp33231_c0_seq1 | 2986 | A | A->G | A->G | A->G | - | - | - |
| comp11699_c0_seq1 | 913 | A | A->G | A->G | A->G | - | - | - |
| comp14124_c0_seq1 | 454 | A | A->G | A->G | A->G | - | - | - |
| comp10933_c0_seq1 | 121 | A | A->G | A->G | A->G | - | - | - |
| comp11699_c0_seq1 | 930 | A | A->G | A->G | A->G | - | - | - |
| comp29502_c0_seq1 | 2334 | A | A->G | A->G | A->G | - | - | - |
| comp11699_c0_seq1 | 1095 | A | A->G | A->G | A->G | - | - | - |
| comp17720_c0_seq1 | 1083 | A | A->G | A->G | A->G | - | - | - |
| comp34073_c0_seq8 | 78 | A | A->G | A->G | A->G | - | - | - |
| comp15137_c0_seq1 | 366 | A | A->G | A->G | A->G | - | - | - |
| comp1338_c0_seq1 | 127 | A | A->G | A->G | A->G | - | - | - |
| comp20696_c0_seq1 | 500 | A | A->G | A->G | A->G | - | - | - |
| comp17443_c0_seq1 | 281 | A | A->G | A->G | A->G | - | - | - |
| comp35889_c0_seq2 | 29 | A | A->G | A->G | A->G | - | - | - |
| comp9125_c0_seq1 | 243 | A | A->G | A->G | A->G | - | - | - |
| comp1338_c0_seq1 | 142 | A | A->G | A->G | A->G | - | - | - |
| comp35463_c1_seq16 | 871 | A | A->G | A->G | A->G | - | - | - |
| comp12697_c0_seq1 | 308 | A | A->G | A->G | A->G | - | - | - |
| comp13877_c0_seq1 | 648 | A | A->G | A->G | A->G | - | - | - |
| comp25887_c1_seq1 | 2776 | A | A->G | A->G | A->G | - | - | - |
| comp34358_c0_seq1 | 76 | A | A->G | A->G | A->G | - | - | - |
| comp34178_c0_seq1 | 433 | A | A->G | A->G | A->G | - | - | - |
| comp17508_c0_seq1 | 1075 | A | A->G | A->G | A->G | - | - | - |
| comp16966_c0_seq1 | 691 | A | A->G | A->G | A->G | - | - | - |
| comp10933_c0_seq1 | 154 | A | A->G | A->G | A->G | - | - | - |
| comp17766_c0_seq1 | 267 | A | A->G | A->G | A->G | - | - | - |
| comp17536_c0_seq1 | 142 | A | A->G | A->G | A->G | - | - | - |
| comp12007_c0_seq1 | 93 | A | A->G | A->G | A->G | - | - | - |
| comp25000_c0_seq1 | 435 | A | A->G | A->G | A->G | - | - | - |
| comp14437_c0_seq1 | 777 | A | A->G | A->G | A->G | - | - | - |
| comp15644_c0_seq1 | 257 | A | A->G | A->G | A->G | - | - | - |
| comp16126_c0_seq1 | 100 | A | A->G | A->G | A->G | - | - | - |
| comp16126_c0_seq1 | 108 | A | A->G | A->G | A->G | - | - | - |
| comp991728_c0_seq1 | 108 | A | A->G | A->G | A->G | - | - | - |
| comp28522_c0_seq1 | 946 | A | A->G | A->G | A->G | - | - | - |
| comp14507_c1_seq1 | 200 | A | A->G | A->G | A->G | - | - | - |
| comp34288_c0_seq1 | 604 | A | A->G | A->G | A->G | - | - | - |
| comp33892_c0_seq2 | 1440 | A | A->G | A->G | A->G | - | - | - |
| comp31405_c1_seq5 | 50 | A | A->G | A->G | A->G | - | - | - |
| comp16693_c0_seq1 | 369 | A | A->G | A->G | A->G | - | - | - |
| comp36280_c1_seq3 | 1247 | A | A->G | A->G | A->G | - | - | - |
| comp33165_c0_seq1 | 145 | A | A->G | A->G | A->G | - | - | - |
| comp34318_c0_seq2 | 1981 | A | A->G | A->G | A->G | - | - | - |
| comp32437_c2_seq1 | 1695 | A | A->G | A->G | A->G | - | - | - |
| comp35334_c1_seq3 | 6322 | A | A->G | A->G | A->G | - | - | - |
| comp14508_c0_seq1 | 1041 | A | A->G | A->G | A->G | - | - | - |
| comp11506_c0_seq1 | 722 | A | A->G | A->G | A->G | - | - | - |
| comp11699_c0_seq1 | 966 | A | A->G | A->G | A->G | - | - | - |
| comp20581_c3_seq1 | 252 | A | A->G | A->G | A->G | - | - | - |
| comp11699_c0_seq1 | 849 | A | A->G | A->G | A->G | - | - | - |
| comp15623_c0_seq1 | 40 | A | A->G | A->G | A->G | - | - | - |
| comp14029_c0_seq1 | 315 | A | A->G | A->G | A->G | - | - | - |
| comp16096_c0_seq1 | 183 | A | A->G | A->G | A->G | - | - | - |
| comp25568_c0_seq1 | 2522 | A | A->G | A->G | A->G | - | - | - |
| comp30622_c1_seq1 | 63 | A | A->G | A->G | A->G | - | - | - |
| comp762199_c0_seq1 | 118 | A | A->G | A->G | A->G | - | - | - |
| comp27612_c0_seq1 | 207 | A | A->G | A->G | A->G | - | - | - |
| comp28710_c0_seq1 | 31 | A | A->G | A->G | A->G | - | - | - |
| comp30735_c0_seq1 | 2866 | A | A->G | A->G | A->G | - | - | - |
| comp17536_c0_seq1 | 199 | A | A->G | A->G | A->G | - | - | - |
| comp17536_c0_seq1 | 193 | A | A->G | A->G | A->G | - | - | - |
| comp11699_c0_seq1 | 1137 | A | A->G | A->G | A->G | - | - | - |
| comp12095_c0_seq1 | 1702 | A | A->G | A->G | A->G | - | - | - |
| comp25003_c0_seq1 | 894 | A | A->G | A->G | A->G | - | - | - |
| comp22220_c0_seq1 | 150 | A | A->G | A->G | A->G | - | - | - |
| comp30685_c0_seq2 | 1344 | A | A->G | A->G | A->G | - | - | - |
| comp17536_c0_seq1 | 139 | A | A->G | A->G | A->G | - | - | - |
| comp16433_c0_seq1 | 282 | A | A->G | A->G | A->G | - | - | - |
| comp22341_c0_seq1 | 104 | A | A->G | A->G | A->G | - | - | - |
| comp18082_c0_seq1 | 197 | A | A->G | A->G | A->G | - | - | - |
| comp35726_c0_seq1 | 953 | A | A->G | A->G | A->G | - | - | - |
| comp53_c1_seq1 | 169 | A | A->G | A->G | A->G | - | - | - |
| comp10933_c0_seq1 | 106 | A | A->G | A->G | A->G | - | - | - |
| comp25578_c0_seq4 | 202 | A | A->G | A->G | A->G | - | - | - |
| comp12194_c0_seq1 | 175 | A | A->G | A->G | A->G | - | - | - |
| comp23384_c0_seq1 | 63 | A | A->G | A->G | A->G | - | - | - |
| comp35658_c3_seq1 | 249 | A | A->G | A->G | A->G | - | - | - |
| comp15869_c0_seq1 | 396 | A | A->G | A->G | A->G | - | - | - |
| comp34236_c1_seq1 | 2207 | A | A->G | A->G | A->G | - | - | - |
| comp15083_c0_seq1 | 78 | A | A->G | A->G | A->G | - | - | - |
| comp17720_c0_seq1 | 1075 | A | A->G | A->G | A->G | - | - | - |
| comp25535_c0_seq1 | 15 | A | A->G | A->G | A->G | - | - | - |
| comp26911_c1_seq1 | 937 | A | A->G | A->G | A->G | - | - | - |
| comp12841_c0_seq1 | 858 | A | A->G | A->G | A->G | - | - | - |
| comp1127129_c0_seq1 | 244 | A | A->G | A->G | A->G | - | - | - |
| comp30975_c0_seq3 | 2292 | A | A->G | A->G | A->G | - | - | - |
| comp23557_c0_seq2 | 465 | A | A->G | A->G | A->G | - | - | - |
| comp12194_c0_seq1 | 165 | A | A->G | A->G | A->G | - | - | - |
| comp896261_c0_seq1 | 740 | A | A->G | A->G | A->G | - | - | - |
| comp33582_c1_seq1 | 117 | A | A->G | A->G | A->G | - | - | - |
| comp30559_c0_seq1 | 47 | A | A->G | A->G | A->G | - | - | - |
| comp1242091_c0_seq1 | 268 | A | A->G | A->G | A->G | - | - | - |
| comp25164_c1_seq1 | 2044 | A | A->G | A->G | A->G | - | - | - |
| comp30195_c0_seq4 | 40 | A | A->G | A->G | A->G | - | - | - |
| comp27107_c0_seq1 | 623 | A | A->G | A->G | A->G | - | - | - |
| comp546_c0_seq2 | 319 | A | A->G | A->G | A->G | - | - | - |
| comp28931_c0_seq1 | 1197 | A | A->G | A->G | A->G | - | - | - |
| comp15137_c0_seq1 | 336 | A | A->G | A->G | A->G | - | - | - |
| comp17543_c0_seq1 | 153 | A | A->G | A->G | A->G | - | - | - |
| comp30588_c0_seq1 | 1858 | A | A->G | A->G | A->G | - | - | - |
| comp36732_c1_seq8 | 95 | A | A->G | A->G | A->G | - | - | - |
| comp16006_c1_seq1 | 150 | A | A->G | A->G | A->G | - | - | - |
| comp36788_c0_seq2 | 291 | A | A->G | A->G | A->G | - | - | - |
| comp18091_c0_seq1 | 109 | A | A->G | A->G | A->G | - | - | - |
| comp2601_c0_seq1 | 209 | A | A->G | A->G | A->G | - | - | - |
| comp11699_c0_seq1 | 339 | A | A->G | A->G | A->G | - | - | - |
| comp29960_c0_seq1 | 101 | A | A->G | A->G | A->G | - | - | - |
| comp17422_c0_seq1 | 412 | A | A->G | A->G | A->G | - | - | - |
| comp15623_c0_seq1 | 53 | A | A->G | A->G | A->G | - | - | - |
| comp36842_c0_seq3 | 2417 | A | A->G | A->G | A->G | - | - | - |
| comp11699_c0_seq1 | 994 | A | A->G | A->G | A->G | - | - | - |
| comp11773_c0_seq1 | 8 | A | A->G | A->G | A->G | - | - | - |
| comp12194_c0_seq1 | 185 | A | A->G | A->G | A->G | - | - | - |
| comp10933_c0_seq1 | 118 | A | A->G | A->G | A->G | - | - | - |
| comp16006_c1_seq1 | 115 | A | A->G | A->G | A->G | - | - | - |
| comp17508_c0_seq1 | 306 | A | A->G | A->G | A->G | - | - | - |
| comp15026_c0_seq1 | 1743 | A | A->G | A->G | A->G | - | - | - |
| comp16918_c0_seq1 | 151 | A | A->G | A->G | A->G | - | - | - |
| comp23652_c0_seq1 | 151 | A | A->G | A->G | A->G | - | - | - |
| comp10933_c0_seq1 | 137 | A | A->G | A->G | A->G | - | - | - |
| comp1338_c0_seq1 | 130 | A | A->G | A->G | A->G | - | - | - |
| comp794134_c0_seq1 | 258 | A | A->G | A->G | A->G | - | - | - |
| comp12460_c0_seq1 | 138 | A | A->G | A->G | A->G | - | - | - |
| comp16658_c1_seq1 | 247 | A | A->G | A->G | A->G | - | - | - |
| comp16006_c1_seq1 | 118 | A | A->G | A->G | A->G | - | - | - |
| comp13288_c0_seq1 | 1714 | A | A->G | A->G | A->G | - | - | - |
| comp16006_c1_seq1 | 45 | A | A->G | A->G | A->G | - | - | - |
| comp24479_c3_seq1 | 1956 | A | A->G | A->G | A->G | - | - | - |
| comp16006_c1_seq1 | 119 | A | A->G | A->G | A->G | - | - | - |
| comp11699_c0_seq1 | 924 | A | A->G | A->G | A->G | - | - | - |
| comp15137_c0_seq1 | 435 | A | A->G | A->G | A->G | - | - | - |
| comp16006_c1_seq1 | 59 | A | A->G | A->G | A->G | - | - | - |
| comp36587_c0_seq4 | 542 | A | A->G | A->G | A->G | - | - | - |
| comp36587_c0_seq4 | 544 | A | A->G | A->G | A->G | - | - | - |
| comp36587_c0_seq4 | 553 | A | A->G | A->G | A->G | - | - | - |
| comp10117_c0_seq1 | 45 | A | A->G | A->G | A->G | - | - | - |
| comp12751_c0_seq1 | 24 | A | A->G | A->G | A->G | - | - | - |
| comp1740_c0_seq1 | 255 | A | A->G | A->G | A->G | - | - | - |
| comp12751_c0_seq1 | 33 | A | A->G | A->G | A->G | - | - | - |
| comp16006_c1_seq1 | 30 | A | A->G | A->G | A->G | - | - | - |
| comp28762_c0_seq1 | 326 | A | A->G | A->G | A->G | - | - | - |
| comp17423_c1_seq1 | 33 | A | A->G | A->G | A->G | - | - | - |
| comp36842_c0_seq3 | 2121 | A | A->G | A->G | A->G | - | - | - |
| comp33021_c0_seq2 | 493 | A | A->G | A->G | A->G | - | - | - |
| comp33021_c0_seq2 | 504 | A | A->G | A->G | A->G | - | - | - |
| comp958_c0_seq1 | 37 | A | A->G | A->G | A->G | - | - | - |
| comp958_c0_seq1 | 49 | A | A->G | A->G | A->G | - | - | - |
| comp958_c0_seq1 | 39 | A | A->G | A->G | A->G | - | - | - |
| comp958_c0_seq1 | 27 | A | A->G | A->G | A->G | - | - | - |
| comp3920_c0_seq1 | 180 | A | A->G | A->G | A->G | - | - | - |
| comp2888_c0_seq1 | 64 | A | A->G | A->G | A->G | - | - | - |
| comp16254_c0_seq1 | 276 | A | A->G | A->G | A->G | - | - | - |
| comp33021_c0_seq2 | 472 | A | A->G | A->G | A->G | - | - | - |
| comp36842_c0_seq3 | 1260 | A | A->G | A->G | A->G | - | - | - |
| comp25704_c1_seq1 | 715 | A | A->G | A->G | A->G | - | - | - |
| comp31808_c1_seq1 | 157 | A | A->G | A->G | A->G | - | - | - |
| comp14119_c0_seq1 | 369 | A | A->G | A->G | A->G | - | - | - |
| comp16632_c0_seq1 | 405 | A | A->G | A->G | A->G | - | - | - |
| comp12230_c0_seq1 | 370 | A | A->G | A->G | A->G | - | - | - |
| comp100119_c0_seq1 | 16 | A | A->G | A->G | A->G | - | - | - |
| comp36982_c0_seq7 | 233 | A | A->G | A->G | A->G | - | - | - |
| comp36982_c0_seq7 | 241 | A | A->G | A->G | A->G | - | - | - |
| comp36982_c0_seq7 | 247 | A | A->G | A->G | A->G | - | - | - |
| comp1099562_c0_seq1 | 229 | A | A->G | A->G | A->G | - | - | - |
| comp1099562_c0_seq1 | 250 | A | A->G | A->G | A->G | - | - | - |
| comp36934_c0_seq3 | 1807 | A | A->G | A->G | A->G | - | - | - |
| comp12194_c0_seq1 | 174 | A | A->G | A->G | A->G | - | - | - |
| comp10099_c0_seq1 | 244 | A | A->G | A->G | A->G | - | - | - |
| comp37004_c0_seq2 | 607 | A | A->G | A->G | A->G | - | - | - |
| comp10099_c0_seq1 | 249 | A | A->G | A->G | A->G | - | - | - |
| comp12230_c0_seq1 | 321 | A | A->G | A->G | A->G | - | - | - |
| comp35250_c0_seq3 | 1366 | A | A->G | A->G | A->G | - | - | - |
| comp26494_c0_seq2 | 623 | A | A->G | A->G | A->G | - | - | - |
| comp35250_c0_seq3 | 1356 | A | A->G | A->G | A->G | - | - | - |
| comp10099_c0_seq1 | 272 | A | A->G | A->G | A->G | - | - | - |
| comp1376072_c0_seq1 | 236 | A | A->G | A->G | A->G | - | - | - |
| comp14089_c0_seq1 | 260 | A | A->G | A->G | A->G | - | - | - |
| comp12230_c0_seq1 | 408 | A | A->G | A->G | A->G | - | - | - |
| comp36828_c0_seq2 | 3852 | A | A->G | A->G | A->G | - | - | - |
| comp36934_c0_seq3 | 315 | A | A->G | A->G | A->G | - | - | - |
| comp10099_c0_seq1 | 277 | A | A->G | A->G | A->G | - | - | - |
| comp1376072_c0_seq1 | 242 | A | A->G | A->G | A->G | - | - | - |
| comp10099_c0_seq1 | 293 | A | A->G | A->G | A->G | - | - | - |
| comp34885_c0_seq4 | 1677 | A | A->G | A->G | A->G | - | - | - |
| comp35726_c0_seq1 | 1017 | A | A->G | A->G | A->G | - | - | - |
| comp10099_c0_seq1 | 296 | A | A->G | A->G | A->G | - | - | - |
| comp30024_c0_seq1 | 9 | A | A->G | A->G | A->G | - | - | - |
| comp14946_c1_seq1 | 43 | A | A->G | A->G | A->G | - | - | - |
| comp15561_c0_seq1 | 320 | A | A->G | A->G | A->G | - | - | - |
| comp36828_c0_seq2 | 2111 | A | A->G | A->G | A->G | - | - | - |
| comp10099_c0_seq1 | 299 | A | A->G | A->G | A->G | - | - | - |
| comp10099_c0_seq1 | 314 | A | A->G | A->G | A->G | - | - | - |
| comp1033618_c0_seq1 | 111 | A | A->G | A->G | A->G | - | - | - |
| comp36329_c0_seq3 | 236 | A | A->G | A->G | A->G | - | - | - |
| comp12230_c0_seq1 | 341 | A | A->G | A->G | A->G | - | - | - |
| comp36982_c0_seq25 | 171 | A | A->G | A->G | A->G | - | - | - |
| comp36732_c1_seq8 | 132 | A | A->G | A->G | A->G | - | - | - |
| comp28762_c0_seq1 | 208 | A | A->G | A->G | A->G | - | - | - |
| comp36700_c0_seq1 | 1054 | A | A->G | A->G | A->G | - | - | - |
| comp36700_c0_seq12 | 1086 | A | A->G | A->G | A->G | - | - | - |
| comp36700_c0_seq16 | 2738 | A | A->G | A->G | A->G | - | - | - |
| comp36700_c0_seq2 | 1054 | A | A->G | A->G | A->G | - | - | - |
| comp36700_c0_seq24 | 1041 | A | A->G | A->G | A->G | - | - | - |
| comp36700_c0_seq3 | 1041 | A | A->G | A->G | A->G | - | - | - |
| comp36700_c0_seq6 | 2738 | A | A->G | A->G | A->G | - | - | - |
| comp36700_c0_seq9 | 1086 | A | A->G | A->G | A->G | - | - | - |
| comp33018_c0_seq1 | 1 | A | A->G | A->G | A->G | - | - | - |
| comp36842_c0_seq3 | 1676 | A | A->G | A->G | A->G | - | - | - |
| comp24027_c0_seq1 | 132 | A | A->G | A->G | A->G | - | - | - |
| comp36732_c1_seq8 | 148 | A | A->G | A->G | A->G | - | - | - |
| comp36732_c1_seq8 | 98 | A | A->G | A->G | A->G | - | - | - |
| comp36732_c1_seq8 | 106 | A | A->G | A->G | A->G | - | - | - |
| comp36329_c0_seq3 | 225 | A | A->G | A->G | A->G | - | - | - |
| comp36828_c0_seq2 | 2894 | A | A->G | A->G | A->G | - | - | - |
| comp36828_c0_seq2 | 2902 | A | A->G | A->G | A->G | - | - | - |
| comp17412_c0_seq1 | 406 | A | A->G | A->G | A->G | - | - | - |
| comp36732_c1_seq8 | 112 | A | A->G | A->G | A->G | - | - | - |
| comp10821_c0_seq1 | 25 | A | A->G | A->G | A->G | - | - | - |
| comp14946_c1_seq1 | 44 | A | A->G | A->G | A->G | - | - | - |
| comp19872_c0_seq1 | 86 | A | A->G | A->G | A->G | - | - | - |
| comp36732_c1_seq8 | 40 | A | A->G | A->G | A->G | - | - | - |
| comp36732_c1_seq8 | 119 | A | A->G | A->G | A->G | - | - | - |
| comp36732_c1_seq8 | 137 | A | A->G | A->G | A->G | - | - | - |
| comp12751_c0_seq1 | 29 | A | A->G | A->G | A->G | - | - | - |
| comp16461_c0_seq1 | 60 | A | A->G | A->G | A->G | - | - | - |
| comp15280_c0_seq1 | 314 | A | A->C | A->C | A->C | - | - | - |
| comp11833_c0_seq1 | 16 | A | A->C | A->C | A->C | - | - | - |
| comp14842_c0_seq1 | 147 | A | A->C | A->C | A->C | - | - | - |
| comp17570_c0_seq1 | 277 | A | A->C | A->C | A->C | - | - | - |
| comp1033_c0_seq1 | 1228 | A | A->C | A->C | A->C | - | - | - |
| comp1338_c0_seq1 | 100 | A | A->C | A->C | A->C | - | - | - |
| comp24881_c0_seq1 | 139 | A | A->C | A->C | A->C | - | - | - |
| comp11833_c0_seq1 | 9 | A | A->C | A->C | A->C | - | - | - |
| comp11699_c0_seq1 | 936 | A | A->C | A->C | A->C | - | - | - |
| comp20299_c0_seq1 | 118 | A | A->C | A->C | A->C | - | - | - |
| comp17536_c0_seq1 | 141 | A | A->C | A->C | A->C | - | - | - |
| comp10933_c0_seq1 | 157 | A | A->C | A->C | A->C | - | - | - |
| comp857739_c0_seq1 | 112 | A | A->C | A->C | A->C | - | - | - |
| comp15020_c0_seq1 | 217 | A | A->C | A->C | A->C | - | - | - |
| comp16016_c0_seq1 | 213 | A | A->C | A->C | A->C | - | - | - |
| comp32754_c0_seq1 | 81 | A | A->C | A->C | A->C | - | - | - |
| comp30501_c0_seq1 | 86 | A | A->C | A->C | A->C | - | - | - |
| comp12332_c0_seq1 | 256 | A | A->C | A->C | A->C | - | - | - |
| comp11866_c0_seq1 | 655 | A | A->C | A->C | A->C | - | - | - |
| comp11699_c0_seq1 | 1102 | A | A->C | A->C | A->C | - | - | - |
| comp35802_c0_seq3 | 274 | A | A->C | A->C | A->C | - | - | - |
| comp16918_c0_seq1 | 163 | A | A->C | A->C | A->C | - | - | - |
| comp16126_c0_seq1 | 82 | A | A->C | A->C | A->C | - | - | - |
| comp34606_c1_seq4 | 25 | A | A->C | A->C | A->C | - | - | - |
| comp36905_c0_seq2 | 333 | A | A->C | A->C | A->C | - | - | - |
| comp1416779_c0_seq1 | 178 | A | A->C | A->C | A->C | - | - | - |
| comp16804_c1_seq1 | 473 | A | A->C | A->C | A->C | - | - | - |
| comp2627_c0_seq1 | 105 | A | A->C | A->C | A->C | - | - | - |
| comp30993_c0_seq1 | 366 | A | A->C | A->C | A->C | - | - | - |
| comp21199_c0_seq1 | 215 | A | A->C | A->C | A->C | - | - | - |
| comp22810_c0_seq1 | 545 | A | A->C | A->C | A->C | - | - | - |
| comp30501_c0_seq1 | 4339 | A | A->C | A->C | A->C | - | - | - |
| comp162_c0_seq1 | 212 | A | A->C | A->C | A->C | - | - | - |
| comp2932_c0_seq1 | 700 | A | A->C | A->C | A->C | - | - | - |
| comp11699_c0_seq1 | 324 | A | A->C | A->C | A->C | - | - | - |
| comp10933_c0_seq1 | 65 | A | A->C | A->C | A->C | - | - | - |
| comp32377_c0_seq1 | 124 | A | A->C | A->C | A->C | - | - | - |
| comp26220_c0_seq1 | 2569 | A | A->C | A->C | A->C | - | - | - |
| comp10400_c0_seq1 | 504 | A | A->C | A->C | A->C | - | - | - |
| comp26814_c0_seq2 | 17 | A | A->C | A->C | A->C | - | - | - |
| comp13288_c0_seq1 | 1716 | A | A->C | A->C | A->C | - | - | - |
| comp17570_c0_seq1 | 151 | A | A->C | A->C | A->C | - | - | - |
| comp11699_c0_seq1 | 1025 | A | A->C | A->C | A->C | - | - | - |
| comp11699_c0_seq1 | 934 | A | A->C | A->C | A->C | - | - | - |
| comp16006_c1_seq1 | 123 | A | A->C | A->C | A->C | - | - | - |
| comp11699_c0_seq1 | 1065 | A | A->C | A->C | A->C | - | - | - |
| comp11833_c0_seq1 | 150 | A | A->C | A->C | A->C | - | - | - |
| comp11699_c0_seq1 | 915 | A | A->C | A->C | A->C | - | - | - |
| comp16006_c1_seq1 | 75 | A | A->C | A->C | A->C | - | - | - |
| comp10933_c0_seq1 | 130 | A | A->C | A->C | A->C | - | - | - |
| comp24869_c0_seq1 | 127 | A | A->C | A->C | A->C | - | - | - |
| comp958_c0_seq1 | 25 | A | A->C | A->C | A->C | - | - | - |
| comp36746_c0_seq5 | 92 | A | A->C | A->C | A->C | - | - | - |
| comp1099562_c0_seq1 | 169 | A | A->C | A->C | A->C | - | - | - |
| comp1239504_c0_seq1 | 67 | A | A->C | A->C | A->C | - | - | - |
| comp2987_c0_seq1 | 36 | A | A->C | A->C | A->C | - | - | - |
| comp25395_c1_seq1 | 626 | A | A->C | A->C | A->C | - | - | - |
| comp25395_c1_seq1 | 607 | A | A->C | A->C | A->C | - | - | - |
| comp18176_c0_seq1 | 354 | A | A->C | A->C | A->C | - | - | - |
| comp1052985_c0_seq1 | 36 | A | A->C | A->C | A->C | - | - | - |
| comp13466_c0_seq1 | 1790 | A | A->C | A->C | A->C | - | - | - |
| comp1052985_c0_seq1 | 27 | A | A->C | A->C | A->C | - | - | - |
| comp10099_c0_seq1 | 289 | A | A->C | A->C | A->C | - | - | - |
| comp14977_c0_seq1 | 16 | A | A->C | A->C | A->C | - | - | - |
| comp22014_c1_seq1 | 129 | A | A->C | A->C | A->C | - | - | - |
| comp36982_c0_seq7 | 280 | A | A->C | A->C | A->C | - | - | - |
| comp1236573_c0_seq1 | 355 | A | A->C | A->C | A->C | - | - | - |
| comp17423_c1_seq1 | 37 | A | A->C | A->C | A->C | - | - | - |
| comp36828_c0_seq2 | 3051 | A | A->C | A->C | A->C | - | - | - |
| comp36982_c0_seq25 | 106 | A | A->C | A->C | A->C | - | - | - |
| comp36732_c1_seq8 | 99 | A | A->C | A->C | A->C | - | - | - |
| comp36732_c1_seq8 | 110 | A | A->C | A->C | A->C | - | - | - |
| comp37004_c0_seq2 | 1016 | A | A->C | A->C | A->C | - | - | - |
| comp16632_c0_seq1 | 412 | A | A->C | A->C | A->C | - | - | - |
| comp17423_c1_seq1 | 34 | A | A->C | A->C | A->C | - | - | - |
| comp36732_c1_seq8 | 56 | A | A->C | A->C | A->C | - | - | - |
| comp36779_c0_seq4 | 675 | C | C->T | C->T | C->T | - | - | - |
| comp36017_c0_seq1 | 2943 | C | C->T | C->T | C->T | - | - | - |
| comp33630_c1_seq1 | 2066 | C | C->T | C->T | C->T | - | - | - |
| comp15302_c0_seq1 | 241 | C | C->T | C->T | C->T | - | - | - |
| comp37004_c0_seq2 | 1015 | C | C->T | C->T | C->T | - | - | - |
| comp12194_c0_seq1 | 119 | C | C->T | C->T | C->T | - | - | - |
| comp13328_c2_seq1 | 1466 | C | C->T | C->T | C->T | - | - | - |
| comp34606_c1_seq4 | 23 | C | C->T | C->T | C->T | - | - | - |
| comp20274_c0_seq1 | 1218 | C | C->T | C->T | C->T | - | - | - |
| comp12194_c0_seq1 | 77 | C | C->T | C->T | C->T | - | - | - |
| comp21759_c0_seq1 | 24 | C | C->T | C->T | C->T | - | - | - |
| comp17144_c1_seq1 | 257 | C | C->T | C->T | C->T | - | - | - |
| comp36828_c0_seq2 | 2135 | C | C->T | C->T | C->T | - | - | - |
| comp831084_c0_seq1 | 78 | C | C->T | C->T | C->T | - | - | - |
| comp33264_c0_seq24 | 1522 | C | C->T | C->T | C->T | - | - | - |
| comp13897_c0_seq1 | 301 | C | C->T | C->T | C->T | - | - | - |
| comp1558_c0_seq1 | 15 | C | C->T | C->T | C->T | - | - | - |
| comp17779_c0_seq1 | 441 | C | C->T | C->T | C->T | - | - | - |
| comp35726_c0_seq1 | 1004 | C | C->T | C->T | C->T | - | - | - |
| comp35054_c0_seq9 | 10 | C | C->T | C->T | C->T | - | - | - |
| comp12194_c0_seq1 | 182 | C | C->T | C->T | C->T | - | - | - |
| comp10117_c0_seq1 | 42 | C | C->T | C->T | C->T | - | - | - |
| comp35025_c2_seq1 | 326 | C | C->T | C->T | C->T | - | - | - |
| comp1376072_c0_seq1 | 235 | C | C->T | C->T | C->T | - | - | - |
| comp12230_c0_seq1 | 368 | C | C->T | C->T | C->T | - | - | - |
| comp2987_c0_seq1 | 32 | C | C->T | C->T | C->T | - | - | - |
| comp25395_c1_seq1 | 606 | C | C->T | C->T | C->T | - | - | - |
| comp16632_c0_seq1 | 429 | C | C->T | C->T | C->T | - | - | - |
| comp36448_c0_seq5 | 825 | C | C->T | C->T | C->T | - | - | - |
| comp10099_c0_seq1 | 229 | C | C->T | C->T | C->T | - | - | - |
| comp33113_c0_seq4 | 2198 | C | C->T | C->T | C->T | - | - | - |
| comp1099562_c0_seq1 | 199 | C | C->T | C->T | C->T | - | - | - |
| comp36828_c0_seq2 | 4064 | C | C->T | C->T | C->T | - | - | - |
| comp36934_c0_seq3 | 1770 | C | C->T | C->T | C->T | - | - | - |
| comp958_c0_seq1 | 48 | C | C->T | C->T | C->T | - | - | - |
| comp27107_c0_seq1 | 492 | C | C->T | C->T | C->T | - | - | - |
| comp27720_c0_seq1 | 492 | C | C->T | C->T | C->T | - | - | - |
| comp27720_c0_seq1 | 505 | C | C->T | C->T | C->T | - | - | - |
| comp25459_c0_seq1 | 9 | C | C->T | C->T | C->T | - | - | - |
| comp2008_c0_seq1 | 266 | C | C->T | C->T | C->T | - | - | - |
| comp14211_c0_seq1 | 131 | C | C->T | C->T | C->T | - | - | - |
| comp12194_c0_seq1 | 161 | C | C->T | C->T | C->T | - | - | - |
| comp16918_c0_seq1 | 101 | C | C->T | C->T | C->T | - | - | - |
| comp16918_c0_seq1 | 117 | C | C->T | C->T | C->T | - | - | - |
| comp16918_c0_seq1 | 110 | C | C->T | C->T | C->T | - | - | - |
| comp26158_c1_seq1 | 85 | C | C->T | C->T | C->T | - | - | - |
| comp16048_c0_seq1 | 631 | C | C->T | C->T | C->T | - | - | - |
| comp16006_c1_seq1 | 63 | C | C->T | C->T | C->T | - | - | - |
| comp16006_c1_seq1 | 117 | C | C->T | C->T | C->T | - | - | - |
| comp16006_c1_seq1 | 93 | C | C->T | C->T | C->T | - | - | - |
| comp26232_c0_seq1 | 32 | C | C->T | C->T | C->T | - | - | - |
| comp16006_c1_seq1 | 90 | C | C->T | C->T | C->T | - | - | - |
| comp16006_c1_seq1 | 133 | C | C->T | C->T | C->T | - | - | - |
| comp16918_c0_seq1 | 119 | C | C->T | C->T | C->T | - | - | - |
| comp16006_c1_seq1 | 138 | C | C->T | C->T | C->T | - | - | - |
| comp17570_c0_seq1 | 148 | C | C->T | C->T | C->T | - | - | - |
| comp35445_c0_seq1 | 1406 | C | C->T | C->T | C->T | - | - | - |
| comp29032_c0_seq1 | 1140 | C | C->T | C->T | C->T | - | - | - |
| comp4616_c0_seq1 | 103 | C | C->T | C->T | C->T | - | - | - |
| comp17274_c0_seq1 | 109 | C | C->T | C->T | C->T | - | - | - |
| comp36828_c0_seq2 | 2285 | C | C->T | C->T | C->T | - | - | - |
| comp11699_c0_seq1 | 861 | C | C->T | C->T | C->T | - | - | - |
| comp1183_c0_seq1 | 114 | C | C->T | C->T | C->T | - | - | - |
| comp33634_c0_seq5 | 174 | C | C->T | C->T | C->T | - | - | - |
| comp30447_c0_seq1 | 216 | C | C->T | C->T | C->T | - | - | - |
| comp15937_c0_seq1 | 381 | C | C->T | C->T | C->T | - | - | - |
| comp8288_c0_seq1 | 522 | C | C->T | C->T | C->T | - | - | - |
| comp11699_c0_seq1 | 1152 | C | C->T | C->T | C->T | - | - | - |
| comp8288_c0_seq1 | 498 | C | C->T | C->T | C->T | - | - | - |
| comp33478_c0_seq1 | 698 | C | C->T | C->T | C->T | - | - | - |
| comp30447_c0_seq1 | 55 | C | C->T | C->T | C->T | - | - | - |
| comp31109_c0_seq1 | 1576 | C | C->T | C->T | C->T | - | - | - |
| comp996794_c0_seq1 | 159 | C | C->T | C->T | C->T | - | - | - |
| comp33212_c0_seq4 | 4227 | C | C->T | C->T | C->T | - | - | - |
| comp1081242_c0_seq1 | 139 | C | C->T | C->T | C->T | - | - | - |
| comp10805_c0_seq1 | 145 | C | C->T | C->T | C->T | - | - | - |
| comp18565_c0_seq1 | 336 | C | C->T | C->T | C->T | - | - | - |
| comp28710_c0_seq1 | 24 | C | C->T | C->T | C->T | - | - | - |
| comp36842_c0_seq3 | 1290 | C | C->T | C->T | C->T | - | - | - |
| comp20740_c0_seq1 | 156 | C | C->T | C->T | C->T | - | - | - |
| comp1132626_c0_seq1 | 98 | C | C->T | C->T | C->T | - | - | - |
| comp27258_c0_seq1 | 1659 | C | C->T | C->T | C->T | - | - | - |
| comp35641_c1_seq4 | 272 | C | C->T | C->T | C->T | - | - | - |
| comp743428_c0_seq1 | 313 | C | C->T | C->T | C->T | - | - | - |
| comp27805_c1_seq2 | 694 | C | C->T | C->T | C->T | - | - | - |
| comp20303_c1_seq1 | 206 | C | C->T | C->T | C->T | - | - | - |
| comp14946_c1_seq1 | 55 | C | C->T | C->T | C->T | - | - | - |
| comp30843_c0_seq2 | 318 | C | C->T | C->T | C->T | - | - | - |
| comp1489_c0_seq1 | 45 | C | C->T | C->T | C->T | - | - | - |
| comp29853_c0_seq2 | 501 | C | C->T | C->T | C->T | - | - | - |
| comp13473_c0_seq1 | 203 | C | C->T | C->T | C->T | - | - | - |
| comp708392_c0_seq1 | 243 | C | C->T | C->T | C->T | - | - | - |
| comp4400_c0_seq1 | 475 | C | C->T | C->T | C->T | - | - | - |
| comp896261_c0_seq1 | 732 | C | C->T | C->T | C->T | - | - | - |
| comp840742_c0_seq1 | 399 | C | C->T | C->T | C->T | - | - | - |
| comp11699_c0_seq1 | 1128 | C | C->T | C->T | C->T | - | - | - |
| comp17422_c0_seq1 | 330 | C | C->T | C->T | C->T | - | - | - |
| comp23384_c0_seq1 | 79 | C | C->T | C->T | C->T | - | - | - |
| comp1127129_c0_seq1 | 245 | C | C->T | C->T | C->T | - | - | - |
| comp25704_c1_seq1 | 703 | C | C->T | C->T | C->T | - | - | - |
| comp14274_c0_seq1 | 36 | C | C->T | C->T | C->T | - | - | - |
| comp22827_c0_seq1 | 2683 | C | C->T | C->T | C->T | - | - | - |
| comp22827_c0_seq1 | 2678 | C | C->T | C->T | C->T | - | - | - |
| comp16611_c0_seq1 | 158 | C | C->T | C->T | C->T | - | - | - |
| comp21199_c0_seq1 | 221 | C | C->T | C->T | C->T | - | - | - |
| comp31001_c0_seq1 | 15 | C | C->T | C->T | C->T | - | - | - |
| comp22382_c0_seq1 | 1008 | C | C->T | C->T | C->T | - | - | - |
| comp36017_c0_seq1 | 3270 | C | C->T | C->T | C->T | - | - | - |
| comp29694_c0_seq1 | 1128 | C | C->T | C->T | C->T | - | - | - |
| comp33130_c0_seq1 | 896 | C | C->T | C->T | C->T | - | - | - |
| comp26825_c0_seq1 | 163 | C | C->T | C->T | C->T | - | - | - |
| comp22226_c1_seq1 | 393 | C | C->T | C->T | C->T | - | - | - |
| comp17165_c0_seq1 | 877 | C | C->T | C->T | C->T | - | - | - |
| comp22810_c0_seq1 | 334 | C | C->T | C->T | C->T | - | - | - |
| comp12524_c0_seq1 | 20 | C | C->T | C->T | C->T | - | - | - |
| comp11699_c0_seq1 | 1005 | C | C->T | C->T | C->T | - | - | - |
| comp36974_c0_seq5 | 723 | C | C->T | C->T | C->T | - | - | - |
| comp14030_c0_seq2 | 596 | C | C->T | C->T | C->T | - | - | - |
| comp1308_c0_seq1 | 74 | C | C->T | C->T | C->T | - | - | - |
| comp13278_c1_seq1 | 196 | C | C->T | C->T | C->T | - | - | - |
| comp1053913_c0_seq1 | 208 | C | C->T | C->T | C->T | - | - | - |
| comp14595_c0_seq1 | 117 | C | C->T | C->T | C->T | - | - | - |
| comp34265_c0_seq1 | 794 | C | C->T | C->T | C->T | - | - | - |
| comp36017_c0_seq1 | 3972 | C | C->T | C->T | C->T | - | - | - |
| comp17536_c0_seq1 | 137 | C | C->T | C->T | C->T | - | - | - |
| comp830210_c0_seq1 | 81 | C | C->T | C->T | C->T | - | - | - |
| comp36691_c0_seq7 | 1109 | C | C->T | C->T | C->T | - | - | - |
| comp11833_c0_seq1 | 1 | C | C->T | C->T | C->T | - | - | - |
| comp29781_c1_seq1 | 418 | C | C->T | C->T | C->T | - | - | - |
| comp35641_c1_seq4 | 284 | C | C->T | C->T | C->T | - | - | - |
| comp17009_c1_seq1 | 643 | C | C->T | C->T | C->T | - | - | - |
| comp32437_c2_seq1 | 1288 | C | C->T | C->T | C->T | - | - | - |
| comp32437_c2_seq1 | 544 | C | C->T | C->T | C->T | - | - | - |
| comp31181_c0_seq1 | 774 | C | C->T | C->T | C->T | - | - | - |
| comp12153_c0_seq1 | 118 | C | C->T | C->T | C->T | - | - | - |
| comp12553_c0_seq1 | 75 | C | C->T | C->T | C->T | - | - | - |
| comp17777_c0_seq1 | 681 | C | C->T | C->T | C->T | - | - | - |
| comp33095_c0_seq1 | 654 | C | C->T | C->T | C->T | - | - | - |
| comp25664_c0_seq1 | 3026 | C | C->T | C->T | C->T | - | - | - |
| comp11899_c0_seq1 | 91 | C | C->T | C->T | C->T | - | - | - |
| comp12391_c0_seq1 | 75 | C | C->T | C->T | C->T | - | - | - |
| comp12962_c0_seq1 | 131 | C | C->T | C->T | C->T | - | - | - |
| comp35397_c0_seq1 | 426 | C | C->T | C->T | C->T | - | - | - |
| comp14508_c0_seq1 | 1268 | C | C->T | C->T | C->T | - | - | - |
| comp14706_c0_seq1 | 205 | C | C->T | C->T | C->T | - | - | - |
| comp28320_c0_seq1 | 157 | C | C->T | C->T | C->T | - | - | - |
| comp16029_c0_seq1 | 593 | C | C->T | C->T | C->T | - | - | - |
| comp29163_c0_seq1 | 1729 | C | C->T | C->T | C->T | - | - | - |
| comp34672_c0_seq1 | 1766 | C | C->T | C->T | C->T | - | - | - |
| comp33144_c0_seq1 | 2242 | C | C->T | C->T | C->T | - | - | - |
| comp12159_c0_seq1 | 127 | C | C->T | C->T | C->T | - | - | - |
| comp30864_c0_seq2 | 72 | C | C->T | C->T | C->T | - | - | - |
| comp15756_c0_seq1 | 224 | C | C->T | C->T | C->T | - | - | - |
| comp36385_c0_seq2 | 453 | C | C->T | C->T | C->T | - | - | - |
| comp33055_c0_seq1 | 576 | C | C->T | C->T | C->T | - | - | - |
| comp36208_c0_seq1 | 4910 | C | C->T | C->T | C->T | - | - | - |
| comp24276_c0_seq2 | 142 | C | C->T | C->T | C->T | - | - | - |
| comp29635_c1_seq1 | 997 | C | C->T | C->T | C->T | - | - | - |
| comp34587_c1_seq5 | 466 | C | C->T | C->T | C->T | - | - | - |
| comp15565_c0_seq1 | 200 | C | C->T | C->T | C->T | - | - | - |
| comp12418_c0_seq1 | 766 | C | C->T | C->T | C->T | - | - | - |
| comp34107_c1_seq6 | 3822 | C | C->T | C->T | C->T | - | - | - |
| comp11827_c0_seq1 | 24 | C | C->T | C->T | C->T | - | - | - |
| comp16126_c0_seq1 | 52 | C | C->T | C->T | C->T | - | - | - |
| comp31255_c0_seq1 | 263 | C | C->T | C->T | C->T | - | - | - |
| comp2900_c0_seq1 | 172 | C | C->T | C->T | C->T | - | - | - |
| comp36556_c0_seq1 | 712 | C | C->T | C->T | C->T | - | - | - |
| comp29181_c0_seq1 | 4254 | C | C->T | C->T | C->T | - | - | - |
| comp36415_c0_seq5 | 1930 | C | C->T | C->T | C->T | - | - | - |
| comp29654_c0_seq1 | 1049 | C | C->T | C->T | C->T | - | - | - |
| comp13801_c0_seq1 | 590 | C | C->T | C->T | C->T | - | - | - |
| comp13225_c0_seq1 | 393 | C | C->T | C->T | C->T | - | - | - |
| comp34454_c0_seq3 | 918 | C | C->T | C->T | C->T | - | - | - |
| comp35137_c0_seq1 | 605 | C | C->T | C->T | C->T | - | - | - |
| comp21774_c0_seq1 | 289 | C | C->T | C->T | C->T | - | - | - |
| comp35525_c0_seq1 | 91 | C | C->T | C->T | C->T | - | - | - |
| comp25376_c0_seq1 | 322 | C | C->T | C->T | C->T | - | - | - |
| comp23013_c0_seq1 | 68 | C | C->T | C->T | C->T | - | - | - |
| comp35888_c3_seq9 | 261 | C | C->T | C->T | C->T | - | - | - |
| comp15280_c0_seq1 | 480 | C | C->T | C->T | C->T | - | - | - |
| comp21701_c0_seq1 | 809 | C | C->T | C->T | C->T | - | - | - |
| comp9865_c0_seq1 | 166 | C | C->T | C->T | C->T | - | - | - |
| comp1051241_c0_seq1 | 570 | C | C->T | C->T | C->T | - | - | - |
| comp27968_c0_seq1 | 18 | C | C->T | C->T | C->T | - | - | - |
| comp16348_c0_seq1 | 98 | C | C->T | C->T | C->T | - | - | - |
| comp11699_c0_seq1 | 1171 | C | C->T | C->T | C->T | - | - | - |
| comp16497_c0_seq1 | 990 | C | C->T | C->T | C->T | - | - | - |
| comp36836_c0_seq1 | 1127 | C | C->T | C->T | C->T | - | - | - |
| comp15200_c0_seq1 | 359 | C | C->T | C->T | C->T | - | - | - |
| comp14730_c0_seq1 | 1724 | C | C->T | C->T | C->T | - | - | - |
| comp16005_c0_seq1 | 227 | C | C->T | C->T | C->T | - | - | - |
| comp16789_c0_seq1 | 221 | C | C->T | C->T | C->T | - | - | - |
| comp10933_c0_seq1 | 139 | C | C->T | C->T | C->T | - | - | - |
| comp10933_c0_seq1 | 163 | C | C->T | C->T | C->T | - | - | - |
| comp708392_c0_seq1 | 512 | C | C->T | C->T | C->T | - | - | - |
| comp17274_c0_seq1 | 112 | C | C->T | C->T | C->T | - | - | - |
| comp10933_c0_seq1 | 136 | C | C->T | C->T | C->T | - | - | - |
| comp560665_c0_seq1 | 304 | C | C->T | C->T | C->T | - | - | - |
| comp36732_c1_seq8 | 139 | C | C->G | C->G | C->G | - | - | - |
| comp36732_c1_seq8 | 55 | C | C->G | C->G | C->G | - | - | - |
| comp36732_c1_seq8 | 64 | C | C->G | C->G | C->G | - | - | - |
| comp36732_c1_seq8 | 76 | C | C->G | C->G | C->G | - | - | - |
| comp36732_c1_seq8 | 83 | C | C->G | C->G | C->G | - | - | - |
| comp33264_c0_seq24 | 1471 | C | C->G | C->G | C->G | - | - | - |
| comp36828_c0_seq2 | 3018 | C | C->G | C->G | C->G | - | - | - |
| comp36732_c1_seq8 | 97 | C | C->G | C->G | C->G | - | - | - |
| comp12230_c0_seq1 | 295 | C | C->G | C->G | C->G | - | - | - |
| comp13325_c0_seq1 | 499 | C | C->G | C->G | C->G | - | - | - |
| comp12709_c0_seq3 | 142 | C | C->G | C->G | C->G | - | - | - |
| comp14128_c0_seq1 | 31 | C | C->G | C->G | C->G | - | - | - |
| comp694013_c0_seq1 | 159 | C | C->G | C->G | C->G | - | - | - |
| comp35726_c0_seq1 | 1005 | C | C->G | C->G | C->G | - | - | - |
| comp17423_c1_seq1 | 44 | C | C->G | C->G | C->G | - | - | - |
| comp32483_c0_seq3 | 71 | C | C->G | C->G | C->G | - | - | - |
| comp17423_c1_seq1 | 49 | C | C->G | C->G | C->G | - | - | - |
| comp17423_c1_seq1 | 36 | C | C->G | C->G | C->G | - | - | - |
| comp1099562_c0_seq1 | 214 | C | C->G | C->G | C->G | - | - | - |
| comp30036_c1_seq2 | 869 | C | C->G | C->G | C->G | - | - | - |
| comp1052985_c0_seq1 | 28 | C | C->G | C->G | C->G | - | - | - |
| comp825887_c0_seq1 | 113 | C | C->G | C->G | C->G | - | - | - |
| comp1099562_c0_seq1 | 132 | C | C->G | C->G | C->G | - | - | - |
| comp12751_c0_seq1 | 39 | C | C->G | C->G | C->G | - | - | - |
| comp11699_c0_seq1 | 996 | C | C->G | C->G | C->G | - | - | - |
| comp16006_c1_seq1 | 120 | C | C->G | C->G | C->G | - | - | - |
| comp16006_c1_seq1 | 129 | C | C->G | C->G | C->G | - | - | - |
| comp16918_c0_seq1 | 94 | C | C->G | C->G | C->G | - | - | - |
| comp23692_c0_seq1 | 1203 | C | C->G | C->G | C->G | - | - | - |
| comp819551_c0_seq1 | 273 | C | C->G | C->G | C->G | - | - | - |
| comp35974_c0_seq1 | 418 | C | C->G | C->G | C->G | - | - | - |
| comp857739_c0_seq1 | 48 | C | C->G | C->G | C->G | - | - | - |
| comp22782_c0_seq1 | 169 | C | C->G | C->G | C->G | - | - | - |
| comp14308_c0_seq1 | 485 | C | C->G | C->G | C->G | - | - | - |
| comp30843_c0_seq2 | 294 | C | C->G | C->G | C->G | - | - | - |
| comp25482_c0_seq1 | 39 | C | C->G | C->G | C->G | - | - | - |
| comp16169_c0_seq1 | 114 | C | C->G | C->G | C->G | - | - | - |
| comp14595_c0_seq1 | 246 | C | C->G | C->G | C->G | - | - | - |
| comp402441_c0_seq1 | 962 | C | C->G | C->G | C->G | - | - | - |
| comp17536_c0_seq1 | 109 | C | C->G | C->G | C->G | - | - | - |
| comp28710_c0_seq1 | 42 | C | C->G | C->G | C->G | - | - | - |
| comp17477_c0_seq1 | 54 | C | C->G | C->G | C->G | - | - | - |
| comp1461_c0_seq1 | 254 | C | C->G | C->G | C->G | - | - | - |
| comp11699_c0_seq1 | 918 | C | C->G | C->G | C->G | - | - | - |
| comp4755_c0_seq1 | 158 | C | C->G | C->G | C->G | - | - | - |
| comp33686_c1_seq2 | 855 | C | C->G | C->G | C->G | - | - | - |
| comp10897_c0_seq1 | 188 | C | C->G | C->G | C->G | - | - | - |
| comp32534_c0_seq1 | 1274 | C | C->G | C->G | C->G | - | - | - |
| comp11833_c0_seq1 | 13 | C | C->A | C->A | C->A | - | - | - |
| comp33500_c0_seq1 | 1728 | C | C->A | C->A | C->A | - | - | - |
| comp35942_c1_seq3 | 229 | C | C->A | C->A | C->A | - | - | - |
| comp11833_c0_seq1 | 11 | C | C->A | C->A | C->A | - | - | - |
| comp11971_c0_seq1 | 50 | C | C->A | C->A | C->A | - | - | - |
| comp28260_c0_seq1 | 1490 | C | C->A | C->A | C->A | - | - | - |
| comp11699_c0_seq1 | 916 | C | C->A | C->A | C->A | - | - | - |
| comp16088_c0_seq1 | 71 | C | C->A | C->A | C->A | - | - | - |
| comp16077_c0_seq1 | 83 | C | C->A | C->A | C->A | - | - | - |
| comp15137_c0_seq1 | 369 | C | C->A | C->A | C->A | - | - | - |
| comp15137_c0_seq1 | 372 | C | C->A | C->A | C->A | - | - | - |
| comp12949_c0_seq1 | 90 | C | C->A | C->A | C->A | - | - | - |
| comp9335_c0_seq1 | 322 | C | C->A | C->A | C->A | - | - | - |
| comp12343_c0_seq1 | 1378 | C | C->A | C->A | C->A | - | - | - |
| comp36971_c0_seq1 | 301 | C | C->A | C->A | C->A | - | - | - |
| comp17536_c0_seq1 | 129 | C | C->A | C->A | C->A | - | - | - |
| comp16126_c0_seq1 | 66 | C | C->A | C->A | C->A | - | - | - |
| comp26534_c0_seq1 | 329 | C | C->A | C->A | C->A | - | - | - |
| comp33220_c0_seq1 | 728 | C | C->A | C->A | C->A | - | - | - |
| comp35627_c0_seq2 | 379 | C | C->A | C->A | C->A | - | - | - |
| comp27260_c1_seq1 | 494 | C | C->A | C->A | C->A | - | - | - |
| comp16525_c0_seq2 | 52 | C | C->A | C->A | C->A | - | - | - |
| comp14308_c0_seq1 | 482 | C | C->A | C->A | C->A | - | - | - |
| comp18703_c0_seq1 | 1999 | C | C->A | C->A | C->A | - | - | - |
| comp28709_c0_seq1 | 263 | C | C->A | C->A | C->A | - | - | - |
| comp10933_c0_seq1 | 187 | C | C->A | C->A | C->A | - | - | - |
| comp17279_c0_seq1 | 28 | C | C->A | C->A | C->A | - | - | - |
| comp21486_c0_seq1 | 348 | C | C->A | C->A | C->A | - | - | - |
| comp1132626_c0_seq1 | 89 | C | C->A | C->A | C->A | - | - | - |
| comp667491_c0_seq1 | 55 | C | C->A | C->A | C->A | - | - | - |
| comp14584_c1_seq1 | 444 | C | C->A | C->A | C->A | - | - | - |
| comp14308_c0_seq1 | 521 | C | C->A | C->A | C->A | - | - | - |
| comp34466_c0_seq2 | 42 | C | C->A | C->A | C->A | - | - | - |
| comp18582_c0_seq1 | 812 | C | C->A | C->A | C->A | - | - | - |
| comp16632_c0_seq1 | 406 | C | C->A | C->A | C->A | - | - | - |
| comp36329_c0_seq3 | 226 | C | C->A | C->A | C->A | - | - | - |
| comp36828_c0_seq2 | 2017 | C | C->A | C->A | C->A | - | - | - |
| comp958_c0_seq1 | 26 | C | C->A | C->A | C->A | - | - | - |
| comp958_c0_seq1 | 40 | C | C->A | C->A | C->A | - | - | - |
| comp36842_c0_seq3 | 1733 | C | C->A | C->A | C->A | - | - | - |
| comp25395_c1_seq1 | 643 | C | C->A | C->A | C->A | - | - | - |
| comp1052985_c0_seq1 | 37 | C | C->A | C->A | C->A | - | - | - |
| comp35904_c0_seq12 | 1224 | C | C->A | C->A | C->A | - | - | - |
| comp34930_c2_seq3 | 23 | C | C->A | C->A | C->A | - | - | - |
| comp18064_c0_seq1 | 40 | C | C->A | C->A | C->A | - | - | - |
| comp2631_c0_seq1 | 1090 | C | C->A | C->A | C->A | - | - | - |
| comp14128_c0_seq1 | 25 | C | C->A | C->A | C->A | - | - | - |
| comp36828_c0_seq2 | 1991 | C | C->A | C->A | C->A | - | - | - |
| comp36828_c0_seq2 | 1961 | G | G->T | G->T | G->T | - | - | - |
| comp36732_c1_seq8 | 63 | G | G->T | G->T | G->T | - | - | - |
| comp34141_c0_seq1 | 79 | G | G->T | G->T | G->T | - | - | - |
| comp25704_c1_seq1 | 690 | G | G->T | G->T | G->T | - | - | - |
| comp958_c0_seq1 | 28 | G | G->T | G->T | G->T | - | - | - |
| comp19014_c0_seq1 | 431 | G | G->T | G->T | G->T | - | - | - |
| comp34008_c2_seq6 | 36 | G | G->T | G->T | G->T | - | - | - |
| comp1052985_c0_seq1 | 16 | G | G->T | G->T | G->T | - | - | - |
| comp17423_c1_seq1 | 51 | G | G->T | G->T | G->T | - | - | - |
| comp12230_c0_seq1 | 380 | G | G->T | G->T | G->T | - | - | - |
| comp933796_c0_seq1 | 148 | G | G->T | G->T | G->T | - | - | - |
| comp1099562_c0_seq1 | 156 | G | G->T | G->T | G->T | - | - | - |
| comp1099562_c0_seq1 | 183 | G | G->T | G->T | G->T | - | - | - |
| comp10117_c0_seq1 | 48 | G | G->T | G->T | G->T | - | - | - |
| comp17423_c1_seq1 | 35 | G | G->T | G->T | G->T | - | - | - |
| comp21759_c0_seq1 | 28 | G | G->T | G->T | G->T | - | - | - |
| comp36923_c0_seq40 | 1435 | G | G->T | G->T | G->T | - | - | - |
| comp15849_c0_seq1 | 335 | G | G->T | G->T | G->T | - | - | - |
| comp16918_c0_seq1 | 104 | G | G->T | G->T | G->T | - | - | - |
| comp13408_c0_seq2 | 255 | G | G->T | G->T | G->T | - | - | - |
| comp16636_c0_seq1 | 355 | G | G->T | G->T | G->T | - | - | - |
| comp18506_c0_seq1 | 66 | G | G->T | G->T | G->T | - | - | - |
| comp35236_c1_seq1 | 3065 | G | G->T | G->T | G->T | - | - | - |
| comp35445_c0_seq1 | 1405 | G | G->T | G->T | G->T | - | - | - |
| comp16804_c1_seq1 | 457 | G | G->T | G->T | G->T | - | - | - |
| comp28710_c0_seq1 | 37 | G | G->T | G->T | G->T | - | - | - |
| comp20601_c0_seq1 | 412 | G | G->T | G->T | G->T | - | - | - |
| comp1130571_c0_seq1 | 237 | G | G->T | G->T | G->T | - | - | - |
| comp1558_c0_seq1 | 23 | G | G->T | G->T | G->T | - | - | - |
| comp30024_c0_seq1 | 10 | G | G->T | G->T | G->T | - | - | - |
| comp29724_c0_seq1 | 131 | G | G->T | G->T | G->T | - | - | - |
| comp15644_c0_seq1 | 238 | G | G->T | G->T | G->T | - | - | - |
| comp11699_c0_seq1 | 1092 | G | G->T | G->T | G->T | - | - | - |
| comp11729_c0_seq1 | 198 | G | G->T | G->T | G->T | - | - | - |
| comp35616_c0_seq1 | 5006 | G | G->T | G->T | G->T | - | - | - |
| comp30793_c0_seq1 | 1554 | G | G->T | G->T | G->T | - | - | - |
| comp17570_c0_seq1 | 523 | G | G->T | G->T | G->T | - | - | - |
| comp22782_c0_seq1 | 237 | G | G->T | G->T | G->T | - | - | - |
| comp26881_c0_seq1 | 833 | G | G->T | G->T | G->T | - | - | - |
| comp19265_c0_seq1 | 486 | G | G->T | G->T | G->T | - | - | - |
| comp20256_c0_seq1 | 514 | G | G->T | G->T | G->T | - | - | - |
| comp13425_c0_seq1 | 124 | G | G->T | G->T | G->T | - | - | - |
| comp11699_c0_seq1 | 954 | G | G->T | G->T | G->T | - | - | - |
| comp24670_c0_seq1 | 32 | G | G->T | G->T | G->T | - | - | - |
| comp12777_c0_seq1 | 154 | G | G->T | G->T | G->T | - | - | - |
| comp1739_c0_seq1 | 106 | G | G->T | G->T | G->T | - | - | - |
| comp36828_c0_seq2 | 2963 | G | G->C | G->C | G->C | - | - | - |
| comp21199_c0_seq1 | 235 | G | G->C | G->C | G->C | - | - | - |
| comp36732_c1_seq8 | 58 | G | G->C | G->C | G->C | - | - | - |
| comp1099562_c0_seq1 | 176 | G | G->C | G->C | G->C | - | - | - |
| comp36934_c0_seq3 | 316 | G | G->C | G->C | G->C | - | - | - |
| comp28710_c0_seq1 | 32 | G | G->C | G->C | G->C | - | - | - |
| comp857739_c0_seq1 | 25 | G | G->C | G->C | G->C | - | - | - |
| comp12230_c0_seq1 | 314 | G | G->C | G->C | G->C | - | - | - |
| comp37004_c0_seq2 | 1076 | G | G->C | G->C | G->C | - | - | - |
| comp13897_c0_seq1 | 207 | G | G->C | G->C | G->C | - | - | - |
| comp1077_c0_seq1 | 242 | G | G->C | G->C | G->C | - | - | - |
| comp12230_c0_seq1 | 369 | G | G->C | G->C | G->C | - | - | - |
| comp11833_c0_seq1 | 5 | G | G->C | G->C | G->C | - | - | - |
| comp15316_c0_seq1 | 690 | G | G->C | G->C | G->C | - | - | - |
| comp16126_c0_seq1 | 172 | G | G->C | G->C | G->C | - | - | - |
| comp16918_c0_seq1 | 109 | G | G->C | G->C | G->C | - | - | - |
| comp17279_c0_seq1 | 29 | G | G->C | G->C | G->C | - | - | - |
| comp19872_c0_seq1 | 59 | G | G->C | G->C | G->C | - | - | - |
| comp35114_c0_seq1 | 3366 | G | G->C | G->C | G->C | - | - | - |
| comp965594_c0_seq1 | 166 | G | G->C | G->C | G->C | - | - | - |
| comp21030_c2_seq1 | 106 | G | G->C | G->C | G->C | - | - | - |
| comp12043_c0_seq1 | 339 | G | G->C | G->C | G->C | - | - | - |
| comp16006_c1_seq1 | 144 | G | G->C | G->C | G->C | - | - | - |
| comp36415_c0_seq5 | 768 | G | G->C | G->C | G->C | - | - | - |
| comp24381_c0_seq1 | 1777 | G | G->C | G->C | G->C | - | - | - |
| comp31997_c0_seq1 | 2593 | G | G->C | G->C | G->C | - | - | - |
| comp16320_c0_seq1 | 339 | G | G->C | G->C | G->C | - | - | - |
| comp24670_c0_seq1 | 44 | G | G->C | G->C | G->C | - | - | - |
| comp996794_c0_seq1 | 195 | G | G->C | G->C | G->C | - | - | - |
| comp11699_c0_seq1 | 1089 | G | G->C | G->C | G->C | - | - | - |
| comp11699_c0_seq1 | 1103 | G | G->C | G->C | G->C | - | - | - |
| comp36828_c0_seq2 | 3835 | G | G->C | G->C | G->C | - | - | - |
| comp35994_c0_seq40 | 402 | G | G->C | G->C | G->C | - | - | - |
| comp13832_c0_seq1 | 1177 | G | G->C | G->C | G->C | - | - | - |
| comp13288_c0_seq1 | 1734 | G | G->C | G->C | G->C | - | - | - |
| comp25359_c0_seq1 | 96 | G | G->C | G->C | G->C | - | - | - |
| comp12765_c0_seq1 | 81 | G | G->C | G->C | G->C | - | - | - |
| comp11699_c0_seq1 | 1185 | G | G->C | G->C | G->C | - | - | - |
| comp11699_c0_seq1 | 1032 | G | G->C | G->C | G->C | - | - | - |
| comp11699_c0_seq1 | 1167 | G | G->C | G->C | G->C | - | - | - |
| comp11699_c0_seq1 | 1035 | G | G->C | G->C | G->C | - | - | - |
| comp16918_c0_seq1 | 99 | G | G->C | G->C | G->C | - | - | - |
| comp210_c0_seq1 | 51 | G | G->A | G->A | G->A | - | - | - |
| comp16006_c1_seq1 | 24 | G | G->A | G->A | G->A | - | - | - |
| comp29055_c0_seq2 | 298 | G | G->A | G->A | G->A | - | - | - |
| comp15137_c0_seq1 | 426 | G | G->A | G->A | G->A | - | - | - |
| comp36923_c0_seq40 | 1449 | G | G->A | G->A | G->A | - | - | - |
| comp15799_c0_seq1 | 239 | G | G->A | G->A | G->A | - | - | - |
| comp29466_c0_seq1 | 895 | G | G->A | G->A | G->A | - | - | - |
| comp14196_c0_seq1 | 167 | G | G->A | G->A | G->A | - | - | - |
| comp17376_c0_seq1 | 265 | G | G->A | G->A | G->A | - | - | - |
| comp28406_c0_seq1 | 1621 | G | G->A | G->A | G->A | - | - | - |
| comp11699_c0_seq1 | 282 | G | G->A | G->A | G->A | - | - | - |
| comp24670_c0_seq1 | 40 | G | G->A | G->A | G->A | - | - | - |
| comp1026_c0_seq1 | 176 | G | G->A | G->A | G->A | - | - | - |
| comp34273_c0_seq1 | 584 | G | G->A | G->A | G->A | - | - | - |
| comp31407_c0_seq1 | 104 | G | G->A | G->A | G->A | - | - | - |
| comp15137_c0_seq1 | 360 | G | G->A | G->A | G->A | - | - | - |
| comp11699_c0_seq1 | 939 | G | G->A | G->A | G->A | - | - | - |
| comp12791_c0_seq1 | 272 | G | G->A | G->A | G->A | - | - | - |
| comp11866_c0_seq1 | 717 | G | G->A | G->A | G->A | - | - | - |
| comp1338_c0_seq1 | 96 | G | G->A | G->A | G->A | - | - | - |
| comp14180_c0_seq1 | 229 | G | G->A | G->A | G->A | - | - | - |
| comp21272_c0_seq1 | 617 | G | G->A | G->A | G->A | - | - | - |
| comp36766_c0_seq8 | 1289 | G | G->A | G->A | G->A | - | - | - |
| comp23138_c0_seq1 | 47 | G | G->A | G->A | G->A | - | - | - |
| comp18590_c0_seq1 | 483 | G | G->A | G->A | G->A | - | - | - |
| comp19009_c0_seq1 | 117 | G | G->A | G->A | G->A | - | - | - |
| comp28826_c1_seq3 | 1054 | G | G->A | G->A | G->A | - | - | - |
| comp16366_c0_seq1 | 380 | G | G->A | G->A | G->A | - | - | - |
| comp15137_c0_seq1 | 378 | G | G->A | G->A | G->A | - | - | - |
| comp10398_c0_seq1 | 239 | G | G->A | G->A | G->A | - | - | - |
| comp15799_c0_seq1 | 312 | G | G->A | G->A | G->A | - | - | - |
| comp27260_c1_seq1 | 484 | G | G->A | G->A | G->A | - | - | - |
| comp17536_c0_seq1 | 114 | G | G->A | G->A | G->A | - | - | - |
| comp28427_c0_seq1 | 126 | G | G->A | G->A | G->A | - | - | - |
| comp29741_c0_seq2 | 558 | G | G->A | G->A | G->A | - | - | - |
| comp32188_c0_seq1 | 266 | G | G->A | G->A | G->A | - | - | - |
| comp10146_c0_seq1 | 35 | G | G->A | G->A | G->A | - | - | - |
| comp1699_c0_seq1 | 259 | G | G->A | G->A | G->A | - | - | - |
| comp15137_c0_seq1 | 375 | G | G->A | G->A | G->A | - | - | - |
| comp36859_c0_seq3 | 261 | G | G->A | G->A | G->A | - | - | - |
| comp13259_c0_seq1 | 144 | G | G->A | G->A | G->A | - | - | - |
| comp17485_c0_seq1 | 245 | G | G->A | G->A | G->A | - | - | - |
| comp36017_c0_seq1 | 3957 | G | G->A | G->A | G->A | - | - | - |
| comp31036_c0_seq1 | 2379 | G | G->A | G->A | G->A | - | - | - |
| comp34458_c1_seq9 | 726 | G | G->A | G->A | G->A | - | - | - |
| comp35974_c0_seq3 | 1305 | G | G->A | G->A | G->A | - | - | - |
| comp4866_c0_seq1 | 468 | G | G->A | G->A | G->A | - | - | - |
| comp34358_c0_seq1 | 68 | G | G->A | G->A | G->A | - | - | - |
| comp15469_c0_seq1 | 935 | G | G->A | G->A | G->A | - | - | - |
| comp14649_c0_seq1 | 321 | G | G->A | G->A | G->A | - | - | - |
| comp33634_c0_seq4 | 340 | G | G->A | G->A | G->A | - | - | - |
| comp28391_c0_seq2 | 1455 | G | G->A | G->A | G->A | - | - | - |
| comp29741_c0_seq2 | 492 | G | G->A | G->A | G->A | - | - | - |
| comp28752_c0_seq1 | 413 | G | G->A | G->A | G->A | - | - | - |
| comp16437_c0_seq1 | 1080 | G | G->A | G->A | G->A | - | - | - |
| comp17321_c0_seq1 | 222 | G | G->A | G->A | G->A | - | - | - |
| comp10396_c0_seq1 | 678 | G | G->A | G->A | G->A | - | - | - |
| comp35562_c0_seq1 | 2753 | G | G->A | G->A | G->A | - | - | - |
| comp17153_c0_seq1 | 588 | G | G->A | G->A | G->A | - | - | - |
| comp34397_c0_seq1 | 3049 | G | G->A | G->A | G->A | - | - | - |
| comp32286_c0_seq1 | 1290 | G | G->A | G->A | G->A | - | - | - |
| comp34126_c0_seq1 | 2890 | G | G->A | G->A | G->A | - | - | - |
| comp32279_c0_seq1 | 2257 | G | G->A | G->A | G->A | - | - | - |
| comp33772_c0_seq8 | 649 | G | G->A | G->A | G->A | - | - | - |
| comp12418_c0_seq1 | 759 | G | G->A | G->A | G->A | - | - | - |
| comp35137_c0_seq1 | 1205 | G | G->A | G->A | G->A | - | - | - |
| comp17455_c0_seq1 | 626 | G | G->A | G->A | G->A | - | - | - |
| comp34060_c0_seq1 | 657 | G | G->A | G->A | G->A | - | - | - |
| comp31963_c0_seq1 | 785 | G | G->A | G->A | G->A | - | - | - |
| comp27565_c0_seq3 | 616 | G | G->A | G->A | G->A | - | - | - |
| comp16966_c0_seq1 | 958 | G | G->A | G->A | G->A | - | - | - |
| comp17236_c0_seq1 | 276 | G | G->A | G->A | G->A | - | - | - |
| comp14843_c0_seq1 | 303 | G | G->A | G->A | G->A | - | - | - |
| comp28755_c0_seq2 | 468 | G | G->A | G->A | G->A | - | - | - |
| comp817756_c0_seq1 | 218 | G | G->A | G->A | G->A | - | - | - |
| comp13290_c0_seq1 | 203 | G | G->A | G->A | G->A | - | - | - |
| comp17536_c0_seq1 | 197 | G | G->A | G->A | G->A | - | - | - |
| comp415226_c0_seq1 | 43 | G | G->A | G->A | G->A | - | - | - |
| comp809001_c0_seq1 | 218 | G | G->A | G->A | G->A | - | - | - |
| comp17462_c0_seq2 | 1170 | G | G->A | G->A | G->A | - | - | - |
| comp36017_c0_seq1 | 1503 | G | G->A | G->A | G->A | - | - | - |
| comp36587_c0_seq4 | 532 | G | G->A | G->A | G->A | - | - | - |
| comp1489_c0_seq1 | 51 | G | G->A | G->A | G->A | - | - | - |
| comp1053913_c0_seq1 | 193 | G | G->A | G->A | G->A | - | - | - |
| comp33835_c0_seq1 | 1554 | G | G->A | G->A | G->A | - | - | - |
| comp17625_c1_seq1 | 281 | G | G->A | G->A | G->A | - | - | - |
| comp1430_c0_seq1 | 282 | G | G->A | G->A | G->A | - | - | - |
| comp36998_c0_seq5 | 4416 | G | G->A | G->A | G->A | - | - | - |
| comp1484193_c0_seq1 | 168 | G | G->A | G->A | G->A | - | - | - |
| comp16652_c0_seq1 | 261 | G | G->A | G->A | G->A | - | - | - |
| comp14308_c0_seq1 | 505 | G | G->A | G->A | G->A | - | - | - |
| comp36239_c0_seq9 | 34 | G | G->A | G->A | G->A | - | - | - |
| comp8130_c0_seq1 | 375 | G | G->A | G->A | G->A | - | - | - |
| comp36788_c0_seq4 | 11212 | G | G->A | G->A | G->A | - | - | - |
| comp30993_c0_seq1 | 364 | G | G->A | G->A | G->A | - | - | - |
| comp13133_c0_seq1 | 72 | G | G->A | G->A | G->A | - | - | - |
| comp17590_c0_seq1 | 42 | G | G->A | G->A | G->A | - | - | - |
| comp20333_c0_seq1 | 25 | G | G->A | G->A | G->A | - | - | - |
| comp23431_c0_seq1 | 319 | G | G->A | G->A | G->A | - | - | - |
| comp6335_c0_seq1 | 26 | G | G->A | G->A | G->A | - | - | - |
| comp29207_c0_seq1 | 504 | G | G->A | G->A | G->A | - | - | - |
| comp36260_c1_seq1 | 21 | G | G->A | G->A | G->A | - | - | - |
| comp36645_c0_seq7 | 4349 | G | G->A | G->A | G->A | - | - | - |
| comp8288_c0_seq1 | 570 | G | G->A | G->A | G->A | - | - | - |
| comp17753_c0_seq2 | 51 | G | G->A | G->A | G->A | - | - | - |
| comp15832_c0_seq1 | 159 | G | G->A | G->A | G->A | - | - | - |
| comp29598_c3_seq1 | 1497 | G | G->A | G->A | G->A | - | - | - |
| comp17910_c1_seq1 | 847 | G | G->A | G->A | G->A | - | - | - |
| comp36998_c0_seq5 | 4435 | G | G->A | G->A | G->A | - | - | - |
| comp25750_c0_seq1 | 222 | G | G->A | G->A | G->A | - | - | - |
| comp16167_c0_seq1 | 249 | G | G->A | G->A | G->A | - | - | - |
| comp22283_c2_seq1 | 113 | G | G->A | G->A | G->A | - | - | - |
| comp13311_c0_seq1 | 209 | G | G->A | G->A | G->A | - | - | - |
| comp34765_c0_seq1 | 2440 | G | G->A | G->A | G->A | - | - | - |
| comp22063_c0_seq1 | 1594 | G | G->A | G->A | G->A | - | - | - |
| comp24333_c0_seq1 | 3043 | G | G->A | G->A | G->A | - | - | - |
| comp22823_c1_seq1 | 1038 | G | G->A | G->A | G->A | - | - | - |
| comp15026_c0_seq1 | 1736 | G | G->A | G->A | G->A | - | - | - |
| comp433554_c0_seq1 | 243 | G | G->A | G->A | G->A | - | - | - |
| comp20313_c0_seq1 | 1940 | G | G->A | G->A | G->A | - | - | - |
| comp13658_c0_seq1 | 1112 | G | G->A | G->A | G->A | - | - | - |
| comp10400_c0_seq1 | 501 | G | G->A | G->A | G->A | - | - | - |
| comp20782_c0_seq1 | 161 | G | G->A | G->A | G->A | - | - | - |
| comp26425_c1_seq1 | 1198 | G | G->A | G->A | G->A | - | - | - |
| comp819551_c0_seq1 | 322 | G | G->A | G->A | G->A | - | - | - |
| comp1338_c0_seq1 | 154 | G | G->A | G->A | G->A | - | - | - |
| comp29741_c0_seq2 | 452 | G | G->A | G->A | G->A | - | - | - |
| comp10933_c0_seq1 | 61 | G | G->A | G->A | G->A | - | - | - |
| comp819551_c0_seq1 | 275 | G | G->A | G->A | G->A | - | - | - |
| comp6326_c0_seq1 | 956 | G | G->A | G->A | G->A | - | - | - |
| comp34555_c0_seq1 | 2354 | G | G->A | G->A | G->A | - | - | - |
| comp20587_c0_seq1 | 1302 | G | G->A | G->A | G->A | - | - | - |
| comp34441_c1_seq2 | 5006 | G | G->A | G->A | G->A | - | - | - |
| comp13396_c0_seq1 | 333 | G | G->A | G->A | G->A | - | - | - |
| comp15294_c0_seq1 | 145 | G | G->A | G->A | G->A | - | - | - |
| comp34535_c0_seq8 | 4 | G | G->A | G->A | G->A | - | - | - |
| comp14946_c1_seq1 | 47 | G | G->A | G->A | G->A | - | - | - |
| comp16768_c0_seq1 | 166 | G | G->A | G->A | G->A | - | - | - |
| comp14213_c0_seq1 | 82 | G | G->A | G->A | G->A | - | - | - |
| comp16006_c1_seq1 | 108 | G | G->A | G->A | G->A | - | - | - |
| comp30260_c1_seq1 | 3793 | G | G->A | G->A | G->A | - | - | - |
| comp15154_c0_seq1 | 222 | G | G->A | G->A | G->A | - | - | - |
| comp35956_c1_seq1 | 62 | G | G->A | G->A | G->A | - | - | - |
| comp16918_c0_seq1 | 121 | G | G->A | G->A | G->A | - | - | - |
| comp35658_c0_seq1 | 996 | G | G->A | G->A | G->A | - | - | - |
| comp18047_c0_seq1 | 1412 | G | G->A | G->A | G->A | - | - | - |
| comp1338_c0_seq1 | 79 | G | G->A | G->A | G->A | - | - | - |
| comp16006_c1_seq1 | 110 | G | G->A | G->A | G->A | - | - | - |
| comp775150_c0_seq1 | 611 | G | G->A | G->A | G->A | - | - | - |
| comp1722_c0_seq1 | 590 | G | G->A | G->A | G->A | - | - | - |
| comp120974_c0_seq1 | 339 | G | G->A | G->A | G->A | - | - | - |
| comp31_c1_seq1 | 218 | G | G->A | G->A | G->A | - | - | - |
| comp1084466_c0_seq1 | 151 | G | G->A | G->A | G->A | - | - | - |
| comp13897_c0_seq1 | 229 | G | G->A | G->A | G->A | - | - | - |
| comp14156_c0_seq1 | 335 | G | G->A | G->A | G->A | - | - | - |
| comp36828_c0_seq2 | 3511 | G | G->A | G->A | G->A | - | - | - |
| comp22159_c1_seq1 | 58 | G | G->A | G->A | G->A | - | - | - |
| comp27720_c0_seq1 | 508 | G | G->A | G->A | G->A | - | - | - |
| comp27720_c0_seq1 | 491 | G | G->A | G->A | G->A | - | - | - |
| comp17535_c1_seq1 | 16 | G | G->A | G->A | G->A | - | - | - |
| comp32618_c0_seq4 | 833 | G | G->A | G->A | G->A | - | - | - |
| comp33021_c0_seq2 | 505 | G | G->A | G->A | G->A | - | - | - |
| comp36842_c0_seq3 | 2067 | G | G->A | G->A | G->A | - | - | - |
| comp1099562_c0_seq1 | 144 | G | G->A | G->A | G->A | - | - | - |
| comp36746_c0_seq5 | 105 | G | G->A | G->A | G->A | - | - | - |
| comp1107799_c0_seq1 | 179 | G | G->A | G->A | G->A | - | - | - |
| comp12230_c0_seq1 | 355 | G | G->A | G->A | G->A | - | - | - |
| comp17412_c0_seq1 | 428 | G | G->A | G->A | G->A | - | - | - |
| comp12230_c0_seq1 | 382 | G | G->A | G->A | G->A | - | - | - |
| comp36448_c0_seq5 | 760 | G | G->A | G->A | G->A | - | - | - |
| comp36982_c0_seq7 | 225 | G | G->A | G->A | G->A | - | - | - |
| comp1099562_c0_seq1 | 221 | G | G->A | G->A | G->A | - | - | - |
| comp12524_c0_seq1 | 3 | G | G->A | G->A | G->A | - | - | - |
| comp25395_c1_seq1 | 605 | G | G->A | G->A | G->A | - | - | - |
| comp2987_c0_seq1 | 37 | G | G->A | G->A | G->A | - | - | - |
| comp22557_c0_seq1 | 1957 | G | G->A | G->A | G->A | - | - | - |
| comp36775_c0_seq1 | 693 | G | G->A | G->A | G->A | - | - | - |
| comp12524_c0_seq1 | 9 | G | G->A | G->A | G->A | - | - | - |
| comp1052985_c0_seq1 | 31 | G | G->A | G->A | G->A | - | - | - |
| comp12524_c0_seq1 | 16 | G | G->A | G->A | G->A | - | - | - |
| comp35904_c0_seq12 | 1223 | G | G->A | G->A | G->A | - | - | - |
| comp32483_c0_seq3 | 176 | G | G->A | G->A | G->A | - | - | - |
| comp17955_c1_seq1 | 113 | G | G->A | G->A | G->A | - | - | - |
| comp36802_c0_seq4 | 6 | G | G->A | G->A | G->A | - | - | - |
| comp15380_c1_seq1 | 31 | G | G->A | G->A | G->A | - | - | - |
| comp17639_c1_seq1 | 294 | G | G->A | G->A | G->A | - | - | - |
| comp18352_c0_seq1 | 341 | G | G->A | G->A | G->A | - | - | - |
| comp25395_c1_seq1 | 503 | G | G->A | G->A | G->A | - | - | - |
| comp1052985_c0_seq1 | 24 | G | G->A | G->A | G->A | - | - | - |
| comp35739_c0_seq1 | 462 | G | G->A | G->A | G->A | - | - | - |
| comp32386_c0_seq1 | 26 | G | G->A | G->A | G->A | - | - | - |
| comp666624_c0_seq1 | 21 | G | G->A | G->A | G->A | - | - | - |
| comp36587_c0_seq4 | 480 | G | G->A | G->A | G->A | - | - | - |
| comp36139_c0_seq5 | 656 | G | G->A | G->A | G->A | - | - | - |
| comp36814_c0_seq13 | 561 | G | G->A | G->A | G->A | - | - | - |
| comp36842_c0_seq3 | 1694 | G | G->A | G->A | G->A | - | - | - |
| comp28399_c0_seq3 | 573 | G | G->A | G->A | G->A | - | - | - |
| comp652554_c0_seq1 | 21 | G | G->A | G->A | G->A | - | - | - |
| comp12194_c0_seq1 | 114 | G | G->A | G->A | G->A | - | - | - |
| comp36828_c0_seq2 | 3053 | G | G->A | G->A | G->A | - | - | - |
| comp36982_c0_seq25 | 51 | G | G->A | G->A | G->A | - | - | - |
| comp36982_c0_seq25 | 144 | G | G->A | G->A | G->A | - | - | - |
| comp30024_c0_seq1 | 8 | G | G->A | G->A | G->A | - | - | - |
| comp1756_c0_seq1 | 217 | G | G->A | G->A | G->A | - | - | - |
| comp36842_c0_seq3 | 582 | G | G->A | G->A | G->A | - | - | - |
| comp36828_c0_seq2 | 2936 | G | G->A | G->A | G->A | - | - | - |
| comp36828_c0_seq2 | 1996 | G | G->A | G->A | G->A | - | - | - |
| comp36828_c0_seq2 | 1990 | G | G->A | G->A | G->A | - | - | - |
| comp10099_c0_seq1 | 219 | G | G->A | G->A | G->A | - | - | - |
| comp120974_c0_seq1 | 1 | G | G->A | G->A | G->A | - | - | - |
| comp26262_c2_seq1 | 2801 | G | G->A | G->A | G->A | - | - | - |
| comp36842_c0_seq3 | 1601 | T | T->G | T->G | T->G | - | - | - |
| comp36732_c1_seq8 | 100 | T | T->G | T->G | T->G | - | - | - |
| comp34466_c0_seq2 | 46 | T | T->G | T->G | T->G | - | - | - |
| comp14977_c0_seq1 | 24 | T | T->G | T->G | T->G | - | - | - |
| comp1052985_c0_seq1 | 20 | T | T->G | T->G | T->G | - | - | - |
| comp958_c0_seq1 | 36 | T | T->G | T->G | T->G | - | - | - |
| comp36139_c0_seq5 | 709 | T | T->G | T->G | T->G | - | - | - |
| comp35919_c0_seq11 | 178 | T | T->G | T->G | T->G | - | - | - |
| comp694013_c0_seq1 | 152 | T | T->G | T->G | T->G | - | - | - |
| comp36461_c1_seq1 | 33 | T | T->G | T->G | T->G | - | - | - |
| comp36934_c0_seq3 | 369 | T | T->G | T->G | T->G | - | - | - |
| comp10099_c0_seq1 | 273 | T | T->G | T->G | T->G | - | - | - |
| comp36653_c0_seq5 | 995 | T | T->G | T->G | T->G | - | - | - |
| comp36842_c0_seq3 | 2047 | T | T->G | T->G | T->G | - | - | - |
| comp958_c0_seq1 | 43 | T | T->G | T->G | T->G | - | - | - |
| comp958_c0_seq1 | 44 | T | T->G | T->G | T->G | - | - | - |
| comp1099562_c0_seq1 | 122 | T | T->G | T->G | T->G | - | - | - |
| comp36391_c1_seq7 | 3 | T | T->G | T->G | T->G | - | - | - |
| comp28762_c0_seq1 | 292 | T | T->G | T->G | T->G | - | - | - |
| comp12751_c0_seq1 | 43 | T | T->G | T->G | T->G | - | - | - |
| comp15430_c0_seq1 | 135 | T | T->G | T->G | T->G | - | - | - |
| comp16918_c0_seq1 | 120 | T | T->G | T->G | T->G | - | - | - |
| comp16006_c1_seq1 | 126 | T | T->G | T->G | T->G | - | - | - |
| comp13123_c0_seq1 | 221 | T | T->G | T->G | T->G | - | - | - |
| comp14779_c0_seq1 | 638 | T | T->G | T->G | T->G | - | - | - |
| comp16918_c0_seq1 | 103 | T | T->G | T->G | T->G | - | - | - |
| comp34587_c1_seq5 | 459 | T | T->G | T->G | T->G | - | - | - |
| comp18364_c0_seq1 | 542 | T | T->G | T->G | T->G | - | - | - |
| comp14848_c0_seq1 | 54 | T | T->G | T->G | T->G | - | - | - |
| comp857739_c0_seq1 | 47 | T | T->G | T->G | T->G | - | - | - |
| comp16846_c0_seq1 | 246 | T | T->G | T->G | T->G | - | - | - |
| comp23238_c0_seq1 | 40 | T | T->G | T->G | T->G | - | - | - |
| comp33338_c0_seq1 | 2702 | T | T->G | T->G | T->G | - | - | - |
| comp1052985_c0_seq1 | 17 | T | T->G | T->G | T->G | - | - | - |
| comp1052985_c0_seq1 | 18 | T | T->G | T->G | T->G | - | - | - |
| comp36982_c0_seq7 | 163 | T | T->G | T->G | T->G | - | - | - |
| comp11699_c0_seq1 | 1014 | T | T->G | T->G | T->G | - | - | - |
| comp17536_c0_seq1 | 136 | T | T->G | T->G | T->G | - | - | - |
| comp17543_c0_seq1 | 142 | T | T->G | T->G | T->G | - | - | - |
| comp13042_c0_seq1 | 153 | T | T->G | T->G | T->G | - | - | - |
| comp16666_c0_seq1 | 437 | T | T->G | T->G | T->G | - | - | - |
| comp23404_c0_seq1 | 821 | T | T->G | T->G | T->G | - | - | - |
| comp22837_c0_seq1 | 74 | T | T->G | T->G | T->G | - | - | - |
| comp14601_c0_seq1 | 257 | T | T->G | T->G | T->G | - | - | - |
| comp24881_c0_seq1 | 116 | T | T->G | T->G | T->G | - | - | - |
| comp16077_c0_seq1 | 81 | T | T->G | T->G | T->G | - | - | - |
| comp10098_c0_seq1 | 127 | T | T->G | T->G | T->G | - | - | - |
| comp3187_c0_seq1 | 334 | T | T->G | T->G | T->G | - | - | - |
| comp10933_c0_seq1 | 37 | T | T->G | T->G | T->G | - | - | - |
| comp15971_c0_seq1 | 55 | T | T->C | T->C | T->C | - | - | - |
| comp13897_c0_seq1 | 169 | T | T->C | T->C | T->C | - | - | - |
| comp12230_c0_seq1 | 354 | T | T->C | T->C | T->C | - | - | - |
| comp13897_c0_seq1 | 287 | T | T->C | T->C | T->C | - | - | - |
| comp12230_c0_seq1 | 331 | T | T->C | T->C | T->C | - | - | - |
| comp1052985_c0_seq1 | 21 | T | T->C | T->C | T->C | - | - | - |
| comp36732_c1_seq8 | 103 | T | T->C | T->C | T->C | - | - | - |
| comp12230_c0_seq1 | 350 | T | T->C | T->C | T->C | - | - | - |
| comp10099_c0_seq1 | 305 | T | T->C | T->C | T->C | - | - | - |
| comp36587_c0_seq4 | 466 | T | T->C | T->C | T->C | - | - | - |
| comp10099_c0_seq1 | 290 | T | T->C | T->C | T->C | - | - | - |
| comp35904_c0_seq12 | 1203 | T | T->C | T->C | T->C | - | - | - |
| comp32483_c0_seq3 | 88 | T | T->C | T->C | T->C | - | - | - |
| comp25395_c1_seq1 | 467 | T | T->C | T->C | T->C | - | - | - |
| comp8708_c0_seq1 | 96 | T | T->C | T->C | T->C | - | - | - |
| comp1052985_c0_seq1 | 22 | T | T->C | T->C | T->C | - | - | - |
| comp10099_c0_seq1 | 285 | T | T->C | T->C | T->C | - | - | - |
| comp32549_c0_seq3 | 72 | T | T->C | T->C | T->C | - | - | - |
| comp1052985_c0_seq1 | 30 | T | T->C | T->C | T->C | - | - | - |
| comp10099_c0_seq1 | 264 | T | T->C | T->C | T->C | - | - | - |
| comp1052985_c0_seq1 | 32 | T | T->C | T->C | T->C | - | - | - |
| comp33386_c0_seq2 | 118 | T | T->C | T->C | T->C | - | - | - |
| comp36934_c0_seq3 | 343 | T | T->C | T->C | T->C | - | - | - |
| comp12194_c0_seq1 | 85 | T | T->C | T->C | T->C | - | - | - |
| comp10117_c0_seq1 | 49 | T | T->C | T->C | T->C | - | - | - |
| comp8274_c0_seq1 | 217 | T | T->C | T->C | T->C | - | - | - |
| comp1084466_c0_seq1 | 190 | T | T->C | T->C | T->C | - | - | - |
| comp12230_c0_seq1 | 351 | T | T->C | T->C | T->C | - | - | - |
| comp15013_c0_seq1 | 561 | T | T->C | T->C | T->C | - | - | - |
| comp15013_c0_seq2 | 512 | T | T->C | T->C | T->C | - | - | - |
| comp10099_c0_seq1 | 242 | T | T->C | T->C | T->C | - | - | - |
| comp36828_c0_seq2 | 4109 | T | T->C | T->C | T->C | - | - | - |
| comp37004_c0_seq2 | 647 | T | T->C | T->C | T->C | - | - | - |
| comp36842_c0_seq3 | 1551 | T | T->C | T->C | T->C | - | - | - |
| comp12230_c0_seq1 | 320 | T | T->C | T->C | T->C | - | - | - |
| comp10099_c0_seq1 | 233 | T | T->C | T->C | T->C | - | - | - |
| comp36842_c0_seq3 | 1506 | T | T->C | T->C | T->C | - | - | - |
| comp10099_c0_seq1 | 321 | T | T->C | T->C | T->C | - | - | - |
| comp16632_c0_seq1 | 428 | T | T->C | T->C | T->C | - | - | - |
| comp10099_c0_seq1 | 238 | T | T->C | T->C | T->C | - | - | - |
| comp12751_c0_seq1 | 50 | T | T->C | T->C | T->C | - | - | - |
| comp1099562_c0_seq1 | 215 | T | T->C | T->C | T->C | - | - | - |
| comp36842_c0_seq3 | 983 | T | T->C | T->C | T->C | - | - | - |
| comp36828_c0_seq2 | 2258 | T | T->C | T->C | T->C | - | - | - |
| comp1099562_c0_seq1 | 171 | T | T->C | T->C | T->C | - | - | - |
| comp933796_c0_seq1 | 121 | T | T->C | T->C | T->C | - | - | - |
| comp36982_c0_seq7 | 174 | T | T->C | T->C | T->C | - | - | - |
| comp33289_c0_seq1 | 108 | T | T->C | T->C | T->C | - | - | - |
| comp36828_c0_seq2 | 3822 | T | T->C | T->C | T->C | - | - | - |
| comp36828_c0_seq2 | 3797 | T | T->C | T->C | T->C | - | - | - |
| comp5422_c0_seq1 | 285 | T | T->C | T->C | T->C | - | - | - |
| comp33630_c1_seq1 | 2022 | T | T->C | T->C | T->C | - | - | - |
| comp1109629_c0_seq1 | 174 | T | T->C | T->C | T->C | - | - | - |
| comp12857_c0_seq1 | 219 | T | T->C | T->C | T->C | - | - | - |
| comp36923_c0_seq40 | 1448 | T | T->C | T->C | T->C | - | - | - |
| comp1338_c0_seq1 | 103 | T | T->C | T->C | T->C | - | - | - |
| comp1338_c0_seq1 | 85 | T | T->C | T->C | T->C | - | - | - |
| comp16006_c1_seq1 | 51 | T | T->C | T->C | T->C | - | - | - |
| comp16966_c0_seq1 | 1286 | T | T->C | T->C | T->C | - | - | - |
| comp11699_c0_seq1 | 960 | T | T->C | T->C | T->C | - | - | - |
| comp16006_c1_seq1 | 105 | T | T->C | T->C | T->C | - | - | - |
| comp36988_c0_seq1 | 4675 | T | T->C | T->C | T->C | - | - | - |
| comp17321_c0_seq1 | 192 | T | T->C | T->C | T->C | - | - | - |
| comp11699_c0_seq1 | 951 | T | T->C | T->C | T->C | - | - | - |
| comp11699_c0_seq1 | 1062 | T | T->C | T->C | T->C | - | - | - |
| comp34283_c0_seq2 | 63 | T | T->C | T->C | T->C | - | - | - |
| comp16006_c1_seq1 | 48 | T | T->C | T->C | T->C | - | - | - |
| comp263865_c0_seq1 | 268 | T | T->C | T->C | T->C | - | - | - |
| comp16006_c1_seq1 | 142 | T | T->C | T->C | T->C | - | - | - |
| comp13534_c0_seq1 | 1561 | T | T->C | T->C | T->C | - | - | - |
| comp16006_c1_seq1 | 132 | T | T->C | T->C | T->C | - | - | - |
| comp11699_c0_seq1 | 885 | T | T->C | T->C | T->C | - | - | - |
| comp29286_c0_seq1 | 1326 | T | T->C | T->C | T->C | - | - | - |
| comp31068_c0_seq2 | 3639 | T | T->C | T->C | T->C | - | - | - |
| comp29845_c0_seq2 | 1165 | T | T->C | T->C | T->C | - | - | - |
| comp10400_c0_seq1 | 506 | T | T->C | T->C | T->C | - | - | - |
| comp11699_c0_seq1 | 957 | T | T->C | T->C | T->C | - | - | - |
| comp36329_c0_seq3 | 234 | T | T->C | T->C | T->C | - | - | - |
| comp589143_c0_seq1 | 170 | T | T->C | T->C | T->C | - | - | - |
| comp1131293_c0_seq1 | 195 | T | T->C | T->C | T->C | - | - | - |
| comp30843_c0_seq2 | 232 | T | T->C | T->C | T->C | - | - | - |
| comp30843_c0_seq2 | 228 | T | T->C | T->C | T->C | - | - | - |
| comp404799_c0_seq1 | 294 | T | T->C | T->C | T->C | - | - | - |
| comp1132626_c0_seq1 | 134 | T | T->C | T->C | T->C | - | - | - |
| comp31041_c0_seq4 | 692 | T | T->C | T->C | T->C | - | - | - |
| comp24185_c0_seq3 | 1194 | T | T->C | T->C | T->C | - | - | - |
| comp12505_c0_seq1 | 245 | T | T->C | T->C | T->C | - | - | - |
| comp35383_c0_seq1 | 826 | T | T->C | T->C | T->C | - | - | - |
| comp1241907_c0_seq1 | 233 | T | T->C | T->C | T->C | - | - | - |
| comp14684_c0_seq1 | 42 | T | T->C | T->C | T->C | - | - | - |
| comp18224_c1_seq1 | 313 | T | T->C | T->C | T->C | - | - | - |
| comp25003_c2_seq1 | 1404 | T | T->C | T->C | T->C | - | - | - |
| comp589040_c0_seq1 | 46 | T | T->C | T->C | T->C | - | - | - |
| comp1139824_c0_seq1 | 223 | T | T->C | T->C | T->C | - | - | - |
| comp18425_c0_seq1 | 669 | T | T->C | T->C | T->C | - | - | - |
| comp11699_c0_seq1 | 1161 | T | T->C | T->C | T->C | - | - | - |
| comp33591_c1_seq3 | 180 | T | T->C | T->C | T->C | - | - | - |
| comp1444849_c0_seq1 | 154 | T | T->C | T->C | T->C | - | - | - |
| comp8288_c0_seq1 | 542 | T | T->C | T->C | T->C | - | - | - |
| comp17279_c0_seq1 | 36 | T | T->C | T->C | T->C | - | - | - |
| comp21199_c0_seq1 | 206 | T | T->C | T->C | T->C | - | - | - |
| comp750210_c0_seq1 | 369 | T | T->C | T->C | T->C | - | - | - |
| comp17536_c0_seq1 | 145 | T | T->C | T->C | T->C | - | - | - |
| comp27446_c0_seq4 | 2500 | T | T->C | T->C | T->C | - | - | - |
| comp13403_c0_seq1 | 252 | T | T->C | T->C | T->C | - | - | - |
| comp1558_c0_seq1 | 22 | T | T->C | T->C | T->C | - | - | - |
| comp36842_c0_seq3 | 1305 | T | T->C | T->C | T->C | - | - | - |
| comp28710_c0_seq1 | 40 | T | T->C | T->C | T->C | - | - | - |
| comp22382_c0_seq1 | 1011 | T | T->C | T->C | T->C | - | - | - |
| comp16689_c0_seq1 | 545 | T | T->C | T->C | T->C | - | - | - |
| comp18466_c0_seq2 | 108 | T | T->C | T->C | T->C | - | - | - |
| comp13575_c0_seq1 | 1816 | T | T->C | T->C | T->C | - | - | - |
| comp16804_c1_seq1 | 472 | T | T->C | T->C | T->C | - | - | - |
| comp20581_c3_seq1 | 260 | T | T->C | T->C | T->C | - | - | - |
| comp15644_c0_seq1 | 313 | T | T->C | T->C | T->C | - | - | - |
| comp34060_c0_seq1 | 610 | T | T->C | T->C | T->C | - | - | - |
| comp16522_c0_seq1 | 151 | T | T->C | T->C | T->C | - | - | - |
| comp17131_c0_seq1 | 414 | T | T->C | T->C | T->C | - | - | - |
| comp13087_c0_seq1 | 659 | T | T->C | T->C | T->C | - | - | - |
| comp12678_c0_seq1 | 868 | T | T->C | T->C | T->C | - | - | - |
| comp20577_c1_seq1 | 463 | T | T->C | T->C | T->C | - | - | - |
| comp26431_c0_seq1 | 233 | T | T->C | T->C | T->C | - | - | - |
| comp35250_c0_seq3 | 1372 | T | T->C | T->C | T->C | - | - | - |
| comp16496_c1_seq2 | 370 | T | T->C | T->C | T->C | - | - | - |
| comp35659_c0_seq3 | 1021 | T | T->C | T->C | T->C | - | - | - |
| comp1338_c0_seq1 | 121 | T | T->C | T->C | T->C | - | - | - |
| comp15264_c0_seq2 | 305 | T | T->C | T->C | T->C | - | - | - |
| comp1338_c0_seq1 | 58 | T | T->C | T->C | T->C | - | - | - |
| comp15283_c1_seq1 | 531 | T | T->C | T->C | T->C | - | - | - |
| comp15058_c0_seq1 | 956 | T | T->C | T->C | T->C | - | - | - |
| comp427076_c0_seq1 | 1412 | T | T->C | T->C | T->C | - | - | - |
| comp11208_c0_seq1 | 317 | T | T->C | T->C | T->C | - | - | - |
| comp11699_c0_seq1 | 1086 | T | T->C | T->C | T->C | - | - | - |
| comp17705_c0_seq1 | 407 | T | T->C | T->C | T->C | - | - | - |
| comp17204_c0_seq1 | 105 | T | T->C | T->C | T->C | - | - | - |
| comp34769_c0_seq1 | 6620 | T | T->C | T->C | T->C | - | - | - |
| comp14180_c0_seq1 | 180 | T | T->C | T->C | T->C | - | - | - |
| comp16793_c1_seq1 | 483 | T | T->C | T->C | T->C | - | - | - |
| comp11833_c0_seq1 | 22 | T | T->C | T->C | T->C | - | - | - |
| comp20256_c0_seq1 | 535 | T | T->C | T->C | T->C | - | - | - |
| comp10933_c0_seq1 | 166 | T | T->C | T->C | T->C | - | - | - |
| comp11699_c0_seq1 | 1026 | T | T->C | T->C | T->C | - | - | - |
| comp11699_c0_seq1 | 1044 | T | T->C | T->C | T->C | - | - | - |
| comp10933_c0_seq1 | 127 | T | T->C | T->C | T->C | - | - | - |
| comp16918_c0_seq1 | 37 | T | T->C | T->C | T->C | - | - | - |
| comp16918_c0_seq1 | 118 | T | T->C | T->C | T->C | - | - | - |
| comp32288_c2_seq1 | 85 | T | T->C | T->C | T->C | - | - | - |
| comp16918_c0_seq1 | 105 | T | T->A | T->A | T->A | - | - | - |
| comp10933_c0_seq1 | 64 | T | T->A | T->A | T->A | - | - | - |
| comp10933_c0_seq1 | 109 | T | T->A | T->A | T->A | - | - | - |
| comp16918_c0_seq1 | 89 | T | T->A | T->A | T->A | - | - | - |
| comp24852_c0_seq1 | 105 | T | T->A | T->A | T->A | - | - | - |
| comp17536_c0_seq1 | 128 | T | T->A | T->A | T->A | - | - | - |
| comp17536_c0_seq1 | 115 | T | T->A | T->A | T->A | - | - | - |
| comp1695_c0_seq1 | 239 | T | T->A | T->A | T->A | - | - | - |
| comp35228_c0_seq2 | 161 | T | T->A | T->A | T->A | - | - | - |
| comp16309_c0_seq1 | 79 | T | T->A | T->A | T->A | - | - | - |
| comp16126_c0_seq1 | 86 | T | T->A | T->A | T->A | - | - | - |
| comp17277_c0_seq2 | 305 | T | T->A | T->A | T->A | - | - | - |
| comp17031_c0_seq1 | 531 | T | T->A | T->A | T->A | - | - | - |
| comp1558_c0_seq1 | 21 | T | T->A | T->A | T->A | - | - | - |
| comp36385_c0_seq2 | 517 | T | T->A | T->A | T->A | - | - | - |
| comp16077_c0_seq1 | 103 | T | T->A | T->A | T->A | - | - | - |
| comp10764_c0_seq1 | 129 | T | T->A | T->A | T->A | - | - | - |
| comp34687_c0_seq3 | 3933 | T | T->A | T->A | T->A | - | - | - |
| comp32426_c0_seq2 | 1565 | T | T->A | T->A | T->A | - | - | - |
| comp24871_c1_seq1 | 51 | T | T->A | T->A | T->A | - | - | - |
| comp36587_c0_seq4 | 534 | T | T->A | T->A | T->A | - | - | - |
| comp36788_c0_seq4 | 11161 | T | T->A | T->A | T->A | - | - | - |
| comp22226_c1_seq1 | 385 | T | T->A | T->A | T->A | - | - | - |
| comp14308_c0_seq1 | 498 | T | T->A | T->A | T->A | - | - | - |
| comp6456_c0_seq1 | 383 | T | T->A | T->A | T->A | - | - | - |
| comp27581_c1_seq1 | 112 | T | T->A | T->A | T->A | - | - | - |
| comp576472_c0_seq1 | 331 | T | T->A | T->A | T->A | - | - | - |
| comp17543_c0_seq1 | 101 | T | T->A | T->A | T->A | - | - | - |
| comp12777_c0_seq1 | 118 | T | T->A | T->A | T->A | - | - | - |
| comp29684_c0_seq1 | 1417 | T | T->A | T->A | T->A | - | - | - |
| comp16918_c0_seq1 | 111 | T | T->A | T->A | T->A | - | - | - |
| comp16768_c0_seq1 | 175 | T | T->A | T->A | T->A | - | - | - |
| comp16632_c0_seq1 | 404 | T | T->A | T->A | T->A | - | - | - |
| comp16632_c0_seq1 | 413 | T | T->A | T->A | T->A | - | - | - |
| comp958_c0_seq1 | 29 | T | T->A | T->A | T->A | - | - | - |
| comp958_c0_seq1 | 24 | T | T->A | T->A | T->A | - | - | - |
| comp1099562_c0_seq1 | 159 | T | T->A | T->A | T->A | - | - | - |
| comp1558_c0_seq1 | 13 | T | T->A | T->A | T->A | - | - | - |
| comp33021_c0_seq2 | 491 | T | T->A | T->A | T->A | - | - | - |
| comp36017_c0_seq1 | 1976 | T | T->A | T->A | T->A | - | - | - |
| comp25395_c1_seq1 | 623 | T | T->A | T->A | T->A | - | - | - |
| comp27539_c2_seq3 | 497 | T | T->A | T->A | T->A | - | - | - |
| comp9177_c0_seq1 | 97 | T | T->A | T->A | T->A | - | - | - |
| comp25395_c1_seq1 | 634 | T | T->A | T->A | T->A | - | - | - |
| comp34008_c2_seq6 | 24 | T | T->A | T->A | T->A | - | - | - |
| comp1051_c0_seq1 | 218 | T | T->A | T->A | T->A | - | - | - |
| comp16780_c0_seq1 | 353 | T | T->A | T->A | T->A | - | - | - |
| comp34008_c2_seq6 | 29 | T | T->A | T->A | T->A | - | - | - |
| comp1052985_c0_seq1 | 14 | T | T->A | T->A | T->A | - | - | - |
| comp1052985_c0_seq1 | 25 | T | T->A | T->A | T->A | - | - | - |
| comp36934_c0_seq3 | 327 | T | T->A | T->A | T->A | - | - | - |
| comp36828_c0_seq2 | 3890 | T | T->A | T->A | T->A | - | - | - |
| comp22382_c0_seq1 | 1039 | T | T->A | T->A | T->A | - | - | - |
| comp34466_c0_seq2 | 49 | T | T->A | T->A | T->A | - | - | - |
| comp34535_c0_seq8 | 3 | T | T->A | T->A | T->A | - | - | - |
| comp12016_c0_seq1 | 33 | T | T->A | T->A | T->A | - | - | - |
| comp36732_c1_seq8 | 62 | T | T->A | T->A | T->A | - | - | - |
| comp36746_c0_seq5 | 132 | T | T->A | T->A | T->A | - | - | - |
| comp36828_c0_seq2 | 2758 | T | T->A | T->A | T->A | - | - | - |
