# Supplementary material for: Integrated analysis of mRNA-seq and miRNA-seq reveals the potential roles of sex-biased miRNA-mRNA pairs in gonad tissue of dark sleeper (Odontobutis potamophila)
Source: BMC Genomics. 2017 Aug 14;18:613. doi: 10.1186/s12864-017-3995-9 (PMC5557427; doi:10.1186/s12864-017-3995-9)
Supplement: Supplementary file 14 — Information for validated SNPs. (DOCX 17 kb) [file 12864_2017_3995_MOESM14_ESM.docx]

**Table S11** Information for validated SNPs

| Transcript | SNP Position | SNP Type | **Forward primer (5′-3′)** | **Reverse primer (5′-3′)** |
| --- | --- | --- | --- | --- |
| comp18705_c0_seq2 | 548 | A->G | AGCTACAGACAACTTCCCACTT | CCCTATTCAAATTCTCCCAAA |
| comp36524_c0_seq1 | 576 | C->T | AAAGCCAAGATGAAGAACAAG | CTATGAGCAAAGAAAATGAAAAC |
| comp34847_c0_seq2 | 2665 | C->T | TCACAAGTAGCCCTGTAATCCA | AGTCCTCGACAACCAGCACAT |
| comp24389_c0_seq1 | 1828 | A->T | ACCGCATTGGCCTGACATT | AAGACTGGTTGAAGTAGTGGAGC |
| comp33772_c0_seq8 | 649 | G->A | CAGGATCAGTGACCTAACGAG | GGATGGTGCTTTACCTACTGC |
| comp34147_c0_seq1 | 825 | A->T | CGCTGGCAGGACATCTCTAT | GGGTTTGTGCAGGAAGAAGT |
| comp36615_c0_seq5 | 340 | A->T | CCAACCCAATATGCAGATGA | TGGTCCTGACAGACATTGCT |
| comp13782_c0_seq1 | 323 | A->C | GATGCAGGTCCTGGAGGATA | TCCTTGCCAAGCTCTTTCAC |
| comp35100_c0_seq4 | 508 | A->G | CCAACAGCTGTCGAGGAAAC | TGCAAAACATTTGGATGAGG |
| comp14863_c0_seq1 | 552 | C->A | CAGTACAGGCGATCCACCTT | CCTGCATGTCTGCATCATCT |
| comp36959_c0_seq2 | 516 | G->A | CGCGAGTCAATTGGAGAAAT | AGCAGACTCTGGCTCCAAAA |
| comp17613_c0_seq1 | 370 | T->G | ACGCTATGAACATGCAGCAG | CAAACCCCCTCTCCTTTCTC |
| comp15280_c0_seq1 | 314 | A->C | CAGCAGCGTCCTACAATTCA | GGCCTAGAGAAGGGGTCATT |
| comp35445_c0_seq1 | 1405 | G->T | GGCAGACATCCAAAGACCAC | CGATCCAAGTGTGAACCTCA |
| comp16366_c0_seq1 | 380 | G->A | ACCCCTAAACGGGACTAAGC | ACCCTTTGAGAGCCACCTTT |
| comp36139_c0_seq5 | 709 | T->G | TTCCCAATTGCTTGAAGGTC | TTCGACCCACGAATTAGTCC |
| comp16666_c0_seq1 | 437 | T->G | CTTGATGCTGCTGATGCAAT | GCATTCCGATGTGTTTCCTT |
| comp17031_c0_seq1 | 531 | T->A | TGGTCAAGTGTCCACCGTTA | ATTCTCCGCTAGGCAACAAA |
