# Supplementary material for: Integrated analysis of mRNA-seq and miRNA-seq reveals the potential roles of sex-biased miRNA-mRNA pairs in gonad tissue of dark sleeper (Odontobutis potamophila)
Source: BMC Genomics. 2017 Aug 14;18:613. doi: 10.1186/s12864-017-3995-9 (PMC5557427; doi:10.1186/s12864-017-3995-9)
Supplement: Supplementary file 15 — Histological section analysis of testis and ovary structure in dark sleeper with hematoxylin and eosin staining. (DOCX 1183 kb) [file 12864_2017_3995_MOESM15_ESM.docx]

**Fig. S4** Histological section analysis of testis and ovary structure in dark sleeper with hematoxylin and eosin staining


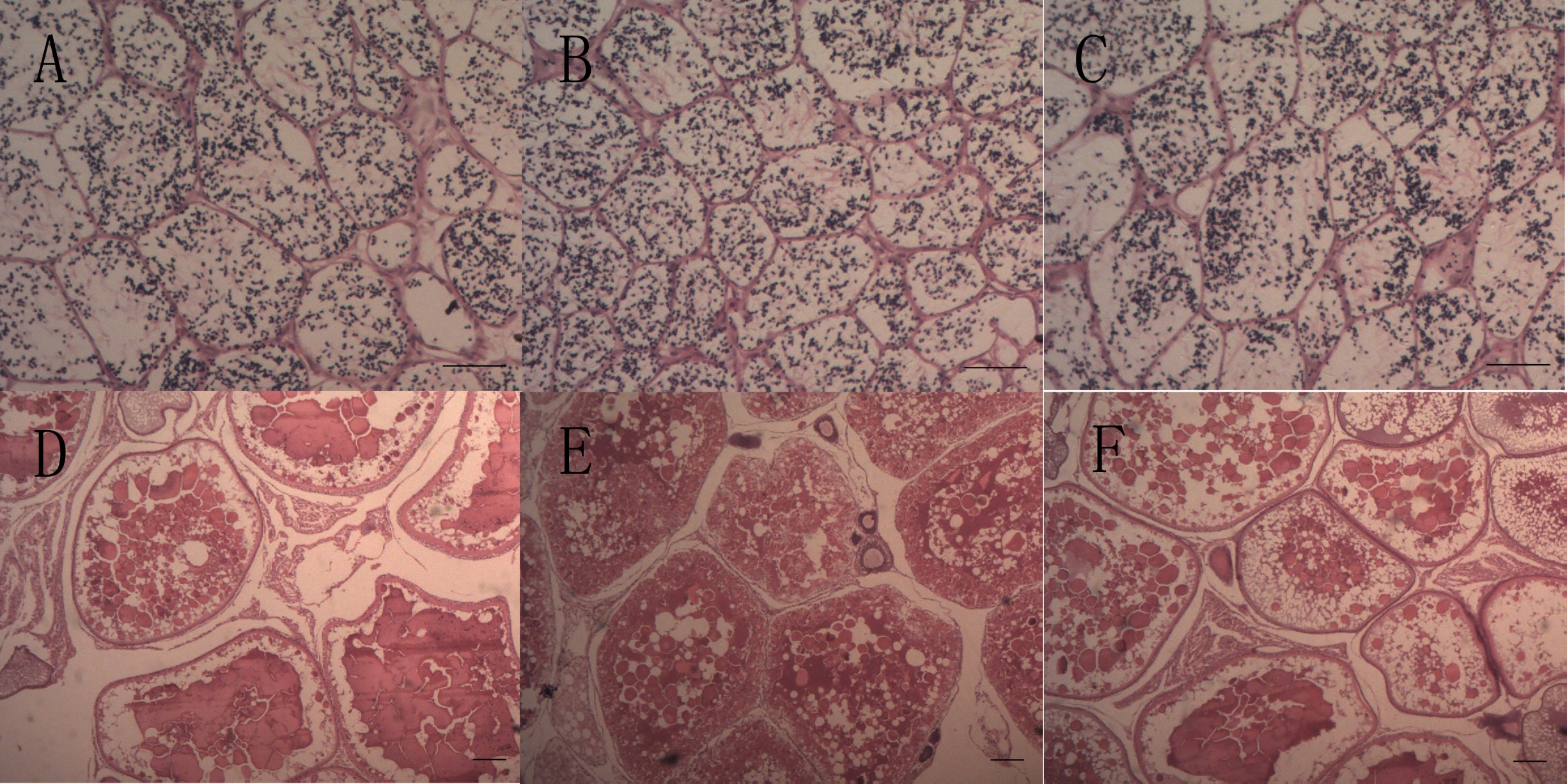


Histological features of the testis and ovary. Both gonads are in relatively late development stage (IV stage) and contain mostly spermatid and oocytes. A, B and C-testis, D E and F-ovary. Scale bars: 100 μm (A B C), 50 μm (D E F). A, B, C were from OTa, OTb, OTc; D, E, F were from OOa, OOb, OOc respectively.
